# EI24 Inhibits Cell Proliferation and Drug Resistance of Esophageal Squamous Cell Carcinoma

**DOI:** 10.3389/fonc.2020.01570

**Published:** 2020-08-21

**Authors:** Lili Duan, Jiaojiao Ma, Wanli Yang, Lu Cao, Xiaoqian Wang, Liaoran Niu, Yiding Li, Wei Zhou, Yujie Zhang, Jinqiang Liu, Hongwei Zhang, Qingchuan Zhao, Liu Hong, Daiming Fan

**Affiliations:** ^1^State Key Laboratory of Cancer Biology, National Clinical Research Center for Digestive Diseases and Xijing Hospital of Digestive Diseases, Air Force Military Medical University, Xi’an, China; ^2^Department of Endocrinology, The Second Affiliated Hospital of Xi’an Jiaotong University, Xi’an, China; ^3^Department of Biomedical Engineering, Air Force Military Medical University, Xi’an, China; ^4^Department of Digestive Diseases, Wuxi Mingci Cardiovascular Hospital, Wuxi, China

**Keywords:** EI24, esophageal squamous cell carcinoma, proliferation, drug resistance, prognosis

## Abstract

Drug resistance, whether intrinsic or acquired, often leads to treatment failure in esophageal squamous cell carcinoma (ESCC). Clarifying the mechanism of drug resistance in ESCC has great significance for reversing drug resistance, as well as improving the prognosis of patients. Previously, we demonstrated that etoposide-induced 2.4-kb mRNA (EI24) is the target of miR-483-3p, which promoted the growth, migration, and drug resistance in ESCC, suggesting that EI24 participates in repressing the tumorigenesis and progression of ESCC. Here, we observed that EI24 was remarkably decreased in ESCC tissues. Moreover, its expression was directly linked to the prognosis of patients. We then confirmed that the forced overexpression of EI24 repressed cell growth and sensitized ESCC cells to chemotherapeutic agents, whereas EI24 silencing had the opposite effect. Furthermore, gene microarray and ingenuity pathway analysis (IPA) were performed to establish the potential mechanisms and indicated that EI24 exerts a tumor-suppressive role via suppressing the acute phase response signaling pathway or IL-1 signaling pathway in ESCC. Collectively, our data reveal that EI24 overexpression attenuates malignant phenotypes of ESCC and that it is a novel possible ESCC therapeutic target.

## Introduction

Esophageal cancer (EC) is the seventh most prevalent malignant cancer globally, ranking the sixth regarding causes of cancer-linked fatalities (1). Approximately over 572,034 new EC cases, as well as 508,585 fatalities that occurred in 2018, are responsible for approximately 4% of cancer cases and fatalities (2). Esophageal adenocarcinoma (EAC) and esophageal squamous cell carcinoma (ESCC) are the two subtypes of EC. Of note, ESCC accounts for approximately 88% of EC patients (3). The initiation and development of ESCC present a gradual transformation process, from mild dysplasia to severe dysplasia, then to carcinoma *in situ*, and finally to be an invasive cancer (4). To our knowledge, several variables have been identified as predisposing risk factors for ESCC, including heavy smoking, low socioeconomic status, alcohol consumption, mycotoxin-contaminated or nitrosamine-rich foods, and nutritional deficiencies (2).

Since ESCC patients are largely asymptomatic, most of them were diagnosed at the advanced stage and were unsuitable for resection, resulting in elevated mortality and poor prognosis (5). Chemotherapy is identified as a relatively curative approach for ESCC patients who could not tolerate surgical resection or radiotherapy. Integrated chemotherapy for the therapy of ESCC was 5-fluorouracil (5-FU), cisplatin (CDDP), and adriamycin (ADR), or 5-FU, cisplatin, and paclitaxel. However, certain patients treated with neoadjuvant chemotherapy could not obtain better prognosis on account of the primary or acquired drug resistance (6). Therefore, identifying robust biosignatures for ESCC to reverse chemoresistance and improve clinical practice is urgent.

Etoposide-induced 2.4-kb transcript (EI24), additionally referred to as p53-induced gene 8 (PIG8), is located on human chromosome 11q23 (7). Serving as a p53 responsive pro-apoptotic factor, EI24 crucially participates in repressing cell growth, as well as activating autophagy (8, 9). Recent reports have posited that EI24 was downmodulated in invasive breast cancer and cervical carcinoma and significantly associated with poor prognosis (10, 11). In dedifferentiated liposarcoma and osteosarcoma, EI24 also exhibits a decreased expression level, implying that EI24 could serve as a tumor repressor gene (12, 13). Furthermore, diminished expression of EI24 contributes to the induction of epithelial-to-mesenchymal transition (EMT), as well as tumor growth (14). Besides, the loss of EI24 is linked to etoposide and gefitinib resistance, indicating that EI24 status may have great potential as a biomarker for chemotherapy responsiveness (15, 16). Previously, we demonstrated that miR-483-3p could enhance the growth, migration, and drug resistance in ESCC via targeting EI24, suggesting that EI24 probably participates in the tumorigenesis and progression of ESCC (17). These findings have been driving us to clarify the function of EI24 in ESCC proliferation and drug resistance and to explore the underlying molecular mechanisms.

Herein, we established that EI24 was downmodulated in ESCC and that decreased EI24 expression was related to poor prognosis in ESCC patients. Furthermore, EI24 silencing enhanced the proliferation of ESCC cells and the growth of implanted tumors and repressed cell apoptosis but enhanced chemotherapy resistance to 5-FU, CDDP, ADR, and vincristine (VCR), whereas EI24 overexpression exhibited opposite effects. Ingenuity pathway analysis (IPA) indicated that EI24 may exert its tumor-suppressive role through regulating the acute phase response signaling pathway or IL-1 signaling pathway in ESCC.

## Materials and Methods

### Cells and Cell Culture

We obtained the human ESCC cell lines EC109 and EC9706 from Basic Medical Cell Center, the Institute of Basic Medicine, Chinese Academy of Medical Sciences. The human ESCC cell lines KYSE150, TE-1, and Eca109 were acquired from the Cell Resource Center of Shanghai Institute of Life Sciences, Chinese Academy of Sciences. We passaged the cells and stored them at −80°C in our laboratory for not more than 6 months after acquisition. We performed all the study experiments within eight passages of resuscitation. We grew all the cells in Roswell Park Memorial Institute (RPMI)-1640 medium (Invitrogen Life Technologies, Carlsbad, CA, United States), added with 10% fetal bovine serum (Invitrogen Life Technologies), 100 U/ml of penicillin, and 100 μg/ml of streptomycin. The growth of cells was carried out in a humid incubator (5% CO_2_ at 37°C).

### RNA Isolation and qRT-PCR

Total RNA extraction from the tissue samples was implemented using the TRIzol reagent (Invitrogen, Waltham, MA, United States). We conducted reverse transcription of these tRNAs to obtain cDNA employing the PrimeScript RT Reagent Kit (TaKaRa, Tokyo, Japan) as outlined by the manufacturer. After that, we implemented qRT-PCR to examine the expression levels of EI24 utilizing the SYBR Premix Ex Taq II Kit (TaKaRa, Tokyo, Japan). The qRT_PCR process was implemented on the ABI StepOne Real-Time PCR system. We utilized the GAPDH as an internal standard. The primer sequences were EI24 forward: 5′-TTCCTGTGCTTCAGTCGGTA-3′; reverse: 5′-GGCTTCCTCCCTGATACCT-3′; GAPDH forward: 5′-GACAG TCAGCCGCATCTTCT-3′; reverse: 5′-GCGCCCAATACGACC AAATC-3′. Finally, we employed the comparative 2^–ΔΔCt^ approach to establish the relative mRNA expression.

### Western Blot Assessment

We lysed the cells in radioimmunoprecipitation assay (RIPA) buffer added with protease repressors on ice. Then, we centrifuged the lysates at 12,000 *g* for 15 min. Subsequently, employing the BCA Protein Assay Kit (Pierce, Rockford, IL, United States), we assessed the protein concentration. Employing 10% sodium dodecyl sulfate–polyacrylamide gel electrophoresis (SDS-PAGE), we separated an equal volume of the proteins, which we transfer-embedded onto nitrocellulose membranes (Millipore). Then, we blocked the membranes with 5% non-fat milk, followed by conjugation with primary antibodies against EI24 (#ab130957, Abcam), GAPDH (#ab8245, Abcam), MDR1 (#13342, Cell Signaling Technology), ABCG2 (#42078, Cell Signaling Technology), cyclin-dependent kinase (CDK) 2 (#18048, Cell Signaling Technology), CDK4 (#12790, Cell Signaling Technology), cyclin D1 (#55506, Cell Signaling Technology), cleaved caspase-3 (#9664, Cell Signaling Technology), cleaved caspase-9 (#20750, Cell Signaling Technology), and β-actin (#3700, Cell Signaling Technology) via incubation overnight at 4°C. Subsequently, the membranes were conjugated with horseradish peroxidase (HRP)-labeled secondary anti-mouse IgG or anti-rabbit IgG antibodies (Abcam) for 1 h at 37°C. We purchased all the antibodies from Abcam Inc. (Cambridge, MA, United States). Protein bands visualization was implemented on an enhanced chemiluminescence detection system (Pierce) and analyzed by Image J software.

### Cell Transfection

CRISPR-Cas9 gene editing approach was used to knockdown EI24 in ESCC cells; and the following two single-guide RNAs (sgRNAs) were used: sgEI24-1: 5′-AAAATTCTACTAACAATA CG-3′; sgEI24-2: 5′-TCGAATCCAGCAAAAGAGAG-3′; sgEI24-3: 5′-CCTGTGTGTAGTTGATAGTT-3′. The sgRNA/Cas9 dual-expression vector was introduced by lentiviral transduction and was transiently transfected into KYSE150 and TE-1 cell lines. For excessive expression of EI24, we purchased the respective lentivirus expression vector from GeneChem Bio-Medical Biotechnology (Shanghai, China). We seeded 5 × 10^4^ cells in 6-well plates, followed by transfection with expression vectors employing Lipofectamine 2000 (Invitrogen) as outlined in the protocol of the manufacturer. Stable clones were selected with puromycin, and then we confirmed the transfection efficiency via Western blot assessment.

### MTT Assessment

We conducted the 3-(4,5-dimethylthiazol-2-yl)-2,5-diphenyl-tetrazolium bromide (MTT) test to inspect cell proliferation. We seeded the ESCC cells in 96-well plates at 1 × 10^4^ cells/well. After overnight incubation, we added a total of 20 μl of MTT (5 mg/ml) to each well. Then, after 4-h incubation, medium was replaced by 150 μl of DMSO to facilitate the dissolving of the MTT formazan crystals. After that, establishing the absorbance was implemented at 490 nm.

### *In vitro* Drug Sensitivity Assay

Drug resistance was determined via the Cell Counting Kit-8 (CCK-8) assessment. We seeded the ESCC cells into 96-well plates (2 × 103 cells/well) and left them standing overnight for the cells to attach. Before each experiment, we freshly prepared 5-FU, CDDP, VCR, and ADR. After adhesion, cells were then exposed to these antitumor drugs at various concentrations selected in preliminary experiments. After 48 h, we added 10 μl of CCK-8 solution (Dojindo, Japan) to each well and then grew the cells for another 2 h. Then, using a microplate reader, we determined the absorbance at 450 nm. We computed cell viability (%) as cell viability (%) = (1 − OD_drug_/OD_control_) × 100.

### Colony Formation Assay

We uniformly dispersed the ESCC cells suspension (1,000 cells), seeded in 6-well plates, and then grown for over a span of 2 weeks in 5% CO_2_ incubator under 37°C. Subsequently, we fixed the cells with 10% formalin for 15 min, and then we performed 0.1% crystal violet staining for 30 min.

### Cell Cycle Assay

We harvested the seeded stable transfected ESCC cells in the six-well plates that had attained the log phase via trypsinization. Then, the cells were rinsed in phosphate-buffered saline (PBS) buffer. We then fixed the samples in 70% ethanol for cell cycle assessment, followed by staining using 0.5% propidium iodide (PI) (Servicebio), added with 0.01% RNaseA. We utilized the flow cytometer (CytoFLEX, Beckman Coulter, Brea, CA, United States) to implement cell cycle analysis.

### Flow Cytometry

Apoptosis was inspected employing the Annexin V-FITC Apoptosis Detection Kit (Beyotime, China). We harvested the ESCC cells from the various treatments, followed by centrifugation at 1,500 rpm for 5 min and subsequent resuspension in PBS. We discarded the supernatant and accomplished cell resuspension in 1 × Annexin V binding buffer (195 μl). We added Annexin-V/FITC (5 μl) to the cell suspension. After that, we added PI (10 μl) to the cells, followed by room temperature (RT) incubation in the dark for 15 min. Cell apoptosis determination was implemented on the FACSCalibur cytometer (Becton Dickinson, United States).

### Intracellular Adriamycin Concentration Analysis

Flow cytometry was implemented to inspect the intracellular accumulation and retention of adriamycin. We seeded the ESCC cells harvested at the logarithmic growth phase into 6-well plates at 1 × 10^5^ cells/well density and grown overtime at 37°C. We added adriamycin to all culture wells at a final 5 mg/ml concentration, and the incubation continued for an extra 1 h. After that, cells either were harvested for detecting intracellular accumulation of adriamycin or were transferred to adriamycin-free culture medium for another 1-h incubation to detect adriamycin retention. We deduced intracellular adriamycin fluorescence intensity via flow cytometry. This was implemented at an excitation wavelength of 488 nm and emission wavelength of 575 nm. We computed the releasing index of adriamycin as (accumulation value - retention value)/accumulation value.

### Xenograft Experiments

Balb/c mice (4–6 weeks) were acquired from Shanghai Experimental Animal Center of the Chinese Academy of Sciences (Shanghai, China). We raised these mice under specific pathogen-free (SPF) conditions. The Animal Research and Care Committee of Air Force Military Medical University ratified all the experimental animal procedures. We created the xenograft tumor models via subcutaneous injection of 4 × 10^6^ EC9706/EI24-OE, EC9706/EI24-NC, KYSE150/EI24-KO, and KYSE150/EI24-NC cells (*n* = 6 per group). We measured the tumor size every 2 days, and we employed the formula volume = (length × width^2^)/2 to compute the volume (16).

### Immunohistochemistry

We purchased the human ESCC tissue microarray (TMA) from Shanghai Xinchao Biotechnology (Shanghai, China) (Lot No. HEso-Squ180Sur-01). It contained 95 ESCC and 85 paratumor tissues collected from patients who underwent radical esophagectomy between January 2006 and October 2008. All these patients were followed up for 6–8 years. The ethics committee of Xijing Hospital, Air Force Military Medical University, ratified the study. We deparaffinized the TMA slides using xylene, followed by graded alcohol rehydration. Following the retrieval of antigens, 3% H_2_O_2_ was employed to block the activity of the endogenous peroxidase. Subsequently, blocking of the slices using 10% goat plasma was conducted, followed by 0.1% Triton X-100 incubation. Thereafter, overnight anti-EI24 antibody (1:200, Abcam, Cambridge, MA, United States) conjugation at 4°C was accomplished in a humidified chamber. After conjugation with biotinylated secondary antibody and HRP, we assessed the sections using DAB and then performed hematoxylin counterstaining. The fluorescence images were acquired on a fluorescence microscope (Carl Zeiss, Jena, Germany). We assessed the histochemical score (H-score) to establish the staining intensity and the percentage of stained cells. We scored the staining intensity as follows: 0 (no staining), 1 (weakly staining), 2 (moderate staining), and 3 (strong staining). The percentage of stained cells was scored on a scale of 0 to 4: 0 (no staining, 0%), 1 (staining range, 1–25%), 2 (staining range, 26–50%), 3 (staining range, 51–75%), or 4 (staining range, 76–100%). The final H-score was computed by multiplying values acquired from staining intensity and percentage of stained cells. H-score ≤ 4 was regarded as low expression, while >4 score was considered as upregulated. Analyses were performed by three independent senior pathologists who were blinded to the clinical outcome, and any differences can be resolved in a common interpretation.

### cDNA Microarray Test and Ingenuity Pathway Analysis

cDNA microarray assay was conducted to profile the expression of EI24-modulated genes. RNA purification from the EC109/EI24-OE, TE-1/EI24-KO, and corresponding control cells was accomplished using the TRIzol reagent (Invitrogen). The concentration of the RNA concentration and purity were examined on a Nanodrop 2000 (Thermo Fisher Scientific, Waltham, MA, United States). Moreover, the RNA integrity was inspected on an Agilent Bioanalyzer 2100 (Agilent Technologies, Santa Clara, CA, United States). Then, the quality-checked RNA samples were submitted to GeneChem (Shanghai, China) for labeling using the GeneChip 3′ IVT Express Kit (Affymetrix, Santa Clara, CA, United States) and hybridization using Affymetrix GeneChip PrimeView Human Gene Expression array (Affymetrix). Microarray data were analyzed by GeneChip Scanner 3000 (Affymetrix). A fold change ≥1.5 or ≤-1.5 in expression and *P* < 0.05 (false discovery rate (FDR) < 0.05) were employed as cutoff to deduce differentially expressed genes (DEGs). IPA (Ingenuity Systems; www.ingenuity.com; Redwood City, CA, United States), an online software package, is carried out to deduce gene networks, functions, and canonical cascades linked to DEGs. *Z*-score, a statistical index of IPA, demonstrates whether the results are consistent with experiments and results mentioned in the literatures. |*Z*| > 2 represents activation or repression of a network/cascade.

### Statistical Computations

We implemented all the statistical computations on the IBM SPSS Statistics 22.0 software. The correlation between EI24 expression in ESCC tissues and that in paratumor tissues was analyzed by Mann–Whitney test. Chi-square test was used to analyze the connection linking EI24 expression and the clinicopathological parameters of ESCC patients. The Kaplan–Meier approach was utilized to draw the survival curve of patients with different expression of EI24, and log-rank assessment was employed to inspect the connection linking EI24 expression and the prognosis of ESCC. Two-tailed Student *t*-test was performed to inspect the differences between variables in continuous variable groups.

## Results

### EI24 Expression Is Decreased in Esophageal Squamous Cell Carcinoma and Is Closely Related to Prognosis

**FIGURE 1:**
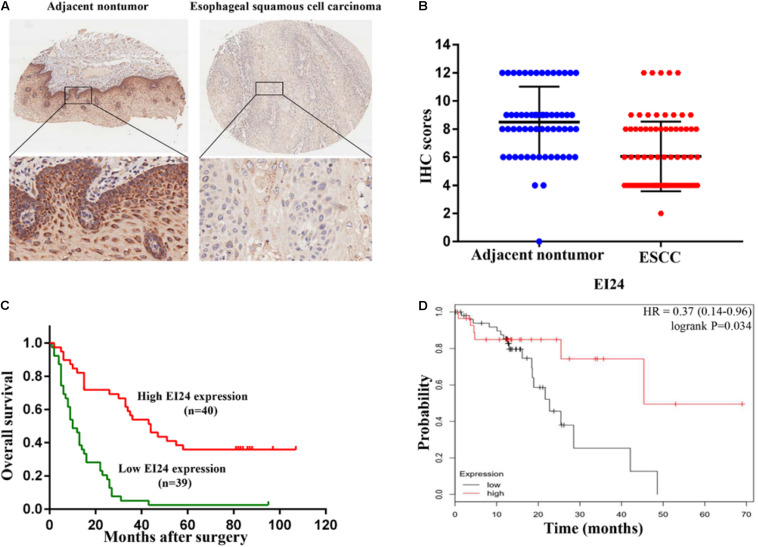
Clinical relevance of EI24 expression in esophageal squamous cell carcinoma (ESCC). **(A,B)** Representative immunohistochemical (IHC) staining of EI24 expression and IHC scores of ESCC tissues and paratumor tissues. **(C)** The Kaplan–Meier curves for overall survival according to the EI24 expression (log-rank test; *P* < 0.001). **(D)** Patients with higher EI24 expression had a better survival than patients with lower EI24 expression.

To establish if EI24 was unusually expressed in ESCC, estimation of the expression profile of EI24 in ESCC tissues was conducted by employing TMAs involving 95 cases of human ESCC and 85 cases of corresponding paratumor tissues. Immunohistochemistry posited that EI24 protein was primarily expressed in the cytoplasm of ESCC cells (Figure 1A). The analysis results of Mann–Whitney test pointed that the content of EI24 was remarkably diminished in ESCC tissues relative to the paratumor tissues (*P* < 0.001) (Figure 1B). Furthermore, we inspected the connection linking EI24 expression and ESCC clinicopathological manifestations in 93 cases of ESCC. The median EI24 expression of all ESCC tissues was employed as the cutoff value to group the ESCC patients into two clusters. Chi-square assessment posited that elevated EI24 protein expression was negatively linked to tumor size, American Joint Committee on Cancer (AJCC) stage, T stage, and lymph node metastasis and not linked to the patient’s age, gender, and pathological grade (Table 1). As for survival, log-rank test disclosed that patients with higher EI24 expression exhibited favorable overall survival (OS) (*P* = 0.001) (Figure 1C). The online Kaplan–Meier plotter tool also confirmed that patients who had lower EI24 expression had a poor survival relative to the patients with elevated EI24 expression [hazard ratio (HR) = 0.37; 95% confidence interval (CI), 0.14–0.96, *P* = 0.034] (Figure 1D). Univariate logistic regression assessment pointed out that the T stage, lymph node metastasis, AJCC stage, and EI24 expression were related to the OS of ESCC patients, and multivariate logistic regression assessment posited that decreased EI24 expression and advanced AJCC stage were shown to be independent predictors of worse survival (Table 2).

**TABLE 1:** Correlation of EI24 expression in 135 cases of esophageal squamous cell carcinoma (ESCC) tissue with patients’ clinicopathological variables.

Clinicopathological variables	Tumor EI24 expression			*P*-value
	All cases *n* = 79	Low expression *n* = 39	High expression *n* = 40	
**Age**				0.257
≤65.5^a^	25	10	15	
>65.5	54	29	25	
**Gender**				0.468
Male	60	31	29	
Female	19	8	11	
**Pathological grade**				0.794
I	5	2	3	
II	54	26	28	
III	20	11	9	
**Tumor size (cm)**				0.003
≤5	39	18	21	
>5	40	34	6	
**Tumor invasion**				0.006
T1	5	0	5	
T2	9	1	8	
T3	62	36	26	
T4	3	2	1	
**Lymph node metastasis**				<0.001
Absent	39	11	28	
Present	40	28	12	
**American Joint Committee on Cancer (AJCC) stage**				<0.001
Stage I	5	0	5	
Stage II	35	11	24	
Stage III	39	28	11	

*^*a*^Mean age at operation.*

**TABLE 2:** Univariate and multivariate analysis of factors associated with survival of esophageal squamous cell carcinoma (ESCC) patients.

Variables	Case number^a^	Survival					
		Univariate analysis^b^			multivariate analysis^b^		
		HR	95% CI	*P*-value	HR	95% CI	*P*-value
Age (≤65.8 versus >65.8)	25/54	0.943	0.562–1.584	0.825			
Gender (male versus female)	60/19	0.512	0.267–0.984	0.045	0.493	0.244–0.997	0.049
Pathological grade (I/II versus III)	59/20	0.907	0.329–2.502	0.850			
Tumor size (≤5 versus >5)	39/40	1.656	0.990–2.769	0.055			
Tumor invasion (T1–T2 versus T3–T4)	14/65	2.386	1.132–5.030	0.022	0.951	0.408–2.219	0.908
Lymph node metastasis (absent versus present)	39/40	2.261	1.359–3.763	0.002	0.222	0.046–1.079	0.062
American Joint Committee on Cancer (AJCC) stage (I–II versus III–IV)	40/39	3.068	1.814–5.189	<0.001	6.943	1.458–33.063	0.015
EI24 expression (low versus high)	39/40	0.239	0.137–0.415	<0.001	0.271	0.141–0.522	<0.001

*^*a*^Analysis was conducted on 79 cases shown in Table 1. ^*b*^ Hazard ratios [95% confidence interval (CI)] and *P*-values were calculated using univariate or multivariate Cox proportional hazard regression.*

### EI24 Represses the Growth of Esophageal Squamous Cell Carcinoma Cells *in vitro* and *in vivo*

To examine the function of EI24 in ESCC, first, we examined the protein expression of EI24 endogenously in five tumor-derived ESCC cell lines (EC9706, KYSE150, TE-1, EC109, and Eca109) by Western blotting and found that EC9706 and EC109 cells had lower levels of EI24 protein relative to the other cells, while KYSE150 and TE-1 cells expressed EI24 at a much higher level (Supplementary Figure S1A). Therefore, we selected EC9706, EC109, KYSE150, and TE-1 cells for the subsequent studies.

**FIGURE 2:**
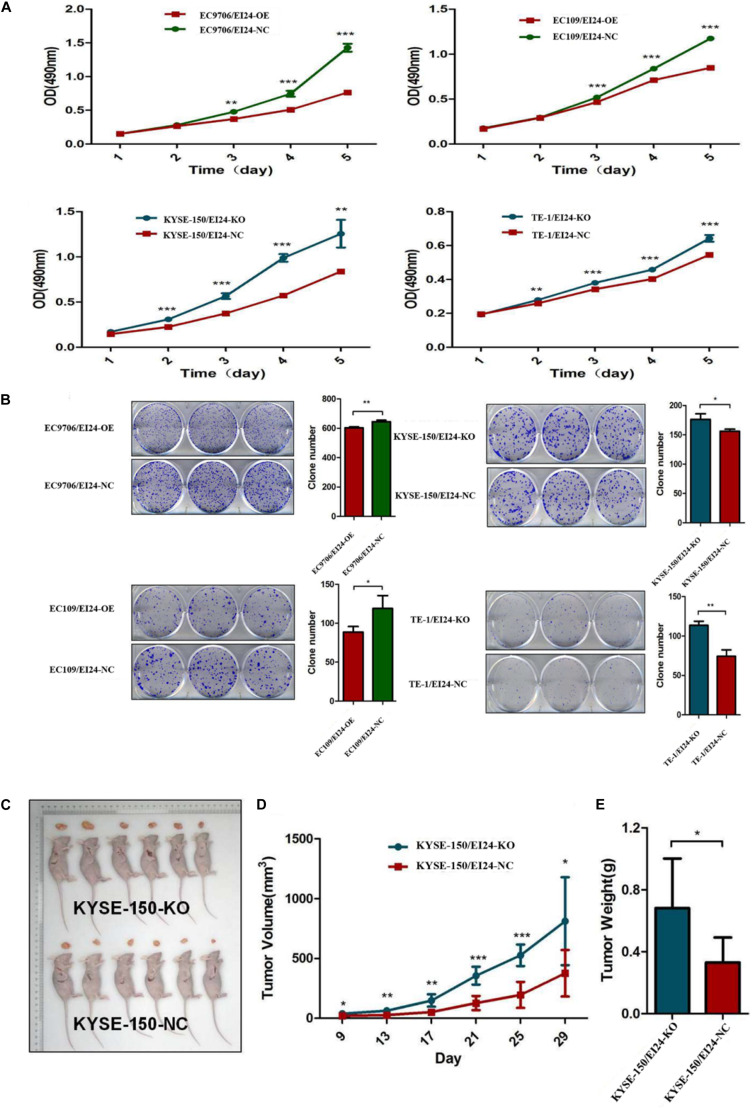
Effects of EI24 knockdown or overexpression on cell proliferation *in vitro* and *in vivo*. **(A,B)** MTT proliferation assay and colony formation assay were used for detecting the proliferation ability in EI24-knockdown or EI24-overexpressing ESCC cell lines; EI24 silencing significantly promoted the growth of xenografts in comparison with the NC group. Cells were injected into the hindlimbs of nude mice (*n* = 6). **(C)** Representative images of tumor-bearing mice and tumors isolated from tumor-bearing mice. **(D)** Tumor growth curves were measured during the growth of the tumors. Tumors derived from KYSE150 cells expressing EI24-KO grew significantly faster than those from cells with EI24-NC. **(E)** Final tumor weights were measured in each group. *P < 0.05, **P < 0.01, ***P < 0.001.

We established stable EI24-knockout (KO) KYSE150, as well as TE-1 cells by using CRISPR-Cas9 technology as well as EI24-overexpressed (OE) EC9706 and EC109 cells via transfecting lentiviral vectors. Our Western blot and qRT-PCR assessments pointed out that Cas9 and EI24-specific sgRNA markedly silenced intracellular EI24 protein relative to the negative control (EI24-NC), while the EI24-OE arm had a remarkably elevated level of EI24 protein (Supplementary Figures S1B–E). MTT assay demonstrated that cell proliferation activity was remarkably increased in EI24-silenced cells but was decreased in ectopic-EI24-expressing cells (Figure 2A). Consistently, the colony-forming potential was distinctly increased in KYSE150/EI24-KO and TE-1/EI24-KO cells relative to the EI24-NC cells but was remarkably suppressed in EC9706/EI24-OE and EC109/EI24-OE cells (Figure 2B).

To verify the prospective influence of EI24 depletion on ESCC cell growth *in vivo*, KYSE150/EI24-KO cells and KYSE150/EI24-NC cells were subcutaneously injected in nude mice. Consequently, tumors in mice implanted in KYSE150/EI24-KO cells grew faster relative to the control cells. EI24 silenced cells had remarkably higher tumor volume and weight relative to the control cells 29 days after the injection (Figures 2C–E). These data provided support that EI24 is a distinctive determinant for ESCC cell growth.

**FIGURE 3:**
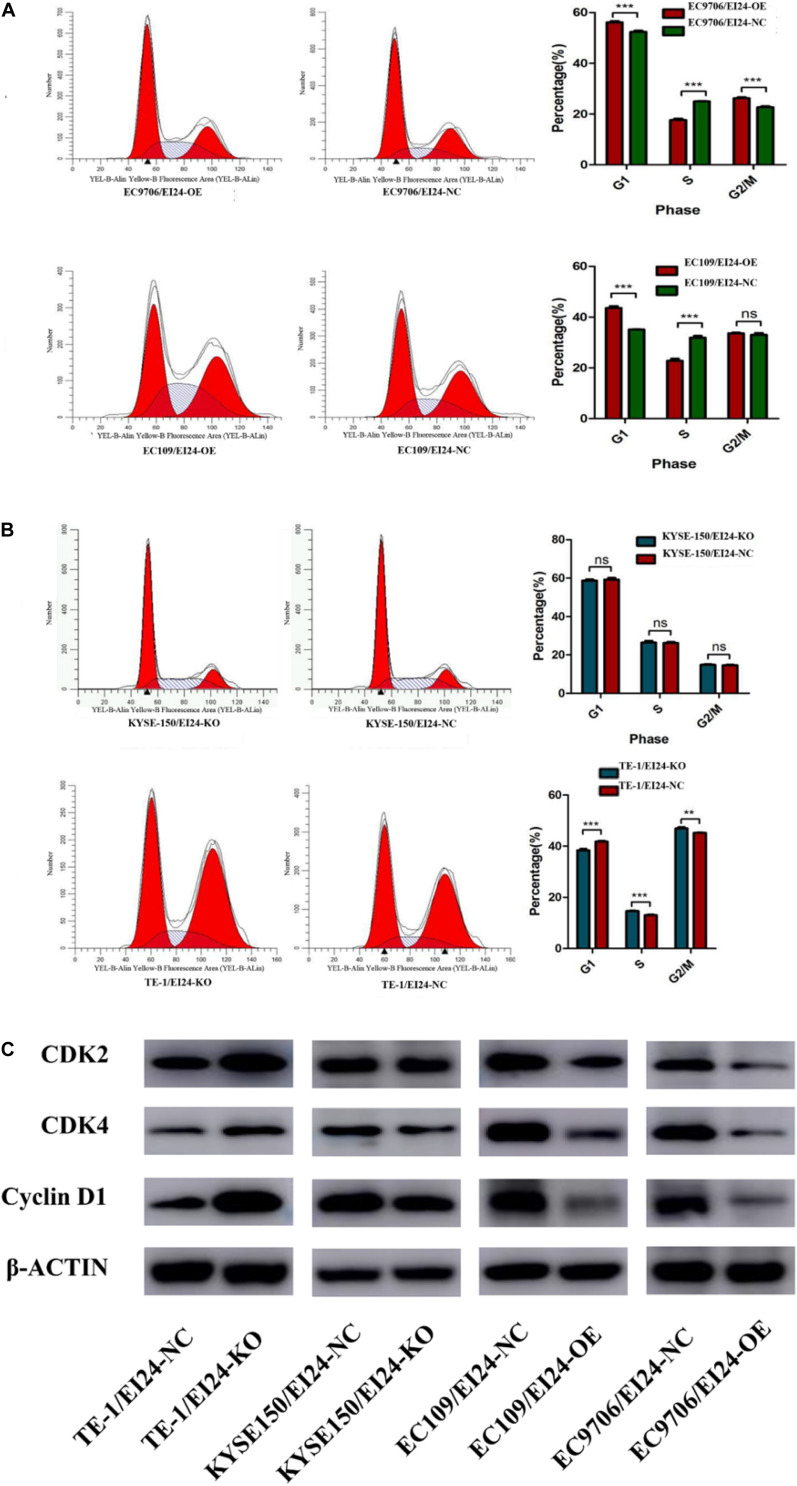
Effects of EI24 knockdown or overexpression on cell cycle progression in esophageal squamous cell carcinoma (ESCC) cells. Cell-cycle analysis revealed that ectopic expression of EI24 in EC9706 and EC109 cells increased the percentage of cells in the G1 phase and decreased the percentage of cells in S phase **(A)**, while knocking down EI24 expression in TE-1 cells decreased the percentage of cells in the G1 phase and increased the percentage in S phase **(B)**. **(C)** Western blot analysis of the expression of CDK2, CDK4, and cyclin D1 in ESCC cells. CDK2, CDK4, and cyclin D1 expression were decreased in EI24-OE cells compared with EI24-NC cells. EI24 depletion reversed this effect on CDK2, CDK4, and cyclin D1 expression. β-Actin was used as an internal control. *P < 0.05, **P < 0.01, ***P < 0.001.

Besides, we conducted flow cytometry assessments to verify whether the alteration of cell growth is linked to changes in the cell cycle profile. Consequently, the results pointed out that ectopic expression of EI24 in EC9706 and EC109 cells induced G1/S arrest (Figure 3A) while EI24 knockdown in TE-1 cells enhanced the cell cycle progression into the S phase (Figure 3B). However, there was no remarkable difference in the cell cycle profile of KYSE150/EI24-KO cells compared with the controls, which may be due to gene expression heterogeneity among cells. On the basis of the findings of cell cycle distribution, we then investigated whether the G1/S arrest induced by EI24 was linked to the expression of cell cycle-correlated genes such as CDK2, CDK4, and cyclin D1, which are responsible for cell cycle progression from G1 to S phase (18). Western blot evaluations disclosed that the forced expression of EI24 markedly suppressed the expression of CDK2, CDK4, and cyclin D1, whereas EI24 depletion exhibited opposite effects (Figure 3C). Consistent with the results obtained by flow cytometry, no significant differences in the expression of cell cycle-correlated genes were observed between KYSE150/EI24-KO cells and KYSE150/EI24-NC cells. These data implied that EI24 suppresses cell growth at least partly by repressing G1/S transition in ESCC cells.

### EI24 Attenuates Chemotherapy Resistance of Esophageal Squamous Cell Carcinoma Cells and Promotes Cell Apoptosis

**FIGURE 4:**
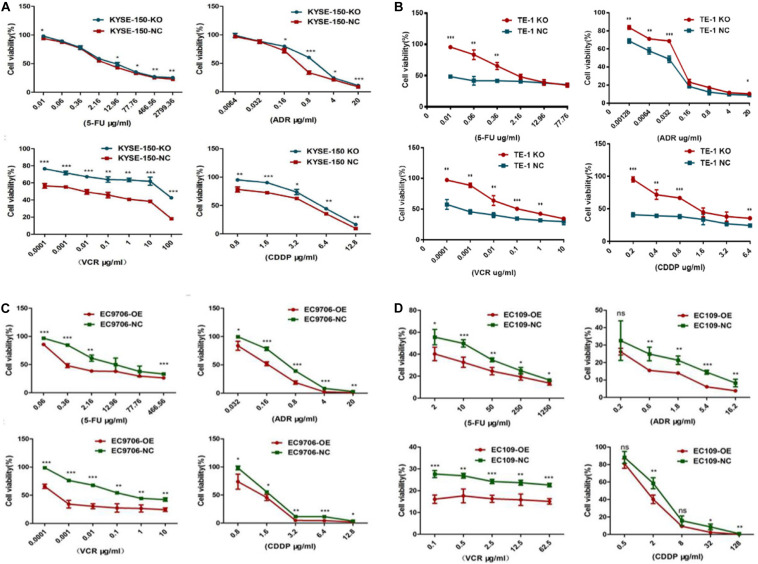
EI24 promotes chemosensitivity of esophageal squamous cell carcinoma (ESCC) cells to 5-fluorouracil (5-FU), adriamycin (ADR), vincristine (VCR), and cisplatin (CDDP). **(A–D)** ESCC cells were treated with the indicated concentrations of chemotherapeutic agents (5-FU, ADR, VCR, and CDDP) for 48 h before cell viability was measured by the Cell Counting Kit-8 (CCK-8) assay. *P < 0.05, **P < 0.01, ***P < 0.001.

We then inspected the influence of EI24 on the tumor cell responses to a wide range of chemotherapy drugs (5-FU, ADR, VCR, and CDDP) that have been used for treatment of ESCC. As shown in Figures 4A,B, the EI24-knockdown ESCC cells displayed decreased sensitivity to chemotherapeutic reagents with a dramatic increase in cell viability at various drug concentrations tested in comparison with control cells. Furthermore, we tested whether the overexpression of EI24 would alter chemotherapeutic reagents sensitivity in ESCC cell lines. Interestingly, results of CCK8 assessments pointed out that ectopic expression of EI24 distinctly reduced cell viability (Figures 4C,D).

**FIGURE 5:**
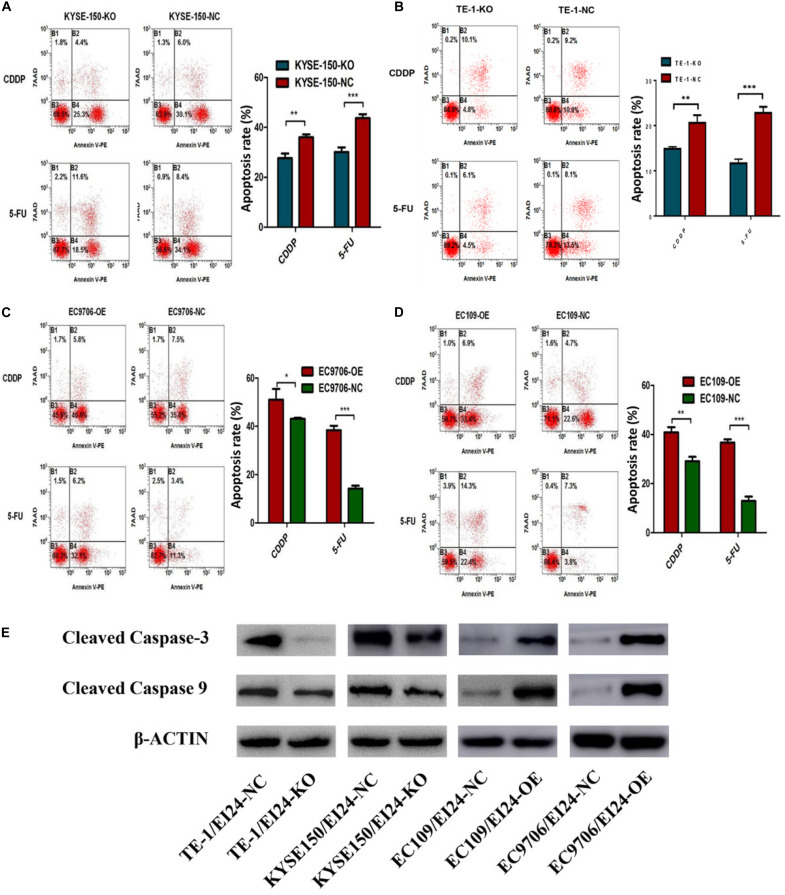
Effects of EI24 knockdown or overexpression on apoptosis in esophageal squamous cell carcinoma (ESCC) cells. **(A–D)** Flow cytometric analysis of the role of EI24 in 5-fluorouracil (5-FU) or cisplatin (CDDP)-induced apoptosis. **(A,B)** EI24 depletion significantly decreased the proportion of apoptotic cells in EI24-KO cells compared with controls. **(C,D)** Overexpression of EI24 significantly increased the proportion of apoptotic cells in EI24-OE cells compared with controls. **(E)** Western blot analysis of the expression of cleaved caspase-3 and cleaved caspase-9 in ESCC cells. Cleaved caspase-3 and cleaved caspase-9 expressions were decreased in EI24-KO cells compared with EI24-NC cells. EI24 overexpression reversed this effect on cleaved caspase-3 and cleaved caspase-9 expression. β-Actin was used as an internal control. *P < 0.05, **P < 0.01, ***P < 0.001.

Notably, the dysregulation of drug-induced apoptosis is significantly associated with drug resistance in multiple cancers (19). We then explored whether knocking down EI24 could influence 5-FU or cisplatin-induced apoptosis in KYSE150 cells and TE-1 cells using flow cytometry. As depicted in Figures 5A,B, depleted EI24 potentiated apoptosis of KYSE150 cells and TE-1 cells induced through low-concentration 5-FU (10 μg/ml) or cisplatin (5 μg/ml). Opposite effects on cell apoptosis induced by 5-FU (50 μg/ml) or cisplatin (20 μg/ml) were observed in ectopic-EI24-expressing EC9706 and EC109 cells (Figures 5C,D). Moreover, apoptotic markers, including cleaved caspase-3, as well as cleaved caspase-9, were downmodulated in EI24-KO cells but upregulated in EI24-OE cells, which was congruent with the above results (Figure 5E).

**FIGURE 6:**
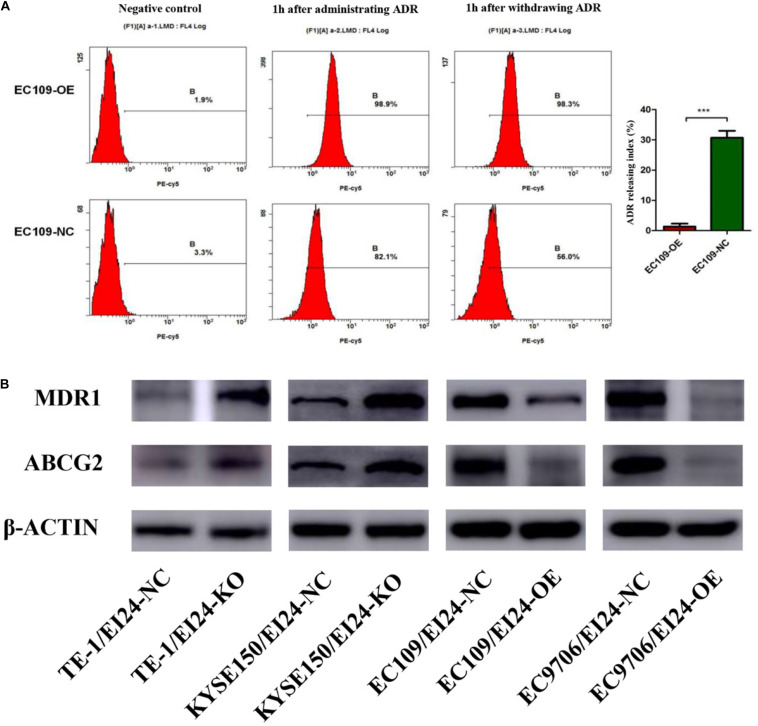
EI24 overexpression effects on drug accumulation of esophageal squamous cell carcinoma (ESCC) cells. **(A)** Adriamycin (ADR) accumulation in EC109/EI24-OE and EC109/EI24-NC cells was measured by flow cytometry. **(B)** Western blot analysis of the expression of MDR1 and ABCG2 in ESCC cells. MDR1 and ABCG2 expressions were increased in EI24-KO cells compared with EI24-NC cells. EI24 overexpression reversed this effect on MDR1 and ABCG2 expression. β-Actin was used as an internal control. *P < 0.05, **P < 0.01, ***P < 0.001.

Since elevated drug efflux has been extensively thought as a crucial process in MDR (20), we hypothesized that EI24 affected the chemoresistance of ESCC cells through regulating drug efflux. Afterwards, we carried out the drug efflux assay in ectopic-EI24-expressing EC109 cells. Flow cytometry analysis showed that EI24 overexpression markedly increased intracellular ADR accumulation and retention rates in EC109/EI24-NC cells compared with EC109/EI24-OE cells (Figure 6A). Notably, ATP-binding cassette transporters (ABC transporters) are essential for the efflux of excessive intracellular drugs, thus giving rise to a significant impairment of chemotherapeutic effects (21). Western blot assessments revealed that ATP Binding Cassette Subfamily B Member 1 (also known as MDR1), as well as ATP Binding Cassette Subfamily G Member 2 (ABCG2) expression, was upregulated in EI24-KO cells but downregulated in EI24-OE cells (Figure 6B). The data verified the ability of EI24 to efficiently alleviate drug efflux through regulating the expression of MDR1 and ABCG2, which might be directly associated with the drug resistance.

### The Global Gene Expression Profile Mediated by EI24

The Affymetrix GeneChip PrimeView Human cDNA microarray evaluation was conducted to profile the expression of EI24-modulated genes in ESCC cells. The results revealed that EI24 overexpression in EC109 cells led to remarkable differences in the expression of 1,197 genes (FDR < 0.05 for |fold change| > 1.5), with 197 upregulated genes and 1,000 downregulated genes (Supplementary Figures S2A–D and Supplementary Data Sheet 1). Additionally, we carried out microarray analysis to identify gene-expression changes in TE-1 cells with EI24 knockdown. The findings pointed out that the expressions of 1,353 genes were upregulated and those of 1,290 genes were downregulated compared with those in control cells (FDR < 0.05 for |fold change| > 1.5) (Supplementary Figures S2A–D and Supplementary Data Sheet 2). We further have drawn the Venn diagrams of DEGs between the EC109 cells and TE-1 cells (Supplementary Figure S2B).

### Functional Analysis of Differentially Expressed Genes Relative to Classical Pathways, Upstream Regulators, and Disease

To evaluate the potential mechanisms of the proliferative and multidrug resistance functions of EI24, those DEGs obtained from gene microarray analysis were uploaded to IPA database, which is commonly used to combine DEGs with canonical pathways, associated networks, and functions (22).

**FIGURE 7:**
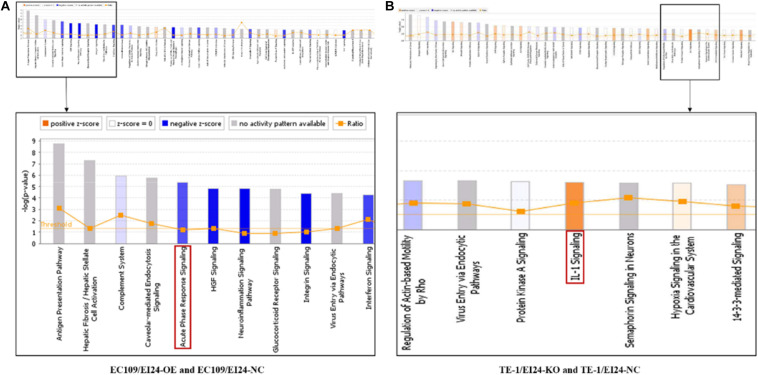
Ingenuity pathway analyses of differentially expressed genes (DEGs). **(A,B)** Histograms represented enrichment analysis of signaling pathways regulated by DEGs, ranked by the log *P*-value; orange indicates that the pathway is activated (*Z*-score > 0), and blue indicates that the pathway is inhibited (*Z*-score < 0). The color intensity (or absolute value of *Z*-score) represents the degree of activation or inhibition (according to the internal algorithm and standard of Ingenuity Pathway Analysis (IPA), *Z*-score > 2 indicates that the pathway is significantly activated, *Z*-score < −2 indicates that the pathway is significantly inhibited). The ratio denotes the ratio of the number of DEGs to all the genes involved in the signaling pathways.

**FIGURE 8:**
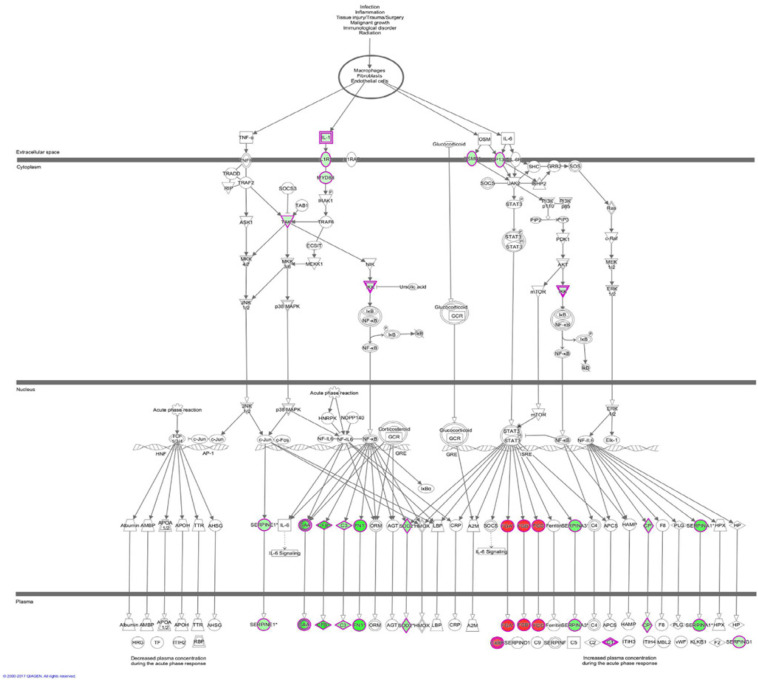
Expression patterns of genes in Acute Phase Response Signaling Pathway upon EI24 overexpressing. Red indicates upregulation; green indicates downregulation.

**FIGURE 9:**
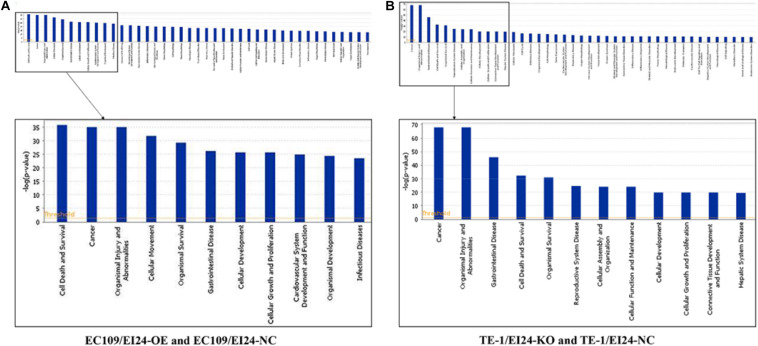
Histograms represented enrichment analysis of diseases and biofunctions regulated by differentially expressed genes (DEGs), ranked by the log *P*-value.

**FIGURE 10:**
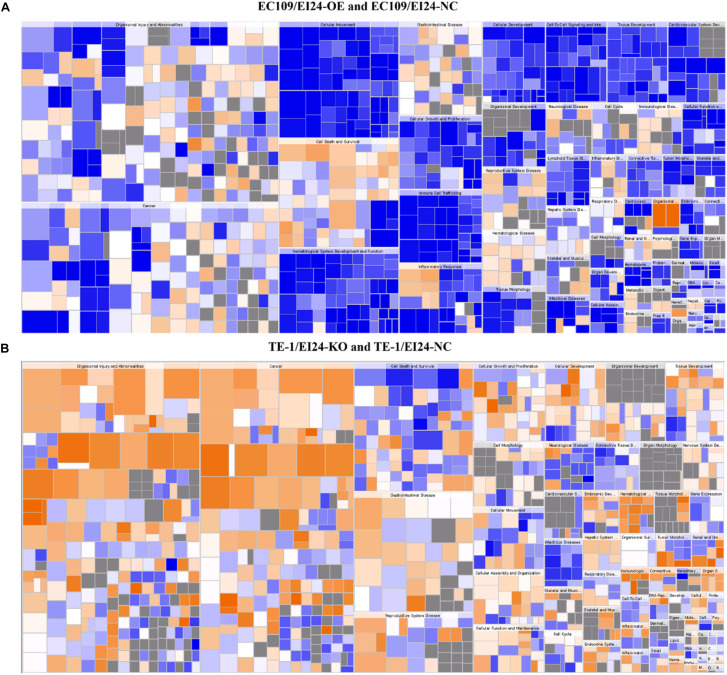
Disease and function heat maps. The figures demonstrate the relationships between changes in differentially expressed gene between EC109/EI24-OE and EC109/EI24-NC cells **(A)** or between TE-1/EI24-KO and TE-1/EI24-NC cells **(B)** and the activation and inhibition of diseases and functions. Positive *Z*-scores (orange) represent the activated state of the disease or biofunction, while negative *Z*-scores (blue) represent the suppressed state of the disease or biofunction. The intensity of the color in the heat map based on |*Z*-score| indicates robustness of the prediction.

The results of the canonical pathway analysis by IPA showed that upregulating EI24 expression significantly inhibited acute phase response signaling pathway in comparison with the control cells (*Z*-score = −2.828) (Figure 7A). The elaborate expression tendency of Acute Phase Response Signaling Pathway-related genes in EC109/EI24-OE cells is demonstrated in Figure 8. For an in-depth knowledge of the variation trend in the pathway, the expression of molecules involved in activated acute response signaling pathway reported in literatures was assessed and shown in Supplementary Figure S3A. Activation *Z*-score algorithm then was used to make predictions of activation or inhibition of upstream regulators of the DEGs between EC109/EI24-OE and EC109/EI24-NC cells by IPA. One hundred fifty-three molecules (including transcription factors, cytokines, chemical molecules and drugs, small RNAs, and kinases) were predicted as activators, and 391 molecules were identified as suppressors. Table 3 exhibits the IPA-predicted upstream activation or suppressive modulators acting on the EI24 gene (top 10). Afterward, we investigated the enrichment of DEGs in diseases and biofunctions upon EI24 overexpression. As shown in Figure 9A, the DEGs mainly involved in cell death, as well as survival, cancer, organismal injury and abnormality, cellular migration, organismal survival, gastrointestinal diseases, cellular growth, and proliferation. The heat map of diseases and biofunctions predicted by IPA based on the DEGs demonstrated that organismal death (*Z*-score = 10.648) and tumor cell death and apoptosis (*Z*-score = 10.237) were significantly activated, while several diseases and biofunctions were dramatically inhibited, including cell survival, tumor cell proliferation and movement, migration of macrophage, and angiogenesis (Figure 10A). In addition, we analyzed potential pathways involved in the upstream regulatory networks and downstream functions in which DEGs participate (Supplementary Table S1). Analysis of regulatory effects listed in the top position illustrated that regulator IL1A could inhibit migration of macrophage through regulating APP and other related genes (Supplementary Figure S4). Interestingly, infiltration of macrophages correlated prognosis of patients with solid tumors and was also associated with chemotherapy resistance in most cancers (23). The above analysis results were consistent with the experimental results of EI24 inhibiting proliferation and drug resistance of ESCC. To sum up, overexpression of EI24 may inhibit the proliferation and drug resistance of ESCC through suppressing Acute Phase Response Signaling Pathway and macrophage migration.

**TABLE 3:** Predicted upstream regulators for all differentially expressed genes (DEGs) between EC109/EI24-OE and EC109/EI24-NC cells (top 10).

Upstream regulator	Entrez gene name	Predicted state	*Z*-score	*P*-value
MiR-155-5p	MicroRNA-155-5p	Activated	6.369	3.09E-19
SB203580	SB203580	Activated	5.031	1.39E-17
PD98059	PD98059	Activated	4.964	1.18E-20
IL1RN	Interleukin 1 receptor antagonist	Activated	4.961	5.84E-12
U0126	U0126	Activated	4.763	2.98E-15
Lipopolysaccharide	Lipopolysaccharide	Inhibited	–9.476	3.39E-33
TNF	Tumor necrosis factor	Inhibited	–8.448	6.68E-44
IFNG	Interferon gamma	Inhibited	–7.821	1.38E-35
Poly rI:rC-RNA	Polyinosinic–polycytidylic acid	Inhibited	–7.245	1.13E-23
IL1B	Interleukin 1 beta	Inhibited	–7.065	1.26E-22

**FIGURE 11:**
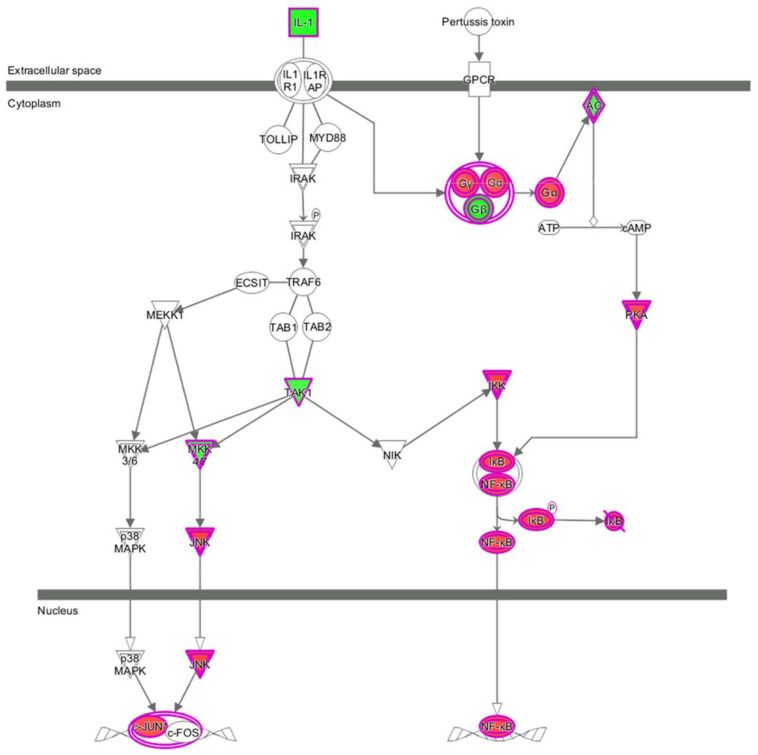
Expression patterns of genes in IL-1 Signaling Pathway upon EI24 silencing.

Apart from conducting IPA on DEGs between EC109/EI24-OE and EC109/EI24-NC cells, we also subjected the DEGs obtained from the microarray analysis of EI24-KO TE-1 cells to IPA software. IL-1 Signaling Pathway was identified as the top statistically significant canonical pathway (*Z*-score = 2.183) (Figure 7B), and the elaborate expression tendency of IL-1 signaling cascade is shown in Figure 11. Literature mining was performed to investigate the expression of molecules that participate in the activated IL-1 signaling cascade for understanding the expression tendency of pathway-related genes in detail (Supplementary Figure S3B). The “upstream regulators” module was also used for further analysis of DEGs between TE-1/EI24-KO and TE-1/EI24-NC cells. The IPA software analyzed and identified 69 potential molecules eligible for upstream regulators, including 42 activators and 27 inhibitors (Table 4). IPA system for analysis of the microarray data also revealed that EI24 silencing gave rise to dysregulation of several diseases and biofunctions, including cancer, organismal injury and abnormality, cell death, and survival (Figure 9B). IPA deduced 200 diseases or functions predicted to be activated upon EI24 knockdown, of which the top 5 were systemic autoimmune syndrome, growth of lesion, growth of tumor, renal cancer and tumors, and proliferation of lung cancer cell lines. Of the predicted 228 diseases or functions to be repressed, the top 5 were seizure disorder, autosomal dominant disease, apoptosis of neuroblastoma cell lines, apoptosis of tumor cell lines, and cell movement of lung cancer cell lines (Figure 10B). As for regulatory effect analysis, Supplementary Table S2 exhibited the potential pathways involved in the upstream regulatory networks and downstream functions in which DEGs participate.

**TABLE 4:** Predicted upstream regulators for all differentially expressed genes (DEGs) between TE-1/EI24-KO and TE-1/EI24-NC cells (top 10).

Upstream regulator	Entrez gene name	Predicted state	*Z*-score	*P*-value
Valproic acid	Valproic acid	Activated	3.556	2.72E-12
SRF	Serum response factor	Activated	3.174	0.00000783
UCP1	Uncoupling protein 1	Activated	3.057	0.000226
Kainic acid	Kainic acid	Activated	3.019	0.000624
Oltipraz	Oltipraz	Activated	2.928	0.00104
IgG	Immunoglobulin G	Inhibited	–2.642	0.00163
HSF2	Heat shock factor 2	Inhibited	–2.538	0.000621
TP63	Tumor protein 63	Inhibited	–2.525	2.34E-09
KDM5B	Lysine demethylase 5B	Inhibited	–2.447	0.000197
Ap1	Activator protein-1	Inhibited	–2.444	0.00104

## Discussion

ESCC has attracted great attention for its characteristics of complicated pathogenesis and high aggressiveness (4). Despite that great progress has been made in therapeutic approaches, the prognosis of ESCC patients still remains quite poor (24). Notably, the evolution of drug resistance is the critical cause of therapy failure in the management of ESCC patients (25). Therefore, we desperately need to identify novel and robust biomarkers to overcome the drug resistance and provide personalized therapy for ESCC patients.

Our previous report revealed that miR-483-3p could enhance the growth, migration, and drug resistance of ESCC via targeting EI24, indicating that EI24 is implicated in the tumorigenesis and/or development of ESCC (17). Aberrant expression of EI24 has been reported in various cancer types and is closely related to poor prognosis (10–13). The limited data indicate that EI24 has been implicated in cell growth, apoptosis, migration, and infiltration of cancer cells (9, 26, 27). In addition, it was reported that EI24 could sensitize tumor cells to chemotherapeutic agents and targeted drugs (15, 16). Thus, investigating the influence of EI24 in ESCC tumorigenesis and progression could provide a novel diagnostic biosignature and help to design more effective treatment approaches for ESCC patients.

Here, we established that EI24 was lowly expressed in ESCC tissues and that its expression was directly linked to the prognosis of ESCC patients. Meanwhile, high EI24 protein expression inversely linked to tumor size, T stage, lymph node metastasis, and AJCC stage. More importantly, multivariate Cox regression evaluation showed that EI24 was an independent prognostic factor for ESCC patients. To elucidate the putative roles of EI24 in ESCC, we conducted loss-of-function and gain-of-function assays. We showed that the forced expression of EI24 remarkably repressed the growth and colony formation potential of ESCC cells and triggers cycle arrest at G1/S phase, together with promoting cell apoptosis and enhancing the sensitivity of ESCC cells to chemotherapeutic drugs. On the contrary, CRISPR/Cas9-mediated gene knockdown of EI24 escalated the aggressive behavior of ESCC cells. The results of animal experiments also revealed that EI24 silencing caused the promotion of tumorigenesis.

Notably, cell cycle participates in the modulation of cell proliferation, and modification of cell cycle checkpoints has also been linked to drug resistance and apoptosis (28, 29). A previous study demonstrated that EI24 repressed cell growth and induced cell cycle arrest in pancreatic cancer cells through targeting c-Myc (30). Herein, our data indicated that EI24 was implicated in regulating the expression of CDK2, CDK4, and cyclin D1, which are crucial for cell cycle transition from G1 to S phase, and ectopic expression of EI24 could induce G1/S arrest and apoptosis. G1/S transition is responsible for activation and completion of DNA replication, whereas G1/S arrest is remarkably related to drug resistance (31). In addition, our study also indicated that EI24 was involved in regulating the expression of MDR1 and ABCG2, further reversing drug resistance via decreasing drug efflux and increasing intracellular drug accumulation. Of note, targeting CDK2 has been proven to be able to overcame multidrug resistance phenotype mediated by MDR1 and ABCG2 (32). Therefore, we hypothesize that EI24 may affect the expression of ABC transporters by regulating cyclin kinases and thus reverse drug resistance in ESCC, and the specific mechanism still needs further exploration. These findings underscored the pivotal function of EI24 in the proliferation, as well as drug resistance of ESCC.

Microarray experimentation and bioinformatic analysis then were performed to explore the underlying mechanism of EI24 in regulating ESCC cell proliferation and drug resistance. The identified DEGs obtained from gene microarray analysis were subjected to IPA software. The canonical pathway enrichment analysis of IPA indicated that the acute phase response signaling pathway was remarkably inhibited upon EI24 overexpressing, which was associated with inflammatory reactions induced in response to tissue injury or infection (33). Inflammatory response dysfunction had been identified to be strongly connected to the pathogenesis and progression of cancers (34). Notably, certain acute phase response signaling pathway-related genes, such as fibrinogen α chain (FGA) and serum amyloid a (SAA), were reported to be associated with malignant phenotypes of tumors, and their expressions dysregulated on account of EI24 overexpression (35, 36). Moreover, inflammation response has great potential to induce cell cycle arrest and inhibit cellular proliferation (37). Therefore, EI24 may exert its tumor suppressor effects by interacting with those molecules in the acute phase response signaling pathway. Furthermore, the canonical pathway analysis showed that EI24 silencing led to significant activation of IL-1 signaling pathway and, together with NF-κB as well as other pathway-related gene expressions, was dramatically upregulated. As reported in literatures, IL-1 participates in development, as well as in the progression of malignant tumors via modulating MAPKs and NF-κB signaling pathways (38, 39). Notably, EI24 could suppress NF-κB activity by interacting with the complex I component TNFR-associated factors 2/5 (TRAF2/5) and causing their lysosome-dependent degradation, resulting in reduction of EMT- and inflammation-linked gene transcription and repression of tumor progression (14). Together, knocking down EI24 expression may enhance the proliferation, as well as drug resistance of ESCC cells by activating IL-1 signaling cascade.

In conclusion, here, we provide the latest insights into the function of EI24 in regulating the proliferation, as well as drug resistance of ESCC. Biological or pharmacological intervention based on EI24 may be a prospective novel approach to revert the drug resistance and enhance the chemotherapeutic efficacy in human ESCC. We only conducted a preliminary investigation of the underlying mechanism of EI24 in ESCC. Therefore, further studies should be conducted on its function and mechanism to clarify its distinct function in ESCC. However, we still hope that the results of the present study will draw more attention to EI24 in ESCC research and shed a light on the direction of future studies.

## Data Availability Statement

The datasets presented in this study can be found in online repositories. The names of the repository/repositories and accession number(s) can be found below: the NCBI Gene Expression Omnibus (GSE154423 and GSE154422).

## Ethics Statement

The animal study was reviewed and approved by the Committee on the Use of Live Animals in Teaching and Research (CULATR) at the Fourth Military Medical University.

## Author Contributions

LD, JM, and LH conceived the study and wrote the manuscript. WY, LC, and XW collected and analyzed the data. LN, YL, WZ, YZ, JL, QZ, and DF participated in analyzing and discussing the results. All authors edited and approved the final manuscript.

## Conflict of Interest

The authors declare that the research was conducted in the absence of any commercial or financial relationships that could be construed as a potential conflict of interest.
