# Supplementary material for: EI24 Inhibits Cell Proliferation and Drug Resistance of Esophageal Squamous Cell Carcinoma
Source: Front Oncol. 2020 Aug 21;10:1570. doi: 10.3389/fonc.2020.01570 (PMC7471874; doi:10.3389/fonc.2020.01570)
Supplement: Supplementary file 1 [file Data_Sheet_1.PDF]

Gene Symbol	Gene Title	Fold Change	absolute FC	Regulation	P-value	FDR
FGA	fibrinogen alpha chain	4.656738921	4.656738921	up	3.74816E-13	9.81619E-11
PCDH9	protocadherin 9	2.208800827	2.208800827	up	4.16881E-07	5.71216E-06
AKR1B10	aldo-keto reductase family 1, member B10 (aldose reductase)	2.202257755	2.202257755	up	6.16099E-15	3.3615E-12
AKR1B15	aldo-keto reductase family 1, member B15	2.202257755	2.202257755	up	6.16099E-15	3.3615E-12
CCL26	chemokine (C-C motif) ligand 26	2.166465606	2.166465606	up	3.4655E-12	5.27669E-10
FGB	fibrinogen beta chain	2.154644716	2.154644716	up	2.02245E-10	1.21856E-08
KRT5	keratin 5, type II	2.134285733	2.134285733	up	1.71848E-10	1.06987E-08
CCDC167	coiled-coil domain containing 167	2.134014531	2.134014531	up	1.7904E-13	5.369E-11
COX20P1	COX20 cytochrome c oxidase assembly factor pseudogene 1	2.12700978	2.12700978	up	1.05844E-11	1.20173E-09
CD55	CD55 molecule, decay accelerating factor for complement (Cromer blood group)	2.073531552	2.073531552	up	2.10437E-07	3.2585E-06
ABCD3	ATP binding cassette subfamily D member 3	2.059560729	2.059560729	up	3.06549E-07	4.43638E-06
THBS1	thrombospondin 1	2.056446206	2.056446206	up	3.56295E-09	1.18717E-07
LOC105369237	sperm protein associated with the nucleus on the X chromosome B/F	2.041689295	2.041689295	up	1.16961E-07	2.00729E-06
SPANXB1	SPANX family member B1	2.041689295	2.041689295	up	1.16961E-07	2.00729E-06
SAMD5	sterile alpha motif domain containing 5	2.027141318	2.027141318	up	3.36842E-09	1.13292E-07
FGG	fibrinogen gamma chain	2.008465017	2.008465017	up	4.82318E-07	6.4403E-06
KIAA1324	KIAA1324	1.95156746	1.95156746	up	3.05075E-09	1.04123E-07
TP53I3	tumor protein p53 inducible protein 3	1.922584045	1.922584045	up	1.86054E-09	6.98084E-08
SCLT1	sodium channel and clathrin linker 1	1.904479379	1.904479379	up	3.95417E-05	0.000254221
TM4SF1	transmembrane 4 L six family member 1	1.903375228	1.903375228	up	7.45305E-08	1.38172E-06
C2orf76	chromosome 2 open reading frame 76	1.870853599	1.870853599	up	5.57551E-08	1.08915E-06
CREM	cAMP responsive element modulator	1.867959704	1.867959704	up	1.07823E-07	1.87338E-06
MKKS	McKusick-Kaufman syndrome	1.842770831	1.842770831	up	5.72795E-13	1.34741E-10
CALB2	calbindin 2	1.832580619	1.832580619	up	9.61089E-12	1.11373E-09
LOC105377283	uncharacterized LOC105377283	1.813994397	1.813994397	up	8.43834E-09	2.38827E-07
DSC2	desmocollin 2	1.808708606	1.808708606	up	7.60021E-07	9.38888E-06
GPAM	glycerol-3-phosphate acyltransferase, mitochondrial	1.804918048	1.804918048	up	9.54592E-06	7.67346E-05
ALDH3A1	aldehyde dehydrogenase 3 family member A1	1.795791679	1.795791679	up	3.56993E-16	3.50603E-13
C4BPB	complement component 4 binding protein, beta	1.782489197	1.782489197	up	2.35729E-06	2.39968E-05
CYP4F11	cytochrome P450, family 4, subfamily F, polypeptide 11	1.778905657	1.778905657	up	4.77295E-13	1.16296E-10
ZNF226	zinc finger protein 226	1.762675208	1.762675208	up	2.65211E-10	1.51432E-08
EPHX1	epoxide hydrolase 1, microsomal (xenobiotic)	1.755874825	1.755874825	up	3.35899E-11	2.95201E-09
CPNE3	copine III	1.755724725	1.755724725	up	7.60693E-10	3.44672E-08
C17orf89	chromosome 17 open reading frame 89	1.755087957	1.755087957	up	2.96805E-09	1.02278E-07
SCARA5	scavenger receptor class A, member 5	1.750972793	1.750972793	up	1.9768E-08	4.66966E-07
MIER1	mesoderm induction early response 1, transcriptional regulator	1.750851429	1.750851429	up	2.02775E-05	0.000144204
PSTK	phosphoseryl-tRNA kinase	1.742831789	1.742831789	up	8.47983E-07	1.02829E-05
UFM1	ubiquitin-fold modifier 1	1.73136912	1.73136912	up	2.44423E-12	4.05143E-10
POPDC3	popeye domain containing 3	1.730993131	1.730993131	up	1.09663E-11	1.23536E-09
TMEM256	transmembrane protein 256	1.726344034	1.726344034	up	9.27681E-13	1.9282E-10
PLEKHS1	pleckstrin homology domain containing S1	1.719822888	1.719822888	up	1.39311E-07	2.31796E-06
PSCA	prostate stem cell antigen	1.715841947	1.715841947	up	9.03181E-12	1.06903E-09
MAP2	microtubule associated protein 2	1.713219496	1.713219496	up	1.14256E-10	7.69889E-09
GCLM	glutamate-cysteine ligase, modifier subunit	1.713144289	1.713144289	up	2.89896E-10	1.60172E-08
LOC105371964	uncharacterized LOC105371964	1.712012616	1.712012616	up	3.28583E-09	1.10826E-07
LFNG	LFNG O-fucosylpeptide 3-beta-N-acetylglucosaminyltransferase	1.708787896	1.708787896	up	8.41769E-10	3.71134E-08
CTH	cystathionine gamma-lyase	1.704576483	1.704576483	up	1.66951E-07	2.68681E-06
PIF1	PIF1 5'-to-3' DNA helicase	1.701747118	1.701747118	up	1.4133E-09	5.58551E-08
FAM98B	family with sequence similarity 98 member B	1.688005962	1.688005962	up	6.81213E-11	5.13642E-09
FRMD4A	FERM domain containing 4A	1.687479528	1.687479528	up	3.44217E-07	4.86586E-06
SLC25A15	solute carrier family 25 (mitochondrial carrier; ornithine transporter) member 15	1.685920686	1.685920686	up	1.12839E-07	1.9442E-06
ASPH	aspartate beta-hydroxylase	1.683919689	1.683919689	up	0.000114256	0.000625739
CTSC	cathepsin C	1.676384786	1.676384786	up	7.75565E-10	3.49395E-08
PCOLCE2	procollagen C-endopeptidase enhancer 2	1.675138056	1.675138056	up	1.40952E-05	0.00010673
POLR2A	polymerase (RNA) II (DNA directed) polypeptide A, 220kDa	1.67505678	1.67505678	up	1.23648E-10	8.21893E-09
IQCG	IQ motif containing G	1.67219914	1.67219914	up	1.00431E-07	1.76842E-06
LZIC	leucine zipper and CTNNBIP1 domain containing	1.670723896	1.670723896	up	3.47307E-08	7.41904E-07
ANAPC15	anaphase promoting complex subunit 15	1.670276174	1.670276174	up	8.52858E-11	6.15876E-09
GNL3L	guanine nucleotide binding protein-like 3 (nucleolar)-like	1.667511449	1.667511449	up	2.14316E-09	7.81001E-08
SIPA1L2	signal-induced proliferation-associated 1 like 2	1.666798839	1.666798839	up	2.47148E-05	0.000170362
RNASEH2C	ribonuclease H2 subunit C	1.665743965	1.665743965	up	9.35783E-12	1.09735E-09
GPI	glucose-6-phosphate isomerase	1.663121228	1.663121228	up	1.57088E-13	5.01462E-11
C8orf82	chromosome 8 open reading frame 82	1.660199559	1.660199559	up	1.6758E-08	4.10168E-07
GAL	galanin/GMAP prepropeptide	1.659371217	1.659371217	up	7.26715E-08	1.35776E-06
CREB3L2	cAMP responsive element binding protein 3-like 2	1.658267403	1.658267403	up	3.08022E-05	0.000204917
NMB	neuromedin B	1.650763373	1.650763373	up	1.43891E-12	2.67896E-10
UST	uronyl-2-sulfotransferase	1.650580308	1.650580308	up	3.10637E-06	3.01088E-05
RABGAP1L	RAB GTPase activating protein 1-like	1.649852061	1.649852061	up	2.35369E-11	2.21673E-09
NEK1	NIMA-related kinase 1	1.648019523	1.648019523	up	8.02166E-05	0.00046419
FAM104B	family with sequence similarity 104 member B	1.644656922	1.644656922	up	1.07064E-06	1.24619E-05
FAM13A	family with sequence similarity 13 member A	1.644246577	1.644246577	up	7.32308E-06	6.18667E-05
MROH1	maestro heat-like repeat family member 1	1.643893306	1.643893306	up	2.41523E-09	8.60197E-08
YWHAE	tyrosine 3-monooxygenase/tryptophan 5-monooxygenase activation protein, epsilon	1.634972836	1.634972836	up	3.77495E-07	5.25124E-06
ARMC5	armadillo repeat containing 5	1.634421401	1.634421401	up	9.41582E-08	1.67751E-06
RNGTT	RNA guanylyltransferase and 5'-phosphatase	1.633462498	1.633462498	up	1.51273E-06	1.65717E-05
NAP1L2	nucleosome assembly protein 1-like 2	1.629711582	1.629711582	up	1.59482E-09	6.14733E-08
MRPS11	mitochondrial ribosomal protein S11	1.629026418	1.629026418	up	1.41493E-05	0.000107057
TUSC2	tumor suppressor candidate 2	1.628657602	1.628657602	up	1.95757E-09	7.26856E-08
FOSB	FBJ murine osteosarcoma viral oncogene homolog B	1.624970281	1.624970281	up	5.73676E-12	7.74443E-10
CPS1	carbamoyl-phosphate synthase 1	1.620194109	1.620194109	up	1.78971E-05	0.000129886
LETM1	leucine zipper-EF-hand containing transmembrane protein 1	1.618903135	1.618903135	up	1.03563E-07	1.81461E-06
SQSTM1	sequestosome 1	1.618570269	1.618570269	up	8.48414E-12	1.02868E-09
FLVCR1-AS1	FLVCR1 antisense RNA 1 (head to head)	1.616548359	1.616548359	up	9.7557E-09	2.68002E-07
LANCL2	LanC lantibiotic synthetase component C-like 2 (bacterial)	1.616148762	1.616148762	up	6.29521E-09	1.88492E-07
PSRC1	proline/serine-rich coiled-coil 1	1.615316273	1.615316273	up	9.95617E-08	1.75752E-06
TYW3	tRNA-yW synthesizing protein 3 homolog	1.611182591	1.611182591	up	1.55743E-10	9.86808E-09
WFDC3	WAP four-disulfide core domain 3	1.609177346	1.609177346	up	1.12613E-11	1.26397E-09
PDSS2	prenyl (decaprenyl) diphosphate synthase, subunit 2	1.609062093	1.609062093	up	4.61716E-09	1.45737E-07
OPA3	optic atrophy 3 (autosomal recessive, with chorea and spastic paraplegia)	1.60868293	1.60868293	up	5.4853E-06	4.83583E-05
FBN2	fibrillin 2	1.605348662	1.605348662	up	4.79601E-05	0.000299229
ALKBH7	alkB homolog 7	1.60527448	1.60527448	up	6.2805E-09	1.88338E-07
CRCP	CGRP receptor component	1.605185468	1.605185468	up	7.59813E-07	9.38888E-06
ADIRF	adipogenesis regulatory factor	1.604744185	1.604744185	up	2.3102E-13	6.62438E-11
CDKN2A	cyclin-dependent kinase inhibitor 2A	1.604581053	1.604581053	up	1.59345E-05	0.000118125
GPATCH2L	G-patch domain containing 2 like	1.603617426	1.603617426	up	1.92061E-08	4.57546E-07
MBD3	methyl-CpG binding domain protein 3	1.60353221	1.60353221	up	1.55402E-11	1.63668E-09

CRACR2A	calcium release activated channel regulator 2A	1.60091865	1.60091865	up	2.71975E-05	0.000184804
PPIG	peptidylprolyl isomerase G (cyclophilin G)	1.600726319	1.600726319	up	0.000215764	0.001074051
ZNF780A	zinc finger protein 780A	1.600071824	1.600071824	up	3.79972E-05	0.000246154
C14orf2	chromosome 14 open reading frame 2	1.595387265	1.595387265	up	4.21384E-09	1.35686E-07
SWI5	SWI5 homologous recombination repair protein	1.594053445	1.594053445	up	1.13421E-09	4.70002E-08
ZFAT	zinc finger and AT-hook domain containing	1.591892962	1.591892962	up	2.11652E-05	0.00014938
N6AMT2	N-6 adenine-specific DNA methyltransferase 2 (putative)	1.591812047	1.591812047	up	2.71998E-06	2.70237E-05
IMPDH2	IMP (inosine 5'-monophosphate) dehydrogenase 2	1.589797852	1.589797852	up	4.73757E-13	1.16296E-10
PTRHD1	peptidyl-tRNA hydrolase domain containing 1	1.587001326	1.587001326	up	4.42821E-11	3.66999E-09
RXRA	retinoid X receptor alpha	1.584143835	1.584143835	up	2.26532E-07	3.46673E-06
ZC3HAV1	zinc finger CCCH-type, antiviral 1	1.582563439	1.582563439	up	5.0486E-11	4.08085E-09
NUDT16	nudix hydrolase 16	1.578312994	1.578312994	up	4.06355E-07	5.58506E-06
TAF9B	TATA-box binding protein associated factor 9b	1.57475057	1.57475057	up	7.18418E-09	2.09497E-07
SF3B3	splicing factor 3b subunit 3	1.573739407	1.573739407	up	5.7944E-08	1.12076E-06
COX11	COX11 cytochrome c oxidase copper chaperone	1.571152624	1.571152624	up	1.99203E-08	4.69717E-07
COX20	COX20 cytochrome c oxidase assembly factor	1.570564653	1.570564653	up	6.34811E-06	5.48086E-05
CTRL	chymotrypsin-like	1.569813676	1.569813676	up	1.81643E-05	0.00013161
CEBPA	CCAAT/enhancer binding protein (C/EBP), alpha	1.564523505	1.564523505	up	1.39873E-07	2.32433E-06
AP5S1	adaptor-related protein complex 5, sigma 1 subunit	1.564097015	1.564097015	up	1.12155E-08	3.00538E-07
ANKRD13C	ankyrin repeat domain 13C	1.563544196	1.563544196	up	1.88858E-05	0.000135906
BCAS3	breast carcinoma amplified sequence 3	1.563475559	1.563475559	up	4.01581E-07	5.5334E-06
TMEM99	transmembrane protein 99	1.563002407	1.563002407	up	3.92185E-10	2.03522E-08
PCBD2	pterin-4 alpha-carbinolamine dehydratase/dimerization cofactor of hepatocyte nuclear factor 1 alpha (TCF1) 2	1.561338483	1.561338483	up	2.75634E-08	6.16629E-07
ATP7A	ATPase, Cu++ transporting, alpha polypeptide	1.561093195	1.561093195	up	2.32358E-07	3.53E-06
SMIM11B	small integral membrane protein 11B	1.558653238	1.558653238	up	6.25129E-08	1.19443E-06
SMIM11A	small integral membrane protein 11A	1.558653238	1.558653238	up	6.25129E-08	1.19443E-06
GTPBP6	GTP binding protein 6 (putative)	1.555965422	1.555965422	up	3.56402E-07	5.01106E-06
LRTOMT	leucine rich transmembrane and O-methyltransferase domain containing	1.555922282	1.555922282	up	2.107E-07	3.26129E-06
NRG1	neuregulin 1	1.552245411	1.552245411	up	3.60385E-09	1.19673E-07
NDST1	N-deacetylase/N-sulfotransferase (heparan glucosaminyl) 1	1.549962524	1.549962524	up	2.2251E-06	2.28764E-05
IDS	iduronate 2-sulfatase	1.548008436	1.548008436	up	4.75484E-10	2.36442E-08
LMNA	lamin A/C	1.547840342	1.547840342	up	6.3463E-13	1.45798E-10
AVEN	apoptosis, caspase activation inhibitor	1.547582872	1.547582872	up	1.35306E-08	3.46918E-07
SERPINB8	serpin peptidase inhibitor, clade B (ovalbumin), member 8	1.547554267	1.547554267	up	0.000145683	0.000769534
DPY19L1P2	DPY19L1 pseudogene 2	1.547161	1.547161	up	4.31817E-07	5.88602E-06
DPY19L1	dpy-19-like 1 (C. elegans)	1.547161	1.547161	up	4.31817E-07	5.88602E-06
HMGN5	high mobility group nucleosome binding domain 5	1.547078784	1.547078784	up	3.09715E-06	3.0049E-05
CLPB	ClpB homolog, mitochondrial AAA ATPase chaperonin	1.546556993	1.546556993	up	9.19852E-07	1.09402E-05
PDE4D	phosphodiesterase 4D	1.545356828	1.545356828	up	2.89068E-06	2.83823E-05
TXNL4B	thioredoxin like 4B	1.544364539	1.544364539	up	3.53116E-07	4.97019E-06
RAB38	RAB38, member RAS oncogene family	1.541843853	1.541843853	up	3.71308E-11	3.21033E-09
SVBP	small vasohibin binding protein	1.5412348	1.5412348	up	4.71455E-08	9.55658E-07
CYP4F3	cytochrome P450, family 4, subfamily F, polypeptide 3	1.538982329	1.538982329	up	2.89694E-08	6.40424E-07
CYP4F2	cytochrome P450, family 4, subfamily F, polypeptide 2	1.538982329	1.538982329	up	2.89694E-08	6.40424E-07
ZSCAN5A	zinc finger and SCAN domain containing 5A	1.538868548	1.538868548	up	3.04452E-06	2.96629E-05
TPM3	tropomyosin 3	1.536324885	1.536324885	up	6.39488E-08	1.21596E-06
CABYR	calcium binding tyrosine-(Y)-phosphorylation regulated	1.536214849	1.536214849	up	1.40803E-10	9.06768E-09
S100A2	S100 calcium binding protein A2	1.535987704	1.535987704	up	3.74312E-12	5.56988E-10
ANKHD1	ankyrin repeat and KH domain containing 1	1.535207147	1.535207147	up	7.4307E-07	9.24196E-06
LANCL1	LanC lantibiotic synthetase component C-like 1 (bacterial)	1.534987244	1.534987244	up	7.08185E-07	8.87171E-06
ING3	inhibitor of growth family member 3	1.53472482	1.53472482	up	2.49924E-08	5.67516E-07
ATF5	activating transcription factor 5	1.532092427	1.532092427	up	5.00509E-07	6.64706E-06
DLEU1	deleted in lymphocytic leukemia 1 (non-protein coding)	1.53043312	1.53043312	up	4.24087E-09	1.36333E-07
PDGFA	platelet-derived growth factor alpha polypeptide	1.528803868	1.528803868	up	8.33012E-10	3.68099E-08
KIF9	kinesin family member 9	1.528634327	1.528634327	up	5.06841E-08	1.00712E-06
TMEM19	transmembrane protein 19	1.527772788	1.527772788	up	2.03149E-09	7.46981E-08
AGR2	anterior gradient 2, protein disulphide isomerase family member	1.52613579	1.52613579	up	5.62376E-12	7.67096E-10
NMU	neuromedin U	1.524750653	1.524750653	up	0.000196414	0.000991681
FECH	ferrochelatase	1.524113137	1.524113137	up	8.69018E-05	0.000495695
ZBTB44	zinc finger and BTB domain containing 44	1.523373812	1.523373812	up	1.13818E-05	8.89442E-05
PRKAR2A	protein kinase, cAMP-dependent, regulatory subunit type II alpha	1.523363253	1.523363253	up	7.40547E-10	3.36709E-08
RPS2P32	ribosomal protein S2 pseudogene 32	1.523349174	1.523349174	up	3.46752E-08	7.41524E-07
MAOB	monoamine oxidase B	1.521956017	1.521956017	up	8.5828E-06	7.04653E-05
SERPINI1	serpin peptidase inhibitor, clade I (neuroserpin), member 1	1.521864591	1.521864591	up	8.2429E-05	0.00047494
PLK1	polo-like kinase 1	1.52134779	1.52134779	up	1.78476E-08	4.32259E-07
GPX2	glutathione peroxidase 2	1.52110527	1.52110527	up	3.85457E-13	9.89693E-11
SMTN	smoothelin	1.52107364	1.52107364	up	7.22356E-08	1.35064E-06
CLN5	ceroid-lipofuscinosis, neuronal 5	1.520384968	1.520384968	up	4.98854E-06	4.46502E-05
NHLRC1	NHL repeat containing E3 ubiquitin protein ligase 1	1.519261276	1.519261276	up	1.58856E-06	1.72819E-05
WARS2	tryptophanyl tRNA synthetase 2, mitochondrial	1.518955916	1.518955916	up	1.91448E-05	0.000137367
RABL3	RAB, member of RAS oncogene family-like 3	1.517913942	1.517913942	up	3.01232E-06	2.93929E-05
BBS4	Bardet-Biedl syndrome 4	1.517391471	1.517391471	up	1.11461E-08	2.99497E-07
INHBB	inhibin beta B	1.516823618	1.516823618	up	7.54122E-13	1.64583E-10
MYEOV2	myeloma overexpressed 2	1.516662415	1.516662415	up	9.5951E-09	2.64701E-07
BCL2L11	BCL2-like 11 (apoptosis facilitator)	1.515443431	1.515443431	up	0.000381474	0.001744706
HIST1H1A	histone cluster 1, H1a	1.514554332	1.514554332	up	1.18886E-05	9.22627E-05
ANP32E	acidic nuclear phosphoprotein 32 family member E	1.513914084	1.513914084	up	9.36958E-10	4.04034E-08
NQO2	NAD(P)H dehydrogenase, quinone 2	1.513053849	1.513053849	up	4.10559E-10	2.10328E-08
EPHA4	EPH receptor A4	1.511544375	1.511544375	up	8.60094E-05	0.000492033
ATP5I	ATP synthase, H+ transporting, mitochondrial Fo complex subunit E	1.51111487	1.51111487	up	1.7988E-12	3.15465E-10
NAALADL2	N-acetylated alpha-linked acidic dipeptidase-like 2	1.508718169	1.508718169	up	4.49652E-06	4.08703E-05
MCCC2	methylcrotonoyl-CoA carboxylase 2	1.508167501	1.508167501	up	3.17276E-07	4.55385E-06
CRADD	CASP2 and RIPK1 domain containing adaptor with death domain	1.507996765	1.507996765	up	3.61991E-06	3.41103E-05
OBFC1	oligonucleotide/oligosaccharide-binding fold containing 1	1.507554335	1.507554335	up	1.22805E-07	2.09751E-06
FGF13	fibroblast growth factor 13	1.507031947	1.507031947	up	2.67687E-05	0.000182376
SLC25A21	solute carrier family 25 (mitochondrial oxoadipate carrier), member 21	1.507024983	1.507024983	up	1.98451E-05	0.000141538
NECAB2	N-terminal EF-hand calcium binding protein 2	1.506955345	1.506955345	up	3.17295E-07	4.55385E-06
DUS3L	dihydrouridine synthase 3-like	1.506749932	1.506749932	up	1.78928E-06	1.90798E-05
ADCK1	aarF domain containing kinase 1	1.506488855	1.506488855	up	9.46136E-06	7.62264E-05
PAPSS2	3'-phosphoadenosine 5'-phosphosulfate synthase 2	1.505761558	1.505761558	up	5.08075E-09	1.57159E-07
FMR1	fragile X mental retardation 1	1.505545873	1.505545873	up	5.50856E-07	7.18932E-06
FAT2	FAT atypical cadherin 2	1.503560931	1.503560931	up	3.86606E-06	3.60148E-05
C1orf53	chromosome 1 open reading frame 53	1.502873243	1.502873243	up	5.29695E-08	1.04408E-06
STK11IP	serine/threonine kinase 11 interacting protein	1.502539932	1.502539932	up	8.30982E-05	0.000477954
TSPAN5	tetraspanin 5	1.502258759	1.502258759	up	2.12768E-06	2.20713E-05
PTHLH	parathyroid hormone-like hormone	1.501405146	1.501405146	up	1.13758E-05	8.8915E-05
ATG9A	autophagy related 9A	1.5011554	1.5011554	up	2.98123E-09	1.02405E-07

ABCB6	ATP binding cassette subfamily B member 6 (Langereis blood group)	1.5011554	1.5011554	up	2.98123E-09	1.02405E-07
BHLHE41	basic helix-loop-helix family member e41	1.501082566	1.501082566	up	4.54063E-09	1.43966E-07
VGLL3	vestigial like family member 3	1.500992394	1.500992394	up	0.000139991	0.000743668
ACTR2	ARP2 actin-related protein 2 homolog (yeast)	1.50008058	1.50008058	up	5.71768E-09	1.72647E-07
TRIO	trio Rho guanine nucleotide exchange factor	-1.500153366	1.500153366	down	2.1153E-11	2.0567E-09
ADPGK	ADP-dependent glucokinase	-1.500829406	1.500829406	down	7.47671E-07	9.28303E-06
ZNF277	zinc finger protein 277	-1.501918644	1.501918644	down	7.99111E-05	0.000462741
COL4A2	collagen, type IV, alpha 2	-1.502116456	1.502116456	down	7.63926E-08	1.40892E-06
RAI14	retinoic acid induced 14	-1.502206695	1.502206695	down	1.92631E-07	3.0233E-06
POLK	polymerase (DNA directed) kappa	-1.502237933	1.502237933	down	4.81615E-06	4.33426E-05
VPS45	vacuolar protein sorting 45 homolog (S. cerevisiae)	-1.503092019	1.503092019	down	1.10972E-05	8.70841E-05
LMAN1	lectin, mannose-binding, 1	-1.503182316	1.503182316	down	5.18249E-08	1.02564E-06
SQLE	squalene epoxidase	-1.503522718	1.503522718	down	4.68101E-09	1.47465E-07
PTAR1	protein prenyltransferase alpha subunit repeat containing 1	-1.503713793	1.503713793	down	1.02173E-05	8.12011E-05
PLRG1	pleiotropic regulator 1	-1.503901418	1.503901418	down	9.46702E-07	1.1212E-05
GPR153	G protein-coupled receptor 153	-1.503908367	1.503908367	down	6.18419E-07	7.96002E-06
PTCD3	pentatricopeptide repeat domain 3	-1.504012614	1.504012614	down	1.8262E-08	4.39317E-07
TTC17	tetratricopeptide repeat domain 17	-1.504523527	1.504523527	down	2.6942E-09	9.4499E-08
NUPR1	nuclear protein 1, transcriptional regulator	-1.504527003	1.504527003	down	3.31314E-08	7.11998E-07
OXA1L	oxidase (cytochrome c) assembly 1-like	-1.504554813	1.504554813	down	2.76639E-08	6.18174E-07
ATPIF1	ATPase inhibitory factor 1	-1.504735589	1.504735589	down	1.17491E-05	9.12881E-05
DDR1	discoidin domain receptor tyrosine kinase 1	-1.505545873	1.505545873	down	2.24981E-09	8.11583E-08
TMEM183A	transmembrane protein 183A	-1.505556309	1.505556309	down	6.04169E-05	0.000364748
TMEM183B	transmembrane protein 183B	-1.505556309	1.505556309	down	6.04169E-05	0.000364748
ISG20	interferon stimulated exonuclease gene 20kDa	-1.505973795	1.505973795	down	1.2234E-07	2.0923E-06
BAZ1A	bromodomain adjacent to zinc finger domain 1A	-1.506527144	1.506527144	down	5.28922E-08	1.04308E-06
ADA	adenosine deaminase	-1.506551509	1.506551509	down	4.2135E-06	3.87552E-05
RUNX2	runt-related transcription factor 2	-1.507390634	1.507390634	down	1.54543E-08	3.8207E-07
PCSK6	proprotein convertase subtilisin/kexin type 6	-1.507571751	1.507571751	down	4.70027E-05	0.000294021
PLPP5	phospholipid phosphatase 5	-1.507665801	1.507665801	down	2.17205E-05	0.000152779
SMARCE1	SWI/SNF related, matrix associated, actin dependent regulator of chromatin, subfamily e, member 1	-1.508693768	1.508693768	down	2.22287E-05	0.000155883
SEC63	SEC63 homolog, protein translocation regulator	-1.508930823	1.508930823	down	7.12135E-05	0.000420305
C1GALT1	core 1 synthase, glycoprotein-N-acetylgalactosamine 3-beta-galactosyltransferase 1	-1.508979633	1.508979633	down	2.37692E-09	8.49634E-08
RPS6KA3	ribosomal protein S6 kinase, 90kDa, polypeptide 3	-1.509865459	1.509865459	down	3.07123E-06	2.98492E-05
SPTLC2	serine palmitoyltransferase, long chain base subunit 2	-1.510130611	1.510130611	down	1.50742E-09	5.86311E-08
DUSP16	dual specificity phosphatase 16	-1.510231799	1.510231799	down	8.21682E-08	1.49301E-06
MCL1	myeloid cell leukemia 1	-1.510350443	1.510350443	down	2.15187E-05	0.000151494
BTG3	BTG family member 3	-1.510420237	1.510420237	down	7.29731E-11	5.42931E-09
OSBPL2	oxysterol binding protein like 2	-1.510580777	1.510580777	down	2.79229E-05	0.000188572
PARP6	poly(ADP-ribose) polymerase family member 6	-1.510601719	1.510601719	down	7.0475E-08	1.31961E-06
AK4	adenylate kinase 4	-1.510919363	1.510919363	down	2.91582E-08	6.43745E-07
AK4P1	adenylate kinase 4 pseudogene 1	-1.510919363	1.510919363	down	2.91582E-08	6.43745E-07
RHOA	ras homolog family member A	-1.510919363	1.510919363	down	3.73266E-09	1.22706E-07
ODF2L	outer dense fiber of sperm tails 2-like	-1.511956535	1.511956535	down	3.91435E-06	3.63783E-05
STARD13	StAR related lipid transfer domain containing 13	-1.511984483	1.511984483	down	8.22808E-08	1.49368E-06
SNORA6	small nucleolar RNA, H/ACA box 6	-1.512959463	1.512959463	down	1.4099E-05	0.000106738
RPSA	ribosomal protein SA	-1.512959463	1.512959463	down	1.4099E-05	0.000106738
NLRC5	NLR family, CARD domain containing 5	-1.512997916	1.512997916	down	2.41051E-08	5.50548E-07
TPTE2P1	transmembrane phosphoinositide 3-phosphatase and tensin homolog 2 pseudogene 1	-1.513123768	1.513123768	down	2.91866E-05	0.000195894
DNMT3B	DNA (cytosine-5-)-methyltransferase 3 beta	-1.513207676	1.513207676	down	3.52307E-05	0.000230744
RAB11FIP1	RAB11 family interacting protein 1 (class I)	-1.513511881	1.513511881	down	1.69106E-05	0.000123986
SELM	selenoprotein M	-1.513518875	1.513518875	down	2.18723E-07	3.36426E-06
HIVEP1	human immunodeficiency virus type I enhancer binding protein 1	-1.513963055	1.513963055	down	0.000107103	0.000592427
FAM111B	family with sequence similarity 111 member B	-1.514347884	1.514347884	down	2.89269E-05	0.000194317
LRRD1	leucine-rich repeats and death domain containing 1	-1.514449355	1.514449355	down	1.42791E-07	2.35987E-06
CYP51A1	cytochrome P450, family 51, subfamily A, polypeptide 1	-1.514449355	1.514449355	down	1.42791E-07	2.35987E-06
RAP1B	RAP1B, member of RAS oncogene family	-1.514764308	1.514764308	down	0.000162657	0.000846892
RAP1BL	RAP1B, member of RAS oncogene family pseudogene	-1.514764308	1.514764308	down	0.000162657	0.000846892
MOV10	Mov10 RISC complex RNA helicase	-1.515478446	1.515478446	down	1.59197E-10	1.00446E-08
SS18	synovial sarcoma translocation, chromosome 18	-1.515527468	1.515527468	down	4.98725E-07	6.6256E-06
ITGB2	integrin, beta 2 (complement component 3 receptor 3 and 4 subunit)	-1.51668344	1.51668344	down	3.20882E-06	3.08884E-05
ACADVL	acyl-CoA dehydrogenase, very long chain	-1.516711475	1.516711475	down	2.16462E-08	5.03918E-07
EGFR	epidermal growth factor receptor	-1.517065455	1.517065455	down	1.16791E-09	4.81933E-08
ZNF544	zinc finger protein 544	-1.517097002	1.517097002	down	4.02965E-07	5.54858E-06
MCM4	minichromosome maintenance complex component 4	-1.517258252	1.517258252	down	0.000447266	0.001993126
C15orf52	chromosome 15 open reading frame 52	-1.517486133	1.517486133	down	4.65417E-10	2.32318E-08
CTSS	cathepsin S	-1.517493146	1.517493146	down	6.5381E-07	8.32014E-06
SASH1	SAM and SH3 domain containing 1	-1.517664957	1.517664957	down	1.34155E-06	1.49508E-05
KCTD3	potassium channel tetramerization domain containing 3	-1.517808732	1.517808732	down	2.05676E-06	2.14318E-05
POLR2C	polymerase (RNA) II (DNA directed) polypeptide C, 33kDa	-1.518282235	1.518282235	down	3.17137E-07	4.55385E-06
SUCLA2	succinate-CoA ligase, ADP-forming, beta subunit	-1.518840106	1.518840106	down	3.60801E-07	5.0611E-06
MATR3	matrin 3	-1.518840106	1.518840106	down	7.04503E-10	3.22937E-08
HERC1	HECT and RLD domain containing E3 ubiquitin protein ligase family member 1	-1.518889237	1.518889237	down	1.76956E-05	0.000128778
PPP4R2	protein phosphatase 4 regulatory subunit 2	-1.519085775	1.519085775	down	1.46987E-05	0.000110406
ENPP5	ectonucleotide pyrophosphatase/phosphodiesterase 5 (putative)	-1.519155973	1.519155973	down	0.000137227	0.000731356
MORC3	MORC family CW-type zinc finger 3	-1.519675541	1.519675541	down	1.79607E-05	0.000130311
CFD	complement factor D (adipsin)	-1.519991582	1.519991582	down	7.46743E-07	9.27444E-06
BCAR3	breast cancer anti-estrogen resistance 3	-1.520290125	1.520290125	down	7.19023E-09	2.09497E-07
EIF4A1	eukaryotic translation initiation factor 4A1	-1.520725751	1.520725751	down	7.02768E-07	8.81466E-06
SNORD10	small nucleolar RNA, C/D box 10	-1.520725751	1.520725751	down	7.02768E-07	8.81466E-06
IL6ST	interleukin 6 signal transducer	-1.52130561	1.52130561	down	9.71804E-06	7.78474E-05
SRSF10	serine/arginine-rich splicing factor 10	-1.521344275	1.521344275	down	1.91749E-10	1.17067E-08
MYZAP	myocardial zonula adherens protein	-1.521527068	1.521527068	down	5.73106E-06	5.0187E-05
GCOM1	GRINL1A complex locus 1	-1.521527068	1.521527068	down	5.73106E-06	5.0187E-05
FKBP14	FK506 binding protein 14	-1.52167824	1.52167824	down	2.29964E-07	3.50968E-06
FLOT1	flotillin 1	-1.521723948	1.521723948	down	1.20841E-08	3.18172E-07
SNRPG	small nuclear ribonucleoprotein polypeptide G	-1.521906787	1.521906787	down	0.000563918	0.002424268
HNRNPU	heterogeneous nuclear ribonucleoprotein U (scaffold attachment factor A)	-1.522145918	1.522145918	down	2.40537E-07	3.63293E-06
EZH2	enhancer of zeste 2 polycomb repressive complex 2 subunit	-1.522297152	1.522297152	down	6.07849E-11	4.70054E-09
IL1A	interleukin 1 alpha	-1.522402673	1.522402673	down	8.44902E-06	6.96122E-05
COMMD3-BMI1	COMMD3-BMI1 readthrough	-1.522736872	1.522736872	down	6.05546E-05	0.000365524
BMI1	BMI1 proto-oncogene, polycomb ring finger	-1.522736872	1.522736872	down	6.05546E-05	0.000365524
PVR	poliovirus receptor	-1.523057068	1.523057068	down	4.78309E-08	9.66559E-07
SLC22A4	solute carrier family 22 (organic cation/zwitterion transporter), member 4	-1.523109854	1.523109854	down	1.03353E-06	1.20801E-05

S1PR1	sphingosine-1-phosphate receptor 1	-1.523218951	1.523218951	down	4.50677E-05	0.000284088
PPIA	peptidylprolyl isomerase A (cyclophilin A)	-1.52341605	1.52341605	down	1.93779E-06	2.04251E-05
FYTTD1	forty-two-three domain containing 1	-1.52367654	1.52367654	down	9.70914E-07	1.1447E-05
PIGT	phosphatidylinositol glycan anchor biosynthesis class T	-1.523711745	1.523711745	down	2.31056E-09	8.31209E-08
PADI1	peptidyl arginine deiminase, type I	-1.524000455	1.524000455	down	3.04698E-05	0.000203083
DDX23	DEAD (Asp-Glu-Ala-Asp) box polypeptide 23	-1.524542814	1.524542814	down	0.000276487	0.00132684
PGS1	phosphatidylglycerophosphate synthase 1	-1.524648491	1.524648491	down	8.70969E-05	0.000496447
METAP1	methionyl aminopeptidase 1	-1.524849298	1.524849298	down	6.026E-06	5.23845E-05
HSPA13	heat shock protein family A (Hsp70) member 13	-1.525057178	1.525057178	down	1.28523E-05	9.84385E-05
MCM7	minichromosome maintenance complex component 7	-1.525539991	1.525539991	down	4.21992E-08	8.72901E-07
IKBKE	inhibitor of kappa light polypeptide gene enhancer in B-cells, kinase epsilon	-1.526015906	1.526015906	down	0.00013976	0.000742639
TGFBI	transforming growth factor beta induced	-1.526058217	1.526058217	down	1.6474E-13	5.13623E-11
CDK1	cyclin-dependent kinase 1	-1.52618163	1.52618163	down	3.64124E-05	0.000237178
C4orf19	chromosome 4 open reading frame 19	-1.526185156	1.526185156	down	4.19181E-08	8.6806E-07
COL16A1	collagen, type XVI, alpha 1	-1.5265872	1.5265872	down	9.39261E-06	7.58593E-05
IFITM3	interferon induced transmembrane protein 3	-1.526710656	1.526710656	down	3.97868E-12	5.85388E-10
SIN3A	SIN3 transcription regulator family member A	-1.527017575	1.527017575	down	4.84586E-06	4.35617E-05
EFHD1	EF-hand domain family member D1	-1.52708814	1.52708814	down	1.1211E-08	3.00538E-07
SNX3	sorting nexin 3	-1.527363374	1.527363374	down	6.36325E-08	1.21237E-06
LETM2	leucine zipper-EF-hand containing transmembrane protein 2	-1.527536303	1.527536303	down	1.76779E-05	0.000128699
KLHDC10	kelch domain containing 10	-1.527772788	1.527772788	down	3.75225E-06	3.51128E-05
MXD1	MAX dimerization protein 1	-1.527797497	1.527797497	down	3.53427E-09	1.18061E-07
PSD3	pleckstrin and Sec7 domain containing 3	-1.528048145	1.528048145	down	0.000188443	0.000957545
RAB29	RAB29, member RAS oncogene family	-1.528560159	1.528560159	down	3.12941E-05	0.000207872
MFGE8	milk fat globule-EGF factor 8 protein	-1.528630795	1.528630795	down	1.93845E-05	0.000138859
LOC102724985	pyridoxal-dependent decarboxylase domain-containing protein 1	-1.528906308	1.528906308	down	4.08678E-06	3.77931E-05
PDXDC1	pyridoxal-dependent decarboxylase domain containing 1	-1.528906308	1.528906308	down	4.08678E-06	3.77931E-05
PKN2	protein kinase N2	-1.529037016	1.529037016	down	4.26709E-06	3.91655E-05
IL4R	interleukin 4 receptor	-1.529733142	1.529733142	down	1.01818E-06	1.19362E-05
LMNB1	lamin B1	-1.529867456	1.529867456	down	1.50205E-05	0.000112436
NUCKS1	nuclear casein kinase and cyclin-dependent kinase substrate 1	-1.53046848	1.53046848	down	2.43606E-10	1.41775E-08
NELFCD	negative elongation factor complex member C/D	-1.530677127	1.530677127	down	1.21887E-08	3.19227E-07
NFAT5	nuclear factor of activated T-cells 5, tonicity-responsive	-1.531052054	1.531052054	down	1.71121E-11	1.74932E-09
MIR22HG	MIR22 host gene	-1.531098042	1.531098042	down	2.99449E-09	1.02649E-07
AK3	adenylate kinase 3	-1.531267855	1.531267855	down	5.48518E-09	1.66394E-07
PISD	phosphatidylserine decarboxylase	-1.53139169	1.53139169	down	1.36309E-06	1.51608E-05
HPSE	heparanase	-1.531596923	1.531596923	down	2.08077E-07	3.22577E-06
ELF1	E74-like factor 1 (ets domain transcription factor)	-1.531795105	1.531795105	down	8.6492E-05	0.000494075
INPP1	inositol polyphosphate-1-phosphatase	-1.531830497	1.531830497	down	1.86227E-10	1.14308E-08
GSDMB	gasdermin B	-1.532347319	1.532347319	down	6.8864E-09	2.02489E-07
TMEM140	transmembrane protein 140	-1.5324677	1.5324677	down	4.41973E-07	5.99532E-06
HMGN2	high mobility group nucleosomal binding domain 2	-1.532485404	1.532485404	down	3.16075E-06	3.05529E-05
USP42	ubiquitin specific peptidase 42	-1.532843066	1.532843066	down	2.7051E-06	2.69167E-05
GANAB	glucosidase, alpha; neutral AB	-1.533016615	1.533016615	down	8.22853E-10	3.64771E-08
DEK	DEK proto-oncogene	-1.533392115	1.533392115	down	6.61478E-08	1.25051E-06
SLFN5	schlafen family member 5	-1.533594073	1.533594073	down	1.14225E-06	1.31205E-05
ZNF146	zinc finger protein 146	-1.533764164	1.533764164	down	4.69401E-07	6.29564E-06
LYPD6	LY6/PLAUR domain containing 6	-1.534100857	1.534100857	down	2.36472E-05	0.000164272
TPST2	tyrosylprotein sulfotransferase 2	-1.534629082	1.534629082	down	3.28006E-08	7.06823E-07
MBD4	methyl-CpG binding domain 4 DNA glycosylase	-1.53466454	1.53466454	down	7.96759E-07	9.74985E-06
PLSCR1	phospholipid scramblase 1	-1.534834749	1.534834749	down	1.22648E-08	3.2078E-07
ATP5A1	ATP synthase, H+ transporting, mitochondrial F1 complex, alpha subunit 1, cardiac muscle	-1.534905675	1.534905675	down	1.13069E-09	4.69458E-08
RBM48	RNA binding motif protein 48	-1.535405797	1.535405797	down	0.000126588	0.000683183
RHEB	Ras homolog enriched in brain	-1.535501583	1.535501583	down	1.6404E-10	1.02777E-08
PADI3	peptidyl arginine deiminase, type III	-1.536143863	1.536143863	down	1.89199E-09	7.0584E-08
MIPOL1	mirror-image polydactyly 1	-1.537006572	1.537006572	down	9.69697E-06	7.77262E-05
MAP4K3	mitogen-activated protein kinase kinase kinase kinase 3	-1.537262282	1.537262282	down	2.62577E-05	0.000179486
PSTPIP2	proline-serine-threonine phosphatase interacting protein 2	-1.537571322	1.537571322	down	3.04779E-07	4.4148E-06
NUFIP2	nuclear fragile X mental retardation protein interacting protein 2	-1.537770278	1.537770278	down	3.66582E-09	1.21117E-07
CDKN1C	cyclin-dependent kinase inhibitor 1C (p57, Kip2)	-1.538232237	1.538232237	down	9.57733E-05	0.000537864
GPCPD1	glycerophosphocholine phosphodiesterase 1	-1.539021444	1.539021444	down	3.08779E-06	2.99804E-05
HSPBAP1	HSPB (heat shock 27kDa) associated protein 1	-1.539135237	1.539135237	down	3.64165E-06	3.42902E-05
RDM1	RAD52 motif containing 1	-1.539430426	1.539430426	down	6.51686E-05	0.000388956
GIT2	G protein-coupled receptor kinase interacting ArfGAP 2	-1.539896442	1.539896442	down	0.00014322	0.000758257
SLPI	secretory leukocyte peptidase inhibitor	-1.5408645	1.5408645	down	5.121E-13	1.23419E-10
PRKAR1A	protein kinase, cAMP-dependent, regulatory subunit type I alpha	-1.541092366	1.541092366	down	9.0233E-06	7.32077E-05
TMEM9	transmembrane protein 9	-1.541373686	1.541373686	down	1.05923E-09	4.44634E-08
ASS1	argininosuccinate synthase 1	-1.541519707	1.541519707	down	3.01191E-11	2.68908E-09
HIP1	huntingtin interacting protein 1	-1.541562448	1.541562448	down	2.06722E-05	0.00014664
RTP4	receptor (chemosensory) transporter protein 4	-1.542046924	1.542046924	down	3.71099E-10	1.95681E-08
LIPA	lipase A, lysosomal acid, cholesterol esterase	-1.542349799	1.542349799	down	3.65227E-07	5.11501E-06
TRIM37	tripartite motif containing 37	-1.542545809	1.542545809	down	0.000292891	0.00139449
AZIN1	antizyme inhibitor 1	-1.544717835	1.544717835	down	1.08685E-12	2.18954E-10
GALNT10	polypeptide N-acetylgalactosaminyltransferase 10	-1.545517511	1.545517511	down	4.48429E-06	4.07969E-05
RDH11	retinol dehydrogenase 11 (all-trans/9-cis/11-cis)	-1.545606786	1.545606786	down	1.05485E-09	4.44336E-08
TMEM67	transmembrane protein 67	-1.545863927	1.545863927	down	0.000792226	0.003248962
OLA1	Obg-like ATPase 1	-1.54667849	1.54667849	down	5.4912E-06	4.83995E-05
EML2	echinoderm microtubule associated protein like 2	-1.546971552	1.546971552	down	2.73E-06	2.71163E-05
HSPA4	heat shock protein family A (Hsp70) member 4	-1.54723607	1.54723607	down	4.21221E-05	0.000268487
HIBADH	3-hydroxyisobutyrate dehydrogenase	-1.547432701	1.547432701	down	2.6633E-09	9.35848E-08
SCAF8	SR-related CTD-associated factor 8	-1.54830175	1.54830175	down	2.20154E-07	3.38363E-06
TIAM2	T-cell lymphoma invasion and metastasis 2	-1.54830175	1.54830175	down	2.20154E-07	3.38363E-06
RHPN2	rhophilin, Rho GTPase binding protein 2	-1.548430539	1.548430539	down	4.76394E-06	4.29333E-05
UTP6	UTP6 small subunit processome component	-1.54845916	1.54845916	down	2.91943E-06	2.86288E-05
MARCH9	membrane associated ring-CH-type finger 9	-1.548620165	1.548620165	down	1.36818E-08	3.49082E-07
LACTB2	lactamase beta 2	-1.548677415	1.548677415	down	8.64448E-10	3.78162E-08
OPTN	optineurin	-1.54914981	1.54914981	down	2.69164E-08	6.03739E-07
PRDM1	PR domain containing 1, with ZNF domain	-1.549493461	1.549493461	down	1.28588E-05	9.84385E-05
PLK4	polo-like kinase 4	-1.550066381	1.550066381	down	8.81989E-05	0.000501683
IVNS1ABP	influenza virus NS1A binding protein	-1.550327847	1.550327847	down	7.41488E-12	9.27663E-10
CLTC	clathrin, heavy chain (Hc)	-1.55044964	1.55044964	down	1.7401E-10	1.07991E-08
TRAM1	translocation associated membrane protein 1	-1.551026497	1.551026497	down	1.36157E-11	1.46542E-09
PIK3C2A	phosphatidylinositol-4-phosphate 3-kinase catalytic subunit type 2 alpha	-1.55163942	1.55163942	down	1.02454E-06	1.19964E-05
SLC2A6	solute carrier family 2 (facilitated glucose transporter), member 6	-1.551675271	1.551675271	down	4.96724E-08	9.94055E-07
SLC3A2	solute carrier family 3 (amino acid transporter heavy chain), member 2	-1.552058927	1.552058927	down	8.32487E-10	3.68099E-08
ELOVL7	ELOVL fatty acid elongase 7	-1.552449852	1.552449852	down	1.51313E-08	3.76452E-07
FKBP10	FK506 binding protein 10	-1.552661495	1.552661495	down	3.84562E-08	8.07005E-07

ACTN4	actinin, alpha 4	-1.552736832	1.552736832	down	3.60691E-06	3.40201E-05
MUC15	mucin 15, cell surface associated	-1.552855227	1.552855227	down	6.51618E-09	1.93486E-07
AAGAB	alpha- and gamma-adaptin binding protein	-1.553282241	1.553282241	down	5.34385E-06	4.7313E-05
ABHD4	abhydrolase domain containing 4	-1.553896054	1.553896054	down	3.50855E-07	4.94191E-06
GOLGA8A	golgin A8 family member A	-1.553992995	1.553992995	down	8.62579E-06	7.07719E-05
NXF3	nuclear RNA export factor 3	-1.553992995	1.553992995	down	8.62579E-06	7.07719E-05
GOLGA8B	golgin A8 family member B	-1.553992995	1.553992995	down	8.62579E-06	7.07719E-05
ARHGAP29	Rho GTPase activating protein 29	-1.554380815	1.554380815	down	1.03997E-06	1.21445E-05
SLC38A2	solute carrier family 38, member 2	-1.554686113	1.554686113	down	7.53899E-14	2.82059E-11
MAP3K13	mitogen-activated protein kinase kinase kinase 13	-1.554833395	1.554833395	down	4.95411E-05	0.000307535
GPRC5B	G protein-coupled receptor, class C, group 5, member B	-1.555526889	1.555526889	down	0.000211839	0.001058091
SRP9	signal recognition particle 9kDa	-1.556033729	1.556033729	down	1.48667E-09	5.82276E-08
LIMK2	LIM domain kinase 2	-1.556073277	1.556073277	down	5.19277E-06	4.61835E-05
MARCKSL1	MARCKS-like 1	-1.556472406	1.556472406	down	2.11051E-11	2.0567E-09
HIST1H2BC	histone cluster 1, H2bc	-1.557109066	1.557109066	down	7.61929E-07	9.40654E-06
FAM111A	family with sequence similarity 111 member A	-1.557224196	1.557224196	down	3.90429E-06	3.63021E-05
LAMA3	laminin subunit alpha 3	-1.55723499	1.55723499	down	7.74258E-11	5.70656E-09
SVIL	supervillin	-1.55753725	1.55753725	down	4.89037E-06	4.38715E-05
CERS2	ceramide synthase 2	-1.557720793	1.557720793	down	3.19383E-09	1.08161E-07
ZNF211	zinc finger protein 211	-1.557763983	1.557763983	down	1.36313E-05	0.000103677
TMSB4X	thymosin beta 4, X-linked	-1.558213947	1.558213947	down	2.1927E-11	2.10137E-09
OTUD1	OTU deubiquitinase 1	-1.558260751	1.558260751	down	5.51527E-13	1.3131E-10
ZNHIT6	zinc finger, HIT-type containing 6	-1.558613625	1.558613625	down	0.000154863	0.00081191
IGFBP7	insulin like growth factor binding protein 7	-1.559330421	1.559330421	down	5.33446E-08	1.04937E-06
SERTAD4	SERTA domain containing 4	-1.559647501	1.559647501	down	6.23698E-08	1.19286E-06
SRGAP2	SLIT-ROBO Rho GTPase activating protein 2	-1.559950229	1.559950229	down	1.63938E-05	0.000120987
EIF2AK1	eukaryotic translation initiation factor 2 alpha kinase 1	-1.56025662	1.56025662	down	1.38004E-10	8.97574E-09
HIST1H3D	histone cluster 1, H3d	-1.560595523	1.560595523	down	3.66967E-07	5.13387E-06
TNFAIP8	TNF alpha induced protein 8	-1.560664034	1.560664034	down	3.07757E-05	0.000204823
TIMM23	translocase of inner mitochondrial membrane 23 homolog (yeast)	-1.560689275	1.560689275	down	9.47582E-09	2.62429E-07
TIMM23B	translocase of inner mitochondrial membrane 23 homolog B (yeast)	-1.560689275	1.560689275	down	9.47582E-09	2.62429E-07
RAD1	RAD1 checkpoint DNA exonuclease	-1.560772215	1.560772215	down	0.000115457	0.000631001
HBP1	HMG-box transcription factor 1	-1.561031879	1.561031879	down	4.80886E-06	4.32985E-05
CREBZF	CREB/ATF bZIP transcription factor	-1.56111123	1.56111123	down	5.98322E-10	2.8387E-08
STX16	syntaxin 16	-1.561760612	1.561760612	down	4.27444E-07	5.83653E-06
CERCAM	cerebral endothelial cell adhesion molecule	-1.562117888	1.562117888	down	1.10554E-06	1.2766E-05
LGALS3BP	lectin, galactoside-binding, soluble, 3 binding protein	-1.562821852	1.562821852	down	1.17299E-12	2.30283E-10
CCDC64	coiled-coil domain containing 64	-1.563627287	1.563627287	down	1.0773E-07	1.8726E-06
RSRP1	arginine/serine-rich protein 1	-1.563746512	1.563746512	down	4.84853E-05	0.000301854
CLMN	calmin (calponin-like, transmembrane)	-1.563750125	1.563750125	down	4.49389E-08	9.16129E-07
PIBF1	progesterone immunomodulatory binding factor 1	-1.564223504	1.564223504	down	1.25536E-05	9.66779E-05
THSD4	thrombospondin type 1 domain containing 4	-1.56467895	1.56467895	down	4.37225E-07	5.94324E-06
RHBDF1	rhomboid 5 homolog 1 (Drosophila)	-1.564711487	1.564711487	down	0.001156974	0.004504071
ZEB1-AS1	ZEB1 antisense RNA 1	-1.565199622	1.565199622	down	2.23174E-05	0.000156417
ZEB1-AS1	ZEB1 antisense RNA 1	-1.565199622	1.565199622	down	2.23174E-05	0.000156417
CNPY3	canopy FGF signaling regulator 3	-1.565203238	1.565203238	down	2.78441E-07	4.09521E-06
SDHAP1	succinate dehydrogenase complex subunit A, flavoprotein pseudogene 1	-1.565441938	1.565441938	down	2.71407E-12	4.41574E-10
SDHAP2	succinate dehydrogenase complex subunit A, flavoprotein pseudogene 2	-1.565441938	1.565441938	down	2.71407E-12	4.41574E-10
SDHA	succinate dehydrogenase complex subunit A, flavoprotein (Fp)	-1.565441938	1.565441938	down	2.71407E-12	4.41574E-10
SAMHD1	SAM domain and HD domain 1	-1.56668304	1.56668304	down	4.25982E-08	8.789E-07
ETHE1	ethylmalonic encephalopathy 1	-1.566950928	1.566950928	down	1.52788E-09	5.91927E-08
FBXO11	F-box protein 11	-1.566958169	1.566958169	down	2.6998E-05	0.000183684
PTTG1IP	pituitary tumor-transforming 1 interacting protein	-1.56703058	1.56703058	down	3.24724E-08	7.01676E-07
GAD1	glutamate decarboxylase 1	-1.567910635	1.567910635	down	1.03907E-05	8.23295E-05
DKFZp686K168	uncharacterized LOC440034	-1.568243953	1.568243953	down	3.90238E-09	1.27115E-07
RCN1	reticulocalbin 1	-1.568243953	1.568243953	down	3.90238E-09	1.27115E-07
MAP3K7	mitogen-activated protein kinase kinase kinase 7	-1.568265694	1.568265694	down	2.11021E-06	2.19247E-05
IQCK	IQ motif containing K	-1.568570094	1.568570094	down	4.58113E-06	4.15432E-05
ZNF331	zinc finger protein 331	-1.568812932	1.568812932	down	1.48843E-08	3.71247E-07
TRAPPC13	trafficking protein particle complex 13	-1.568896303	1.568896303	down	2.20468E-06	2.26962E-05
CAPZA2	capping protein (actin filament) muscle Z-line, alpha 2	-1.570404995	1.570404995	down	1.33587E-09	5.35492E-08
B4GALNT4	beta-1,4-N-acetyl-galactosaminyl transferase 4	-1.570477565	1.570477565	down	2.06962E-07	3.21101E-06
PTK7	protein tyrosine kinase 7 (inactive)	-1.570931202	1.570931202	down	1.13646E-07	1.95467E-06
EEF1A1	eukaryotic translation elongation factor 1 alpha 1	-1.571958721	1.571958721	down	6.75095E-07	8.53022E-06
HSPA9	heat shock protein family A (Hsp70) member 9	-1.572064052	1.572064052	down	5.22445E-11	4.20568E-09
STC1	stanniocalcin 1	-1.572325595	1.572325595	down	0.000382038	0.001745579
USP37	ubiquitin specific peptidase 37	-1.572485448	1.572485448	down	1.99529E-05	0.000142178
CDH5	cadherin 5, type 2 (vascular endothelium)	-1.572641684	1.572641684	down	3.98001E-10	2.05995E-08
SP100	SP100 nuclear antigen	-1.572863348	1.572863348	down	2.48039E-11	2.30354E-09
TNIP1	TNFAIP3 interacting protein 1	-1.573059601	1.573059601	down	8.53411E-07	1.0325E-05
MYD88	myeloid differentiation primary response 88	-1.573299499	1.573299499	down	7.56052E-06	6.35039E-05
IRAK2	interleukin 1 receptor associated kinase 2	-1.573866676	1.573866676	down	2.84911E-05	0.000191848
B4GAT1	beta-1,4-glucuronyltransferase 1	-1.574270368	1.574270368	down	7.40644E-07	9.2191E-06
CASP1	caspase 1	-1.574324929	1.574324929	down	8.73725E-06	7.14178E-05
BAG5	BCL2 associated athanogene 5	-1.575078064	1.575078064	down	6.35436E-09	1.90118E-07
ARHGAP12	Rho GTPase activating protein 12	-1.575285512	1.575285512	down	9.20157E-07	1.09405E-05
DNAJC6	DnaJ heat shock protein family (Hsp40) member C6	-1.575358308	1.575358308	down	8.72051E-06	7.13255E-05
AP5M1	adaptor-related protein complex 5, mu 1 subunit	-1.575442026	1.575442026	down	5.76032E-08	1.1173E-06
SLC25A29	solute carrier family 25 (mitochondrial carnitine/acylcarnitine carrier), member 29	-1.575809714	1.575809714	down	1.11643E-06	1.28691E-05
MAEA	macrophage erythroblast attacher	-1.575911662	1.575911662	down	4.88052E-06	4.38032E-05
ABAT	4-aminobutyrate aminotransferase	-1.576144712	1.576144712	down	9.30676E-08	1.66185E-06
PTK2	protein tyrosine kinase 2	-1.576640056	1.576640056	down	3.77151E-07	5.25124E-06
PAM	peptidylglycine alpha-amidating monooxygenase	-1.576650985	1.576650985	down	6.13477E-06	5.32123E-05
SEC23A	Sec23 homolog A, COPII coat complex component	-1.576680128	1.576680128	down	7.39494E-08	1.37484E-06
KMT2C	lysine (K)-specific methyltransferase 2C	-1.5771392	1.5771392	down	1.89665E-06	2.0056E-05
PDZK1P1	PDZ domain containing 1 pseudogene 1	-1.577973889	1.577973889	down	5.24583E-09	1.60747E-07
PDZK1	PDZ domain containing 1	-1.577973889	1.577973889	down	5.24583E-09	1.60747E-07
ZNF655	zinc finger protein 655	-1.578747008	1.578747008	down	3.28937E-07	4.68278E-06
FRMD3	FERM domain containing 3	-1.579286957	1.579286957	down	0.00012915	0.000694717
AP3M1	adaptor-related protein complex 3, mu 1 subunit	-1.57968839	1.57968839	down	7.55164E-05	0.000441719
RICTOR	RPTOR independent companion of MTOR, complex 2	-1.579827091	1.579827091	down	2.99181E-06	2.92146E-05
CCL5	chemokine (C-C motif) ligand 5	-1.580137386	1.580137386	down	1.08603E-07	1.88495E-06
SPTSSA	serine palmitoyltransferase small subunit A	-1.580487911	1.580487911	down	3.47507E-08	7.41926E-07
PTGS2	prostaglandin-endoperoxide synthase 2 (prostaglandin G/H synthase and cyclooxygenase)	-1.580549991	1.580549991	down	3.27334E-06	3.1397E-05
SETD3	SET domain containing 3	-1.580747203	1.580747203	down	4.10274E-07	5.63342E-06
ADIPOR1	adiponectin receptor 1	-1.58167516	1.58167516	down	3.02643E-09	1.03563E-07
ZRANB2	zinc finger, RAN-binding domain containing 2	-1.582621944	1.582621944	down	2.65989E-07	3.94455E-06

HPS3	Hermansky-Pudlak syndrome 3	-1.582753589	1.582753589	down	2.9216E-08	6.44426E-07
SRPX2	sushi-repeat containing protein, X-linked 2	-1.582888901	1.582888901	down	6.25436E-12	8.18987E-10
RAP1GAP2	RAP1 GTPase activating protein 2	-1.582991307	1.582991307	down	2.79291E-08	6.23745E-07
CYR61	cysteine-rich, angiogenic inducer, 61	-1.583060801	1.583060801	down	1.29083E-13	4.37146E-11
KLHL5	kelch like family member 5	-1.583090063	1.583090063	down	1.36148E-07	2.27788E-06
XPR1	xenotropic and polytropic retrovirus receptor 1	-1.583755907	1.583755907	down	2.33437E-05	0.000162364
SPNS2	spinster homolog 2 (Drosophila)	-1.584154816	1.584154816	down	2.57708E-09	9.09597E-08
WDR45B	WD repeat domain 45B	-1.584389084	1.584389084	down	1.06234E-08	2.87243E-07
WNT5A	wingless-type MMTV integration site family member 5A	-1.584531858	1.584531858	down	5.17877E-07	6.83152E-06
GUSB	glucuronidase, beta	-1.584649016	1.584649016	down	1.21852E-12	2.36302E-10
SNORA21	small nucleolar RNA, H/ACA box 21	-1.585223947	1.585223947	down	2.45852E-06	2.48662E-05
RPL23	ribosomal protein L23	-1.585223947	1.585223947	down	2.45852E-06	2.48662E-05
AFAP1L2	actin filament associated protein 1 like 2	-1.585359471	1.585359471	down	1.29047E-07	2.18445E-06
GNG4	guanine nucleotide binding protein (G protein), gamma 4	-1.585795423	1.585795423	down	2.92704E-09	1.01042E-07
FANCL	Fanconi anemia complementation group L	-1.585982296	1.585982296	down	3.38827E-07	4.80003E-06
HDAC7	histone deacetylase 7	-1.586477067	1.586477067	down	7.93367E-07	9.71528E-06
FOXO3	forkhead box O3	-1.587676151	1.587676151	down	8.83543E-10	3.84801E-08
FOXO3B	forkhead box O3B pseudogene	-1.587676151	1.587676151	down	8.83543E-10	3.84801E-08
PPP1CB	protein phosphatase 1, catalytic subunit, beta isozyme	-1.588997293	1.588997293	down	2.18596E-05	0.000153592
EXOSC10	exosome component 10	-1.589941113	1.589941113	down	1.63793E-06	1.77257E-05
CCNG1	cyclin G1	-1.590521639	1.590521639	down	3.89997E-06	3.62704E-05
HIST2H4A	histone cluster 2, H4a	-1.590617189	1.590617189	down	3.00303E-07	4.358E-06
HIST2H4B	histone cluster 2, H4b	-1.590617189	1.590617189	down	3.00303E-07	4.358E-06
GRHL1	grainyhead-like transcription factor 1	-1.590878143	1.590878143	down	6.58613E-11	4.97556E-09
CLK1	CDC like kinase 1	-1.590881819	1.590881819	down	7.17122E-06	6.07536E-05
JADE1	jade family PHD finger 1	-1.591006798	1.591006798	down	3.63087E-06	3.42051E-05
TAP2	transporter 2, ATP-binding cassette, sub-family B (MDR/TAP)	-1.591918709	1.591918709	down	1.81903E-09	6.87103E-08
BAGE2	B melanoma antigen family member 2	-1.591937099	1.591937099	down	2.43815E-05	0.00016836
RAPGEF3	Rap guanine nucleotide exchange factor 3	-1.592518354	1.592518354	down	2.22887E-07	3.42027E-06
PRC1	protein regulator of cytokinesis 1	-1.592985719	1.592985719	down	6.84095E-07	8.62591E-06
UXS1	UDP-glucuronate decarboxylase 1	-1.593640997	1.593640997	down	8.36908E-09	2.37551E-07
SNRPB	small nuclear ribonucleoprotein polypeptides B and B1	-1.593707276	1.593707276	down	2.41108E-05	0.000166872
SKIL	SKI-like proto-oncogene	-1.594215507	1.594215507	down	3.81876E-06	3.56672E-05
ITGA2	integrin, alpha 2 (CD49B, alpha 2 subunit of VLA-2 receptor)	-1.594701792	1.594701792	down	6.74525E-10	3.11375E-08
MCM3	minichromosome maintenance complex component 3	-1.594889715	1.594889715	down	5.73965E-11	4.52764E-09
TMEM30A	transmembrane protein 30A	-1.595037121	1.595037121	down	8.10229E-07	9.88172E-06
SLC1A3	solute carrier family 1 (glial high affinity glutamate transporter), member 3	-1.595059233	1.595059233	down	7.99057E-08	1.46173E-06
NPTN	neuroplastin	-1.59535409	1.59535409	down	1.39159E-09	5.50861E-08
PSMB9	proteasome subunit beta 9	-1.596002967	1.596002967	down	1.03085E-05	8.18096E-05
ABHD3	abhydrolase domain containing 3	-1.596187355	1.596187355	down	3.14646E-07	4.52767E-06
PRDM8	PR domain containing 8	-1.596497175	1.596497175	down	6.37671E-06	5.49951E-05
DPYD	dihydropyrimidine dehydrogenase	-1.596530374	1.596530374	down	1.05006E-07	1.83402E-06
SRSF3	serine/arginine-rich splicing factor 3	-1.596659486	1.596659486	down	6.41055E-06	5.52142E-05
APOD	apolipoprotein D	-1.596855018	1.596855018	down	4.57393E-11	3.75904E-09
UBA3	ubiquitin-like modifier activating enzyme 3	-1.597331037	1.597331037	down	2.4925E-07	3.7284E-06
PSEN1	presenilin 1	-1.597393779	1.597393779	down	8.22619E-07	1.00204E-05
DDX5	DEAD (Asp-Glu-Ala-Asp) box helicase 5	-1.597404851	1.597404851	down	1.35721E-05	0.000103267
PMS1	PMS1 homolog 1, mismatch repair system component	-1.597689067	1.597689067	down	1.77827E-05	0.000129198
KCTD20	potassium channel tetramerization domain containing 20	-1.598741475	1.598741475	down	2.74456E-05	0.000186064
SOD2	superoxide dismutase 2, mitochondrial	-1.598759945	1.598759945	down	1.93475E-09	7.20424E-08
LOC100129518	uncharacterized LOC100129518	-1.598759945	1.598759945	down	1.93475E-09	7.20424E-08
RBMX	RNA binding motif protein, X-linked	-1.599048097	1.599048097	down	8.42691E-09	2.38675E-07
RARRES3	retinoic acid receptor responder (tazarotene induced) 3	-1.599724349	1.599724349	down	0.000153456	0.000805931
RGL2	ral guanine nucleotide dissociation stimulator-like 2	-1.600537708	1.600537708	down	2.98147E-09	1.02405E-07
RAPH1	Ras association (RalGDS/AF-6) and pleckstrin homology domains 1	-1.600988931	1.600988931	down	2.80089E-06	2.76597E-05
BCL6	B-cell CLL/lymphoma 6	-1.601473583	1.601473583	down	2.29199E-07	3.49935E-06
TOP2A	topoisomerase (DNA) II alpha	-1.601577191	1.601577191	down	3.76796E-10	1.98419E-08
ANO6	anoctamin 6	-1.601913966	1.601913966	down	7.98312E-11	5.84002E-09
ZNF189	zinc finger protein 189	-1.601939874	1.601939874	down	6.70824E-06	5.73507E-05
HSPB8	heat shock protein family B (small) member 8	-1.602154562	1.602154562	down	8.01879E-08	1.46516E-06
WAPL	WAPL cohesin release factor	-1.602180475	1.602180475	down	2.91212E-06	2.85714E-05
APMAP	adipocyte plasma membrane associated protein	-1.604373453	1.604373453	down	1.74472E-07	2.7839E-06
FAM49B	family with sequence similarity 49 member B	-1.605185468	1.605185468	down	1.42868E-08	3.60324E-07
NDFIP2	Nedd4 family interacting protein 2	-1.605727039	1.605727039	down	1.18847E-10	7.95361E-09
STIP1	stress induced phosphoprotein 1	-1.605745589	1.605745589	down	3.64768E-09	1.20721E-07
CDC23	cell division cycle 23	-1.607597977	1.607597977	down	1.2947E-06	1.45441E-05
NSUN7	NOP2/Sun RNA methyltransferase family member 7	-1.607958308	1.607958308	down	3.38775E-06	3.22707E-05
ARRDC3	arrestin domain containing 3	-1.608066052	1.608066052	down	8.92909E-07	1.07054E-05
ANXA8	annexin A8	-1.608746117	1.608746117	down	9.47817E-07	1.12218E-05
LOC102725207	annexin A8-like	-1.608746117	1.608746117	down	9.47817E-07	1.12218E-05
IL7R	interleukin 7 receptor	-1.608775854	1.608775854	down	1.56973E-09	6.06346E-08
TPD52L2	tumor protein D52-like 2	-1.608939413	1.608939413	down	1.3577E-06	1.51093E-05
SSBP2	single-stranded DNA binding protein 2	-1.609021198	1.609021198	down	3.47731E-07	4.90426E-06
CXADR	coxsackie virus and adenovirus receptor	-1.609337228	1.609337228	down	0.00038858	0.001769851
GNE	glucosamine (UDP-N-acetyl)-2-epimerase/N-acetylmannosamine kinase	-1.610494053	1.610494053	down	1.43848E-05	0.000108467
PEX1	peroxisomal biogenesis factor 1	-1.61078432	1.61078432	down	7.49563E-06	6.30767E-05
EIF2AK3	eukaryotic translation initiation factor 2-alpha kinase 3	-1.610981582	1.610981582	down	6.68957E-05	0.000397388
SLC31A2	solute carrier family 31 (copper transporter), member 2	-1.610981582	1.610981582	down	1.89664E-06	2.0056E-05
SLBP	stem-loop binding protein	-1.612154488	1.612154488	down	1.00133E-08	2.73549E-07
HERPUD1	homocysteine-inducible, endoplasmic reticulum stress-inducible, ubiquitin-like domain member 1	-1.612620164	1.612620164	down	1.99392E-06	2.091E-05
RPL37A	ribosomal protein L37a	-1.613074793	1.613074793	down	1.06788E-05	8.43231E-05
METTL3	methyltransferase like 3	-1.613134426	1.613134426	down	1.265E-07	2.14755E-06
MARCH7	membrane associated ring-CH-type finger 7	-1.613387891	1.613387891	down	3.16333E-06	3.05655E-05
PDCD1LG2	programmed cell death 1 ligand 2	-1.614573741	1.614573741	down	3.15077E-05	0.000209005
RNF157	ring finger protein 157	-1.615667135	1.615667135	down	1.69802E-06	1.82753E-05
PJA2	praja ring finger 2, E3 ubiquitin protein ligase	-1.616047944	1.616047944	down	3.14873E-06	3.04817E-05
TPM1	tropomyosin 1 (alpha)	-1.616384027	1.616384027	down	3.70839E-08	7.8365E-07
IGF2R	insulin like growth factor 2 receptor	-1.616955528	1.616955528	down	1.34092E-07	2.25114E-06
CENPJ	centromere protein J	-1.617101237	1.617101237	down	1.39024E-10	9.01227E-09
TMEM132A	transmembrane protein 132A	-1.617609453	1.617609453	down	1.3509E-09	5.39865E-08
EHD4	EH domain containing 4	-1.618009412	1.618009412	down	4.38414E-06	4.00341E-05
RALY	RALY heterogeneous nuclear ribonucleoprotein	-1.618009412	1.618009412	down	7.9678E-12	9.80132E-10
COL5A1	collagen, type V, alpha 1	-1.618529132	1.618529132	down	3.67824E-10	1.94393E-08
APOBEC3A	apolipoprotein B mRNA editing enzyme, catalytic polypeptide-like 3A	-1.618880693	1.618880693	down	3.71034E-12	5.54784E-10
APOBEC3B	apolipoprotein B mRNA editing enzyme, catalytic polypeptide-like 3B	-1.618880693	1.618880693	down	3.71034E-12	5.54784E-10
APOBEC3A_B	APOBEC3A and APOBEC3B deletion hybrid	-1.618880693	1.618880693	down	3.71034E-12	5.54784E-10
LACTB	lactamase beta	-1.619737473	1.619737473	down	2.86119E-08	6.34772E-07

WARS	tryptophanyl-tRNA synthetase	-1.619849748	1.619849748	down	2.49374E-06	2.51706E-05
CDC42EP5	CDC42 effector protein (Rho GTPase binding) 5	-1.620568496	1.620568496	down	8.23622E-10	3.64771E-08
GALNT3	polypeptide N-acetylgalactosaminyltransferase 3	-1.620845599	1.620845599	down	1.21164E-09	4.94781E-08
RIOK1	RIO kinase 1	-1.621175188	1.621175188	down	3.91954E-07	5.41785E-06
PNN	pinin, desmosome associated protein	-1.622595434	1.622595434	down	1.82148E-13	5.38008E-11
PSMC2	proteasome 26S subunit, ATPase 2	-1.622734152	1.622734152	down	2.14053E-05	0.000150751
FOXJ2	forkhead box J2	-1.62497779	1.62497779	down	1.0161E-05	8.08465E-05
BRIX1	BRX1, biogenesis of ribosomes	-1.625206831	1.625206831	down	8.83874E-11	6.33615E-09
ARID5B	AT-rich interaction domain 5B	-1.625338262	1.625338262	down	2.54531E-09	9E-08
MYL9	myosin light chain 9	-1.625623692	1.625623692	down	7.74986E-10	3.49395E-08
PPP4R4	protein phosphatase 4, regulatory subunit 4	-1.625939226	1.625939226	down	6.36314E-05	0.000381182
GADD45A	growth arrest and DNA damage inducible alpha	-1.627333565	1.627333565	down	1.11405E-13	3.87295E-11
SPAG5	sperm associated antigen 5	-1.628484513	1.628484513	down	3.85982E-07	5.34269E-06
MGLL	monoglyceride lipase	-1.629007599	1.629007599	down	1.19693E-09	4.89914E-08
CEP85	centrosomal protein 85kDa	-1.631037554	1.631037554	down	9.3654E-07	1.11118E-05
OGT	O-linked N-acetylglucosamine (GlcNAc) transferase	-1.632270318	1.632270318	down	3.38242E-08	7.24904E-07
CDK2	cyclin-dependent kinase 2	-1.633002123	1.633002123	down	1.17053E-07	2.008E-06
ARFIP1	ADP ribosylation factor interacting protein 1	-1.634081568	1.634081568	down	5.87763E-06	5.1308E-05
NAB1	NGFI-A binding protein 1	-1.63415708	1.63415708	down	2.50952E-08	5.69239E-07
FAM122C	family with sequence similarity 122C	-1.636049804	1.636049804	down	3.7518E-08	7.91544E-07
TSC22D3	TSC22 domain family member 3	-1.636809776	1.636809776	down	3.08592E-08	6.71619E-07
VWA5A	von Willebrand factor A domain containing 5A	-1.637293922	1.637293922	down	9.27147E-07	1.1008E-05
CD40	CD40 molecule, TNF receptor superfamily member 5	-1.637524698	1.637524698	down	1.18128E-06	1.34938E-05
ADSS	adenylosuccinate synthase	-1.637592803	1.637592803	down	3.63431E-09	1.2038E-07
BMPR1A	bone morphogenetic protein receptor type IA	-1.639629668	1.639629668	down	2.96086E-06	2.89699E-05
HLA-DRA	major histocompatibility complex, class II, DR alpha	-1.639762266	1.639762266	down	9.60385E-07	1.13364E-05
RAD51AP1	RAD51 associated protein 1	-1.640091912	1.640091912	down	6.09823E-08	1.16917E-06
PODXL	podocalyxin-like	-1.641145708	1.641145708	down	4.85025E-09	1.51823E-07
SPINT1	serine peptidase inhibitor, Kunitz type 1	-1.641164667	1.641164667	down	1.62752E-09	6.23154E-08
LIMA1	LIM domain and actin binding 1	-1.641255676	1.641255676	down	5.59516E-07	7.29022E-06
MMP7	matrix metallopeptidase 7	-1.641411159	1.641411159	down	7.7095E-10	3.48115E-08
TSPAN4	tetraspanin 4	-1.641555279	1.641555279	down	1.1317E-09	4.69458E-08
PRKAA1	protein kinase, AMP-activated, alpha 1 catalytic subunit	-1.641964953	1.641964953	down	0.000121224	0.000658029
FAM118A	family with sequence similarity 118 member A	-1.642211564	1.642211564	down	1.08137E-07	1.87801E-06
TAGLN	transgelin	-1.64259104	1.64259104	down	7.64442E-08	1.40921E-06
OAS1	2'-5'-oligoadenylate synthetase 1	-1.643335066	1.643335066	down	1.66677E-12	3.00355E-10
RBPMS	RNA binding protein with multiple splicing	-1.644257974	1.644257974	down	2.6545E-07	3.93804E-06
PTRF	polymerase I and transcript release factor	-1.644907738	1.644907738	down	2.70801E-06	2.69388E-05
SIGMAR1	sigma non-opioid intracellular receptor 1	-1.644995153	1.644995153	down	8.81536E-08	1.58312E-06
RBM47	RNA binding motif protein 47	-1.645348661	1.645348661	down	1.11589E-05	8.7466E-05
SYNPO2	synaptopodin 2	-1.645664221	1.645664221	down	4.9345E-07	6.56218E-06
NECAP1	NECAP endocytosis associated 1	-1.646276503	1.646276503	down	7.0416E-06	5.98214E-05
PDIA4	protein disulfide isomerase family A member 4	-1.646675941	1.646675941	down	1.22109E-12	2.36302E-10
CMTM3	CKLF-like MARVEL transmembrane domain containing 3	-1.646786279	1.646786279	down	2.86149E-07	4.18819E-06
DSG2	desmoglein 2	-1.646953702	1.646953702	down	1.38375E-09	5.49085E-08
CCDC50	coiled-coil domain containing 50	-1.647425624	1.647425624	down	5.14134E-08	1.01852E-06
PSMB8	proteasome subunit beta 8	-1.648377489	1.648377489	down	2.21364E-11	2.11583E-09
KIFC3	kinesin family member C3	-1.64843081	1.64843081	down	7.48605E-11	5.54872E-09
PLPP3	phospholipid phosphatase 3	-1.649051742	1.649051742	down	2.88719E-10	1.59822E-08
VCPIP1	valosin containing protein (p97)/p47 complex interacting protein 1	-1.649223206	1.649223206	down	2.79852E-06	2.76501E-05
NUP160	nucleoporin 160kDa	-1.65010367	1.65010367	down	2.41026E-10	1.40481E-08
KIAA0930	KIAA0930	-1.650885428	1.650885428	down	1.2051E-06	1.37221E-05
RACGAP1	Rac GTPase activating protein 1	-1.650912128	1.650912128	down	4.61197E-09	1.45737E-07
MAP3K8	mitogen-activated protein kinase kinase kinase 8	-1.651885091	1.651885091	down	1.76113E-06	1.88358E-05
PSAP	prosaposin	-1.651907992	1.651907992	down	6.21097E-13	1.44374E-10
AKAP10	A-kinase anchoring protein 10	-1.65253787	1.65253787	down	3.72679E-05	0.000241989
DAP	death-associated protein	-1.653259662	1.653259662	down	4.95069E-10	2.42919E-08
TUBGCP3	tubulin, gamma complex associated protein 3	-1.653909163	1.653909163	down	7.84681E-07	9.62692E-06
ERBB2IP	erbb2 interacting protein	-1.654172856	1.654172856	down	2.20776E-06	2.2722E-05
STMN1	stathmin 1	-1.655010075	1.655010075	down	5.98239E-12	7.99361E-10
EED	embryonic ectoderm development	-1.655908933	1.655908933	down	1.11037E-07	1.9175E-06
PLS3	plastin 3	-1.65603902	1.65603902	down	1.76273E-12	3.11924E-10
TCF12	transcription factor 12	-1.656054325	1.656054325	down	7.31385E-05	0.000429987
ADM	adrenomedullin	-1.656069631	1.656069631	down	1.35422E-09	5.40092E-08
DYSF	dysferlin	-1.656268612	1.656268612	down	4.43242E-07	6.01047E-06
MGEA5	meningioma expressed antigen 5 (hyaluronidase)	-1.656567129	1.656567129	down	9.50455E-11	6.70335E-09
EIF2B1	eukaryotic translation initiation factor 2B subunit alpha	-1.657183468	1.657183468	down	6.5705E-08	1.24333E-06
HELZ	helicase with zinc finger	-1.657256219	1.657256219	down	4.90318E-07	6.52937E-06
EDN1	endothelin 1	-1.657570233	1.657570233	down	7.22622E-13	1.5948E-10
DHRS3	dehydrogenase/reductase (SDR family) member 3	-1.657711942	1.657711942	down	4.99829E-08	9.98235E-07
SUB1	SUB1 homolog, transcriptional regulator	-1.657953257	1.657953257	down	5.91241E-06	5.15911E-05
TVP23C	trans-golgi network vesicle protein 23 homolog C (S. cerevisiae)	-1.658140971	1.658140971	down	4.3132E-11	3.6051E-09
TVP23B	trans-golgi network vesicle protein 23 homolog B (S. cerevisiae)	-1.658140971	1.658140971	down	4.3132E-11	3.6051E-09
SIAH1	siah E3 ubiquitin protein ligase 1	-1.658800055	1.658800055	down	2.67411E-06	2.66488E-05
KBTBD2	kelch repeat and BTB (POZ) domain containing 2	-1.658995531	1.658995531	down	8.39963E-08	1.51851E-06
FGD6	FYVE, RhoGEF and PH domain containing 6	-1.659574429	1.659574429	down	1.12461E-08	3.00948E-07
ERBB3	erb-b2 receptor tyrosine kinase 3	-1.661147291	1.661147291	down	2.36653E-07	3.57977E-06
HIST1H2AE	histone cluster 1, H2ae	-1.661746137	1.661746137	down	1.38281E-08	3.51373E-07
CFHR1	complement factor H-related 1	-1.662279906	1.662279906	down	3.17471E-10	1.71784E-08
CFH	complement factor H	-1.662279906	1.662279906	down	3.17471E-10	1.71784E-08
GNPTAB	N-acetylglucosamine-1-phosphate transferase, alpha and beta subunits	-1.662783111	1.662783111	down	5.49728E-06	4.84204E-05
DNM1L	dynamin 1-like	-1.663509379	1.663509379	down	2.3312E-06	2.37867E-05
CROT	carnitine O-octanoyltransferase	-1.663766915	1.663766915	down	9.04331E-09	2.53213E-07
MSMB	microseminoprotein, beta-	-1.664851309	1.664851309	down	8.771E-06	7.16043E-05
PLCG1	phospholipase C gamma 1	-1.664982099	1.664982099	down	8.36063E-05	0.000480314
TBC1D3J	TBC1 domain family member 3J	-1.665236016	1.665236016	down	5.37308E-07	7.03821E-06
TBC1D3B	TBC1 domain family member 3B	-1.665236016	1.665236016	down	5.37308E-07	7.03821E-06
TBC1D3G	TBC1 domain family member 3G	-1.665236016	1.665236016	down	5.37308E-07	7.03821E-06
TBC1D3H	TBC1 domain family member 3H	-1.665236016	1.665236016	down	5.37308E-07	7.03821E-06
TBC1D3E	TBC1 domain family member 3E	-1.665236016	1.665236016	down	5.37308E-07	7.03821E-06
TBC1D3L	TBC1 domain family member 3L	-1.665236016	1.665236016	down	5.37308E-07	7.03821E-06
TBC1D3	TBC1 domain family member 3	-1.665236016	1.665236016	down	5.37308E-07	7.03821E-06
TBC1D3C	TBC1 domain family member 3C	-1.665236016	1.665236016	down	5.37308E-07	7.03821E-06
TBC1D3K	TBC1 domain family member 3K	-1.665236016	1.665236016	down	5.37308E-07	7.03821E-06
CCL3P1	chemokine (C-C motif) ligand 3 pseudogene 1	-1.665236016	1.665236016	down	5.37308E-07	7.03821E-06
TBC1D3F	TBC1 domain family member 3F	-1.665236016	1.665236016	down	5.37308E-07	7.03821E-06
TBC1D3I	TBC1 domain family member 3I	-1.665236016	1.665236016	down	5.37308E-07	7.03821E-06
LOC101060389	TBC1 domain family member-like	-1.665236016	1.665236016	down	5.37308E-07	7.03821E-06

HLA-B	major histocompatibility complex, class I, B	-1.66676803	1.66676803	down	9.87124E-06	7.88014E-05
CDH3	cadherin 3, type 1, P-cadherin (placental)	-1.66761933	1.66761933	down	8.47529E-12	1.02868E-09
TFRC	transferrin receptor	-1.668652259	1.668652259	down	1.12288E-07	1.93639E-06
SKP2	S-phase kinase-associated protein 2, E3 ubiquitin protein ligase	-1.66901085	1.66901085	down	1.65374E-05	0.000121932
NARS	asparaginyl-tRNA synthetase	-1.669030131	1.669030131	down	2.42411E-07	3.65282E-06
ETV4	ets variant 4	-1.670283892	1.670283892	down	1.8004E-09	6.81377E-08
MYO1E	myosin IE	-1.670913056	1.670913056	down	9.85217E-08	1.74418E-06
MAP1LC3B	microtubule associated protein 1 light chain 3 beta	-1.670997992	1.670997992	down	7.07463E-07	8.86789E-06
HIST1H2AD	histone cluster 1, H2ad	-1.67182055	1.67182055	down	4.85243E-05	0.000302049
SNAP23	synaptosome associated protein 23kDa	-1.672724673	1.672724673	down	1.95887E-06	2.05975E-05
GLS	glutaminase	-1.672972039	1.672972039	down	2.11541E-09	7.7304E-08
ARHGEF3	Rho guanine nucleotide exchange factor 3	-1.673540347	1.673540347	down	1.10939E-06	1.28067E-05
IER5	immediate early response 5	-1.676047845	1.676047845	down	1.6792E-11	1.73164E-09
LYPD5	LY6/PLAUR domain containing 5	-1.677070495	1.677070495	down	5.11165E-07	6.75887E-06
HIVEP2	human immunodeficiency virus type I enhancer binding protein 2	-1.677806879	1.677806879	down	7.02988E-10	3.22619E-08
HLA-DRB5	major histocompatibility complex, class II, DR beta 5	-1.679420302	1.679420302	down	5.60116E-07	7.29563E-06
HLA-DRB3	major histocompatibility complex, class II, DR beta 3	-1.679420302	1.679420302	down	5.60116E-07	7.29563E-06
HLA-DRB1	major histocompatibility complex, class II, DR beta 1	-1.679420302	1.679420302	down	5.60116E-07	7.29563E-06
LOC105369230	HLA class II histocompatibility antigen, DRB1-7 beta chain	-1.679420302	1.679420302	down	5.60116E-07	7.29563E-06
HLA-DRB4	major histocompatibility complex, class II, DR beta 4	-1.679420302	1.679420302	down	5.60116E-07	7.29563E-06
TLE2	transducin like enhancer of split 2	-1.680014091	1.680014091	down	4.53435E-07	6.12879E-06
FBXL2	F-box and leucine-rich repeat protein 2	-1.680732351	1.680732351	down	2.90413E-06	2.85E-05
JAK1	Janus kinase 1	-1.681140148	1.681140148	down	2.16618E-09	7.87927E-08
PTGES3	prostaglandin E synthase 3	-1.681295526	1.681295526	down	5.53975E-10	2.65719E-08
CRK	v-crk avian sarcoma virus CT10 oncogene homolog	-1.681493653	1.681493653	down	6.0927E-07	7.86545E-06
EXOC4	exocyst complex component 4	-1.681610209	1.681610209	down	5.45672E-07	7.13351E-06
WFDC2	WAP four-disulfide core domain 2	-1.682146472	1.682146472	down	7.35053E-08	1.36723E-06
SESTD1	SEC14 and spectrin domain containing 1	-1.682371909	1.682371909	down	1.38344E-09	5.49085E-08
HNRNPA3	heterogeneous nuclear ribonucleoprotein A3	-1.683701824	1.683701824	down	4.28682E-06	3.92824E-05
SEMA3C	sema domain, immunoglobulin domain (Ig), short basic domain, secreted, (semaphorin) 3C	-1.683748507	1.683748507	down	4.46657E-12	6.40383E-10
LUC7L3	LUC7-like 3 pre-mRNA splicing factor	-1.683876892	1.683876892	down	2.60244E-11	2.39986E-09
ATAD2	ATPase family, AAA domain containing 2	-1.684044195	1.684044195	down	5.17665E-09	1.58999E-07
CASP7	caspase 7	-1.684234863	1.684234863	down	2.53028E-06	2.54728E-05
ATP11A	ATPase, class VI, type 11A	-1.684265995	1.684265995	down	1.42104E-08	3.5946E-07
F2RL1	coagulation factor II (thrombin) receptor-like 1	-1.686029758	1.686029758	down	2.75151E-06	2.73024E-05
CDC14B	cell division cycle 14B	-1.686785666	1.686785666	down	3.1884E-07	4.56628E-06
MKRN1	makorin ring finger protein 1	-1.687413248	1.687413248	down	7.80145E-10	3.51014E-08
DHRS2	dehydrogenase/reductase (SDR family) member 2	-1.68839602	1.68839602	down	2.87919E-07	4.20873E-06
ZNF398	zinc finger protein 398	-1.688411624	1.688411624	down	1.20782E-08	3.18172E-07
GPNMB	glycoprotein (transmembrane) nmb	-1.688567673	1.688567673	down	3.60047E-05	0.000234913
NTN4	netrin 4	-1.688899327	1.688899327	down	1.98994E-08	4.69506E-07
PPP4R1	protein phosphatase 4 regulatory subunit 1	-1.688989079	1.688989079	down	8.03854E-10	3.60486E-08
CAST	calpastatin	-1.689020299	1.689020299	down	3.30106E-12	5.10547E-10
HIST1H2BD	histone cluster 1, H2bd	-1.69062888	1.69062888	down	1.78709E-12	3.14816E-10
STAT1	signal transducer and activator of transcription 1	-1.691597891	1.691597891	down	1.26983E-08	3.2992E-07
RAB11A	RAB11A, member RAS oncogene family	-1.692414948	1.692414948	down	1.06039E-09	4.44634E-08
RASSF2	Ras association (RalGDS/AF-6) domain family member 2	-1.693412371	1.693412371	down	1.00583E-06	1.18161E-05
IST1	increased sodium tolerance 1 homolog (yeast)	-1.694390807	1.694390807	down	1.1066E-10	7.50984E-09
PNRC2	proline rich nuclear receptor coactivator 2	-1.694461276	1.694461276	down	9.81975E-08	1.74158E-06
SCEL	sciellin	-1.694625715	1.694625715	down	9.70879E-09	2.67274E-07
ZNF791	zinc finger protein 791	-1.694985972	1.694985972	down	7.43475E-06	6.26349E-05
AKIRIN1	akirin 1	-1.6950408	1.6950408	down	6.28643E-09	1.88372E-07
RAB9A	RAB9A, member RAS oncogene family	-1.696623755	1.696623755	down	7.92087E-10	3.55615E-08
CDKN1B	cyclin-dependent kinase inhibitor 1B (p27, Kip1)	-1.697878629	1.697878629	down	9.69875E-08	1.72371E-06
HACE1	HECT domain and ankyrin repeat containing E3 ubiquitin protein ligase 1	-1.699691992	1.699691992	down	1.30137E-07	2.19885E-06
VCPKMT	valosin containing protein lysine (K) methyltransferase	-1.699813737	1.699813737	down	3.96292E-08	8.28963E-07
CXCL2	chemokine (C-X-C motif) ligand 2	-1.702620217	1.702620217	down	5.18905E-09	1.59131E-07
ARHGEF28	Rho guanine nucleotide exchange factor 28	-1.702899546	1.702899546	down	1.54437E-08	3.8207E-07
CCDC14	coiled-coil domain containing 14	-1.703049065	1.703049065	down	1.37886E-10	8.97574E-09
ERAP2	endoplasmic reticulum aminopeptidase 2	-1.706172285	1.706172285	down	1.15945E-06	1.32793E-05
STK3	serine/threonine kinase 3	-1.70721726	1.70721726	down	1.63118E-05	0.000120427
GRIK1-AS2	GRIK1 antisense RNA 2	-1.707225149	1.707225149	down	1.58286E-13	5.01462E-11
BACH1	BTB and CNC homology 1, basic leucine zipper transcription factor 1	-1.707225149	1.707225149	down	1.58286E-13	5.01462E-11
MGAT2	mannosyl (alpha-1,6-)-glycoprotein beta-1,2-N-acetylglucosaminyltransferase	-1.708693144	1.708693144	down	9.01901E-07	1.07811E-05
SESN1	sestrin 1	-1.709084032	1.709084032	down	1.96808E-05	0.000140648
ERGIC1	endoplasmic reticulum-golgi intermediate compartment 1	-1.709135368	1.709135368	down	2.40006E-07	3.62631E-06
EXOC5	exocyst complex component 5	-1.711039818	1.711039818	down	5.60359E-06	4.92244E-05
AP4B1	adaptor-related protein complex 4, beta 1 subunit	-1.711059585	1.711059585	down	4.50806E-05	0.000284124
MARCH6	membrane associated ring-CH-type finger 6	-1.711348206	1.711348206	down	8.26325E-06	6.83829E-05
SNHG1	small nucleolar RNA host gene 1	-1.711391701	1.711391701	down	2.33101E-09	8.37033E-08
SLC16A1	solute carrier family 16 (monocarboxylate transporter), member 1	-1.712887025	1.712887025	down	4.20135E-07	5.74762E-06
SOX4	SRY-box 4	-1.712989926	1.712989926	down	1.86097E-13	5.45571E-11
CNDP2	CNDP dipeptidase 2 (metallopeptidase M20 family)	-1.713227413	1.713227413	down	1.41734E-08	3.58755E-07
C3orf17	chromosome 3 open reading frame 17	-1.713381797	1.713381797	down	1.96148E-08	4.65589E-07
COQ10B	coenzyme Q10B	-1.713595583	1.713595583	down	7.27201E-08	1.35776E-06
ARL6IP1	ADP ribosylation factor like GTPase 6 interacting protein 1	-1.713837114	1.713837114	down	2.80724E-10	1.57093E-08
TAF1D	TATA-box binding protein associated factor, RNA polymerase I, D	-1.714399499	1.714399499	down	9.53452E-12	1.10906E-09
SNORA8	small nucleolar RNA, H/ACA box 8	-1.714399499	1.714399499	down	9.53452E-12	1.10906E-09
SNORA32	small nucleolar RNA, H/ACA box 32	-1.714399499	1.714399499	down	9.53452E-12	1.10906E-09
SYTL2	synaptotagmin like 2	-1.715905379	1.715905379	down	2.53158E-10	1.46682E-08
SRSF11	serine/arginine-rich splicing factor 11	-1.716052075	1.716052075	down	1.52697E-09	5.91927E-08
B2M	beta-2-microglobulin	-1.71611948	1.71611948	down	2.39233E-13	6.81016E-11
HLA-C	major histocompatibility complex, class I, C	-1.716155166	1.716155166	down	4.89765E-05	0.000304526
NR2C1	nuclear receptor subfamily 2 group C member 1	-1.717099134	1.717099134	down	8.19628E-08	1.49066E-06
DDX42	DEAD (Asp-Glu-Ala-Asp) box helicase 42	-1.717380838	1.717380838	down	4.81822E-09	1.50948E-07
COL4A4	collagen, type IV, alpha 4	-1.718365182	1.718365182	down	6.66023E-13	1.50979E-10
PXYLP1	2-phosphoxylose phosphatase 1	-1.718782111	1.718782111	down	1.13697E-06	1.3079E-05
DHCR24	24-dehydrocholesterol reductase	-1.718905223	1.718905223	down	2.10978E-07	3.26302E-06
OSBPL8	oxysterol binding protein like 8	-1.718917138	1.718917138	down	1.77117E-05	0.000128778
CNN2	calponin 2	-1.719965945	1.719965945	down	8.1565E-09	2.33202E-07
VASH2	vasohibin 2	-1.720109014	1.720109014	down	3.14432E-06	3.04465E-05
NPC1	Niemann-Pick disease, type C1	-1.720260043	1.720260043	down	9.74553E-09	2.6791E-07
C11orf73	chromosome 11 open reading frame 73	-1.720399162	1.720399162	down	1.86369E-05	0.000134459
SLC6A14	solute carrier family 6 (amino acid transporter), member 14	-1.720764897	1.720764897	down	2.38674E-06	2.42463E-05
HLA-F	major histocompatibility complex, class I, F	-1.72136535	1.72136535	down	4.85042E-08	9.76147E-07
MYOCD	myocardin	-1.721611954	1.721611954	down	2.63561E-07	3.91593E-06
GBP2	guanylate binding protein 2, interferon-inducible	-1.721973969	1.721973969	down	2.38349E-11	2.23468E-09

ITGAV	integrin alpha V	-1.723566146	1.723566146	down	1.47099E-06	1.61595E-05
FAM3C	family with sequence similarity 3 member C	-1.723633846	1.723633846	down	4.65673E-11	3.81126E-09
CDC16	cell division cycle 16	-1.72445044	1.72445044	down	3.70717E-07	5.17712E-06
NBPF8	neuroblastoma breakpoint family member 8	-1.72496848	1.72496848	down	1.98338E-06	2.08218E-05
RPS27A	ribosomal protein S27a	-1.725721908	1.725721908	down	7.68187E-09	2.2222E-07
PDZD2	PDZ domain containing 2	-1.726703054	1.726703054	down	3.43419E-07	4.85807E-06
MCM6	minichromosome maintenance complex component 6	-1.729793714	1.729793714	down	1.15502E-06	1.32324E-05
ELF2	E74-like factor 2 (ets domain transcription factor)	-1.730485277	1.730485277	down	8.15991E-05	0.000470504
SERPINB4	serpin peptidase inhibitor, clade B (ovalbumin), member 4	-1.730869153	1.730869153	down	4.46225E-06	4.0651E-05
SERPINB3	serpin peptidase inhibitor, clade B (ovalbumin), member 3	-1.730869153	1.730869153	down	4.46225E-06	4.0651E-05
NXT2	nuclear transport factor 2-like export factor 2	-1.731325117	1.731325117	down	2.44516E-06	2.47693E-05
PDP1	pyruvate dehyrogenase phosphatase catalytic subunit 1	-1.732437533	1.732437533	down	3.9144E-10	2.03522E-08
CCPG1	cell cycle progression 1	-1.732901917	1.732901917	down	1.68306E-06	1.8134E-05
DYX1C1-	DYX1C1-CCPG1 readthrough (NMD candidate)	-1.732901917	1.732901917	down	1.68306E-06	1.8134E-05
ATAD1	ATPase family, AAA domain containing 1	-1.733470558	1.733470558	down	4.48309E-10	2.25498E-08
SUCO	SUN domain containing ossification factor	-1.734644469	1.734644469	down	2.87982E-07	4.20873E-06
PTPRJ	protein tyrosine phosphatase, receptor type, J	-1.734792767	1.734792767	down	8.3521E-08	1.51283E-06
TSPAN3	tetraspanin 3	-1.734957112	1.734957112	down	2.27144E-12	3.86282E-10
IBTK	inhibitor of Bruton tyrosine kinase	-1.735634698	1.735634698	down	6.70386E-07	8.48162E-06
GBP3	guanylate binding protein 3	-1.735923454	1.735923454	down	2.12001E-11	2.0567E-09
SRI	sorcin	-1.736011695	1.736011695	down	3.83646E-08	8.05513E-07
QKI	QKI, KH domain containing, RNA binding	-1.736416857	1.736416857	down	4.41312E-08	9.04356E-07
KLHL7	kelch like family member 7	-1.736533208	1.736533208	down	6.76124E-05	0.000401221
CLK4	CDC like kinase 4	-1.737412112	1.737412112	down	7.45934E-06	6.28151E-05
HSPA8	heat shock protein family A (Hsp70) member 8	-1.737540573	1.737540573	down	5.21917E-06	4.63449E-05
MYBL1	v-myb avian myeloblastosis viral oncogene homolog-like 1	-1.74001129	1.74001129	down	1.3065E-06	1.4639E-05
MBD2	methyl-CpG binding domain protein 2	-1.740075615	1.740075615	down	1.66047E-08	4.06925E-07
CSRP1	cysteine and glycine rich protein 1	-1.740340984	1.740340984	down	2.17354E-06	2.25053E-05
MBNL2	muscleblind-like splicing regulator 2	-1.740425428	1.740425428	down	2.46788E-07	3.70739E-06
BTG2	BTG family member 2	-1.744120841	1.744120841	down	4.37973E-10	2.21718E-08
KIAA0895	KIAA0895	-1.744580295	1.744580295	down	1.86286E-07	2.94253E-06
B4GALT4	UDP-Gal:betaGlcNAc beta 1,4- galactosyltransferase, polypeptide 4	-1.747020657	1.747020657	down	2.70373E-09	9.47486E-08
MBOAT7	membrane bound O-acyltransferase domain containing 7	-1.747921022	1.747921022	down	4.80774E-12	6.76944E-10
SDCBP	syndecan binding protein	-1.748139118	1.748139118	down	3.37897E-09	1.13453E-07
UBA2	ubiquitin-like modifier activating enzyme 2	-1.751713295	1.751713295	down	1.11365E-08	2.99443E-07
UHRF1	ubiquitin-like with PHD and ring finger domains 1	-1.752292157	1.752292157	down	1.33707E-08	3.43754E-07
WTAP	Wilms tumor 1 associated protein	-1.755278557	1.755278557	down	1.1716E-08	3.10203E-07
GRAMD3	GRAM domain containing 3	-1.756783813	1.756783813	down	2.94675E-05	0.000197543
TRIOBP	TRIO and F-actin binding protein	-1.757258784	1.757258784	down	3.93111E-09	1.27839E-07
TMBIM6	transmembrane BAX inhibitor motif containing 6	-1.757766374	1.757766374	down	1.2339E-11	1.35398E-09
CLDN1	claudin 1	-1.758578822	1.758578822	down	6.18705E-11	4.77509E-09
GDF15	growth differentiation factor 15	-1.759436362	1.759436362	down	4.52652E-12	6.44275E-10
LRP11	LDL receptor related protein 11	-1.760025909	1.760025909	down	7.45667E-07	9.264E-06
PELI1	pellino E3 ubiquitin protein ligase 1	-1.761152697	1.761152697	down	8.27571E-07	1.00713E-05
EMP1	epithelial membrane protein 1	-1.761571866	1.761571866	down	2.10917E-10	1.25731E-08
CCDC80	coiled-coil domain containing 80	-1.762064417	1.762064417	down	4.79585E-13	1.16296E-10
GSAP	gamma-secretase activating protein	-1.765336652	1.765336652	down	4.45788E-09	1.418E-07
ALCAM	activated leukocyte cell adhesion molecule	-1.767630423	1.767630423	down	1.4059E-07	2.33141E-06
LETMD1	LETM1 domain containing 1	-1.767703938	1.767703938	down	4.28611E-07	5.84841E-06
CYBRD1	cytochrome b reductase 1	-1.769918995	1.769918995	down	2.57657E-08	5.83389E-07
STARD7	StAR related lipid transfer domain containing 7	-1.769976248	1.769976248	down	6.58388E-11	4.97556E-09
ARHGAP21	Rho GTPase activating protein 21	-1.770364794	1.770364794	down	1.47919E-07	2.42555E-06
TRIM22	tripartite motif containing 22	-1.771371321	1.771371321	down	4.84699E-08	9.75957E-07
RPL3	ribosomal protein L3	-1.771735612	1.771735612	down	5.01217E-08	9.99541E-07
SPRED1	sprouty-related, EVH1 domain containing 1	-1.772882185	1.772882185	down	0.00011114	0.000611533
EFEMP1	EGF containing fibulin-like extracellular matrix protein 1	-1.775374442	1.775374442	down	2.76031E-10	1.55562E-08
LOC105369242	E3 ubiquitin-protein ligase HERC2-like	-1.775714939	1.775714939	down	4.46335E-08	9.10847E-07
HERC2P2	hect domain and RLD 2 pseudogene 2	-1.775714939	1.775714939	down	4.46335E-08	9.10847E-07
HERC2P9	hect domain and RLD 2 pseudogene 9	-1.775714939	1.775714939	down	4.46335E-08	9.10847E-07
HERC2	HECT and RLD domain containing E3 ubiquitin protein ligase 2	-1.775714939	1.775714939	down	4.46335E-08	9.10847E-07
TCEA1	transcription elongation factor A (SII), 1	-1.776104745	1.776104745	down	4.64421E-07	6.24378E-06
C9orf16	chromosome 9 open reading frame 16	-1.776285316	1.776285316	down	4.65687E-11	3.81126E-09
EHF	ets homologous factor	-1.777048841	1.777048841	down	2.74605E-07	4.05092E-06
TNPO1	transportin 1	-1.779341385	1.779341385	down	5.9255E-08	1.14162E-06
EXT2	exostosin glycosyltransferase 2	-1.780324222	1.780324222	down	2.12089E-09	7.73602E-08
DPEP2	dipeptidase 2	-1.782250345	1.782250345	down	1.47106E-07	2.41695E-06
CAB39	calcium binding protein 39	-1.783337795	1.783337795	down	4.04526E-05	0.000259259
MDK	midkine (neurite growth-promoting factor 2)	-1.784368189	1.784368189	down	9.81484E-14	3.57006E-11
SERP1	stress-associated endoplasmic reticulum protein 1	-1.784619695	1.784619695	down	1.29718E-09	5.23188E-08
ANXA5	annexin A5	-1.784904229	1.784904229	down	6.40476E-09	1.90899E-07
NAV1	neuron navigator 1	-1.785048575	1.785048575	down	8.16345E-09	2.33224E-07
NEBL	nebulette	-1.785230055	1.785230055	down	5.08192E-08	1.00929E-06
ZBED6CL	ZBED6 C-terminal like	-1.787314273	1.787314273	down	2.28845E-11	2.18177E-09
FOLR1	folate receptor 1 (adult)	-1.788301513	1.788301513	down	3.03615E-13	8.11375E-11
PARP9	poly(ADP-ribose) polymerase family member 9	-1.789314104	1.789314104	down	1.32844E-09	5.34151E-08
ERP27	endoplasmic reticulum protein 27	-1.791204428	1.791204428	down	1.90055E-13	5.53046E-11
IFIH1	interferon induced, with helicase C domain 1	-1.791709404	1.791709404	down	3.50259E-12	5.29214E-10
CEP170	centrosomal protein 170kDa	-1.792458851	1.792458851	down	1.98599E-07	3.09963E-06
CCDC71L	coiled-coil domain containing 71-like	-1.794327623	1.794327623	down	3.70113E-07	5.17237E-06
ENOPH1	enolase-phosphatase 1	-1.795119641	1.795119641	down	2.8714E-08	6.36211E-07
CD164	CD164 molecule, sialomucin	-1.795127936	1.795127936	down	1.42944E-11	1.52179E-09
RAB5A	RAB5A, member RAS oncogene family	-1.795729443	1.795729443	down	5.31219E-07	6.97474E-06
APOL1	apolipoprotein L1	-1.796924757	1.796924757	down	4.29189E-10	2.17833E-08
ECT2	epithelial cell transforming 2	-1.797867458	1.797867458	down	1.49744E-09	5.83585E-08
MOSPD1	motile sperm domain containing 1	-1.79876078	1.79876078	down	1.50582E-07	2.45251E-06
DAPP1	dual adaptor of phosphotyrosine and 3-phosphoinositides	-1.799791769	1.799791769	down	2.01576E-07	3.1411E-06
BTN3A2	butyrophilin, subfamily 3, member A2	-1.800669405	1.800669405	down	3.97216E-12	5.85388E-10
DNAJB6	DnaJ heat shock protein family (Hsp40) member B6	-1.801172888	1.801172888	down	6.68752E-12	8.60232E-10
PRKCH	protein kinase C, eta	-1.803713249	1.803713249	down	7.5501E-07	9.3505E-06
HLTF	helicase-like transcription factor	-1.804930559	1.804930559	down	1.10091E-07	1.90353E-06
PMEPA1	prostate transmembrane protein, androgen induced 1	-1.805293409	1.805293409	down	1.92183E-12	3.32833E-10
UACA	uveal autoantigen with coiled-coil domains and ankyrin repeats	-1.805318436	1.805318436	down	4.43871E-08	9.08652E-07
REEP3	receptor accessory protein 3	-1.805998465	1.805998465	down	2.15763E-08	5.0273E-07
CAMK1D	calcium/calmodulin dependent protein kinase ID	-1.806382398	1.806382398	down	8.31751E-08	1.50782E-06
PHTF2	putative homeodomain transcription factor 2	-1.80720479	1.80720479	down	3.47625E-06	3.29698E-05
ORC3	origin recognition complex subunit 3	-1.810029654	1.810029654	down	5.79376E-08	1.12076E-06
SNORA25	small nucleolar RNA, H/ACA box 25	-1.812025595	1.812025595	down	7.33808E-12	9.23762E-10
MX2	MX dynamin-like GTPase 2	-1.815432555	1.815432555	down	1.15989E-07	1.99235E-06

GCNT3	glucosaminyl (N-acetyl) transferase 3, mucin type	-1.81626746	1.81626746	down	3.64054E-08	7.72219E-07
ABLIM3	actin binding LIM protein family member 3	-1.816288442	1.816288442	down	6.08886E-08	1.16851E-06
IL1R1	interleukin 1 receptor, type I	-1.817253898	1.817253898	down	9.69539E-12	1.12022E-09
SSFA2	sperm specific antigen 2	-1.818929971	1.818929971	down	4.20245E-09	1.3543E-07
RPL15	ribosomal protein L15	-1.819014025	1.819014025	down	1.00321E-10	6.97521E-09
MORC4	MORC family CW-type zinc finger 4	-1.819068663	1.819068663	down	6.3164E-07	8.10364E-06
ATP1B1	ATPase, Na+/K+ transporting, beta 1 polypeptide	-1.819842168	1.819842168	down	8.96202E-13	1.90305E-10
PKD1L2	polycystic kidney disease 1-like 2 (gene/pseudogene)	-1.81998934	1.81998934	down	6.89504E-07	8.68156E-06
SLC16A12	solute carrier family 16, member 12	-1.821806836	1.821806836	down	9.48908E-07	1.12314E-05
HNRNPD	heterogeneous nuclear ribonucleoprotein D	-1.822568873	1.822568873	down	9.85681E-06	7.87182E-05
KCNQ1OT1	KCNQ1 opposite strand/antisense transcript 1 (non-protein coding)	-1.824667172	1.824667172	down	2.19037E-06	2.26194E-05
NT5C2	5'-nucleotidase, cytosolic II	-1.824915925	1.824915925	down	2.86892E-08	6.36019E-07
FUBP1	far upstream element (FUSE) binding protein 1	-1.824937008	1.824937008	down	3.4365E-08	7.35292E-07
ENOX1	ecto-NOX disulfide-thiol exchanger 1	-1.825797376	1.825797376	down	4.75312E-07	6.35973E-06
PPM1K	protein phosphatase, Mg2+/Mn2+ dependent 1K	-1.825869092	1.825869092	down	1.78806E-08	4.32793E-07
STK4	serine/threonine kinase 4	-1.826797432	1.826797432	down	2.85902E-11	2.576E-09
RRM1	ribonucleotide reductase M1	-1.826978935	1.826978935	down	2.60519E-09	9.18691E-08
CLIC4	chloride intracellular channel 4	-1.828862569	1.828862569	down	1.05401E-07	1.83861E-06
IRF2BP2	interferon regulatory factor 2 binding protein 2	-1.830181422	1.830181422	down	3.88884E-10	2.03108E-08
IDI1	isopentenyl-diphosphate delta isomerase 1	-1.83162818	1.83162818	down	5.51076E-11	4.36461E-09
NEK2	NIMA-related kinase 2	-1.835076237	1.835076237	down	2.03093E-08	4.77457E-07
MAFB	v-maf avian musculoaponeurotic fibrosarcoma oncogene homolog B	-1.835619028	1.835619028	down	1.59296E-10	1.00446E-08
HIST1H2BG	histone cluster 1, H2bg	-1.836471703	1.836471703	down	4.99289E-08	9.98172E-07
TNNC1	troponin C type 1 (slow)	-1.837596482	1.837596482	down	1.2975E-06	1.45673E-05
PSAT1	phosphoserine aminotransferase 1	-1.837846998	1.837846998	down	1.34342E-10	8.79581E-09
CD22	CD22 molecule	-1.838666723	1.838666723	down	2.97232E-09	1.02335E-07
MTMR11	myotubularin related protein 11	-1.839580317	1.839580317	down	1.39556E-08	3.54155E-07
STOM	stomatin	-1.839920375	1.839920375	down	1.14492E-08	3.0493E-07
P2RX4	purinergic receptor P2X, ligand gated ion channel, 4	-1.842098236	1.842098236	down	6.83381E-08	1.28511E-06
PTP4A1	protein tyrosine phosphatase type IVA, member 1	-1.84618441	1.84618441	down	2.72361E-10	1.54393E-08
H2AFJ	H2A histone family member J	-1.846931039	1.846931039	down	3.33469E-08	7.16238E-07
CRIM1	cysteine rich transmembrane BMP regulator 1 (chordin-like)	-1.848378226	1.848378226	down	2.91688E-08	6.43745E-07
SCML1	sex comb on midleg-like 1 (Drosophila)	-1.852294294	1.852294294	down	8.81885E-06	7.19053E-05
GALNT12	polypeptide N-acetylgalactosaminyltransferase 12	-1.856411663	1.856411663	down	2.09754E-10	1.25609E-08
PATL2	protein associated with topoisomerase II homolog 2 (yeast)	-1.856969346	1.856969346	down	5.9996E-09	1.80231E-07
FZD6	frizzled class receptor 6	-1.857106647	1.857106647	down	5.75154E-09	1.73403E-07
EIF4H	eukaryotic translation initiation factor 4H	-1.857484278	1.857484278	down	1.81489E-09	6.86199E-08
BTAF1	B-TFIID TATA-box binding protein associated factor 1	-1.857866279	1.857866279	down	2.47162E-07	3.71018E-06
PDE9A	phosphodiesterase 9A	-1.858420105	1.858420105	down	1.14687E-06	1.3162E-05
CSE1L	CSE1 chromosome segregation 1-like (yeast)	-1.85876794	1.85876794	down	5.18039E-13	1.24089E-10
HIST1H2BK	histone cluster 1, H2bk	-1.860164231	1.860164231	down	5.93987E-06	5.17962E-05
F3	coagulation factor III (thromboplastin, tissue factor)	-1.862284299	1.862284299	down	7.18808E-12	9.10892E-10
DUSP1	dual specificity phosphatase 1	-1.866014245	1.866014245	down	1.49404E-09	5.82837E-08
PADI2	peptidyl arginine deiminase, type II	-1.866333317	1.866333317	down	3.3353E-07	4.73352E-06
IFIT2	interferon induced protein with tetratricopeptide repeats 2	-1.867226145	1.867226145	down	2.2385E-09	8.08246E-08
TM9SF3	transmembrane 9 superfamily member 3	-1.869198779	1.869198779	down	5.98141E-11	4.66218E-09
PGRMC2	progesterone receptor membrane component 2	-1.870646126	1.870646126	down	1.6061E-10	1.00951E-08
HSD17B12	hydroxysteroid (17-beta) dehydrogenase 12	-1.871713992	1.871713992	down	2.07919E-11	2.05223E-09
MMD	monocyte to macrophage differentiation-associated	-1.873531196	1.873531196	down	1.27648E-13	4.36045E-11
HNRNPDL	heterogeneous nuclear ribonucleoprotein D like	-1.873786611	1.873786611	down	9.05957E-07	1.08095E-05
HSPA5	heat shock protein family A (Hsp70) member 5	-1.874765303	1.874765303	down	2.66431E-13	7.29356E-11
RND3	Rho family GTPase 3	-1.875822519	1.875822519	down	2.17199E-11	2.09642E-09
ACTN1	actinin, alpha 1	-1.877431148	1.877431148	down	2.31677E-12	3.89576E-10
COL17A1	collagen, type XVII, alpha 1	-1.879866224	1.879866224	down	1.68311E-11	1.73164E-09
FHL2	four and a half LIM domains 2	-1.88049178	1.88049178	down	2.53733E-13	7.07377E-11
MAP3K2	mitogen-activated protein kinase kinase kinase 2	-1.883618329	1.883618329	down	5.66891E-07	7.37165E-06
BPIFB1	BPI fold containing family B, member 1	-1.884057941	1.884057941	down	2.75723E-15	1.69242E-12
ADGRF4	adhesion G protein-coupled receptor F4	-1.884737147	1.884737147	down	1.94666E-07	3.04309E-06
BMP2	bone morphogenetic protein 2	-1.888001617	1.888001617	down	1.92924E-07	3.02668E-06
STRA6	stimulated by retinoic acid 6	-1.888948454	1.888948454	down	1.8121E-10	1.12105E-08
TMED2	transmembrane p24 trafficking protein 2	-1.888961547	1.888961547	down	4.51779E-10	2.26686E-08
TIA1	TIA1 cytotoxic granule-associated RNA binding protein	-1.889537739	1.889537739	down	4.63873E-10	2.31842E-08
SRSF5	serine/arginine-rich splicing factor 5	-1.89021892	1.89021892	down	5.86291E-08	1.13179E-06
INHBA	inhibin beta A	-1.891599501	1.891599501	down	7.34075E-08	1.36677E-06
VEGFA	vascular endothelial growth factor A	-1.895357509	1.895357509	down	3.53935E-09	1.18131E-07
EFNA1	ephrin-A1	-1.896847024	1.896847024	down	4.95854E-09	1.54419E-07
CLIC5	chloride intracellular channel 5	-1.897009188	1.897009188	down	2.06664E-07	3.20767E-06
ANLN	anillin actin binding protein	-1.897083701	1.897083701	down	2.13508E-10	1.26699E-08
HMGCR	3-hydroxy-3-methylglutaryl-CoA reductase	-1.900251008	1.900251008	down	4.76752E-09	1.4983E-07
CDK5RAP3	CDK5 regulatory subunit associated protein 3	-1.900694502	1.900694502	down	9.9849E-12	1.14358E-09
GOLPH3	golgi phosphoprotein 3 (coat-protein)	-1.903705086	1.903705086	down	8.05947E-12	9.86319E-10
CTSB	cathepsin B	-1.906918695	1.906918695	down	1.64254E-12	2.99194E-10
DMTF1	cyclin D binding myb-like transcription factor 1	-1.911501943	1.911501943	down	2.40717E-09	8.58105E-08
CD74	CD74 molecule, major histocompatibility complex, class II invariant chain	-1.911727198	1.911727198	down	2.62716E-06	2.62743E-05
CTHRC1	collagen triple helix repeat containing 1	-1.912305916	1.912305916	down	8.35284E-08	1.51283E-06
SUN1	Sad1 and UNC84 domain containing 1	-1.914573881	1.914573881	down	1.21095E-07	2.0728E-06
RHOV	ras homolog family member V	-1.918860723	1.918860723	down	8.22741E-10	3.64771E-08
AKAP12	A-kinase anchoring protein 12	-1.920413079	1.920413079	down	6.5069E-08	1.23486E-06
NCKAP1	NCK-associated protein 1	-1.921638108	1.921638108	down	9.18998E-11	6.51659E-09
MMP13	matrix metallopeptidase 13	-1.92476636	1.92476636	down	1.56928E-12	2.89426E-10
PIM1	Pim-1 proto-oncogene, serine/threonine kinase	-1.924770807	1.924770807	down	7.36018E-12	9.23762E-10
GBP1	guanylate binding protein 1, interferon-inducible	-1.924966493	1.924966493	down	1.52969E-10	9.73942E-09
SNORA44	small nucleolar RNA, H/ACA box 44	-1.925446895	1.925446895	down	1.59145E-07	2.5749E-06
SNHG12	small nucleolar RNA host gene 12	-1.925446895	1.925446895	down	1.59145E-07	2.5749E-06
DLG1	discs, large homolog 1 (Drosophila)	-1.925673794	1.925673794	down	5.25397E-07	6.91447E-06
IRF1	interferon regulatory factor 1	-1.927734899	1.927734899	down	1.12194E-10	7.58592E-09
NHLRC3	NHL repeat containing 3	-1.933341732	1.933341732	down	2.11409E-08	4.94346E-07
CDH1	cadherin 1, type 1	-1.934512432	1.934512432	down	1.70961E-09	6.50777E-08
ASAH1	N-acylsphingosine amidohydrolase (acid ceramidase) 1	-1.935165114	1.935165114	down	2.22177E-10	1.30855E-08
SERPING1	serpin peptidase inhibitor, clade G (C1 inhibitor), member 1	-1.94030036	1.94030036	down	2.88028E-08	6.37459E-07
MGAM	maltase-glucoamylase	-1.940784589	1.940784589	down	1.71972E-05	0.000125712
P4HB	prolyl 4-hydroxylase, beta polypeptide	-1.942664364	1.942664364	down	9.23853E-13	1.9282E-10
OLR1	oxidized low density lipoprotein (lectin-like) receptor 1	-1.944644805	1.944644805	down	3.91897E-10	2.03522E-08
CNBP	CCHC-type zinc finger, nucleic acid binding protein	-1.945161578	1.945161578	down	4.27177E-12	6.19233E-10
LINC00842	long intergenic non-protein coding RNA 842	-1.946681237	1.946681237	down	1.15105E-11	1.28825E-09
ANXA8L1	annexin A8-like 1	-1.946681237	1.946681237	down	1.15105E-11	1.28825E-09
CPEB4	cytoplasmic polyadenylation element binding protein 4	-1.951562951	1.951562951	down	1.09087E-06	1.26561E-05
CAPZA1	capping protein (actin filament) muscle Z-line, alpha 1	-1.951815475	1.951815475	down	3.71831E-11	3.21033E-09

FMO4	flavin containing monooxygenase 4	-1.951833514	1.951833514	down	5.84034E-10	2.78099E-08
TOP1	topoisomerase (DNA) I	-1.951833514	1.951833514	down	5.84034E-10	2.78099E-08
TOP1P1	topoisomerase (DNA) I pseudogene 1	-1.951833514	1.951833514	down	5.84034E-10	2.78099E-08
MEST	mesoderm specific transcript	-1.955182576	1.955182576	down	8.14608E-08	1.48359E-06
GBP4	guanylate binding protein 4	-1.957845154	1.957845154	down	1.58285E-11	1.65965E-09
SLC25A27	solute carrier family 25, member 27	-1.958433308	1.958433308	down	6.73626E-08	1.2692E-06
ANKRD13A	ankyrin repeat domain 13A	-1.958949219	1.958949219	down	5.34645E-08	1.05046E-06
LMAN2L	lectin, mannose-binding 2-like	-1.959605619	1.959605619	down	8.11611E-10	3.62439E-08
TIMP3	TIMP metallopeptidase inhibitor 3	-1.960121839	1.960121839	down	5.0867E-12	7.06098E-10
ADAM10	ADAM metallopeptidase domain 10	-1.965809243	1.965809243	down	5.11491E-09	1.57723E-07
LAMC2	laminin subunit gamma 2	-1.970128867	1.970128867	down	4.75418E-10	2.36442E-08
DLST	dihydrolipoamide S-succinyltransferase (E2 component of 2-oxo-glutarate complex)	-1.97261125	1.97261125	down	2.43376E-08	5.54891E-07
DRAM1	DNA damage regulated autophagy modulator 1	-1.973951669	1.973951669	down	2.09247E-11	2.0567E-09
SMAD3	SMAD family member 3	-1.983653443	1.983653443	down	4.12082E-12	6.01794E-10
ANXA3	annexin A3	-1.985877548	1.985877548	down	1.24308E-10	8.22812E-09
DDIT3	DNA damage inducible transcript 3	-1.987378505	1.987378505	down	6.58317E-11	4.97556E-09
RNF114	ring finger protein 114	-1.994697534	1.994697534	down	4.76613E-13	1.16296E-10
BCAS1	breast carcinoma amplified sequence 1	-1.995347471	1.995347471	down	8.21666E-11	5.98856E-09
IGF2	insulin like growth factor 2	-2.003913173	2.003913173	down	5.67594E-11	4.48639E-09
INS-IGF2	INS-IGF2 readthrough	-2.003913173	2.003913173	down	5.67594E-11	4.48639E-09
LIPH	lipase, member H	-2.005348994	2.005348994	down	1.0975E-11	1.23536E-09
CEACAM1	carcinoembryonic antigen-related cell adhesion molecule 1 (biliary glycoprotein)	-2.006656022	2.006656022	down	6.92786E-09	2.03252E-07
RAB6A	RAB6A, member RAS oncogene family	-2.007959262	2.007959262	down	1.26375E-09	5.12334E-08
PRNP	prion protein	-2.009537268	2.009537268	down	5.16509E-09	1.58892E-07
WSB1	WD repeat and SOCS box containing 1	-2.010089863	2.010089863	down	1.33378E-08	3.43367E-07
NFE2L3	nuclear factor, erythroid 2 like 3	-2.010103796	2.010103796	down	9.87494E-09	2.70742E-07
FILIP1L	filamin A interacting protein 1-like	-2.012027467	2.012027467	down	1.05923E-06	1.23474E-05
PRR5L	proline rich 5 like	-2.012134391	2.012134391	down	1.59194E-06	1.73139E-05
MSH6	mutS homolog 6	-2.012580747	2.012580747	down	1.90802E-08	4.55374E-07
OXR1	oxidation resistance 1	-2.021911709	2.021911709	down	1.39865E-07	2.32433E-06
TWF1	twinfilin actin binding protein 1	-2.024604346	2.024604346	down	3.19726E-08	6.92398E-07
SLC35A1	solute carrier family 35 (CMP-sialic acid transporter), member A1	-2.024768077	2.024768077	down	4.39191E-08	9.00948E-07
TRAFD1	TRAF-type zinc finger domain containing 1	-2.02640143	2.02640143	down	9.91838E-11	6.92067E-09
MCCC1	methylcrotonoyl-CoA carboxylase 1	-2.03167551	2.03167551	down	6.4393E-07	8.22104E-06
RFC3	replication factor C subunit 3	-2.034512767	2.034512767	down	7.24128E-09	2.1056E-07
SYTL5	synaptotagmin like 5	-2.035194486	2.035194486	down	1.25385E-08	3.26633E-07
CD47	CD47 molecule	-2.039364989	2.039364989	down	1.06242E-12	2.15134E-10
TRPV6	transient receptor potential cation channel, subfamily V, member 6	-2.040029479	2.040029479	down	7.60861E-11	5.62894E-09
CCL20	chemokine (C-C motif) ligand 20	-2.040222741	2.040222741	down	2.68288E-06	2.67159E-05
PRSS23	protease, serine 23	-2.042359262	2.042359262	down	1.83884E-15	1.24547E-12
MSANTD2	Myb/SANT-like DNA-binding domain containing 2	-2.042524429	2.042524429	down	4.73103E-08	9.58266E-07
HIST2H2AA3	histone cluster 2, H2aa3	-2.045972376	2.045972376	down	3.28794E-14	1.38885E-11
HIST2H2AA4	histone cluster 2, H2aa4	-2.045972376	2.045972376	down	3.28794E-14	1.38885E-11
VTCN1	V-set domain containing T cell activation inhibitor 1	-2.048029742	2.048029742	down	3.84264E-07	5.32467E-06
ACSS2	acyl-CoA synthetase short-chain family member 2	-2.052193356	2.052193356	down	3.12571E-11	2.77806E-09
C10orf10	chromosome 10 open reading frame 10	-2.052492097	2.052492097	down	9.19502E-13	1.9282E-10
RND1	Rho family GTPase 1	-2.055059265	2.055059265	down	1.28418E-06	1.44467E-05
TOMM20	translocase of outer mitochondrial membrane 20 homolog (yeast)	-2.060055682	2.060055682	down	3.30116E-06	3.15914E-05
PTPN3	protein tyrosine phosphatase, non-receptor type 3	-2.060712631	2.060712631	down	6.3606E-07	8.15148E-06
NCOA7	nuclear receptor coactivator 7	-2.061045946	2.061045946	down	2.43187E-11	2.2692E-09
PCNA	proliferating cell nuclear antigen	-2.061307874	2.061307874	down	1.0497E-06	1.22436E-05
RPL4	ribosomal protein L4	-2.066691905	2.066691905	down	1.90116E-06	2.00983E-05
PTPN12	protein tyrosine phosphatase, non-receptor type 12	-2.072362908	2.072362908	down	6.42142E-13	1.46662E-10
MSMO1	methylsterol monooxygenase 1	-2.073397411	2.073397411	down	3.18234E-10	1.7196E-08
CKLF	chemokine-like factor	-2.073862148	2.073862148	down	2.14134E-07	3.30403E-06
CMTM1	CKLF-like MARVEL transmembrane domain containing 1	-2.073862148	2.073862148	down	2.14134E-07	3.30403E-06
CAPN2	calpain 2, (m/II) large subunit	-2.074801522	2.074801522	down	3.58302E-11	3.12097E-09
HRASLS2	HRAS-like suppressor 2	-2.076839892	2.076839892	down	4.98012E-15	2.79484E-12
H1F0	H1 histone family member 0	-2.077410994	2.077410994	down	1.32076E-12	2.49495E-10
SNORA63	small nucleolar RNA, H/ACA box 63	-2.090005395	2.090005395	down	7.02838E-10	3.22619E-08
LBH	limb bud and heart development	-2.108192588	2.108192588	down	2.7456E-08	6.14577E-07
BIRC2	baculoviral IAP repeat containing 2	-2.11206368	2.11206368	down	7.77339E-15	4.0677E-12
DOCK9	dedicator of cytokinesis 9	-2.118123436	2.118123436	down	2.94931E-08	6.48352E-07
DPP7	dipeptidyl-peptidase 7	-2.121017709	2.121017709	down	1.66895E-10	1.04234E-08
SLC25A37	solute carrier family 25 (mitochondrial iron transporter), member 37	-2.123253557	2.123253557	down	5.28805E-10	2.56148E-08
ANP32A	acidic nuclear phosphoprotein 32 family member A	-2.124971266	2.124971266	down	2.59353E-10	1.4983E-08
ARFGAP3	ADP ribosylation factor GTPase activating protein 3	-2.126120453	2.126120453	down	6.70246E-10	3.09764E-08
HLA-E	major histocompatibility complex, class I, E	-2.128553473	2.128553473	down	3.08286E-15	1.83496E-12
RNF145	ring finger protein 145	-2.138056533	2.138056533	down	1.11433E-09	4.64708E-08
TSPAN2	tetraspanin 2	-2.141759766	2.141759766	down	3.85017E-13	9.89693E-11
PTPRE	protein tyrosine phosphatase, receptor type, E	-2.144879601	2.144879601	down	3.04308E-10	1.65803E-08
TXNRD1	thioredoxin reductase 1	-2.147279513	2.147279513	down	9.39123E-11	6.6473E-09
TNFRSF21	tumor necrosis factor receptor superfamily member 21	-2.149081209	2.149081209	down	1.15817E-11	1.29254E-09
ANO1	anoctamin 1, calcium activated chloride channel	-2.154410749	2.154410749	down	5.79176E-08	1.12076E-06
MLLT10	myeloid/lymphoid or mixed-lineage leukemia; translocated to, 10	-2.16274464	2.16274464	down	1.40794E-10	9.06768E-09
OSMR	oncostatin M receptor	-2.167852601	2.167852601	down	5.0167E-10	2.45425E-08
PPT1	palmitoyl-protein thioesterase 1	-2.169821954	2.169821954	down	8.89257E-14	3.29562E-11
BTN3A3	butyrophilin, subfamily 3, member A3	-2.170107734	2.170107734	down	1.19722E-09	4.89914E-08
FZD10	frizzled class receptor 10	-2.185681724	2.185681724	down	1.02323E-08	2.78756E-07
SYNE2	spectrin repeat containing, nuclear envelope 2	-2.191350109	2.191350109	down	1.0087E-09	4.30249E-08
TNFAIP3	TNF alpha induced protein 3	-2.203540378	2.203540378	down	3.87987E-12	5.75158E-10
NBPF1	neuroblastoma breakpoint family member 1	-2.210301741	2.210301741	down	1.49203E-09	5.82837E-08
NBPF11	neuroblastoma breakpoint family member 11	-2.210301741	2.210301741	down	1.49203E-09	5.82837E-08
NBPF9	neuroblastoma breakpoint family member 9	-2.210301741	2.210301741	down	1.49203E-09	5.82837E-08
NBPF10	neuroblastoma breakpoint family member 10	-2.210301741	2.210301741	down	1.49203E-09	5.82837E-08
NBPF26	neuroblastoma breakpoint family member 26	-2.210301741	2.210301741	down	1.49203E-09	5.82837E-08
LOC102724250	neuroblastoma breakpoint family member 1	-2.210301741	2.210301741	down	1.49203E-09	5.82837E-08
NBPF20	neuroblastoma breakpoint family, member 20	-2.210301741	2.210301741	down	1.49203E-09	5.82837E-08
NBPF12	neuroblastoma breakpoint family member 12	-2.210301741	2.210301741	down	1.49203E-09	5.82837E-08
NBPF14	neuroblastoma breakpoint family member 14	-2.210301741	2.210301741	down	1.49203E-09	5.82837E-08
NBPF19	neuroblastoma breakpoint family member 19	-2.210301741	2.210301741	down	1.49203E-09	5.82837E-08
LOC100996763	notch homolog 2 N-terminal-like protein	-2.210301741	2.210301741	down	1.49203E-09	5.82837E-08
NBPF25P	neuroblastoma breakpoint family member 25, pseudogene	-2.210301741	2.210301741	down	1.49203E-09	5.82837E-08
SPTLC1	serine palmitoyltransferase, long chain base subunit 1	-2.218139346	2.218139346	down	4.22936E-08	8.73994E-07
PPFIA1	protein tyrosine phosphatase, receptor type, f polypeptide (PTPRF), interacting protein (liprin), alpha 1	-2.220826467	2.220826467	down	8.57717E-08	1.54633E-06

HIF1A	hypoxia inducible factor 1, alpha subunit (basic helix-loop-helix transcription factor)	-2.222412574	2.222412574	down	9.0796E-12	1.07112E-09
C1S	complement component 1, s subcomponent	-2.232938216	2.232938216	down	2.5802E-15	1.6089E-12
COL4A3	collagen, type IV, alpha 3 (Goodpasture antigen)	-2.233768999	2.233768999	down	1.82461E-11	1.8379E-09
FBLN1	fibulin 1	-2.240762897	2.240762897	down	5.0116E-12	7.00626E-10
HMGB1	high mobility group box 1	-2.240819847	2.240819847	down	9.08088E-11	6.46256E-09
TRIM31	tripartite motif containing 31	-2.241783049	2.241783049	down	1.51257E-15	1.04245E-12
N4BP2L1	NEDD4 binding protein 2-like 1	-2.243161251	2.243161251	down	7.80944E-10	3.51014E-08
DMXL2	Dmx-like 2	-2.245043398	2.245043398	down	1.6088E-07	2.59975E-06
ITGB1	integrin beta 1	-2.247716371	2.247716371	down	7.18139E-16	5.64227E-13
TXNIP	thioredoxin interacting protein	-2.249716692	2.249716692	down	1.73534E-14	8.11561E-12
ADGRA2	adhesion G protein-coupled receptor A2	-2.25680745	2.25680745	down	7.70366E-11	5.68855E-09
TNFSF15	tumor necrosis factor superfamily member 15	-2.267794635	2.267794635	down	4.81322E-10	2.38139E-08
ELOVL5	ELOVL fatty acid elongase 5	-2.269010576	2.269010576	down	3.01445E-10	1.647E-08
TMEM2	transmembrane protein 2	-2.271307965	2.271307965	down	3.31515E-12	5.10716E-10
CLEC2D	C-type lectin domain family 2, member D	-2.27314545	2.27314545	down	2.87891E-09	1.00084E-07
BPGM	bisphosphoglycerate mutase	-2.277282599	2.277282599	down	9.0848E-14	3.3354E-11
TNFAIP2	TNF alpha induced protein 2	-2.279625233	2.279625233	down	1.77168E-14	8.18807E-12
IDO1	indoleamine 2,3-dioxygenase 1	-2.280842246	2.280842246	down	4.95611E-14	1.94696E-11
MET	MET proto-oncogene, receptor tyrosine kinase	-2.28613405	2.28613405	down	2.12859E-12	3.63563E-10
PPM1B	protein phosphatase, Mg2+/Mn2+ dependent 1B	-2.289104513	2.289104513	down	6.89865E-09	2.02697E-07
LOC100505984	uncharacterized LOC100505984	-2.291506958	2.291506958	down	3.1475E-09	1.07053E-07
ITGB6	integrin beta 6	-2.291506958	2.291506958	down	3.1475E-09	1.07053E-07
ALDH1A3	aldehyde dehydrogenase 1 family member A3	-2.292131795	2.292131795	down	2.94595E-11	2.63822E-09
PLAU	plasminogen activator, urokinase	-2.292253605	2.292253605	down	9.58094E-13	1.98094E-10
ICAM1	intercellular adhesion molecule 1	-2.300748572	2.300748572	down	6.54142E-15	3.52018E-12
ATF3	activating transcription factor 3	-2.305468573	2.305468573	down	2.50973E-08	5.69239E-07
APP	amyloid beta (A4) precursor protein	-2.306752681	2.306752681	down	1.66334E-12	3.00355E-10
IL32	interleukin 32	-2.311148562	2.311148562	down	2.25596E-06	2.31272E-05
TMPRSS4	transmembrane protease, serine 4	-2.314124794	2.314124794	down	2.32056E-12	3.89576E-10
NEURL1B	neuralized E3 ubiquitin protein ligase 1B	-2.325928368	2.325928368	down	2.11311E-14	9.32712E-12
DDX3X	DEAD (Asp-Glu-Ala-Asp) box helicase 3, X-linked	-2.33613989	2.33613989	down	8.0454E-08	1.46934E-06
TMBIM1	transmembrane BAX inhibitor motif containing 1	-2.340570142	2.340570142	down	1.71524E-13	5.26417E-11
CALD1	caldesmon 1	-2.353362125	2.353362125	down	2.36292E-12	3.95E-10
KLF6	Kruppel-like factor 6	-2.372031771	2.372031771	down	4.44518E-10	2.24312E-08
MFSD4	major facilitator superfamily domain containing 4	-2.372196193	2.372196193	down	2.88855E-10	1.59822E-08
TAP1	transporter 1, ATP-binding cassette, sub-family B (MDR/TAP)	-2.373605212	2.373605212	down	9.64783E-15	4.79753E-12
ADORA1	adenosine A1 receptor	-2.37533885	2.37533885	down	7.17852E-12	9.10892E-10
SYNGR3	synaptogyrin 3	-2.392319371	2.392319371	down	5.45708E-10	2.62715E-08
SGK1	serum/glucocorticoid regulated kinase 1	-2.393524655	2.393524655	down	4.42853E-14	1.77521E-11
PICALM	phosphatidylinositol binding clathrin assembly protein	-2.395466548	2.395466548	down	5.43454E-09	1.65112E-07
YAP1	Yes associated protein 1	-2.397343547	2.397343547	down	7.79909E-15	4.0677E-12
KLF5	Kruppel-like factor 5 (intestinal)	-2.408224684	2.408224684	down	1.60799E-11	1.67555E-09
OLFM4	olfactomedin 4	-2.416741778	2.416741778	down	1.52293E-09	5.91757E-08
PI3	peptidase inhibitor 3, skin-derived	-2.445303155	2.445303155	down	1.21892E-08	3.19227E-07
NPAS2	neuronal PAS domain protein 2	-2.453475163	2.453475163	down	1.54533E-09	5.97508E-08
FRY	FRY microtubule binding protein	-2.460207498	2.460207498	down	1.40354E-08	3.5572E-07
NPIPB3	nuclear pore complex interacting protein family, member B3	-2.468087142	2.468087142	down	2.63145E-08	5.93421E-07
NPIPA2	nuclear pore complex interacting protein family member A2	-2.468087142	2.468087142	down	2.63145E-08	5.93421E-07
LOC105376752	NPIP-like protein 1	-2.468087142	2.468087142	down	2.63145E-08	5.93421E-07
LOC102724993	NPIP-like protein 1	-2.468087142	2.468087142	down	2.63145E-08	5.93421E-07
NPIPB5	nuclear pore complex interacting protein family, member B5	-2.468087142	2.468087142	down	2.63145E-08	5.93421E-07
NPIPB11	nuclear pore complex interacting protein family member B11	-2.468087142	2.468087142	down	2.63145E-08	5.93421E-07
NPIPA5	nuclear pore complex interacting protein family member A5	-2.468087142	2.468087142	down	2.63145E-08	5.93421E-07
PDXDC2P	pyridoxal-dependent decarboxylase domain containing 2, pseudogene	-2.468087142	2.468087142	down	2.63145E-08	5.93421E-07
NPIPB1P	nuclear pore complex interacting protein family member B1, pseudogene	-2.468087142	2.468087142	down	2.63145E-08	5.93421E-07
NPIPA1	nuclear pore complex interacting protein family member A1	-2.468087142	2.468087142	down	2.63145E-08	5.93421E-07
NPIPA7	nuclear pore complex interacting protein family member A7	-2.468087142	2.468087142	down	2.63145E-08	5.93421E-07
NPIPA8	nuclear pore complex interacting protein family member A8	-2.468087142	2.468087142	down	2.63145E-08	5.93421E-07
NPIPB4	nuclear pore complex interacting protein family member B4	-2.468087142	2.468087142	down	2.63145E-08	5.93421E-07
PKD1P1	polycystic kidney disease 1 (autosomal dominant) pseudogene 1	-2.468087142	2.468087142	down	2.63145E-08	5.93421E-07
NPIPA3	nuclear pore complex interacting protein family member A3	-2.468087142	2.468087142	down	2.63145E-08	5.93421E-07
SGPP1	sphingosine-1-phosphate phosphatase 1	-2.471608089	2.471608089	down	8.95527E-12	1.06606E-09
SNORA4	small nucleolar RNA, H/ACA box 4	-2.473087584	2.473087584	down	2.487E-07	3.72329E-06
EIF4A2	eukaryotic translation initiation factor 4A2	-2.473087584	2.473087584	down	2.487E-07	3.72329E-06
H3F3B	H3 histone, family 3B (H3.3B)	-2.478848327	2.478848327	down	2.80522E-15	1.69539E-12
FUCA1	fucosidase, alpha-L- 1, tissue	-2.481713659	2.481713659	down	1.26546E-09	5.12497E-08
GRAMD1C	GRAM domain containing 1C	-2.488201518	2.488201518	down	9.94398E-09	2.71844E-07
REEP5	receptor accessory protein 5	-2.496638024	2.496638024	down	7.35649E-09	2.13594E-07
SAMD4A	sterile alpha motif domain containing 4A	-2.500303684	2.500303684	down	9.30518E-10	4.02139E-08
IFI27	interferon, alpha-inducible protein 27	-2.501401541	2.501401541	down	1.8958E-14	8.59082E-12
PALM2-AKAP2	PALM2-AKAP2 readthrough	-2.504692219	2.504692219	down	4.53763E-09	1.43966E-07
AKAP2	A-kinase anchoring protein 2	-2.504692219	2.504692219	down	4.53763E-09	1.43966E-07
LAMP3	lysosomal-associated membrane protein 3	-2.519807168	2.519807168	down	4.92769E-11	3.99133E-09
SERPINE1	serpin peptidase inhibitor, clade E (nexin, plasminogen activator inhibitor type 1), member 1	-2.531495641	2.531495641	down	1.18018E-11	1.30651E-09
RAD23B	RAD23 homolog B, nucleotide excision repair protein	-2.532455058	2.532455058	down	3.44629E-12	5.26786E-10
SMPD3	sphingomyelin phosphodiesterase 3, neutral membrane (neutral sphingomyelinase II)	-2.533473374	2.533473374	down	3.79782E-13	9.88036E-11
LCN2	lipocalin 2	-2.548903344	2.548903344	down	2.48924E-19	1.52268E-15
CSGALNACT1	chondroitin sulfate N-acetylgalactosaminyltransferase 1	-2.550935934	2.550935934	down	6.27667E-11	4.82529E-09
CLIP4	CAP-Gly domain containing linker protein family member 4	-2.556606278	2.556606278	down	8.14872E-09	2.3315E-07
PLK2	polo-like kinase 2	-2.56975354	2.56975354	down	4.24439E-11	3.55515E-09
NEDD9	neural precursor cell expressed, developmentally down-regulated 9	-2.58054657	2.58054657	down	8.56733E-12	1.03239E-09
MAT2A	methionine adenosyltransferase II, alpha	-2.600393585	2.600393585	down	1.38626E-08	3.52022E-07
XAF1	XIAP associated factor 1	-2.601541401	2.601541401	down	5.27965E-09	1.61405E-07
IFI6	interferon, alpha-inducible protein 6	-2.606631542	2.606631542	down	3.68846E-18	9.05609E-15
GFM2	G elongation factor, mitochondrial 2	-2.612473977	2.612473977	down	1.64701E-08	4.03877E-07
HLA-A	major histocompatibility complex, class I, A	-2.649518131	2.649518131	down	7.76984E-19	2.77482E-15
BTN3A1	butyrophilin, subfamily 3, member A1	-2.665962831	2.665962831	down	3.33078E-12	5.11119E-10
MIR18A	microRNA 18a	-2.682305075	2.682305075	down	2.68568E-10	1.52905E-08
MIR17HG	miR-17-92 cluster host gene	-2.682305075	2.682305075	down	2.68568E-10	1.52905E-08
MIR19B1	microRNA 19b-1	-2.682305075	2.682305075	down	2.68568E-10	1.52905E-08
MIR92A1	microRNA 92a-1	-2.682305075	2.682305075	down	2.68568E-10	1.52905E-08
MIR20A	microRNA 20a	-2.682305075	2.682305075	down	2.68568E-10	1.52905E-08
MIR19A	microRNA 19a	-2.682305075	2.682305075	down	2.68568E-10	1.52905E-08
HIST2H2BE	histone cluster 2, H2be	-2.697444606	2.697444606	down	6.34649E-13	1.45798E-10
IFNGR1	interferon gamma receptor 1	-2.718372821	2.718372821	down	8.93655E-12	1.06606E-09

PHLDB2	pleckstrin homology-like domain, family B, member 2	-2.808390073	2.808390073	down	4.05157E-10	2.08975E-08
LOXL4	lysyl oxidase like 4	-2.829747514	2.829747514	down	1.57275E-14	7.44384E-12
SPTA1	spectrin, alpha, erythrocytic 1	-2.899913766	2.899913766	down	3.20833E-12	5.02135E-10
CXCL3	chemokine (C-X-C motif) ligand 3	-2.935907809	2.935907809	down	3.41244E-11	2.99228E-09
SAT1	spermidine/spermine N1-acetyltransferase 1	-2.947802769	2.947802769	down	7.33519E-12	9.23762E-10
C1R	complement component 1, r subcomponent	-2.968272067	2.968272067	down	9.96385E-18	1.77918E-14
PLAT	plasminogen activator, tissue	-2.981661882	2.981661882	down	7.05871E-16	5.64227E-13
RAP1A	RAP1A, member of RAS oncogene family	-3.007622154	3.007622154	down	1.70398E-12	3.04269E-10
IGFBP3	insulin like growth factor binding protein 3	-3.064871668	3.064871668	down	8.78894E-20	1.15088E-15
BIRC3	baculoviral IAP repeat containing 3	-3.11221127	3.11221127	down	2.80036E-10	1.57093E-08
NT5E	5'-nucleotidase, ecto (CD73)	-3.172375031	3.172375031	down	7.98397E-12	9.80132E-10
CP	ceruloplasmin (ferroxidase)	-3.180565579	3.180565579	down	1.36086E-11	1.46542E-09
C3	complement component 3	-3.211632163	3.211632163	down	7.7139E-17	9.77525E-14
CD46	CD46 molecule, complement regulatory protein	-3.245925753	3.245925753	down	2.01066E-15	1.33876E-12
SLC28A3	solute carrier family 28 (concentrative nucleoside transporter), member 3	-3.261984666	3.261984666	down	2.00891E-11	1.99288E-09
PIK3AP1	phosphoinositide-3-kinase adaptor protein 1	-3.319747083	3.319747083	down	4.88974E-10	2.41317E-08
SAA1	serum amyloid A1	-3.546993451	3.546993451	down	6.81935E-12	8.7261E-10
SAA2-SAA4	SAA2-SAA4 readthrough	-3.546993451	3.546993451	down	6.81935E-12	8.7261E-10
SAA2	serum amyloid A2	-3.546993451	3.546993451	down	6.81935E-12	8.7261E-10
CYP3A7	cytochrome P450, family 3, subfamily A, polypeptide 7	-3.577098883	3.577098883	down	9.23145E-10	3.99393E-08
CYP3A7-	CYP3A7-CYP3A51P readthrough	-3.577098883	3.577098883	down	9.23145E-10	3.99393E-08
TGM2	transglutaminase 2	-3.796938593	3.796938593	down	2.84491E-11	2.5751E-09
LTB	lymphotoxin beta	-3.955289608	3.955289608	down	3.05229E-08	6.67713E-07
SERPINA3	serpin peptidase inhibitor, clade A (alpha-1 antiproteinase, antitrypsin), member 3	-3.991976792	3.991976792	down	1.84557E-11	1.85425E-09
HCP5	HLA complex P5 (non-protein coding)	-4.111695234	4.111695234	down	4.39226E-14	1.77521E-11
SERPINA1	serpin peptidase inhibitor, clade A (alpha-1 antiproteinase, antitrypsin), member 1	-4.769925279	4.769925279	down	4.41512E-11	3.66689E-09
S100A8	S100 calcium binding protein A8	-4.847720989	4.847720989	down	6.55607E-14	2.49958E-11
CXCL8	chemokine (C-X-C motif) ligand 8	-4.969784632	4.969784632	down	1.36878E-18	4.13624E-15
C15orf48	chromosome 15 open reading frame 48	-5.365254723	5.365254723	down	5.11066E-19	2.02002E-15
FN1	fibronectin 1	-5.377963635	5.377963635	down	9.86068E-18	1.77918E-14
CYP24A1	cytochrome P450, family 24, subfamily A, polypeptide 1	-5.845095785	5.845095785	down	9.48388E-17	1.12898E-13
PIGR	polymeric immunoglobulin receptor	-6.224422541	6.224422541	down	1.93242E-08	4.59524E-07
CFB	complement factor B	-6.400597849	6.400597849	down	4.65879E-19	2.02002E-15
UBD	ubiquitin D	-6.9511901	6.9511901	down	1.77657E-13	5.36852E-11
GABBR1	gamma-aminobutyric acid (GABA) B receptor, 1	-6.9511901	6.9511901	down	1.77657E-13	5.36852E-11
GABRP	gamma-aminobutyric acid (GABA) A receptor, pi	-7.491856848	7.491856848	down	2.08473E-15	1.36494E-12
S100A9	S100 calcium binding protein A9	-7.497883104	7.497883104	down	4.24081E-20	8.32981E-16
PDZK1IP1	PDZK1 interacting protein 1	-10.17003919	10.17003919	down	5.33043E-18	1.16334E-14
