# Supplementary material for: EI24 Inhibits Cell Proliferation and Drug Resistance of Esophageal Squamous Cell Carcinoma
Source: Front Oncol. 2020 Aug 21;10:1570. doi: 10.3389/fonc.2020.01570 (PMC7471874; doi:10.3389/fonc.2020.01570)
Supplement: Supplementary file 2 [file Data_Sheet_2.PDF]

| Gene Symbol | Gene Title | Fold Change | absolute FC | Regulation | P-value | FDR |
|---|---|---|---|---|---|---|
| ARHGEF25 | Rho guanine nucleotide exchange factor 25 | 2.114087421 | 2.114087421 | up | 0.009970267 | 0.478633621 |
| UGT1A6 | UDP glucuronosyltransferase 1 family, polypeptide A6 | 2.056876304 | 2.056876304 | up | 0.000530202 | 0.163057694 |
| UGT1A10 | UDP glucuronosyltransferase 1 family, polypeptide A10 | 2.056876304 | 2.056876304 | up | 0.000530202 | 0.163057694 |
| UGT1A1 | UDP glucuronosyltransferase 1 family, polypeptide A1 | 2.056876304 | 2.056876304 | up | 0.000530202 | 0.163057694 |
| UGT1A8 | UDP glucuronosyltransferase 1 family, polypeptide A8 | 2.056876304 | 2.056876304 | up | 0.000530202 | 0.163057694 |
| UGT1A3 | UDP glucuronosyltransferase 1 family, polypeptide A3 | 2.056876304 | 2.056876304 | up | 0.000530202 | 0.163057694 |
| UGT1A5 | UDP glucuronosyltransferase 1 family, polypeptide A5 | 2.056876304 | 2.056876304 | up | 0.000530202 | 0.163057694 |
| UGT1A9 | UDP glucuronosyltransferase 1 family, polypeptide A9 | 2.056876304 | 2.056876304 | up | 0.000530202 | 0.163057694 |
| UGT1A7 | UDP glucuronosyltransferase 1 family, polypeptide A7 | 2.056876304 | 2.056876304 | up | 0.000530202 | 0.163057694 |
| UGT1A4 | UDP glucuronosyltransferase 1 family, polypeptide A4 | 2.056876304 | 2.056876304 | up | 0.000530202 | 0.163057694 |
| FAM122C | family with sequence similarity 122C | 1.991444172 | 1.991444172 | up | 0.006714776 | 0.42771383 |
| NUP88 | nucleoporin 88kDa | 1.971925484 | 1.971925484 | up | 0.004320196 | 0.364793404 |
| EPB41L1 | erythrocyte membrane protein band 4.1-like 1 | 1.933790762 | 1.933790762 | up | 0.009237242 | 0.469798502 |
| TMEM106B | transmembrane protein 106B | 1.930835204 | 1.930835204 | up | 0.000144626 | 0.077989008 |
| OR6X1 | olfactory receptor, family 6, subfamily X, member 1 | 1.911875222 | 1.911875222 | up | 0.011931883 | 0.495462647 |
| PDLIM5 | PDZ and LIM domain 5 | 1.885893712 | 1.885893712 | up | 0.000378856 | 0.142035076 |
| NEBL | nebulette | 1.880719945 | 1.880719945 | up | 0.001108332 | 0.227236977 |
| GPR152 | G protein-coupled receptor 152 | 1.873555051 | 1.873555051 | up | 0.001037613 | 0.219600262 |
| FHL1 | four and a half LIM domains 1 | 1.872438545 | 1.872438545 | up | 0.034238842 | 0.595139711 |
| FRY | FRY microtubule binding protein | 1.868648264 | 1.868648264 | up | 0.00165617 | 0.255663523 |
| EGR4 | early growth response 4 | 1.863276402 | 1.863276402 | up | 0.004045056 | 0.35779398 |
| CYP1A1 | cytochrome P450, family 1, subfamily A, polypeptide 1 | 1.857958618 | 1.857958618 | up | 6.68877E-05 | 0.050635261 |
| SPIDR | scaffolding protein involved in DNA repair | 1.85529899 | 1.85529899 | up | 0.001126216 | 0.228707858 |
| OAS2 | 2'-5'-oligoadenylate synthetase 2 | 1.849640683 | 1.849640683 | up | 0.002803393 | 0.315798154 |
| TPM1 | tropomyosin 1 (alpha) | 1.843957029 | 1.843957029 | up | 0.001518131 | 0.251952305 |
| TMLHE | trimethyllysine hydroxylase, epsilon | 1.842726171 | 1.842726171 | up | 0.001706206 | 0.255663523 |
| NPSR1-AS1 | NPSR1 antisense RNA 1 | 1.825255427 | 1.825255427 | up | 0.004219348 | 0.361725211 |
| ZNF321P | zinc finger protein 321, pseudogene | 1.822781587 | 1.822781587 | up | 0.000411693 | 0.144894892 |
| ZNF816-ZNF321P | ZNF816-ZNF321P readthrough | 1.822781587 | 1.822781587 | up | 0.000411693 | 0.144894892 |
| PPFIA3 | protein tyrosine phosphatase, receptor type, f polypeptide (PTPRF), interacting protein (liprin), alpha 3 | 1.820159698 | 1.820159698 | up | 0.00279845 | 0.315798154 |
| OTUD4 | OTU deubiquitinase 4 | 1.813032813 | 1.813032813 | up | 0.010581067 | 0.486801343 |
| C17orf107 | chromosome 17 open reading frame 107 | 1.812936469 | 1.812936469 | up | 0.001463133 | 0.250769966 |
| SPAG9 | sperm associated antigen 9 | 1.811102714 | 1.811102714 | up | 0.002258139 | 0.294343195 |
| ZMYM6 | zinc finger, MYM-type 6 | 1.810395666 | 1.810395666 | up | 0.003723133 | 0.349787905 |
| ANKRD36B | ankyrin repeat domain 36B | 1.809291716 | 1.809291716 | up | 0.004826524 | 0.381949914 |
| ANKRD36 | ankyrin repeat domain 36 | 1.809291716 | 1.809291716 | up | 0.004826524 | 0.381949914 |
| GREB1L | growth regulation by estrogen in breast cancer-like | 1.809229012 | 1.809229012 | up | 0.006180572 | 0.414771056 |
| ZNF385C | zinc finger protein 385C | 1.801974217 | 1.801974217 | up | 0.002154831 | 0.288675084 |
| SHF | Src homology 2 domain containing F | 1.801349809 | 1.801349809 | up | 0.003253969 | 0.33620071 |
| CCDC185 | coiled-coil domain containing 185 | 1.800338726 | 1.800338726 | up | 0.024174069 | 0.562764151 |
| MDM2 | MDM2 proto-oncogene, E3 ubiquitin protein ligase | 1.796354023 | 1.796354023 | up | 0.015850156 | 0.523001994 |
| TRPC1 | transient receptor potential cation channel, subfamily C, member 1 | 1.79472778 | 1.79472778 | up | 0.000426941 | 0.145480134 |
| C18orf54 | chromosome 18 open reading frame 54 | 1.791550076 | 1.791550076 | up | 0.007349984 | 0.437407205 |
| ZNF273 | zinc finger protein 273 | 1.790672747 | 1.790672747 | up | 0.024545838 | 0.563613185 |
| SNX18 | sorting nexin 18 | 1.789527072 | 1.789527072 | up | 0.001911783 | 0.273662987 |
| ZNF641 | zinc finger protein 641 | 1.787716996 | 1.787716996 | up | 0.01270808 | 0.50529726 |
| HECTD4 | HECT domain containing E3 ubiquitin protein ligase 4 | 1.786953016 | 1.786953016 | up | 0.00508629 | 0.392367316 |
| LINC00525 | long intergenic non-protein coding RNA 525 | 1.786333813 | 1.786333813 | up | 0.000698097 | 0.192171998 |
| CCDC109B | coiled-coil domain containing 109B | 1.785978899 | 1.785978899 | up | 0.010744844 | 0.488995826 |
| PRKDC | protein kinase, DNA-activated, catalytic polypeptide | 1.785376534 | 1.785376534 | up | 0.009396403 | 0.473004364 |
| PARP14 | poly(ADP-ribose) polymerase family member 14 | 1.783545931 | 1.783545931 | up | 0.023412684 | 0.556698922 |
| HELT | helt bHLH transcription factor | 1.781301472 | 1.781301472 | up | 0.026287929 | 0.568317266 |
| WWOX | WW domain containing oxidoreductase | 1.780865263 | 1.780865263 | up | 0.010306068 | 0.485753032 |

| | | | | | | |
|---|---|---|---|---|---|---|
| C1QTNF1 | C1q and tumor necrosis factor related protein 1 | 1.779894465 | 1.779894465 | up | 0.006654192 | 0.42771383 |
| AKAP13 | A-kinase anchoring protein 13 | 1.779479158 | 1.779479158 | up | 0.006103484 | 0.414771056 |
| GTF2E1 | general transcription factor IIE subunit 1 | 1.779133828 | 1.779133828 | up | 0.01213801 | 0.499804145 |
| KCNIP2 | Kv channel interacting protein 2 | 1.776041183 | 1.776041183 | up | 0.000857737 | 0.202184487 |
| MAPRE3 | microtubule associated protein RP/EB family member 3 | 1.775856534 | 1.775856534 | up | 0.003458561 | 0.339991958 |
| TBL1X | transducin (beta)-like 1X-linked | 1.775150941 | 1.775150941 | up | 0.014540168 | 0.515525808 |
| LAMTOR3 | late endosomal/lysosomal adaptor, MAPK and MTOR activator 3 | 1.774289842 | 1.774289842 | up | 0.01285336 | 0.505878264 |
| EMSY | EMSY, BRCA2-interacting transcriptional repressor | 1.773547991 | 1.773547991 | up | 0.016885659 | 0.530489988 |
| FLRT3 | fibronectin leucine rich transmembrane protein 3 | 1.773080907 | 1.773080907 | up | 0.001333551 | 0.241913463 |
| ZC3H18 | zinc finger CCCH-type containing 18 | 1.773039941 | 1.773039941 | up | 0.00051998 | 0.161370449 |
| LOC84843 | uncharacterized LOC84843 | 1.772880181 | 1.772880181 | up | 0.011014623 | 0.489992559 |
| FECH | ferrochelatase | 1.772421463 | 1.772421463 | up | 0.025786984 | 0.566329497 |
| MRPL50 | mitochondrial ribosomal protein L50 | 1.771815483 | 1.771815483 | up | 0.005632526 | 0.402842701 |
| ATG12 | autophagy related 12 | 1.770743241 | 1.770743241 | up | 0.002500371 | 0.309519143 |
| TAS2R39 | taste receptor, type 2, member 39 | 1.770526417 | 1.770526417 | up | 0.018750539 | 0.539772279 |
| TMSB15A | thymosin beta 15a | 1.767636593 | 1.767636593 | up | 0.013481091 | 0.509292857 |
| ESCO1 | establishment of sister chromatid cohesion N-acetyltransferase 1 | 1.767240479 | 1.767240479 | up | 0.011591254 | 0.495320532 |
| KIF3A | kinesin family member 3A | 1.766962843 | 1.766962843 | up | 0.001352165 | 0.243935677 |
| ACE2 | angiotensin I converting enzyme 2 | 1.766836289 | 1.766836289 | up | 0.012554091 | 0.50479245 |
| TCF4 | transcription factor 4 | 1.765660989 | 1.765660989 | up | 0.028507918 | 0.574350948 |
| ANKMY1 | ankyrin repeat and MYND domain containing 1 | 1.765236768 | 1.765236768 | up | 0.01080801 | 0.488995826 |
| PRLR | prolactin receptor | 1.76363462 | 1.76363462 | up | 0.007803679 | 0.446499763 |
| NOD2 | nucleotide binding oligomerization domain containing 2 | 1.762579547 | 1.762579547 | up | 0.014190095 | 0.514636149 |
| PIFO | primary cilia formation | 1.760450956 | 1.760450956 | up | 0.00683451 | 0.430926948 |
| KMO | kynurenine 3-monooxygenase (kynurenine 3-hydroxylase) | 1.759231127 | 1.759231127 | up | 8.65075E-05 | 0.059743261 |
| OR5V1 | olfactory receptor, family 5, subfamily V, member 1 | 1.757130938 | 1.757130938 | up | 0.003763629 | 0.351078777 |
| PCDHA5 | protocadherin alpha 5 | 1.75709846 | 1.75709846 | up | 0.014700402 | 0.515525808 |
| GPATCH2L | G-patch domain containing 2 like | 1.756225828 | 1.756225828 | up | 0.044770509 | 0.623333169 |
| FREM2 | FRAS1 related extracellular matrix protein 2 | 1.75565378 | 1.75565378 | up | 0.001949658 | 0.275083511 |
| CUL3 | cullin 3 | 1.755410411 | 1.755410411 | up | 0.001548388 | 0.251952305 |
| ZNF507 | zinc finger protein 507 | 1.754648075 | 1.754648075 | up | 0.00400234 | 0.357261063 |
| SSPN | sarcospan | 1.753412011 | 1.753412011 | up | 0.00791999 | 0.447128041 |
| MRE11A | MRE11 homolog A, double strand break repair nuclease | 1.752784181 | 1.752784181 | up | 0.019103749 | 0.541887884 |
| HACD2 | 3-hydroxyacyl-CoA dehydratase 2 | 1.751529195 | 1.751529195 | up | 0.000222709 | 0.10562575 |
| LOC100128226 | hypothetic protein | 1.75035599 | 1.75035599 | up | 0.006077357 | 0.414619018 |
| NPHP1 | nephronophthisis 1 (juvenile) | 1.750218493 | 1.750218493 | up | 0.02204207 | 0.55021312 |
| SNCAIP | synuclein alpha interacting protein | 1.749381613 | 1.749381613 | up | 0.021942528 | 0.550085548 |
| FAM65B | family with sequence similarity 65 member B | 1.748884527 | 1.748884527 | up | 0.022619628 | 0.551250506 |
| IGF2-AS | IGF2 antisense RNA | 1.747394114 | 1.747394114 | up | 0.015986678 | 0.523557051 |
| IGF2 | insulin like growth factor 2 | 1.746683687 | 1.746683687 | up | 0.000233317 | 0.109339479 |
| ANO6 | anoctamin 6 | 1.746494019 | 1.746494019 | up | 0.028401004 | 0.57421459 |
| GNG12 | guanine nucleotide binding protein (G protein), gamma 12 | 1.745703288 | 1.745703288 | up | 0.003381883 | 0.338252205 |
| TDRP | testis development related protein | 1.743901275 | 1.743901275 | up | 0.023973841 | 0.562265417 |
| TMEM136 | transmembrane protein 136 | 1.743764285 | 1.743764285 | up | 0.014456144 | 0.51506237 |
| ATP10A | ATPase, class V, type 10A | 1.743409774 | 1.743409774 | up | 0.009945027 | 0.478589231 |
| HAAO | 3-hydroxyanthranilate 3,4-dioxygenase | 1.743341297 | 1.743341297 | up | 0.020755891 | 0.549146 |
| KCNAB2 | potassium channel, voltage gated subfamily A regulatory beta subunit 2 | 1.742076972 | 1.742076972 | up | 0.005066185 | 0.39233172 |
| LOC145783 | uncharacterized LOC145783 | 1.740889986 | 1.740889986 | up | 0.025142 | 0.565551322 |
| ZNF280D | zinc finger protein 280D | 1.740889986 | 1.740889986 | up | 0.025142 | 0.565551322 |
| CMTM1 | CKLF-like MARVEL transmembrane domain containing 1 | 1.740777365 | 1.740777365 | up | 0.033715384 | 0.593014207 |
| KANK4 | KN motif and ankyrin repeat domains 4 | 1.739908819 | 1.739908819 | up | 0.014146562 | 0.514636149 |
| NT5DC3 | 5'-nucleotidase domain containing 3 | 1.739257694 | 1.739257694 | up | 0.008165744 | 0.454504307 |
| TMPRSS2 | transmembrane protease, serine 2 | 1.73914116 | 1.73914116 | up | 0.035722974 | 0.602241915 |
| EGR3 | early growth response 3 | 1.736491123 | 1.736491123 | up | 0.038965994 | 0.613717043 |

| | | | | | | |
|---|---|---|---|---|---|---|
| FAM161A | family with sequence similarity 161 member A | 1.736077921 | 1.736077921 | up | 0.019284317 | 0.543126657 |
| HIPK2 | homeodomain interacting protein kinase 2 | 1.735151583 | 1.735151583 | up | 0.015156983 | 0.518081034 |
| WDR27 | WD repeat domain 27 | 1.733356457 | 1.733356457 | up | 0.010731636 | 0.488995826 |
| CACNA1A | calcium channel, voltage-dependent, P/Q type, alpha 1A subunit | 1.73330039 | 1.73330039 | up | 0.010945087 | 0.489869256 |
| PCNX | pecanex homolog (Drosophila) | 1.733136202 | 1.733136202 | up | 0.015552342 | 0.52192494 |
| NTNG2 | netrin G2 | 1.732335508 | 1.732335508 | up | 0.022862622 | 0.552817629 |
| ASCC3 | activating signal cointegrator 1 complex subunit 3 | 1.731791247 | 1.731791247 | up | 0.005501106 | 0.401886731 |
| CA12 | carbonic anhydrase XII | 1.731751235 | 1.731751235 | up | 0.038322967 | 0.612498409 |
| PDGFRL | platelet-derived growth factor receptor-like | 1.731747234 | 1.731747234 | up | 0.028800675 | 0.574350948 |
| LOC645010 | uncharacterized LOC645010 | 1.731083164 | 1.731083164 | up | 0.02301981 | 0.555254191 |
| APOLD1 | apolipoprotein L domain containing 1 | 1.729959627 | 1.729959627 | up | 0.008716142 | 0.466623412 |
| SEMA4D | sema domain, immunoglobulin domain (Ig), transmembrane domain (TM) and short cytoplasmic domain, (semaphorin) 4D | 1.729951633 | 1.729951633 | up | 0.007606686 | 0.443884559 |
| PSORS1C2 | psoriasis susceptibility 1 candidate 2 | 1.729667866 | 1.729667866 | up | 0.005835191 | 0.409879029 |
| MRC2 | mannose receptor, C type 2 | 1.729440088 | 1.729440088 | up | 0.015903397 | 0.523501532 |
| CKAP2L | cytoskeleton associated protein 2 like | 1.729364168 | 1.729364168 | up | 0.008766267 | 0.466623412 |
| AKR1C2 | aldo-keto reductase family 1, member C2 | 1.729276265 | 1.729276265 | up | 0.043269232 | 0.623333169 |
| AEBP2 | AE binding protein 2 | 1.729176381 | 1.729176381 | up | 0.014250277 | 0.514676952 |
| PPP1R3B | protein phosphatase 1, regulatory subunit 3B | 1.728585186 | 1.728585186 | up | 0.010778476 | 0.488995826 |
| LMNTD1 | lamin tail domain containing 1 | 1.72806606 | 1.72806606 | up | 0.040006698 | 0.620933229 |
| TMEM143 | transmembrane protein 143 | 1.728022141 | 1.728022141 | up | 0.014216408 | 0.514636149 |
| DHRS12 | dehydrogenase/reductase (SDR family) member 12 | 1.727706756 | 1.727706756 | up | 0.01249569 | 0.503988558 |
| SPDYE4 | speedy/RINGO cell cycle regulator family member E4 | 1.726657218 | 1.726657218 | up | 0.022643772 | 0.551250506 |
| ZNF343 | zinc finger protein 343 | 1.726657218 | 1.726657218 | up | 0.008174496 | 0.454504307 |
| FOXQ1 | forkhead box Q1 | 1.726178553 | 1.726178553 | up | 0.017424533 | 0.531030242 |
| SETD5 | SET domain containing 5 | 1.726178553 | 1.726178553 | up | 0.02872762 | 0.574350948 |
| ZNF599 | zinc finger protein 599 | 1.724978487 | 1.724978487 | up | 0.028943255 | 0.574350948 |
| MT1M | metallothionein 1M | 1.724926675 | 1.724926675 | up | 0.049988577 | 0.630493844 |
| MT1P3 | metallothionein 1 pseudogene 3 | 1.724926675 | 1.724926675 | up | 0.049988577 | 0.630493844 |
| MT1JP | metallothionein 1J, pseudogene | 1.724926675 | 1.724926675 | up | 0.049988577 | 0.630493844 |
| ERLIN2 | ER lipid raft associated 2 | 1.724787191 | 1.724787191 | up | 0.027647822 | 0.570815756 |
| GBP1 | guanylate binding protein 1, interferon-inducible | 1.72473937 | 1.72473937 | up | 0.021779334 | 0.550085548 |
| FCRL2 | Fc receptor-like 2 | 1.724352869 | 1.724352869 | up | 0.017660484 | 0.531908923 |
| SERINC5 | serine incorporator 5 | 1.72412579 | 1.72412579 | up | 0.028352663 | 0.57421459 |
| POFUT2 | protein O-fucosyltransferase 2 | 1.722939093 | 1.722939093 | up | 0.049508816 | 0.630325871 |
| LINC00472 | long intergenic non-protein coding RNA 472 | 1.72286744 | 1.72286744 | up | 0.024827306 | 0.56430245 |
| CDK1 | cyclin-dependent kinase 1 | 1.722755985 | 1.722755985 | up | 0.009880487 | 0.477233577 |
| ADIRF-AS1 | ADIRF antisense RNA 1 | 1.722628616 | 1.722628616 | up | 0.041800343 | 0.623333169 |
| CYP26A1 | cytochrome P450, family 26, subfamily A, polypeptide 1 | 1.722477378 | 1.722477378 | up | 0.015783616 | 0.522063677 |
| SNORA80A | small nucleolar RNA, H/ACA box 80A | 1.722163005 | 1.722163005 | up | 0.011655584 | 0.495320532 |
| KRTAP9-3 | keratin associated protein 9-3 | 1.721991915 | 1.721991915 | up | 0.004258535 | 0.362398259 |
| PLCB1 | phospholipase C beta 1 | 1.721868581 | 1.721868581 | up | 0.025901157 | 0.566523191 |
| NCOA7 | nuclear receptor coactivator 7 | 1.721860625 | 1.721860625 | up | 0.00080899 | 0.199221133 |
| SMARCA1 | SWI/SNF related, matrix associated, actin dependent regulator of chromatin, subfamily a, member 1 | 1.721808907 | 1.721808907 | up | 0.023995083 | 0.562265417 |
| NKX6-1 | NK6 homeobox 1 | 1.721757191 | 1.721757191 | up | 0.039584439 | 0.617458765 |
| ZBTB39 | zinc finger and BTB domain containing 39 | 1.720866326 | 1.720866326 | up | 0.022799853 | 0.551784312 |
| RNF157 | ring finger protein 157 | 1.72069934 | 1.72069934 | up | 0.019280454 | 0.543126657 |
| STMN1 | stathmin 1 | 1.720564173 | 1.720564173 | up | 0.014597949 | 0.515525808 |
| CEMIP | cell migration inducing protein, hyaluronan binding | 1.720254124 | 1.720254124 | up | 0.036271446 | 0.603215176 |
| GM2A | GM2 ganglioside activator | 1.719812997 | 1.719812997 | up | 0.016226921 | 0.524267534 |
| GDF15 | growth differentiation factor 15 | 1.719240892 | 1.719240892 | up | 0.00082233 | 0.199221133 |
| MAGOHB | mago homolog B, exon junction complex core component | 1.719189253 | 1.719189253 | up | 0.032659812 | 0.590424587 |
| CPN2 | carboxypeptidase N, polypeptide 2 | 1.718883423 | 1.718883423 | up | 0.012800798 | 0.5057776 |
| AKT2 | v-akt murine thymoma viral oncogene homolog 2 | 1.718553823 | 1.718553823 | up | 0.012529738 | 0.50479245 |
| TBX21 | T-box 21 | 1.717763835 | 1.717763835 | up | 0.0225176 | 0.551250506 |

| | | | | | | |
|---|---|---|---|---|---|---|
| FRMD4B | FERM domain containing 4B | 1.717422545 | 1.717422545 | up | 0.011740767 | 0.495320532 |
| HMGCS2 | 3-hydroxy-3-methylglutaryl-CoA synthase 2 (mitochondrial) | 1.717172574 | 1.717172574 | up | 0.02719594 | 0.568317266 |
| MOCS1 | molybdenum cofactor synthesis 1 | 1.716831402 | 1.716831402 | up | 0.009119822 | 0.466623412 |
| FRK | fyn-related Src family tyrosine kinase | 1.716633077 | 1.716633077 | up | 0.041292493 | 0.623333169 |
| TLN1 | talin 1 | 1.715665581 | 1.715665581 | up | 0.01478638 | 0.515560792 |
| ALS2 | ALS2, alsin Rho guanine nucleotide exchange factor | 1.714948242 | 1.714948242 | up | 0.002761876 | 0.315798154 |
| GABRA3 | gamma-aminobutyric acid (GABA) A receptor, alpha 3 | 1.714124267 | 1.714124267 | up | 0.018845001 | 0.539906054 |
| TNNT3 | troponin T type 3 (skeletal, fast) | 1.714088623 | 1.714088623 | up | 0.015685081 | 0.522063677 |
| CR1L | complement component (3b/4b) receptor 1-like | 1.714005457 | 1.714005457 | up | 0.014689725 | 0.515525808 |
| CR1 | complement component (3b/4b) receptor 1 (Knops blood group) | 1.714005457 | 1.714005457 | up | 0.014689725 | 0.515525808 |
| GYPB | glycophorin B (MNS blood group) | 1.713882695 | 1.713882695 | up | 0.01905716 | 0.541720736 |
| LOC284441 | actin-related protein 2 pseudogene | 1.71362532 | 1.71362532 | up | 0.026333572 | 0.568317266 |
| DLG4 | discs, large homolog 4 (Drosophila) | 1.713585728 | 1.713585728 | up | 0.026292302 | 0.568317266 |
| TMTC2 | transmembrane and tetratricopeptide repeat containing 2 | 1.713308604 | 1.713308604 | up | 0.027649743 | 0.570815756 |
| MAN1A1 | mannosidase, alpha, class 1A, member 1 | 1.713253185 | 1.713253185 | up | 0.003714826 | 0.349787905 |
| METTL7A | methyltransferase like 7A | 1.713071106 | 1.713071106 | up | 0.008359256 | 0.4606964 |
| NKX2-2 | NK2 homeobox 2 | 1.712521027 | 1.712521027 | up | 0.021233031 | 0.549146 |
| HDAC6 | histone deacetylase 6 | 1.71244585 | 1.71244585 | up | 0.005468099 | 0.401886731 |
| FRAT2 | frequently rearranged in advanced T-cell lymphomas 2 | 1.712299463 | 1.712299463 | up | 0.011613941 | 0.495320532 |
| PGM5 | phosphoglucomutase 5 | 1.712101662 | 1.712101662 | up | 0.011115567 | 0.489992559 |
| IKBKB | inhibitor of kappa light polypeptide gene enhancer in B-cells, kinase beta | 1.71200277 | 1.71200277 | up | 0.023651734 | 0.55918949 |
| NOXRED1 | NADP-dependent oxidoreductase domain containing 1 | 1.711678444 | 1.711678444 | up | 0.012631146 | 0.5051069 |
| BRSK2 | BR serine/threonine kinase 2 | 1.711615168 | 1.711615168 | up | 0.024745375 | 0.563837901 |
| CINP | cyclin-dependent kinase 2 interacting protein | 1.711456989 | 1.711456989 | up | 0.005659139 | 0.402842701 |
| HEPHL1 | hephaestin like 1 | 1.711413492 | 1.711413492 | up | 0.01237424 | 0.503186668 |
| MAPK9 | mitogen-activated protein kinase 9 | 1.711389767 | 1.711389767 | up | 0.017514458 | 0.531030242 |
| GNG13 | guanine nucleotide binding protein (G protein), gamma 13 | 1.710812558 | 1.710812558 | up | 0.017419948 | 0.531030242 |
| CD300LD | CD300 molecule like family member d | 1.710725598 | 1.710725598 | up | 0.02492623 | 0.56554526 |
| LAMP3 | lysosomal-associated membrane protein 3 | 1.709808837 | 1.709808837 | up | 0.011843697 | 0.495320532 |
| GPD1 | glycerol-3-phosphate dehydrogenase 1 | 1.709666625 | 1.709666625 | up | 0.034454319 | 0.596656508 |
| EFEMP2 | EGF containing fibulin-like extracellular matrix protein 2 | 1.709599474 | 1.709599474 | up | 0.005495262 | 0.401886731 |
| BMP8B | bone morphogenetic protein 8b | 1.709346692 | 1.709346692 | up | 0.01804304 | 0.536048499 |
| BVES | blood vessel epicardial substance | 1.709129487 | 1.709129487 | up | 0.004743616 | 0.37840054 |
| MIS18BP1 | MIS18 binding protein 1 | 1.70873464 | 1.70873464 | up | 0.000330059 | 0.128711653 |
| KIAA2012 | KIAA2012 | 1.708600412 | 1.708600412 | up | 0.026767385 | 0.568317266 |
| ZFAT | zinc finger and AT-hook domain containing | 1.707779487 | 1.707779487 | up | 0.038424465 | 0.613124876 |
| ARNT | aryl hydrocarbon receptor nuclear translocator | 1.707574317 | 1.707574317 | up | 0.005289959 | 0.39664616 |
| NUMA1 | nuclear mitotic apparatus protein 1 | 1.707507248 | 1.707507248 | up | 0.032549653 | 0.590424587 |
| JAZF1 | JAZF zinc finger 1 | 1.70688008 | 1.70688008 | up | 0.003126438 | 0.330839302 |
| ACTR10 | actin-related protein 10 homolog (S. cerevisiae) | 1.706462095 | 1.706462095 | up | 0.017780777 | 0.533491065 |
| ZC3H13 | zinc finger CCCH-type containing 13 | 1.706115167 | 1.706115167 | up | 0.011002368 | 0.489992559 |
| SMAD2 | SMAD family member 2 | 1.705957496 | 1.705957496 | up | 0.011790493 | 0.495320532 |
| ZMYND12 | zinc finger, MYND-type containing 12 | 1.705780133 | 1.705780133 | up | 0.015918561 | 0.523501532 |
| SCN3B | sodium channel, voltage gated, type III beta subunit | 1.704988138 | 1.704988138 | up | 0.038382858 | 0.61288818 |
| GJB2 | gap junction protein beta 2 | 1.704444593 | 1.704444593 | up | 0.041975626 | 0.623333169 |
| OR2D3 | olfactory receptor, family 2, subfamily D, member 3 | 1.704377647 | 1.704377647 | up | 0.03475751 | 0.598002352 |
| MFSD13A | major facilitator superfamily domain containing 13A | 1.703972087 | 1.703972087 | up | 0.012207976 | 0.502159847 |
| COL5A2 | collagen, type V, alpha 2 | 1.703861854 | 1.703861854 | up | 2.5911E-05 | 0.025499643 |
| HIF3A | hypoxia inducible factor 3, alpha subunit | 1.703775247 | 1.703775247 | up | 0.044551338 | 0.623333169 |
| SPC24 | SPC24, NDC80 kinetochore complex component | 1.703184866 | 1.703184866 | up | 0.005972801 | 0.413214927 |
| OR51F2 | olfactory receptor, family 51, subfamily F, member 2 | 1.70316519 | 1.70316519 | up | 0.016093756 | 0.524267534 |
| FMNL1 | formin like 1 | 1.702893687 | 1.702893687 | up | 0.020134656 | 0.549146 |
| MPND | MPN domain containing | 1.702759918 | 1.702759918 | up | 0.045903129 | 0.627149649 |
| NAV2 | neuron navigator 2 | 1.702468812 | 1.702468812 | up | 0.004118132 | 0.358673662 |

| | | | | | | |
|---|---|---|---|---|---|---|
| USB1 | U6 snRNA biogenesis 1 | 1.702394076 | 1.702394076 | up | 0.013072697 | 0.508746116 |
| SNCA | synuclein alpha | 1.701898544 | 1.701898544 | up | 0.008851363 | 0.466623412 |
| ABCC3 | ATP binding cassette subfamily C member 3 | 1.70183563 | 1.70183563 | up | 0.000647723 | 0.184765442 |
| AQP7 | aquaporin 7 | 1.701686218 | 1.701686218 | up | 0.023586343 | 0.558650062 |
| ATMIN | ATM interactor | 1.701458192 | 1.701458192 | up | 0.003629068 | 0.349504153 |
| RNF138 | ring finger protein 138, E3 ubiquitin protein ligase | 1.701175169 | 1.701175169 | up | 0.030801148 | 0.582823189 |
| CDH8 | cadherin 8, type 2 | 1.700715357 | 1.700715357 | up | 0.014382409 | 0.51506237 |
| VAPB | VAMP (vesicle-associated membrane protein)-associated protein B and C | 1.700188888 | 1.700188888 | up | 0.043371959 | 0.623333169 |
| ECM2 | extracellular matrix protein 2, female organ and adipocyte specific | 1.700110324 | 1.700110324 | up | 0.036020169 | 0.603119509 |
| GLIPR2 | GLI pathogenesis-related 2 | 1.699839307 | 1.699839307 | up | 0.003600807 | 0.348269714 |
| ZNF41 | zinc finger protein 41 | 1.699407341 | 1.699407341 | up | 0.022271345 | 0.550353494 |
| MADCAM1 | mucosal vascular addressin cell adhesion molecule 1 | 1.699336667 | 1.699336667 | up | 0.014746147 | 0.515525808 |
| GRIPAP1 | GRIP1 associated protein 1 | 1.699065773 | 1.699065773 | up | 0.044256852 | 0.623333169 |
| MB | myoglobin | 1.69870465 | 1.69870465 | up | 0.007541438 | 0.441112481 |
| CCDC80 | coiled-coil domain containing 80 | 1.698335755 | 1.698335755 | up | 1.4967E-05 | 0.015923666 |
| SOSTDC1 | sclerostin domain containing 1 | 1.698272972 | 1.698272972 | up | 0.033652769 | 0.593014207 |
| DCAF16 | DDB1 and CUL4 associated factor 16 | 1.698108178 | 1.698108178 | up | 0.046653797 | 0.627185789 |
| NEMP2 | nuclear envelope integral membrane protein 2 | 1.69782179 | 1.69782179 | up | 0.007718274 | 0.44458454 |
| HDAC4 | histone deacetylase 4 | 1.697500151 | 1.697500151 | up | 0.011580154 | 0.495320532 |
| CENPF | centromere protein F | 1.696653197 | 1.696653197 | up | 0.000428698 | 0.145480134 |
| OBSL1 | obscurin-like 1 | 1.696367054 | 1.696367054 | up | 0.021643579 | 0.550085548 |
| SLC17A7 | solute carrier family 17 (vesicular glutamate transporter), member 7 | 1.696316102 | 1.696316102 | up | 0.011873018 | 0.495320532 |
| SPECC1 | sperm antigen with calponin homology and coiled-coil domains 1 | 1.69579883 | 1.69579883 | up | 0.013442341 | 0.509292857 |
| AKAP11 | A-kinase anchoring protein 11 | 1.695489327 | 1.695489327 | up | 0.026981717 | 0.568317266 |
| C9orf91 | chromosome 9 open reading frame 91 | 1.69546974 | 1.69546974 | up | 0.03805823 | 0.612253826 |
| RHCE | Rh blood group, CcEe antigens | 1.695367891 | 1.695367891 | up | 0.042303293 | 0.623333169 |
| RHD | Rh blood group, D antigen | 1.695367891 | 1.695367891 | up | 0.042303293 | 0.623333169 |
| GPNMB | glycoprotein (transmembrane) nmb | 1.695172046 | 1.695172046 | up | 0.026692317 | 0.568317266 |
| CAPN10 | calpain 10 | 1.694917481 | 1.694917481 | up | 0.021097776 | 0.549146 |
| CYP11A1 | cytochrome P450, family 11, subfamily A, polypeptide 1 | 1.69447502 | 1.69447502 | up | 0.024379953 | 0.563613185 |
| ASIC1 | acid sensing ion channel 1 | 1.694361487 | 1.694361487 | up | 0.014109512 | 0.514636149 |
| NR0B2 | nuclear receptor subfamily 0 group B member 2 | 1.694228389 | 1.694228389 | up | 0.045995706 | 0.627185789 |
| ENSA | endosulfine alpha | 1.693426107 | 1.693426107 | up | 0.014916809 | 0.516117046 |
| TTC16 | tetratricopeptide repeat domain 16 | 1.692459959 | 1.692459959 | up | 0.031925977 | 0.589351082 |
| PLAT | plasminogen activator, tissue | 1.691220812 | 1.691220812 | up | 0.026573391 | 0.568317266 |
| GPC3 | glypican 3 | 1.691052796 | 1.691052796 | up | 0.033348662 | 0.593014207 |
| THAP6 | THAP domain containing 6 | 1.690802756 | 1.690802756 | up | 0.011343981 | 0.491891526 |
| RDM1 | RAD52 motif containing 1 | 1.690408237 | 1.690408237 | up | 0.008058394 | 0.453169557 |
| STARD3 | StAR related lipid transfer domain containing 3 | 1.690376992 | 1.690376992 | up | 0.001715711 | 0.25582948 |
| APPL1 | adaptor protein, phosphotyrosine interaction, PH domain and leucine zipper containing 1 | 1.690267639 | 1.690267639 | up | 0.008413355 | 0.4606964 |
| SCD5 | stearoyl-CoA desaturase 5 | 1.69020906 | 1.69020906 | up | 0.023446277 | 0.556778981 |
| COPG2 | coatomer protein complex subunit gamma 2 | 1.69020906 | 1.69020906 | up | 0.005476395 | 0.401886731 |
| TPM4 | tropomyosin 4 | 1.690146577 | 1.690146577 | up | 0.044011159 | 0.623333169 |
| RBMS3 | RNA binding motif, single stranded interacting protein 3 | 1.689916194 | 1.689916194 | up | 0.036290557 | 0.603215176 |
| WNK1 | WNK lysine deficient protein kinase 1 | 1.689596052 | 1.689596052 | up | 0.042125472 | 0.623333169 |
| CIDEB | cell death-inducing DFFA-like effector b | 1.689564822 | 1.689564822 | up | 0.006374498 | 0.417524309 |
| AGAP1 | ArfGAP with GTPase domain, ankyrin repeat and PH domain 1 | 1.688394111 | 1.688394111 | up | 0.014772738 | 0.515560792 |
| DHFR | dihydrofolate reductase | 1.688331696 | 1.688331696 | up | 0.001183633 | 0.236516265 |
| HINT3 | histidine triad nucleotide binding protein 3 | 1.688300489 | 1.688300489 | up | 0.0123592 | 0.503186668 |
| ULBP1 | UL16 binding protein 1 | 1.688183469 | 1.688183469 | up | 0.018008403 | 0.535584855 |
| SAFB2 | scaffold attachment factor B2 | 1.68809766 | 1.68809766 | up | 0.041372153 | 0.623333169 |
| NCF1C | neutrophil cytosolic factor 1C pseudogene | 1.687965054 | 1.687965054 | up | 0.026508673 | 0.568317266 |
| NCF1 | neutrophil cytosolic factor 1 | 1.687965054 | 1.687965054 | up | 0.026508673 | 0.568317266 |
| NCF1B | neutrophil cytosolic factor 1B pseudogene | 1.687965054 | 1.687965054 | up | 0.026508673 | 0.568317266 |

| | | | | | | |
|---|---|---|---|---|---|---|
| GFAP | glial fibrillary acidic protein | 1.687403543 | 1.687403543 | up | 0.021848175 | 0.550085548 |
| TENM2 | teneurin transmembrane protein 2 | 1.687282687 | 1.687282687 | up | 0.021421935 | 0.549146 |
| CCDC18 | coiled-coil domain containing 18 | 1.686846117 | 1.686846117 | up | 0.011751468 | 0.495320532 |
| RPL10 | ribosomal protein L10 | 1.686553834 | 1.686553834 | up | 0.031427908 | 0.586609582 |
| HOOK3 | hook microtubule-tethering protein 3 | 1.686503177 | 1.686503177 | up | 0.010787868 | 0.488995826 |
| PRDM15 | PR domain containing 15 | 1.686238225 | 1.686238225 | up | 0.028417557 | 0.57421459 |
| ANKRD7 | ankyrin repeat domain 7 | 1.686051226 | 1.686051226 | up | 0.01795086 | 0.535584855 |
| NFKBIA | nuclear factor of kappa light polypeptide gene enhancer in B-cells inhibitor, alpha | 1.685903199 | 1.685903199 | up | 0.002805168 | 0.315798154 |
| CXCL2 | chemokine (C-X-C motif) ligand 2 | 1.685825296 | 1.685825296 | up | 0.014257619 | 0.514676952 |
| RSBN1 | round spermatid basic protein 1 | 1.685770766 | 1.685770766 | up | 0.001299017 | 0.240074176 |
| KRT5 | keratin 5, type II | 1.685638342 | 1.685638342 | up | 0.017100904 | 0.530571823 |
| CDC37L1 | cell division cycle 37-like 1 | 1.685626658 | 1.685626658 | up | 0.025869894 | 0.566523191 |
| BDKRB1 | bradykinin receptor B1 | 1.685389103 | 1.685389103 | up | 0.028995254 | 0.574540338 |
| CD3G | CD3g molecule, gamma (CD3-TCR complex) | 1.685307329 | 1.685307329 | up | 0.024057437 | 0.56264562 |
| MR1 | major histocompatibility complex, class I-related | 1.684964702 | 1.684964702 | up | 0.002385485 | 0.300161496 |
| LIMD2 | LIM domain containing 2 | 1.684735025 | 1.684735025 | up | 0.04234986 | 0.623333169 |
| EGR1 | early growth response 1 | 1.68428744 | 1.68428744 | up | 0.009605554 | 0.475470418 |
| SPP1 | secreted phosphoprotein 1 | 1.684155133 | 1.684155133 | up | 0.016365631 | 0.524267534 |
| MTMR9LP | myotubularin related protein 9-like, pseudogene | 1.683925566 | 1.683925566 | up | 0.040936763 | 0.623333169 |
| GPD1L | glycerol-3-phosphate dehydrogenase 1-like | 1.683229276 | 1.683229276 | up | 0.025906161 | 0.566523191 |
| PRELP | proline/arginine-rich end leucine-rich repeat protein | 1.683167052 | 1.683167052 | up | 0.015049029 | 0.516932843 |
| MED28 | mediator complex subunit 28 | 1.683135941 | 1.683135941 | up | 0.001895586 | 0.273332415 |
| DNER | delta/notch like EGF repeat containing | 1.682941508 | 1.682941508 | up | 0.010579944 | 0.486801343 |
| ODF2 | outer dense fiber of sperm tails 2 | 1.682918178 | 1.682918178 | up | 0.014619325 | 0.515525808 |
| OR2T34 | olfactory receptor, family 2, subfamily T, member 34 | 1.682735435 | 1.682735435 | up | 0.036355061 | 0.603337676 |
| THTPA | thiamine triphosphatase | 1.681802587 | 1.681802587 | up | 0.034362977 | 0.596552856 |
| KCTD12 | potassium channel tetramerization domain containing 12 | 1.681771501 | 1.681771501 | up | 0.003271791 | 0.336368557 |
| TMTC4 | transmembrane and tetratricopeptide repeat containing 4 | 1.681678246 | 1.681678246 | up | 0.043129772 | 0.623333169 |
| CAPSL | calcyphosine-like | 1.681305279 | 1.681305279 | up | 0.01931999 | 0.543236724 |
| FLJ45079 | FLJ45079 protein | 1.681270318 | 1.681270318 | up | 0.030488627 | 0.581882081 |
| FLT4 | fms-related tyrosine kinase 4 | 1.681208166 | 1.681208166 | up | 0.025877877 | 0.566523191 |
| SLC16A14 | solute carrier family 16 member 14 | 1.681122711 | 1.681122711 | up | 0.004485335 | 0.370935325 |
| PDE5A | phosphodiesterase 5A | 1.681021724 | 1.681021724 | up | 0.013739207 | 0.511408446 |
| B3GALNT1 | beta-1,3-N-acetylgalactosaminyltransferase 1 (globoside blood group) | 1.680652786 | 1.680652786 | up | 0.022510104 | 0.551250506 |
| CCDC71 | coiled-coil domain containing 71 | 1.680602306 | 1.680602306 | up | 0.03832187 | 0.612498409 |
| PPTC7 | PTC7 protein phosphatase homolog | 1.680299458 | 1.680299458 | up | 0.016872855 | 0.530489988 |
| SOAT2 | sterol O-acyltransferase 2 | 1.680016073 | 1.680016073 | up | 0.048791467 | 0.62943979 |
| TNFRSF10C | tumor necrosis factor receptor superfamily member 10c, decoy without an intracellular domain | 1.67956198 | 1.67956198 | up | 0.019512844 | 0.546194159 |
| CARF | calcium responsive transcription factor | 1.679472729 | 1.679472729 | up | 0.038989472 | 0.613717043 |
| SVIP | small VCP/p97-interacting protein | 1.678817068 | 1.678817068 | up | 0.014976373 | 0.516640194 |
| CXCL14 | chemokine (C-X-C motif) ligand 14 | 1.678755007 | 1.678755007 | up | 0.013117676 | 0.508746116 |
| TNNC2 | troponin C type 2 (fast) | 1.67847576 | 1.67847576 | up | 0.033903065 | 0.594474018 |
| RASGRF1 | Ras protein specific guanine nucleotide releasing factor 1 | 1.678312888 | 1.678312888 | up | 0.00466362 | 0.377936677 |
| PARVB | parvin beta | 1.677874762 | 1.677874762 | up | 0.028348086 | 0.57421459 |
| ZFYVE9 | zinc finger, FYVE domain containing 9 | 1.677646051 | 1.677646051 | up | 0.00965669 | 0.476360405 |
| FAM122A | family with sequence similarity 122A | 1.677413496 | 1.677413496 | up | 0.03236324 | 0.590078248 |
| ENTPD4 | ectonucleoside triphosphate diphosphohydrolase 4 | 1.67728173 | 1.67728173 | up | 0.02223131 | 0.550353494 |
| QKI | QKI, KH domain containing, RNA binding | 1.677087974 | 1.677087974 | up | 0.049563281 | 0.630325871 |
| ALPK3 | alpha kinase 3 | 1.677068599 | 1.677068599 | up | 0.021483166 | 0.549146 |
| CFAP57 | cilia and flagella associated protein 57 | 1.676440991 | 1.676440991 | up | 0.035893369 | 0.603048422 |
| RSAD1 | radical S-adenosyl methionine domain containing 1 | 1.67632092 | 1.67632092 | up | 0.016623274 | 0.526872128 |
| C1RL-AS1 | C1RL antisense RNA 1 | 1.676173747 | 1.676173747 | up | 0.028881391 | 0.574350948 |
| PFKFB3 | 6-phosphofructo-2-kinase/fructose-2,6-biphosphatase 3 | 1.676011098 | 1.676011098 | up | 0.01801099 | 0.535584855 |
| KRTAP13-3 | keratin associated protein 13-3 | 1.675782642 | 1.675782642 | up | 0.041268097 | 0.623333169 |

| | | | | | | |
|---|---|---|---|---|---|---|
| TLE4 | transducin like enhancer of split 4 | 1.675449693 | 1.675449693 | up | 0.020579882 | 0.549146 |
| CORO2B | coronin, actin binding protein, 2B | 1.675364531 | 1.675364531 | up | 0.043389638 | 0.623333169 |
| TRIM36 | tripartite motif containing 36 | 1.674671781 | 1.674671781 | up | 0.016021505 | 0.524261476 |
| LOC101060341 | putative uncharacterized protein FLJ46235 | 1.67457892 | 1.67457892 | up | 0.013308899 | 0.509292857 |
| LINC01347 | long intergenic non-protein coding RNA 1347 | 1.67457892 | 1.67457892 | up | 0.013308899 | 0.509292857 |
| LOC101927999 | putative uncharacterized protein encoded by LINC00174 | 1.67457892 | 1.67457892 | up | 0.013308899 | 0.509292857 |
| LOC100132287 | uncharacterized LOC100132287 | 1.67457892 | 1.67457892 | up | 0.013308899 | 0.509292857 |
| LOC100133331 | uncharacterized LOC100133331 | 1.67457892 | 1.67457892 | up | 0.013308899 | 0.509292857 |
| LOC101060128 | putative uncharacterized protein encoded by LINC00174 | 1.67457892 | 1.67457892 | up | 0.013308899 | 0.509292857 |
| LOC100132062 | uncharacterized LOC100132062 | 1.67457892 | 1.67457892 | up | 0.013308899 | 0.509292857 |
| LOC101929819 | uncharacterized LOC101929819 | 1.67457892 | 1.67457892 | up | 0.013308899 | 0.509292857 |
| LINC01001 | long intergenic non-protein coding RNA 1001 | 1.67457892 | 1.67457892 | up | 0.013308899 | 0.509292857 |
| LOC729737 | uncharacterized LOC729737 | 1.67457892 | 1.67457892 | up | 0.013308899 | 0.509292857 |
| GCLC | glutamate-cysteine ligase, catalytic subunit | 1.674358396 | 1.674358396 | up | 0.008320991 | 0.460228374 |
| EREG | epiregulin | 1.674215264 | 1.674215264 | up | 0.001844187 | 0.267883415 |
| ITGB6 | integrin beta 6 | 1.674083748 | 1.674083748 | up | 0.002330942 | 0.298884417 |
| LOC100505984 | uncharacterized LOC100505984 | 1.674083748 | 1.674083748 | up | 0.002330942 | 0.298884417 |
| ICA1 | islet cell autoantigen 1 | 1.67402573 | 1.67402573 | up | 0.005811742 | 0.409879029 |
| KDM5A | lysine (K)-specific demethylase 5A | 1.673863289 | 1.673863289 | up | 0.034447155 | 0.596656508 |
| RET | ret proto-oncogene | 1.673809146 | 1.673809146 | up | 0.045564415 | 0.625770788 |
| DMD | dystrophin | 1.673638993 | 1.673638993 | up | 0.018816623 | 0.53988073 |
| HIST2H2AC | histone cluster 2, H2ac | 1.67285419 | 1.67285419 | up | 0.033297081 | 0.593014207 |
| FGD1 | FYVE, RhoGEF and PH domain containing 1 | 1.672834865 | 1.672834865 | up | 0.023755105 | 0.560818064 |
| DOC2A | double C2-like domains, alpha | 1.672738241 | 1.672738241 | up | 0.034117256 | 0.595139711 |
| PBX1 | pre-B-cell leukemia homeobox 1 | 1.672587519 | 1.672587519 | up | 0.047330588 | 0.627322764 |
| ANKRD35 | ankyrin repeat domain 35 | 1.672502502 | 1.672502502 | up | 0.045017144 | 0.623564284 |
| EYA2 | EYA transcriptional coactivator and phosphatase 2 | 1.672162478 | 1.672162478 | up | 0.024724647 | 0.563837901 |
| USP36 | ubiquitin specific peptidase 36 | 1.672092936 | 1.672092936 | up | 0.04775499 | 0.627854743 |
| AIG1 | androgen-induced 1 | 1.672069756 | 1.672069756 | up | 0.018247807 | 0.536306821 |
| USO1 | USO1 vesicle transport factor | 1.672042713 | 1.672042713 | up | 0.008632768 | 0.466623412 |
| SCGB2B3P | secretoglobin, family 2B, member 3, pseudogene | 1.671922957 | 1.671922957 | up | 0.027424143 | 0.568462255 |
| PRIMA1 | proline rich membrane anchor 1 | 1.671679608 | 1.671679608 | up | 0.047869734 | 0.627854743 |
| MAGIX | MAGI family member, X-linked | 1.671679608 | 1.671679608 | up | 0.016735444 | 0.528635316 |
| KIAA0930 | KIAA0930 | 1.671617811 | 1.671617811 | up | 0.017460551 | 0.531030242 |
| G6PD | glucose-6-phosphate dehydrogenase | 1.671444019 | 1.671444019 | up | 0.002519128 | 0.309987919 |
| CAMK2N1 | calcium/calmodulin-dependent protein kinase II inhibitor 1 | 1.670888004 | 1.670888004 | up | 6.93227E-05 | 0.051488483 |
| CLCC1 | chloride channel CLIC-like 1 | 1.670718147 | 1.670718147 | up | 0.010516895 | 0.486801343 |
| GNE | glucosamine (UDP-N-acetyl)-2-epimerase/N-acetylmannosamine kinase | 1.670644806 | 1.670644806 | up | 0.010600968 | 0.486801343 |
| SLC22A7 | solute carrier family 22 (organic anion transporter), member 7 | 1.670405502 | 1.670405502 | up | 0.029991424 | 0.579152964 |
| RSPH6A | radial spoke head 6 homolog A (Chlamydomonas) | 1.670170092 | 1.670170092 | up | 0.026080888 | 0.567622316 |
| SLC2A9 | solute carrier family 2 (facilitated glucose transporter), member 9 | 1.669957865 | 1.669957865 | up | 0.016282626 | 0.524267534 |
| RACGAP1 | Rac GTPase activating protein 1 | 1.669803535 | 1.669803535 | up | 0.008997118 | 0.466623412 |
| FGD4 | FYVE, RhoGEF and PH domain containing 4 | 1.66935606 | 1.66935606 | up | 0.009316757 | 0.470801192 |
| SYT2 | synaptotagmin II | 1.669348346 | 1.669348346 | up | 0.026432774 | 0.568317266 |
| PAPOLB | poly(A) polymerase beta | 1.669089946 | 1.669089946 | up | 0.0392424 | 0.614613629 |
| DOCK8 | dedicator of cytokinesis 8 | 1.668993538 | 1.668993538 | up | 0.024188923 | 0.562764151 |
| SLC1A3 | solute carrier family 1 (glial high affinity glutamate transporter), member 3 | 1.668426774 | 1.668426774 | up | 0.008957064 | 0.466623412 |
| PNISR | PNN-interacting serine/arginine-rich protein | 1.668314986 | 1.668314986 | up | 0.004061325 | 0.35779398 |
| GNAO1 | guanine nucleotide binding protein (G protein), alpha activating activity polypeptide O | 1.668307277 | 1.668307277 | up | 0.01164149 | 0.495320532 |
| CBX5 | chromobox 5 | 1.668257168 | 1.668257168 | up | 0.024299273 | 0.563333857 |
| BDNF | brain-derived neurotrophic factor | 1.667779279 | 1.667779279 | up | 0.013437036 | 0.509292857 |
| ANK3 | ankyrin 3, node of Ranvier (ankyrin G) | 1.667682947 | 1.667682947 | up | 0.015813855 | 0.522241955 |
| MST1 | macrophage stimulating 1 | 1.667555798 | 1.667555798 | up | 0.031910526 | 0.589351082 |
| KRT15 | keratin 15, type I | 1.667544239 | 1.667544239 | up | 0.000294103 | 0.123163556 |

| | | | | | | |
|---|---|---|---|---|---|---|
| CEP95 | centrosomal protein 95kDa | 1.667239892 | 1.667239892 | up | 0.003338469 | 0.338252205 |
| RASGRP4 | RAS guanyl releasing protein 4 | 1.666900938 | 1.666900938 | up | 0.022015545 | 0.550085548 |
| FGF11 | fibroblast growth factor 11 | 1.666731487 | 1.666731487 | up | 0.034165525 | 0.595139711 |
| DNAAF1 | dynein, axonemal, assembly factor 1 | 1.666619812 | 1.666619812 | up | 0.027325877 | 0.56844604 |
| RAC3 | ras-related C3 botulinum toxin substrate 3 (rho family, small GTP binding protein Rac3) | 1.666600559 | 1.666600559 | up | 0.00131395 | 0.241699342 |
| AKR1C3 | aldo-keto reductase family 1, member C3 | 1.666473492 | 1.666473492 | up | 0.044614308 | 0.623333169 |
| SWAP70 | SWAP switching B-cell complex 70kDa subunit | 1.666061553 | 1.666061553 | up | 0.040552734 | 0.623005985 |
| ACTR2 | ARP2 actin-related protein 2 homolog (yeast) | 1.665468848 | 1.665468848 | up | 0.000666525 | 0.18477288 |
| VPS36 | vacuolar protein sorting 36 homolog (S. cerevisiae) | 1.665341867 | 1.665341867 | up | 0.001769226 | 0.26084488 |
| SMOC2 | SPARC related modular calcium binding 2 | 1.665284152 | 1.665284152 | up | 0.016138276 | 0.524267534 |
| NF1 | neurofibromin 1 | 1.66510717 | 1.66510717 | up | 0.027935092 | 0.572828574 |
| CCR6 | chemokine (C-C motif) receptor 6 | 1.66485712 | 1.66485712 | up | 0.026885344 | 0.568317266 |
| OR6N2 | olfactory receptor, family 6, subfamily N, member 2 | 1.664807115 | 1.664807115 | up | 0.03187103 | 0.589351082 |
| CGREF1 | cell growth regulator with EF-hand domain 1 | 1.664760957 | 1.664760957 | up | 0.022637541 | 0.551250506 |
| ATP7A | ATPase, Cu++ transporting, alpha polypeptide | 1.664749418 | 1.664749418 | up | 0.03808768 | 0.612253826 |
| TRIL | TLR4 interactor with leucine-rich repeats | 1.664745572 | 1.664745572 | up | 0.032401694 | 0.590283874 |
| BORCS5 | BLOC-1 related complex subunit 5 | 1.664703262 | 1.664703262 | up | 0.007372525 | 0.437407205 |
| HPCAL4 | hippocalcin like 4 | 1.66428407 | 1.66428407 | up | 0.049046249 | 0.62943979 |
| CASC5 | cancer susceptibility candidate 5 | 1.66420332 | 1.66420332 | up | 0.032763134 | 0.590424587 |
| THOC3 | THO complex 3 | 1.664018765 | 1.664018765 | up | 0.044071824 | 0.623333169 |
| PEX26 | peroxisomal biogenesis factor 26 | 1.66388805 | 1.66388805 | up | 0.041309845 | 0.623333169 |
| MEI1 | meiotic double-stranded break formation protein 1 | 1.663811164 | 1.663811164 | up | 0.042749034 | 0.623333169 |
| TNFAIP2 | TNF alpha induced protein 2 | 1.663684309 | 1.663684309 | up | 0.007831399 | 0.446814625 |
| CLDN1 | claudin 1 | 1.663549777 | 1.663549777 | up | 0.018928401 | 0.540331052 |
| C9orf3 | chromosome 9 open reading frame 3 | 1.663434473 | 1.663434473 | up | 0.018414065 | 0.537128348 |
| CPT1C | carnitine palmitoyltransferase 1C | 1.663230788 | 1.663230788 | up | 0.001220714 | 0.240074176 |
| GPATCH2 | G-patch domain containing 2 | 1.662285708 | 1.662285708 | up | 0.04228762 | 0.623333169 |
| OLFM2 | olfactomedin 2 | 1.662258824 | 1.662258824 | up | 0.036632684 | 0.604603394 |
| LOXL2 | lysyl oxidase like 2 | 1.662239621 | 1.662239621 | up | 0.025531387 | 0.566329497 |
| LOC100506276 | uncharacterized LOC100506276 | 1.662039922 | 1.662039922 | up | 0.041642759 | 0.623333169 |
| RBM43 | RNA binding motif protein 43 | 1.661644436 | 1.661644436 | up | 0.043419956 | 0.623333169 |
| FBXL20 | F-box and leucine-rich repeat protein 20 | 1.66157917 | 1.66157917 | up | 0.017502364 | 0.531030242 |
| MESTIT1 | MEST intronic transcript 1, antisense RNA | 1.661448647 | 1.661448647 | up | 0.037095548 | 0.60692696 |
| PGBD5 | piggyBac transposable element derived 5 | 1.661272074 | 1.661272074 | up | 0.046287075 | 0.627185789 |
| FARS2 | phenylalanyl-tRNA synthetase 2, mitochondrial | 1.661252882 | 1.661252882 | up | 0.013139484 | 0.509090329 |
| USP45 | ubiquitin specific peptidase 45 | 1.66119147 | 1.66119147 | up | 0.01990404 | 0.549146 |
| RPS6 | ribosomal protein S6 | 1.661064816 | 1.661064816 | up | 0.040546746 | 0.623005985 |
| ZYX | zyxin | 1.660991898 | 1.660991898 | up | 0.000892871 | 0.207975631 |
| ANTXR1 | anthrax toxin receptor 1 | 1.66098806 | 1.66098806 | up | 0.021622331 | 0.550085548 |
| GLIS3 | GLIS family zinc finger 3 | 1.660700258 | 1.660700258 | up | 0.025614579 | 0.566329497 |
| ZCCHC24 | zinc finger, CCHC domain containing 24 | 1.66053911 | 1.66053911 | up | 0.004970476 | 0.388818027 |
| NOTUM | notum pectinacetylesterase homolog (Drosophila) | 1.660493071 | 1.660493071 | up | 0.049626327 | 0.630325871 |
| NT5C1A | 5'-nucleotidase, cytosolic IA | 1.659890841 | 1.659890841 | up | 0.047066124 | 0.627322764 |
| SSBP2 | single-stranded DNA binding protein 2 | 1.659254326 | 1.659254326 | up | 0.021950974 | 0.550085548 |
| HLA-DMA | major histocompatibility complex, class II, DM alpha | 1.659154653 | 1.659154653 | up | 0.01723808 | 0.530571823 |
| LRRTM4 | leucine rich repeat transmembrane neuronal 4 | 1.659116319 | 1.659116319 | up | 0.041619166 | 0.623333169 |
| CDKN3 | cyclin-dependent kinase inhibitor 3 | 1.658173577 | 1.658173577 | up | 0.033037864 | 0.592229281 |
| LINC00654 | long intergenic non-protein coding RNA 654 | 1.657974367 | 1.657974367 | up | 0.033123143 | 0.59236436 |
| PFAS | phosphoribosylformylglycinamidine synthase | 1.657913077 | 1.657913077 | up | 0.014058578 | 0.513372826 |
| PACRGL | PARK2 co-regulated like | 1.657568359 | 1.657568359 | up | 0.041921905 | 0.623333169 |
| NAGS | N-acetylglutamate synthase | 1.657453469 | 1.657453469 | up | 0.033076216 | 0.592343125 |
| RILPL1 | Rab interacting lysosomal protein-like 1 | 1.657388368 | 1.657388368 | up | 0.049601982 | 0.630325871 |
| MBD3L2 | methyl-CpG binding domain protein 3-like 2 | 1.657353904 | 1.657353904 | up | 0.044912259 | 0.623333169 |
| MBD3L4 | methyl-CpG binding domain protein 3-like 4 | 1.657353904 | 1.657353904 | up | 0.044912259 | 0.623333169 |

| | | | | | | |
|---|---|---|---|---|---|---|
| MBD3L5 | methyl-CpG binding domain protein 3-like 5 | 1.657353904 | 1.657353904 | up | 0.044912259 | 0.623333169 |
| MBD3L3 | methyl-CpG binding domain protein 3-like 3 | 1.657353904 | 1.657353904 | up | 0.044912259 | 0.623333169 |
| MYO18A | myosin XVIIIA | 1.65732327 | 1.65732327 | up | 0.03670541 | 0.604817277 |
| CCDC186 | coiled-coil domain containing 186 | 1.65727732 | 1.65727732 | up | 0.026507405 | 0.568317266 |
| PAPOLA | poly(A) polymerase alpha | 1.657047589 | 1.657047589 | up | 0.021919286 | 0.550085548 |
| CNOT6L | CCR4-NOT transcription complex subunit 6-like | 1.656645635 | 1.656645635 | up | 0.045618793 | 0.625770788 |
| TTC3 | tetratricopeptide repeat domain 3 | 1.656645635 | 1.656645635 | up | 0.009456447 | 0.473604392 |
| TRIM16 | tripartite motif containing 16 | 1.656534637 | 1.656534637 | up | 0.000549992 | 0.165072126 |
| TRIM16L | tripartite motif containing 16-like | 1.656534637 | 1.656534637 | up | 0.000549992 | 0.165072126 |
| PDCD6IP | programmed cell death 6 interacting protein | 1.655853498 | 1.655853498 | up | 0.002916412 | 0.326149299 |
| RIMKLA | ribosomal modification protein rimK-like family member A | 1.65581524 | 1.65581524 | up | 0.0458793 | 0.627096746 |
| PASK | PAS domain containing serine/threonine kinase | 1.65581524 | 1.65581524 | up | 0.022015675 | 0.550085548 |
| USP42 | ubiquitin specific peptidase 42 | 1.655612488 | 1.655612488 | up | 0.022771572 | 0.551784312 |
| AGR2 | anterior gradient 2, protein disulphide isomerase family member | 1.655513034 | 1.655513034 | up | 0.0068271 | 0.430926948 |
| ABCG2 | ATP binding cassette subfamily G member 2 (Junior blood group) | 1.655497734 | 1.655497734 | up | 0.007684805 | 0.44458454 |
| DOCK7 | dedicator of cytokinesis 7 | 1.655394462 | 1.655394462 | up | 0.017550371 | 0.531030242 |
| RAB12 | RAB12, member RAS oncogene family | 1.655310319 | 1.655310319 | up | 0.034004132 | 0.595139711 |
| PARM1 | prostate androgen-regulated mucin-like protein 1 | 1.655004381 | 1.655004381 | up | 0.014988012 | 0.516640194 |
| PPP1R36 | protein phosphatase 1 regulatory subunit 36 | 1.654981438 | 1.654981438 | up | 0.024648772 | 0.563613185 |
| RGL1 | ral guanine nucleotide dissociation stimulator-like 1 | 1.654966142 | 1.654966142 | up | 0.04262105 | 0.623333169 |
| PLEKHH2 | pleckstrin homology, MyTH4 and FERM domain containing H2 | 1.654927905 | 1.654927905 | up | 0.032315556 | 0.589996854 |
| TMEM128 | transmembrane protein 128 | 1.654916434 | 1.654916434 | up | 0.023310017 | 0.556498532 |
| KSR1 | kinase suppressor of ras 1 | 1.654916434 | 1.654916434 | up | 0.020959536 | 0.549146 |
| TSPYL2 | TSPY-like 2 | 1.654759671 | 1.654759671 | up | 0.023872627 | 0.561713077 |
| GNS | glucosamine (N-acetyl)-6-sulfatase | 1.65467556 | 1.65467556 | up | 0.01970679 | 0.548238708 |
| MKRN3 | makorin ring finger protein 3 | 1.654622038 | 1.654622038 | up | 0.048777339 | 0.62943979 |
| ZFP91-CNTF | ZFP91-CNTF readthrough (NMD candidate) | 1.65417863 | 1.65417863 | up | 0.047456723 | 0.627322764 |
| CNTF | ciliary neurotrophic factor | 1.65417863 | 1.65417863 | up | 0.047456723 | 0.627322764 |
| OS9 | osteosarcoma amplified 9, endoplasmic reticulum lectin | 1.653853795 | 1.653853795 | up | 0.003785577 | 0.351292726 |
| LTBP1 | latent transforming growth factor beta binding protein 1 | 1.653735342 | 1.653735342 | up | 0.030902914 | 0.583508935 |
| FMR1 | fragile X mental retardation 1 | 1.65357105 | 1.65357105 | up | 0.03194937 | 0.589351082 |
| CALCOCO1 | calcium binding and coiled-coil domain 1 | 1.653448796 | 1.653448796 | up | 0.029609624 | 0.57728016 |
| NAAA | N-acylethanolamine acid amidase | 1.653368572 | 1.653368572 | up | 0.014458121 | 0.51506237 |
| ITGAX | integrin alpha X | 1.653250154 | 1.653250154 | up | 0.041137743 | 0.623333169 |
| SERPINF2 | serpin peptidase inhibitor, clade F (alpha-2 antiplasmin, pigment epithelium derived factor), member 2 | 1.653093549 | 1.653093549 | up | 0.021130919 | 0.549146 |
| SETBP1 | SET binding protein 1 | 1.652872035 | 1.652872035 | up | 0.045422919 | 0.624763521 |
| NUAK1 | NUAK family, SNF1-like kinase, 1 | 1.652772745 | 1.652772745 | up | 0.003209861 | 0.333998479 |
| MMAB | methylmalonic aciduria (cobalamin deficiency) cblB type | 1.652642914 | 1.652642914 | up | 0.001127126 | 0.228707858 |
| GNAZ | guanine nucleotide binding protein (G protein), alpha z polypeptide | 1.651951925 | 1.651951925 | up | 0.010204409 | 0.483971757 |
| C14orf93 | chromosome 14 open reading frame 93 | 1.651906124 | 1.651906124 | up | 0.039057052 | 0.613731439 |
| MAGI2 | membrane associated guanylate kinase, WW and PDZ domain containing 2 | 1.651879407 | 1.651879407 | up | 0.032730381 | 0.590424587 |
| PPA2 | pyrophosphatase (inorganic) 2 | 1.651551208 | 1.651551208 | up | 0.035219129 | 0.600433528 |
| CDH23 | cadherin-related 23 | 1.651013255 | 1.651013255 | up | 0.039869518 | 0.620096235 |
| DMPK | dystrophia myotonica protein kinase | 1.650879747 | 1.650879747 | up | 0.022258429 | 0.550353494 |
| ACOX2 | acyl-CoA oxidase 2, branched chain | 1.650509797 | 1.650509797 | up | 0.042218149 | 0.623333169 |
| SLC2A5 | solute carrier family 2 (facilitated glucose/fructose transporter), member 5 | 1.65004843 | 1.65004843 | up | 0.047112707 | 0.627322764 |
| DLST | dihydrolipoamide S-succinyltransferase (E2 component of 2-oxo-glutarate complex) | 1.649747276 | 1.649747276 | up | 0.001537397 | 0.251952305 |
| NR4A1 | nuclear receptor subfamily 4 group A member 1 | 1.649587191 | 1.649587191 | up | 0.021825171 | 0.550085548 |
| TULP4 | tubby like protein 4 | 1.649434744 | 1.649434744 | up | 0.006538205 | 0.423316505 |
| EFHC2 | EF-hand domain (C-terminal) containing 2 | 1.649316607 | 1.649316607 | up | 0.038304615 | 0.612498409 |
| COQ4 | coenzyme Q4 | 1.649160375 | 1.649160375 | up | 0.031392869 | 0.58634196 |
| STRADA | STE20-related kinase adaptor alpha | 1.649084169 | 1.649084169 | up | 0.02940964 | 0.576629742 |
| DNAJC30 | DnaJ heat shock protein family (Hsp40) member C30 | 1.648783191 | 1.648783191 | up | 0.003652236 | 0.349787905 |
| EBNA1BP2 | EBNA1 binding protein 2 | 1.648459416 | 1.648459416 | up | 0.047400529 | 0.627322764 |

| | | | | | | |
|---|---|---|---|---|---|---|
| ZNF419 | zinc finger protein 419 | 1.647945315 | 1.647945315 | up | 0.032294911 | 0.589996854 |
| CD80 | CD80 molecule | 1.647808248 | 1.647808248 | up | 0.046523511 | 0.627185789 |
| GABARAP | GABA(A) receptor-associated protein | 1.647762562 | 1.647762562 | up | 0.013817153 | 0.511408446 |
| ZBTB49 | zinc finger and BTB domain containing 49 | 1.647720684 | 1.647720684 | up | 0.044482553 | 0.623333169 |
| SUGP1 | SURP and G-patch domain containing 1 | 1.647545569 | 1.647545569 | up | 0.0272058 | 0.568317266 |
| PBX2 | pre-B-cell leukemia homeobox 2 | 1.647515116 | 1.647515116 | up | 0.045694834 | 0.625770788 |
| PDLIM3 | PDZ and LIM domain 3 | 1.647461825 | 1.647461825 | up | 0.034819286 | 0.598019712 |
| CCDC53 | coiled-coil domain containing 53 | 1.647187784 | 1.647187784 | up | 0.017153458 | 0.530571823 |
| TRPV4 | transient receptor potential cation channel, subfamily V, member 4 | 1.647020337 | 1.647020337 | up | 0.028673389 | 0.574350948 |
| AFAP1L1 | actin filament associated protein 1 like 1 | 1.646970867 | 1.646970867 | up | 0.020997387 | 0.549146 |
| CEND1 | cell cycle exit and neuronal differentiation 1 | 1.646879542 | 1.646879542 | up | 0.041373728 | 0.623333169 |
| MANEA | mannosidase, endo-alpha | 1.646795832 | 1.646795832 | up | 0.027225997 | 0.568317266 |
| BBOF1 | basal body orientation factor 1 | 1.646750174 | 1.646750174 | up | 0.010950866 | 0.489869256 |
| PNMA2 | paraneoplastic Ma antigen 2 | 1.646259428 | 1.646259428 | up | 0.03151993 | 0.587491497 |
| SMAD6 | SMAD family member 6 | 1.646228999 | 1.646228999 | up | 0.027748786 | 0.571601753 |
| HLA-F | major histocompatibility complex, class I, F | 1.646084468 | 1.646084468 | up | 0.001136427 | 0.229412627 |
| LOC643923 | uncharacterized LOC643923 | 1.645924739 | 1.645924739 | up | 0.049629617 | 0.630325871 |
| ELMOD1 | ELMO/CED-12 domain containing 1 | 1.645924739 | 1.645924739 | up | 0.049629617 | 0.630325871 |
| DEGS2 | delta(4)-desaturase, sphingolipid 2 | 1.645879105 | 1.645879105 | up | 0.020675302 | 0.549146 |
| ARHGAP29 | Rho GTPase activating protein 29 | 1.645871499 | 1.645871499 | up | 0.046363605 | 0.627185789 |
| GLS2 | glutaminase 2 | 1.64577263 | 1.64577263 | up | 0.047797729 | 0.627854743 |
| ACVR1B | activin A receptor type IB | 1.645715593 | 1.645715593 | up | 0.015670343 | 0.522063677 |
| TMEM104 | transmembrane protein 104 | 1.6455559 | 1.6455559 | up | 0.022549383 | 0.551250506 |
| NFKBID | nuclear factor of kappa light polypeptide gene enhancer in B-cells inhibitor, delta | 1.644928682 | 1.644928682 | up | 0.017201612 | 0.530571823 |
| LY86 | lymphocyte antigen 86 | 1.644902078 | 1.644902078 | up | 0.048464093 | 0.628803231 |
| NR2C1 | nuclear receptor subfamily 2 group C member 1 | 1.644662662 | 1.644662662 | up | 0.008628568 | 0.466623412 |
| OGDHL | oxoglutarate dehydrogenase-like | 1.644286508 | 1.644286508 | up | 0.012543438 | 0.50479245 |
| MEIS2 | Meis homeobox 2 | 1.644278909 | 1.644278909 | up | 0.020653048 | 0.549146 |
| ARHGAP18 | Rho GTPase activating protein 18 | 1.644157343 | 1.644157343 | up | 0.012628506 | 0.5051069 |
| C17orf102 | chromosome 17 open reading frame 102 | 1.644039584 | 1.644039584 | up | 0.048850964 | 0.62943979 |
| TFDP1 | transcription factor Dp-1 | 1.643731931 | 1.643731931 | up | 0.025752221 | 0.566329497 |
| FCGRT | Fc fragment of IgG, receptor, transporter, alpha | 1.643504078 | 1.643504078 | up | 0.041176759 | 0.623333169 |
| POLG2 | polymerase (DNA directed), gamma 2, accessory subunit | 1.643044668 | 1.643044668 | up | 0.00610059 | 0.414771056 |
| LIPG | lipase, endothelial | 1.642832093 | 1.642832093 | up | 0.033448115 | 0.593014207 |
| CALD1 | caldesmon 1 | 1.642771362 | 1.642771362 | up | 0.004899522 | 0.384969395 |
| DCAF13 | DDB1 and CUL4 associated factor 13 | 1.642706838 | 1.642706838 | up | 0.003385529 | 0.338252205 |
| KLK10 | kallikrein related peptidase 10 | 1.642577797 | 1.642577797 | up | 0.004479409 | 0.370935325 |
| TP53 | tumor protein p53 | 1.641572388 | 1.641572388 | up | 0.020500744 | 0.549146 |
| STOM | stomatin | 1.641469984 | 1.641469984 | up | 0.019148128 | 0.541887884 |
| FBXO45 | F-box protein 45 | 1.641219692 | 1.641219692 | up | 0.045191846 | 0.624555255 |
| TTI2 | TELO2 interacting protein 2 | 1.641049059 | 1.641049059 | up | 0.014843544 | 0.516115951 |
| NIPAL4 | NIPA-like domain containing 4 | 1.641033893 | 1.641033893 | up | 0.030215222 | 0.579778515 |
| FNBP1L | formin binding protein 1-like | 1.640901193 | 1.640901193 | up | 0.00866489 | 0.466623412 |
| EIF3G | eukaryotic translation initiation factor 3 subunit G | 1.640461462 | 1.640461462 | up | 0.047377388 | 0.627322764 |
| EHHADH | enoyl-CoA, hydratase/3-hydroxyacyl CoA dehydrogenase | 1.640150689 | 1.640150689 | up | 0.023278526 | 0.556498532 |
| ITGB7 | integrin beta 7 | 1.640037007 | 1.640037007 | up | 0.037510152 | 0.609325627 |
| FTH1 | ferritin, heavy polypeptide 1 | 1.639942277 | 1.639942277 | up | 0.003823593 | 0.351759623 |
| ARL4C | ADP ribosylation factor like GTPase 4C | 1.639802088 | 1.639802088 | up | 0.01568218 | 0.522063677 |
| BOC | BOC cell adhesion associated, oncogene regulated | 1.63979451 | 1.63979451 | up | 0.046517301 | 0.627185789 |
| CHAC1 | ChaC glutathione-specific gamma-glutamylcyclotransferase 1 | 1.639703583 | 1.639703583 | up | 0.02280587 | 0.551784312 |
| SLC2A3 | solute carrier family 2 (facilitated glucose transporter), member 3 | 1.639313412 | 1.639313412 | up | 0.018256094 | 0.536306821 |
| SLC2A14 | solute carrier family 2 (facilitated glucose transporter), member 14 | 1.639313412 | 1.639313412 | up | 0.018256094 | 0.536306821 |
| CLGN | calmegin | 1.639222512 | 1.639222512 | up | 0.027378984 | 0.56844604 |
| GNG7 | guanine nucleotide binding protein (G protein), gamma 7 | 1.639177064 | 1.639177064 | up | 0.042103959 | 0.623333169 |

| | | | | | | |
|---|---|---|---|---|---|---|
| RHBDL2 | rhomboid, veinlet-like 2 (Drosophila) | 1.639033152 | 1.639033152 | up | 0.026815447 | 0.568317266 |
| ATP6V1E2 | ATPase, H+ transporting, lysosomal 31kDa, V1 subunit E2 | 1.639021791 | 1.639021791 | up | 0.021170801 | 0.549146 |
| ZNF564 | zinc finger protein 564 | 1.638787018 | 1.638787018 | up | 0.013002619 | 0.508400247 |
| KIAA0319L | KIAA0319-like | 1.63854092 | 1.63854092 | up | 0.018105505 | 0.536306821 |
| FAM213B | family with sequence similarity 213 member B | 1.638419778 | 1.638419778 | up | 0.041083571 | 0.623333169 |
| ADD3 | adducin 3 | 1.638238081 | 1.638238081 | up | 0.001684031 | 0.255663523 |
| COX15 | cytochrome c oxidase assembly homolog 15 (yeast) | 1.637848259 | 1.637848259 | up | 0.035910337 | 0.603076123 |
| NEO1 | neogenin 1 | 1.637594735 | 1.637594735 | up | 0.011315798 | 0.491885982 |
| FKBP10 | FK506 binding protein 10 | 1.637235328 | 1.637235328 | up | 0.008743318 | 0.466623412 |
| ITGB4 | integrin beta 4 | 1.637205066 | 1.637205066 | up | 0.003462146 | 0.339991958 |
| DND1 | DND microRNA-mediated repression inhibitor 1 | 1.637046198 | 1.637046198 | up | 0.007781515 | 0.446499763 |
| TMEM144 | transmembrane protein 144 | 1.636618845 | 1.636618845 | up | 0.013947936 | 0.511949108 |
| PCSK6 | proprotein convertase subtilisin/kexin type 6 | 1.636206725 | 1.636206725 | up | 0.018334853 | 0.537128348 |
| MUTYH | mutY DNA glycosylase | 1.635246775 | 1.635246775 | up | 0.005207437 | 0.39485568 |
| LRTOMT | leucine rich transmembrane and O-methyltransferase domain containing | 1.635095653 | 1.635095653 | up | 0.008661126 | 0.466623412 |
| MTFR1L | mitochondrial fission regulator 1-like | 1.634970988 | 1.634970988 | up | 0.026517568 | 0.568317266 |
| PLCE1 | phospholipase C epsilon 1 | 1.634944545 | 1.634944545 | up | 0.018181836 | 0.536306821 |
| S1PR4 | sphingosine-1-phosphate receptor 4 | 1.634857664 | 1.634857664 | up | 0.041979624 | 0.623333169 |
| DBT | dihydrolipoamide branched chain transacylase E2 | 1.634736794 | 1.634736794 | up | 0.003042953 | 0.326391956 |
| RALGAPB | Ral GTPase activating protein, beta subunit (non-catalytic) | 1.63460838 | 1.63460838 | up | 0.04704737 | 0.627322764 |
| ETFDH | electron-transferring-flavoprotein dehydrogenase | 1.634600827 | 1.634600827 | up | 0.012443746 | 0.503406041 |
| ZNF239 | zinc finger protein 239 | 1.63455173 | 1.63455173 | up | 0.003704902 | 0.349787905 |
| SEMA4F | sema domain, immunoglobulin domain (Ig), transmembrane domain (TM) and short cytoplasmic domain, (semaphorin) 4F | 1.634344029 | 1.634344029 | up | 0.026944125 | 0.568317266 |
| CRIP2 | cysteine-rich protein 2 | 1.633751283 | 1.633751283 | up | 0.013421478 | 0.509292857 |
| RELA | v-rel avian reticuloendotheliosis viral oncogene homolog A | 1.633577653 | 1.633577653 | up | 0.044280561 | 0.623333169 |
| PTGES3L | prostaglandin E synthase 3 (cytosolic)-like | 1.633562556 | 1.633562556 | up | 0.048984615 | 0.62943979 |
| ZNF326 | zinc finger protein 326 | 1.633558781 | 1.633558781 | up | 0.003992305 | 0.357261063 |
| PHF21A | PHD finger protein 21A | 1.633464426 | 1.633464426 | up | 0.025267229 | 0.565656951 |
| NKX2-5 | NK2 homeobox 5 | 1.633404041 | 1.633404041 | up | 0.027604759 | 0.570725492 |
| TM7SF2 | transmembrane 7 superfamily member 2 | 1.633396494 | 1.633396494 | up | 0.027065785 | 0.568317266 |
| UBP1 | upstream binding protein 1 (LBP-1a) | 1.633354981 | 1.633354981 | up | 0.024170476 | 0.562764151 |
| FLJ42393 | uncharacterized LOC401105 | 1.633347433 | 1.633347433 | up | 0.010973478 | 0.489992559 |
| IGF1R | insulin like growth factor 1 receptor | 1.633298374 | 1.633298374 | up | 0.032313582 | 0.589996854 |
| DYNLT3 | dynein, light chain, Tctex-type 3 | 1.633268184 | 1.633268184 | up | 0.000767563 | 0.195840366 |
| PPFIBP1 | PTPRF interacting protein, binding protein 1 (liprin beta 1) | 1.6330531 | 1.6330531 | up | 0.011909035 | 0.495320532 |
| COLGALT2 | collagen beta(1-O)galactosyltransferase 2 | 1.632936137 | 1.632936137 | up | 0.025616545 | 0.566329497 |
| KCNQ2 | potassium channel, voltage gated KQT-like subfamily Q, member 2 | 1.632917273 | 1.632917273 | up | 0.049703484 | 0.630325871 |
| NPAT | nuclear protein, ataxia-telangiectasia locus | 1.632777683 | 1.632777683 | up | 0.049388704 | 0.630325871 |
| ELFN1 | extracellular leucine-rich repeat and fibronectin type III domain containing 1 | 1.632743731 | 1.632743731 | up | 0.029276683 | 0.576629742 |
| ADAM17 | ADAM metallopeptidase domain 17 | 1.632087459 | 1.632087459 | up | 0.006216561 | 0.414771056 |
| HOMER2 | homer scaffolding protein 2 | 1.631849908 | 1.631849908 | up | 0.045583135 | 0.625770788 |
| USP15 | ubiquitin specific peptidase 15 | 1.631770732 | 1.631770732 | up | 0.047429504 | 0.627322764 |
| CSDC2 | cold shock domain containing C2, RNA binding | 1.631748111 | 1.631748111 | up | 0.036323804 | 0.603215176 |
| LMBRD2 | LMBR1 domain containing 2 | 1.63169156 | 1.63169156 | up | 0.008530293 | 0.466158616 |
| ZNF37A | zinc finger protein 37A | 1.6316614 | 1.6316614 | up | 0.040744313 | 0.623259064 |
| PLEKHM3 | pleckstrin homology domain containing M3 | 1.631631241 | 1.631631241 | up | 0.046221871 | 0.627185789 |
| PPIC | peptidylprolyl isomerase C (cyclophilin C) | 1.631469145 | 1.631469145 | up | 0.001708104 | 0.255663523 |
| KRT2 | keratin 2, type II | 1.631333448 | 1.631333448 | up | 0.043235714 | 0.623333169 |
| RHOU | ras homolog family member U | 1.63117515 | 1.63117515 | up | 0.04902211 | 0.62943979 |
| TP53I11 | tumor protein p53 inducible protein 11 | 1.63106209 | 1.63106209 | up | 0.041282397 | 0.623333169 |
| CNST | consortin, connexin sorting protein | 1.630930196 | 1.630930196 | up | 0.041222148 | 0.623333169 |
| GJC1 | gap junction protein gamma 1 | 1.630711652 | 1.630711652 | up | 0.040544386 | 0.623005985 |
| BMP8A | bone morphogenetic protein 8a | 1.630542112 | 1.630542112 | up | 0.048127761 | 0.627892392 |
| RPRD1A | regulation of nuclear pre-mRNA domain containing 1A | 1.630349989 | 1.630349989 | up | 0.005941322 | 0.413214927 |

| | | | | | | |
|---|---|---|---|---|---|---|
| PPP1R11 | protein phosphatase 1 regulatory inhibitor subunit 11 | 1.630067495 | 1.630067495 | up | 0.045127122 | 0.624184523 |
| ELF2 | E74-like factor 2 (ets domain transcription factor) | 1.629916852 | 1.629916852 | up | 0.024598264 | 0.563613185 |
| VLDLR | very low density lipoprotein receptor | 1.629845301 | 1.629845301 | up | 0.026882068 | 0.568317266 |
| C19orf12 | chromosome 19 open reading frame 12 | 1.62951395 | 1.62951395 | up | 0.033446345 | 0.593014207 |
| LBH | limb bud and heart development | 1.629314418 | 1.629314418 | up | 0.006711654 | 0.42771383 |
| PKP2 | plakophilin 2 | 1.628968119 | 1.628968119 | up | 0.00262914 | 0.314577816 |
| LURAP1 | leucine rich adaptor protein 1 | 1.628862739 | 1.628862739 | up | 0.038287034 | 0.612498409 |
| ZNF668 | zinc finger protein 668 | 1.628821341 | 1.628821341 | up | 0.037676007 | 0.610579997 |
| MYADM | myeloid-associated differentiation marker | 1.628584266 | 1.628584266 | up | 0.002646192 | 0.315658588 |
| DYX1C1-CCPG1 | DYX1C1-CCPG1 readthrough (NMD candidate) | 1.628475147 | 1.628475147 | up | 0.00517298 | 0.394640262 |
| CCPG1 | cell cycle progression 1 | 1.628475147 | 1.628475147 | up | 0.00517298 | 0.394640262 |
| SCUBE3 | signal peptide, CUB domain, EGF-like 3 | 1.628087648 | 1.628087648 | up | 0.040860377 | 0.623333169 |
| MYH9 | myosin, heavy chain 9, non-muscle | 1.628027462 | 1.628027462 | up | 0.003162819 | 0.331128625 |
| BACE1 | beta-site APP-cleaving enzyme 1 | 1.627591182 | 1.627591182 | up | 0.014418106 | 0.51506237 |
| WDR1 | WD repeat domain 1 | 1.627508452 | 1.627508452 | up | 0.000147144 | 0.078274868 |
| SLC9A8 | solute carrier family 9, subfamily A (NHE8, cation proton antiporter 8), member 8 | 1.627459568 | 1.627459568 | up | 0.047431444 | 0.627322764 |
| GCC2 | GRIP and coiled-coil domain containing 2 | 1.627339246 | 1.627339246 | up | 0.007457162 | 0.438278902 |
| MYL9 | myosin light chain 9 | 1.62693698 | 1.62693698 | up | 0.000132285 | 0.074677857 |
| FRMPD2B | FERM and PDZ domain containing 2B, pseudogene | 1.626534815 | 1.626534815 | up | 0.035206606 | 0.600433528 |
| FRMPD2 | FERM and PDZ domain containing 2 | 1.626534815 | 1.626534815 | up | 0.035206606 | 0.600433528 |
| FERMT2 | fermitin family member 2 | 1.626482202 | 1.626482202 | up | 0.00188545 | 0.272870376 |
| NQO1 | NAD(P)H dehydrogenase, quinone 1 | 1.626294314 | 1.626294314 | up | 1.49343E-05 | 0.015923666 |
| THOC2 | THO complex 2 | 1.626290557 | 1.626290557 | up | 0.012571003 | 0.504821772 |
| POLDIP3 | polymerase (DNA-directed), delta interacting protein 3 | 1.625922361 | 1.625922361 | up | 0.017125806 | 0.530571823 |
| JMY | junction mediating and regulatory protein, p53 cofactor | 1.625839716 | 1.625839716 | up | 0.03262948 | 0.590424587 |
| WDR92 | WD repeat domain 92 | 1.625753319 | 1.625753319 | up | 0.034497402 | 0.596656508 |
| TRAPPC8 | trafficking protein particle complex 8 | 1.625362712 | 1.625362712 | up | 0.04612677 | 0.627185789 |
| ANKRD2 | ankyrin repeat domain 2 (stretch responsive muscle) | 1.625250054 | 1.625250054 | up | 0.033668723 | 0.593014207 |
| C1orf131 | chromosome 1 open reading frame 131 | 1.625216259 | 1.625216259 | up | 0.004831984 | 0.381949914 |
| TOM1 | target of myb1 membrane trafficking protein | 1.625193728 | 1.625193728 | up | 0.044316665 | 0.623333169 |
| ALG11 | ALG11, alpha-1,2-mannosyltransferase | 1.624874585 | 1.624874585 | up | 0.005527779 | 0.401886731 |
| UTP14C | UTP14, U3 small nucleolar ribonucleoprotein, homolog C (yeast) | 1.624874585 | 1.624874585 | up | 0.005527779 | 0.401886731 |
| WWTR1 | WW domain containing transcription regulator 1 | 1.624600548 | 1.624600548 | up | 0.0222042 | 0.550353494 |
| CD163L1 | CD163 molecule like 1 | 1.624574272 | 1.624574272 | up | 0.002191223 | 0.288675084 |
| PSIP1 | PC4 and SFRS1 interacting protein 1 | 1.623887515 | 1.623887515 | up | 0.003710098 | 0.349787905 |
| CTGF | connective tissue growth factor | 1.623613644 | 1.623613644 | up | 0.003154718 | 0.331128625 |
| FAM49A | family with sequence similarity 49 member A | 1.623384828 | 1.623384828 | up | 0.007865463 | 0.446814625 |
| CD2AP | CD2-associated protein | 1.622893546 | 1.622893546 | up | 0.022702966 | 0.551668068 |
| SEMA5B | sema domain, seven thrombospondin repeats (type 1 and type 1-like), transmembrane domain (TM) and short cytoplasmic domain, | 1.622792308 | 1.622792308 | up | 0.040511088 | 0.623005985 |
| DYNC1H1 | dynein, cytoplasmic 1, heavy chain 1 | 1.622668581 | 1.622668581 | up | 0.026957267 | 0.568317266 |
| SIPA1L2 | signal-induced proliferation-associated 1 like 2 | 1.622274967 | 1.622274967 | up | 0.002245868 | 0.293716231 |
| SHISA4 | shisa family member 4 | 1.622080069 | 1.622080069 | up | 0.000964573 | 0.21697376 |
| FBXO10 | F-box protein 10 | 1.62199762 | 1.62199762 | up | 0.046313278 | 0.627185789 |
| GDA | guanine deaminase | 1.62199762 | 1.62199762 | up | 0.004226943 | 0.361725211 |
| ZNF621 | zinc finger protein 621 | 1.621941407 | 1.621941407 | up | 0.02318844 | 0.55642591 |
| KRTAP10-8 | keratin associated protein 10-8 | 1.621896437 | 1.621896437 | up | 0.007108759 | 0.434730278 |
| ZNF57 | zinc finger protein 57 | 1.621506757 | 1.621506757 | up | 0.01573622 | 0.522063677 |
| DIS3L2 | DIS3 like 3'-5' exoribonuclease 2 | 1.621386874 | 1.621386874 | up | 0.005969305 | 0.413214927 |
| GRAMD4 | GRAM domain containing 4 | 1.62122205 | 1.62122205 | up | 0.02132624 | 0.549146 |
| SLC16A7 | solute carrier family 16 (monocarboxylate transporter), member 7 | 1.620948628 | 1.620948628 | up | 0.025070774 | 0.565551322 |
| LRP10 | LDL receptor related protein 10 | 1.620944883 | 1.620944883 | up | 0.025713995 | 0.566329497 |
| PRKAA2 | protein kinase, AMP-activated, alpha 2 catalytic subunit | 1.620738911 | 1.620738911 | up | 0.017115936 | 0.530571823 |
| SRF | serum response factor | 1.620420643 | 1.620420643 | up | 0.002659686 | 0.315760691 |
| FXYD3 | FXYD domain containing ion transport regulator 3 | 1.620342022 | 1.620342022 | up | 0.000757164 | 0.195840366 |

| | | | | | | |
|---|---|---|---|---|---|---|
| TRAK1 | trafficking protein, kinesin binding 1 | 1.62033079 | 1.62033079 | up | 0.025206784 | 0.565656951 |
| RAD21 | RAD21 cohesin complex component | 1.620267148 | 1.620267148 | up | 0.000476018 | 0.154312327 |
| KRTDAP | keratinocyte differentiation-associated protein | 1.620225968 | 1.620225968 | up | 0.047103464 | 0.627322764 |
| GDAP1 | ganglioside induced differentiation associated protein 1 | 1.620158586 | 1.620158586 | up | 0.03092086 | 0.583508935 |
| SMAD3 | SMAD family member 3 | 1.620102437 | 1.620102437 | up | 0.00375219 | 0.350843151 |
| MDK | midkine (neurite growth-promoting factor 2) | 1.619866631 | 1.619866631 | up | 0.000433343 | 0.145799553 |
| LPP | LIM domain containing preferred translocation partner in lipoma | 1.619552275 | 1.619552275 | up | 0.024637849 | 0.563613185 |
| ANXA9 | annexin A9 | 1.619492405 | 1.619492405 | up | 0.012804289 | 0.5057776 |
| SNHG12 | small nucleolar RNA host gene 12 | 1.619447504 | 1.619447504 | up | 0.004017254 | 0.35778104 |
| SNORA16A | small nucleolar RNA, H/ACA box 16A | 1.619447504 | 1.619447504 | up | 0.004017254 | 0.35778104 |
| SLC17A5 | solute carrier family 17 (acidic sugar transporter), member 5 | 1.619350222 | 1.619350222 | up | 0.025139149 | 0.565551322 |
| KLHL9 | kelch like family member 9 | 1.619237981 | 1.619237981 | up | 0.029876441 | 0.579152964 |
| HMGB1 | high mobility group box 1 | 1.619103302 | 1.619103302 | up | 0.009672471 | 0.476541686 |
| AMDHD2 | amidohydrolase domain containing 2 | 1.618819017 | 1.618819017 | up | 0.014972923 | 0.516640194 |
| ELMOD3 | ELMO/CED-12 domain containing 3 | 1.618579658 | 1.618579658 | up | 0.010090681 | 0.480015301 |
| MSTO1 | misato 1, mitochondrial distribution and morphology regulator | 1.61841138 | 1.61841138 | up | 0.016364526 | 0.524267534 |
| MSTO2P | misato family member 2, pseudogene | 1.61841138 | 1.61841138 | up | 0.016364526 | 0.524267534 |
| CEP70 | centrosomal protein 70kDa | 1.618329117 | 1.618329117 | up | 0.005903958 | 0.41207323 |
| PCK2 | phosphoenolpyruvate carboxykinase 2 (mitochondrial) | 1.617798247 | 1.617798247 | up | 0.019079001 | 0.541879415 |
| PEX1 | peroxisomal biogenesis factor 1 | 1.617260077 | 1.617260077 | up | 0.043039784 | 0.623333169 |
| RAB11B | RAB11B, member RAS oncogene family | 1.617233921 | 1.617233921 | up | 0.019225588 | 0.542909083 |
| TNS3 | tensin 3 | 1.617140509 | 1.617140509 | up | 0.028351898 | 0.57421459 |
| VPS52 | VPS52 GARP complex subunit | 1.617035893 | 1.617035893 | up | 0.01211766 | 0.499488693 |
| ZBTB6 | zinc finger and BTB domain containing 6 | 1.616946228 | 1.616946228 | up | 0.043084444 | 0.623333169 |
| BPHL | biphenyl hydrolase-like (serine hydrolase) | 1.616867775 | 1.616867775 | up | 0.013110992 | 0.508746116 |
| PRRC2C | proline-rich coiled-coil 2C | 1.616759442 | 1.616759442 | up | 0.026446826 | 0.568317266 |
| SLC1A4 | solute carrier family 1 (glutamate/neutral amino acid transporter), member 4 | 1.616729558 | 1.616729558 | up | 0.044665889 | 0.623333169 |
| PODN | podocan | 1.616576412 | 1.616576412 | up | 0.025896251 | 0.566523191 |
| ASB16 | ankyrin repeat and SOCS box containing 16 | 1.616550267 | 1.616550267 | up | 0.026973538 | 0.568317266 |
| EPB41L4A | erythrocyte membrane protein band 4.1 like 4A | 1.616385934 | 1.616385934 | up | 0.020803628 | 0.549146 |
| INTS6 | integrator complex subunit 6 | 1.616191744 | 1.616191744 | up | 0.025141894 | 0.565551322 |
| TTC14 | tetratricopeptide repeat domain 14 | 1.616049851 | 1.616049851 | up | 0.0245262 | 0.563613185 |
| WDR81 | WD repeat domain 81 | 1.616005045 | 1.616005045 | up | 0.044806126 | 0.623333169 |
| PCM1 | pericentriolar material 1 | 1.615986377 | 1.615986377 | up | 0.039939321 | 0.620691429 |
| AMER1 | APC membrane recruitment protein 1 | 1.615975176 | 1.615975176 | up | 0.043552753 | 0.623333169 |
| RHOBTB3 | Rho-related BTB domain containing 3 | 1.615642911 | 1.615642911 | up | 0.003274949 | 0.336368557 |
| ACOT13 | acyl-CoA thioesterase 13 | 1.615530927 | 1.615530927 | up | 0.00026969 | 0.122081173 |
| RBAK | RB-associated KRAB zinc finger | 1.615501066 | 1.615501066 | up | 0.020842704 | 0.549146 |
| CPNE8 | copine VIII | 1.615482403 | 1.615482403 | up | 0.04239495 | 0.623333169 |
| KLHL8 | kelch like family member 8 | 1.615441345 | 1.615441345 | up | 0.047990579 | 0.627854743 |
| TUFT1 | tuftelin 1 | 1.615243537 | 1.615243537 | up | 0.0098518 | 0.477233577 |
| CSNK1D | casein kinase 1, delta | 1.615213681 | 1.615213681 | up | 0.006211465 | 0.414771056 |
| SH3GL1 | SH3-domain GRB2-like 1 | 1.615206217 | 1.615206217 | up | 0.027973054 | 0.572828574 |
| UNKL | unkempt family zinc finger-like | 1.615079337 | 1.615079337 | up | 0.046685877 | 0.627185789 |
| DCP1B | decapping mRNA 1B | 1.615000975 | 1.615000975 | up | 0.035877768 | 0.603048422 |
| MBD6 | methyl-CpG binding domain protein 6 | 1.614918885 | 1.614918885 | up | 0.026430119 | 0.568317266 |
| SYNPO2 | synaptopodin 2 | 1.614639065 | 1.614639065 | up | 0.038214344 | 0.612253826 |
| OPN3 | opsin 3 | 1.614176536 | 1.614176536 | up | 0.000331212 | 0.128711653 |
| FANCD2P2 | Fanconi anemia complementation group D2 pseudogene 2 | 1.614098218 | 1.614098218 | up | 0.021895006 | 0.550085548 |
| FANCD2 | Fanconi anemia complementation group D2 | 1.614098218 | 1.614098218 | up | 0.021895006 | 0.550085548 |
| FAM219B | family with sequence similarity 219 member B | 1.613915489 | 1.613915489 | up | 0.038083383 | 0.612253826 |
| ZSWIM6 | zinc finger, SWIM-type containing 6 | 1.613609746 | 1.613609746 | up | 0.004128437 | 0.358673662 |
| TMCO4 | transmembrane and coiled-coil domains 4 | 1.613356246 | 1.613356246 | up | 0.023343405 | 0.556498532 |
| TPP1 | tripeptidyl peptidase I | 1.613169874 | 1.613169874 | up | 0.049802446 | 0.630405293 |

| | | | | | | |
|---|---|---|---|---|---|---|
| KIAA1033 | KIAA1033 | 1.613080424 | 1.613080424 | up | 0.008139513 | 0.454504307 |
| FOXRED2 | FAD-dependent oxidoreductase domain containing 2 | 1.613028247 | 1.613028247 | up | 0.006481144 | 0.423101578 |
| USP48 | ubiquitin specific peptidase 48 | 1.612782291 | 1.612782291 | up | 0.028750529 | 0.574350948 |
| TNKS2 | tankyrase, TRF1-interacting ankyrin-related ADP-ribose polymerase 2 | 1.612726397 | 1.612726397 | up | 0.027294576 | 0.56844604 |
| RAB2A | RAB2A, member RAS oncogene family | 1.612659327 | 1.612659327 | up | 0.028947346 | 0.574350948 |
| WNT5A | wingless-type MMTV integration site family member 5A | 1.612543824 | 1.612543824 | up | 0.021372963 | 0.549146 |
| ARL1 | ADP ribosylation factor like GTPase 1 | 1.612525195 | 1.612525195 | up | 0.0157389 | 0.522063677 |
| CRISPLD1 | cysteine-rich secretory protein LCCL domain containing 1 | 1.612480487 | 1.612480487 | up | 0.044037173 | 0.623333169 |
| ZNF33A | zinc finger protein 33A | 1.61220854 | 1.61220854 | up | 0.049138305 | 0.629664508 |
| ZNF33B | zinc finger protein 33B | 1.61220854 | 1.61220854 | up | 0.049138305 | 0.629664508 |
| F8 | coagulation factor VIII, procoagulant component | 1.612148941 | 1.612148941 | up | 0.027271721 | 0.568317266 |
| LAIR2 | leukocyte-associated immunoglobulin-like receptor 2 | 1.612014852 | 1.612014852 | up | 0.026225311 | 0.568317266 |
| OPRL1 | opiate receptor-like 1 | 1.61170202 | 1.61170202 | up | 0.041419767 | 0.623333169 |
| GNG4 | guanine nucleotide binding protein (G protein), gamma 4 | 1.611586586 | 1.611586586 | up | 0.008335903 | 0.460228374 |
| SHISA5 | shisa family member 5 | 1.611333404 | 1.611333404 | up | 0.038353117 | 0.612731512 |
| ATF3 | activating transcription factor 3 | 1.611169602 | 1.611169602 | up | 0.009443105 | 0.473538623 |
| PADI2 | peptidyl arginine deiminase, type II | 1.611165879 | 1.611165879 | up | 0.042104152 | 0.623333169 |
| ABR | active BCR-related | 1.610622474 | 1.610622474 | up | 0.017704223 | 0.532411571 |
| LAMP2 | lysosomal-associated membrane protein 2 | 1.610082971 | 1.610082971 | up | 0.001503151 | 0.251793822 |
| PRR12 | proline rich 12 | 1.610004852 | 1.610004852 | up | 0.044918272 | 0.623333169 |
| CKMT1A | creatine kinase, mitochondrial 1A | 1.609870941 | 1.609870941 | up | 0.001005187 | 0.218262407 |
| CKMT1B | creatine kinase, mitochondrial 1B | 1.609870941 | 1.609870941 | up | 0.001005187 | 0.218262407 |
| GRAMD2 | GRAM domain containing 2 | 1.609469275 | 1.609469275 | up | 0.006268593 | 0.416829693 |
| RASL10B | RAS-like, family 10, member B | 1.609376311 | 1.609376311 | up | 0.009426186 | 0.473293115 |
| ZNF529 | zinc finger protein 529 | 1.609075145 | 1.609075145 | up | 0.026084816 | 0.567622316 |
| CEACAM19 | carcinoembryonic antigen-related cell adhesion molecule 19 | 1.609075145 | 1.609075145 | up | 0.01231576 | 0.503186668 |
| NAB2 | NGFI-A binding protein 2 (EGR1 binding protein 2) | 1.60885953 | 1.60885953 | up | 0.014218242 | 0.514636149 |
| C1QL1 | complement component 1, q subcomponent-like 1 | 1.608666244 | 1.608666244 | up | 0.025650304 | 0.566329497 |
| HSD17B1 | hydroxysteroid (17-beta) dehydrogenase 1 | 1.608432102 | 1.608432102 | up | 0.02295955 | 0.554273475 |
| TAP2 | transporter 2, ATP-binding cassette, sub-family B (MDR/TAP) | 1.608413521 | 1.608413521 | up | 0.041593939 | 0.623333169 |
| ALPP | alkaline phosphatase, placental | 1.608183131 | 1.608183131 | up | 0.000177284 | 0.089266768 |
| BDH2 | 3-hydroxybutyrate dehydrogenase, type 2 | 1.608149691 | 1.608149691 | up | 0.025395658 | 0.565656951 |
| GEMIN4 | gem nuclear organelle associated protein 4 | 1.608012219 | 1.608012219 | up | 0.032293208 | 0.589996854 |
| HTRA1 | HtrA serine peptidase 1 | 1.607677877 | 1.607677877 | up | 0.014454924 | 0.51506237 |
| DNASE1L1 | deoxyribonuclease I-like 1 | 1.607625874 | 1.607625874 | up | 0.015510367 | 0.52192494 |
| CDC14B | cell division cycle 14B | 1.607507018 | 1.607507018 | up | 0.010075696 | 0.480015301 |
| KISS1R | KISS1 receptor | 1.607492161 | 1.607492161 | up | 0.047672965 | 0.627854743 |
| ATXN3 | ataxin 3 | 1.607265617 | 1.607265617 | up | 0.015795195 | 0.522063677 |
| ZNF561 | zinc finger protein 561 | 1.607031679 | 1.607031679 | up | 0.046593787 | 0.627185789 |
| CPA4 | carboxypeptidase A4 | 1.60653421 | 1.60653421 | up | 0.000419612 | 0.144894892 |
| NUDT16P1 | nudix hydrolase 16 pseudogene 1 | 1.606482245 | 1.606482245 | up | 0.040950319 | 0.623333169 |
| PKDCC | protein kinase domain containing, cytoplasmic | 1.606452551 | 1.606452551 | up | 0.011908486 | 0.495320532 |
| SAMD4A | sterile alpha motif domain containing 4A | 1.606445127 | 1.606445127 | up | 0.02217702 | 0.550353494 |
| SPINK2 | serine peptidase inhibitor, Kazal type 2 (acrosin-trypsin inhibitor) | 1.606163065 | 1.606163065 | up | 0.042049614 | 0.623333169 |
| KRTAP10-12 | keratin associated protein 10-12 | 1.6058625 | 1.6058625 | up | 0.029632009 | 0.57728016 |
| C18orf32 | chromosome 18 open reading frame 32 | 1.605747484 | 1.605747484 | up | 0.012006049 | 0.497492736 |
| RPL17-C18orf32 | RPL17-C18orf32 readthrough | 1.605747484 | 1.605747484 | up | 0.012006049 | 0.497492736 |
| AGGF1 | angiogenic factor with G-patch and FHA domains 1 | 1.605710384 | 1.605710384 | up | 0.020225283 | 0.549146 |
| MED20 | mediator complex subunit 20 | 1.605625056 | 1.605625056 | up | 0.033169992 | 0.59236436 |
| DNAL4 | dynein, axonemal, light chain 4 | 1.605506348 | 1.605506348 | up | 0.030336315 | 0.580548881 |
| SCNN1A | sodium channel, non voltage gated 1 alpha subunit | 1.605020476 | 1.605020476 | up | 0.021445728 | 0.549146 |
| ATXN7L3B | ataxin 7-like 3B | 1.604968559 | 1.604968559 | up | 0.04100065 | 0.623333169 |
| DDR1 | discoidin domain receptor tyrosine kinase 1 | 1.604797988 | 1.604797988 | up | 0.00368315 | 0.349787905 |
| MTMR1 | myotubularin related protein 1 | 1.604768325 | 1.604768325 | up | 0.033525258 | 0.593014207 |

| | | | | | | |
|---|---|---|---|---|---|---|
| CLU | clusterin | 1.604675633 | 1.604675633 | up | 0.001454918 | 0.250769966 |
| TTC39A | tetratricopeptide repeat domain 39A | 1.604553287 | 1.604553287 | up | 0.049627468 | 0.630325871 |
| NHLRC4 | NHL repeat containing 4 | 1.604360519 | 1.604360519 | up | 0.037500581 | 0.609325627 |
| SLC41A3 | solute carrier family 41, member 3 | 1.604008406 | 1.604008406 | up | 0.012956486 | 0.507494586 |
| CCDC6 | coiled-coil domain containing 6 | 1.603837937 | 1.603837937 | up | 0.001074229 | 0.222563245 |
| CERS6 | ceramide synthase 6 | 1.603678602 | 1.603678602 | up | 0.046320439 | 0.627185789 |
| BCAR1 | breast cancer anti-estrogen resistance 1 | 1.60364896 | 1.60364896 | up | 0.00678833 | 0.430310156 |
| LGALS3BP | lectin, galactoside-binding, soluble, 3 binding protein | 1.603515578 | 1.603515578 | up | 0.002865334 | 0.32135011 |
| CUX1 | cut-like homeobox 1 | 1.603167354 | 1.603167354 | up | 0.030771406 | 0.582643771 |
| ZCCHC11 | zinc finger, CCHC domain containing 11 | 1.602852536 | 1.602852536 | up | 0.048664847 | 0.62943979 |
| PIAS4 | protein inhibitor of activated STAT 4 | 1.602626646 | 1.602626646 | up | 0.024669262 | 0.563613185 |
| TPR | translocated promoter region, nuclear basket protein | 1.602456324 | 1.602456324 | up | 0.010417047 | 0.486437775 |
| SART1 | squamous cell carcinoma antigen recognized by T-cells 1 | 1.602437812 | 1.602437812 | up | 0.03647668 | 0.603912936 |
| FGF2 | fibroblast growth factor 2 (basic) | 1.602323041 | 1.602323041 | up | 0.032904317 | 0.590894404 |
| ZNF275 | zinc finger protein 275 | 1.602071314 | 1.602071314 | up | 0.027369495 | 0.56844604 |
| NCBP1 | nuclear cap binding protein subunit 1 | 1.60164569 | 1.60164569 | up | 0.043399774 | 0.623333169 |
| LARP6 | La ribonucleoprotein domain family member 6 | 1.601431071 | 1.601431071 | up | 0.003141516 | 0.330869322 |
| C1R | complement component 1, r subcomponent | 1.601257176 | 1.601257176 | up | 0.002167052 | 0.288675084 |
| H1F0 | H1 histone family member 0 | 1.601142489 | 1.601142489 | up | 0.007451722 | 0.438278902 |
| FBLN1 | fibulin 1 | 1.601131391 | 1.601131391 | up | 0.006734117 | 0.428252854 |
| FAF2 | Fas associated factor family member 2 | 1.60065424 | 1.60065424 | up | 0.034475617 | 0.596656508 |
| GNA12 | guanine nucleotide binding protein (G protein) alpha 12 | 1.600595068 | 1.600595068 | up | 0.040119525 | 0.621238987 |
| ASNS | asparagine synthetase (glutamine-hydrolyzing) | 1.600558087 | 1.600558087 | up | 0.001923209 | 0.273936058 |
| IL17RC | interleukin 17 receptor C | 1.600524805 | 1.600524805 | up | 0.038541025 | 0.61336314 |
| PRRC2B | proline-rich coiled-coil 2B | 1.600513711 | 1.600513711 | up | 0.045285931 | 0.624555255 |
| BCL2L11 | BCL2-like 11 (apoptosis facilitator) | 1.600358404 | 1.600358404 | up | 0.030390004 | 0.581011416 |
| SYBU | syntabulin (syntaxin-interacting) | 1.599999775 | 1.599999775 | up | 0.011071607 | 0.489992559 |
| CLIC3 | chloride intracellular channel 3 | 1.599984988 | 1.599984988 | up | 0.001410563 | 0.248565272 |
| PSMA1 | proteasome subunit alpha 1 | 1.59961166 | 1.59961166 | up | 0.033103672 | 0.592343125 |
| ZNF836 | zinc finger protein 836 | 1.599604268 | 1.599604268 | up | 0.044890241 | 0.623333169 |
| FAM13B | family with sequence similarity 13 member B | 1.599153436 | 1.599153436 | up | 0.042261628 | 0.623333169 |
| RRAS | related RAS viral (r-ras) oncogene homolog | 1.599149741 | 1.599149741 | up | 0.004394047 | 0.367259039 |
| BAIAP2 | BAI1-associated protein 2 | 1.599083235 | 1.599083235 | up | 0.035990543 | 0.603119509 |
| MAGI3 | membrane associated guanylate kinase, WW and PDZ domain containing 3 | 1.598772913 | 1.598772913 | up | 0.028276288 | 0.57421459 |
| CTHRC1 | collagen triple helix repeat containing 1 | 1.598529131 | 1.598529131 | up | 0.029930501 | 0.579152964 |
| GDI1 | GDP dissociation inhibitor 1 | 1.598322315 | 1.598322315 | up | 0.046613558 | 0.627185789 |
| FNDC3B | fibronectin type III domain containing 3B | 1.598314929 | 1.598314929 | up | 0.041945325 | 0.623333169 |
| MFSD6 | major facilitator superfamily domain containing 6 | 1.598307543 | 1.598307543 | up | 0.003006909 | 0.326391956 |
| CYP1B1 | cytochrome P450, family 1, subfamily B, polypeptide 1 | 1.598174606 | 1.598174606 | up | 0.034370173 | 0.596552856 |
| EPAS1 | endothelial PAS domain protein 1 | 1.598141373 | 1.598141373 | up | 0.035384448 | 0.600698736 |
| C6orf141 | chromosome 6 open reading frame 141 | 1.598126603 | 1.598126603 | up | 0.041317171 | 0.623333169 |
| LOC100996720 | uncharacterized LOC100996720 | 1.598111833 | 1.598111833 | up | 0.032990851 | 0.591925639 |
| UBA6 | ubiquitin-like modifier activating enzyme 6 | 1.598049063 | 1.598049063 | up | 0.006987208 | 0.432531861 |
| SUPT20H | SPT20 homolog, SAGA complex component | 1.598019525 | 1.598019525 | up | 0.025112542 | 0.565551322 |
| STRA6 | stimulated by retinoic acid 6 | 1.597982604 | 1.597982604 | up | 0.018013828 | 0.535584855 |
| GPR162 | G protein-coupled receptor 162 | 1.597927223 | 1.597927223 | up | 0.032527617 | 0.590424587 |
| B4GALT5 | UDP-Gal:betaGlcNAc beta 1,4- galactosyltransferase, polypeptide 5 | 1.597661421 | 1.597661421 | up | 0.00100567 | 0.218262407 |
| PMEL | premelanosome protein | 1.597569139 | 1.597569139 | up | 0.009862497 | 0.477233577 |
| PDS5A | PDS5 cohesin associated factor A | 1.597451026 | 1.597451026 | up | 0.015541755 | 0.52192494 |
| DDX6 | DEAD (Asp-Glu-Ala-Asp) box helicase 6 | 1.597259111 | 1.597259111 | up | 0.046079131 | 0.627185789 |
| LY6E | lymphocyte antigen 6 complex, locus E | 1.597211136 | 1.597211136 | up | 0.011089932 | 0.489992559 |
| INPP5D | inositol polyphosphate-5-phosphatase D | 1.597141021 | 1.597141021 | up | 0.024181368 | 0.562764151 |
| LIF | leukemia inhibitory factor | 1.59711888 | 1.59711888 | up | 0.033457604 | 0.593014207 |
| DACT3 | dishevelled-binding antagonist of beta-catenin 3 | 1.59700449 | 1.59700449 | up | 0.04288421 | 0.623333169 |

| | | | | | | |
|---|---|---|---|---|---|---|
| FBXL4 | F-box and leucine-rich repeat protein 4 | 1.596960212 | 1.596960212 | up | 0.034679894 | 0.597999402 |
| PQLC3 | PQ loop repeat containing 3 | 1.596938074 | 1.596938074 | up | 0.012313684 | 0.503186668 |
| TRAPPC6A | trafficking protein particle complex 6A | 1.596886419 | 1.596886419 | up | 0.008904777 | 0.466623412 |
| PHF12 | PHD finger protein 12 | 1.596606035 | 1.596606035 | up | 0.029118044 | 0.57512885 |
| ACTA1 | actin, alpha 1, skeletal muscle | 1.59641791 | 1.59641791 | up | 0.033180831 | 0.59236436 |
| RBM26 | RNA binding motif protein 26 | 1.596414221 | 1.596414221 | up | 0.030034399 | 0.579152964 |
| TCEAL6 | transcription elongation factor A (SII)-like 6 | 1.596299882 | 1.596299882 | up | 0.010019121 | 0.479491277 |
| TCEAL3 | transcription elongation factor A (SII)-like 3 | 1.596299882 | 1.596299882 | up | 0.010019121 | 0.479491277 |
| SAMD12 | sterile alpha motif domain containing 12 | 1.596285129 | 1.596285129 | up | 0.043695472 | 0.623333169 |
| PIGP | phosphatidylinositol glycan anchor biosynthesis class P | 1.596237183 | 1.596237183 | up | 0.004269224 | 0.362398259 |
| FAM46A | family with sequence similarity 46 member A | 1.595820483 | 1.595820483 | up | 0.025119327 | 0.565551322 |
| G6PC3 | glucose 6 phosphatase, catalytic, 3 | 1.595437068 | 1.595437068 | up | 0.021231316 | 0.549146 |
| FAM149B1 | family with sequence similarity 149 member B1 | 1.595367031 | 1.595367031 | up | 0.040629736 | 0.623259064 |
| ZNF280C | zinc finger protein 280C | 1.595079542 | 1.595079542 | up | 0.0323288 | 0.589996854 |
| ENC1 | ectodermal-neural cortex 1 (with BTB domain) | 1.595079542 | 1.595079542 | up | 0.015519454 | 0.52192494 |
| HNRNPLL | heterogeneous nuclear ribonucleoprotein L-like | 1.594961613 | 1.594961613 | up | 0.016489 | 0.52581211 |
| GJC2 | gap junction protein gamma 2 | 1.594854748 | 1.594854748 | up | 0.043523014 | 0.623333169 |
| ZNF84 | zinc finger protein 84 | 1.59440157 | 1.59440157 | up | 0.049016636 | 0.62943979 |
| KIAA1549 | KIAA1549 | 1.594342629 | 1.594342629 | up | 0.035376278 | 0.600698736 |
| BRAF | B-Raf proto-oncogene, serine/threonine kinase | 1.594342629 | 1.594342629 | up | 0.035376278 | 0.600698736 |
| MARCH5 | membrane associated ring-CH-type finger 5 | 1.594213705 | 1.594213705 | up | 0.003311623 | 0.338252205 |
| STK16 | serine/threonine kinase 16 | 1.593705474 | 1.593705474 | up | 0.018869888 | 0.539906054 |
| EEF1A2 | eukaryotic translation elongation factor 1 alpha 2 | 1.593580283 | 1.593580283 | up | 0.042450406 | 0.623333169 |
| NUP62CL | nucleoporin 62kDa C-terminal like | 1.593300479 | 1.593300479 | up | 0.0274024 | 0.568462255 |
| ESYT2 | extended synaptotagmin-like protein 2 | 1.593256304 | 1.593256304 | up | 0.005955032 | 0.413214927 |
| C6orf106 | chromosome 6 open reading frame 106 | 1.593079616 | 1.593079616 | up | 0.028325916 | 0.57421459 |
| TMEM184C | transmembrane protein 184C | 1.593017044 | 1.593017044 | up | 0.016995874 | 0.530571823 |
| AP1AR | adaptor-related protein complex 1 associated regulatory protein | 1.59299496 | 1.59299496 | up | 0.047238705 | 0.627322764 |
| CCSER2 | coiled-coil serine rich protein 2 | 1.592972877 | 1.592972877 | up | 0.009756835 | 0.477233577 |
| ASRGL1 | asparaginase like 1 | 1.592943432 | 1.592943432 | up | 0.0165364 | 0.52581211 |
| INAFM1 | InaF-motif containing 1 | 1.59292503 | 1.59292503 | up | 0.011838423 | 0.495320532 |
| KIAA0586 | KIAA0586 | 1.592700539 | 1.592700539 | up | 0.044237199 | 0.623333169 |
| RPIA | ribose 5-phosphate isomerase A | 1.592498157 | 1.592498157 | up | 0.024458686 | 0.563613185 |
| UEVLD | UEV and lactate/malate dehyrogenase domains | 1.592424569 | 1.592424569 | up | 0.034020438 | 0.595139711 |
| AHR | aryl hydrocarbon receptor | 1.592299479 | 1.592299479 | up | 0.034161642 | 0.595139711 |
| CAST | calpastatin | 1.592163362 | 1.592163362 | up | 0.006167883 | 0.414771056 |
| MAN2B2 | mannosidase, alpha, class 2B, member 2 | 1.592159683 | 1.592159683 | up | 0.03647401 | 0.603912936 |
| UTP11L | UTP11-like, U3 small nucleolar ribonucleoprotein (yeast) | 1.592148647 | 1.592148647 | up | 0.006417273 | 0.419627816 |
| FAM213A | family with sequence similarity 213 member A | 1.592016221 | 1.592016221 | up | 0.004581954 | 0.374624543 |
| SLC43A2 | solute carrier family 43 (amino acid system L transporter), member 2 | 1.591777147 | 1.591777147 | up | 0.016510365 | 0.52581211 |
| BTRC | beta-transducin repeat containing E3 ubiquitin protein ligase | 1.59174037 | 1.59174037 | up | 0.029715907 | 0.577947966 |
| CLN3 | ceroid-lipofuscinosis, neuronal 3 | 1.591733014 | 1.591733014 | up | 0.035347872 | 0.600698736 |
| PLEKHG4 | pleckstrin homology and RhoGEF domain containing G4 | 1.591674173 | 1.591674173 | up | 0.027012609 | 0.568317266 |
| SSX2IP | synovial sarcoma, X breakpoint 2 interacting protein | 1.59143515 | 1.59143515 | up | 0.030043184 | 0.579152964 |
| GNPTG | N-acetylglucosamine-1-phosphate transferase, gamma subunit | 1.591431473 | 1.591431473 | up | 0.035184699 | 0.600433528 |
| SCAMP1 | secretory carrier membrane protein 1 | 1.591299107 | 1.591299107 | up | 0.007180135 | 0.434730278 |
| PROSER1 | proline and serine rich 1 | 1.590696245 | 1.590696245 | up | 0.014798642 | 0.515560792 |
| PLK1 | polo-like kinase 1 | 1.59056394 | 1.59056394 | up | 0.049653943 | 0.630325871 |
| C22orf29 | chromosome 22 open reading frame 29 | 1.590427972 | 1.590427972 | up | 0.013448159 | 0.509292857 |
| CAMKMT | calmodulin-lysine N-methyltransferase | 1.59026262 | 1.59026262 | up | 0.017618361 | 0.53179491 |
| SEC23A | Sec23 homolog A, COPII coat complex component | 1.590137699 | 1.590137699 | up | 0.003824543 | 0.351759623 |
| VPS37A | vacuolar protein sorting 37 homolog A (S. cerevisiae) | 1.590056874 | 1.590056874 | up | 0.047084018 | 0.627322764 |
| USPL1 | ubiquitin specific peptidase like 1 | 1.58986952 | 1.58986952 | up | 0.041200224 | 0.623333169 |
| ZFR | zinc finger RNA binding protein | 1.589807074 | 1.589807074 | up | 0.023145995 | 0.55642591 |

| | | | | | | |
|---|---|---|---|---|---|---|
| PNPO | pyridoxamine 5'-phosphate oxidase | 1.589748303 | 1.589748303 | up | 0.031186221 | 0.585954505 |
| RHOQ | ras homolog family member Q | 1.589542623 | 1.589542623 | up | 0.032568313 | 0.590424587 |
| CRBN | cereblon | 1.589472845 | 1.589472845 | up | 0.039375185 | 0.615814128 |
| ENPP5 | ectonucleotide pyrophosphatase/phosphodiesterase 5 (putative) | 1.588969797 | 1.588969797 | up | 0.037201098 | 0.607707429 |
| ZNF250 | zinc finger protein 250 | 1.588800926 | 1.588800926 | up | 0.03716244 | 0.607516377 |
| BMP1 | bone morphogenetic protein 1 | 1.58850361 | 1.58850361 | up | 0.037318667 | 0.608806191 |
| AGA | aspartylglucosaminidase | 1.588239376 | 1.588239376 | up | 0.0217696 | 0.550085548 |
| NEK1 | NIMA-related kinase 1 | 1.587846776 | 1.587846776 | up | 0.043430399 | 0.623333169 |
| ZNF644 | zinc finger protein 644 | 1.587839439 | 1.587839439 | up | 0.01573317 | 0.522063677 |
| ALDH6A1 | aldehyde dehydrogenase 6 family member A1 | 1.587795415 | 1.587795415 | up | 0.048586952 | 0.62943979 |
| RAD51 | RAD51 recombinase | 1.587718377 | 1.587718377 | up | 0.023130504 | 0.55642591 |
| PRR3 | proline rich 3 | 1.587487284 | 1.587487284 | up | 0.009867744 | 0.477233577 |
| PAIP1 | poly(A) binding protein interacting protein 1 | 1.587454274 | 1.587454274 | up | 0.001012755 | 0.218262407 |
| JUN | jun proto-oncogene | 1.587373584 | 1.587373584 | up | 0.03901821 | 0.613717043 |
| GSTM2P1 | glutathione S-transferase mu 2 (muscle) pseudogene 1 | 1.587362581 | 1.587362581 | up | 0.004145726 | 0.358673662 |
| CA2 | carbonic anhydrase II | 1.587285564 | 1.587285564 | up | 0.01272068 | 0.50529726 |
| ULK3 | unc-51 like kinase 3 | 1.587259892 | 1.587259892 | up | 0.046967858 | 0.627322764 |
| PEX12 | peroxisomal biogenesis factor 12 | 1.587091203 | 1.587091203 | up | 0.047280109 | 0.627322764 |
| GSPT2 | G1 to S phase transition 2 | 1.586988531 | 1.586988531 | up | 0.040215528 | 0.621304652 |
| ICAM2 | intercellular adhesion molecule 2 | 1.586988531 | 1.586988531 | up | 0.021780454 | 0.550085548 |
| LCE1D | late cornified envelope 1D | 1.586874867 | 1.586874867 | up | 0.024563724 | 0.563613185 |
| ARRDC4 | arrestin domain containing 4 | 1.586863868 | 1.586863868 | up | 0.005558911 | 0.401886731 |
| PTK7 | protein tyrosine kinase 7 (inactive) | 1.586856535 | 1.586856535 | up | 0.030071827 | 0.579152964 |
| NEDD9 | neural precursor cell expressed, developmentally down-regulated 9 | 1.586794207 | 1.586794207 | up | 0.044742131 | 0.623333169 |
| ZER1 | zyg-11 related, cell cycle regulator | 1.586629233 | 1.586629233 | up | 0.016622534 | 0.526872128 |
| SRBD1 | S1 RNA binding domain 1 | 1.586603572 | 1.586603572 | up | 0.039482696 | 0.616760444 |
| STX17 | syntaxin 17 | 1.586533923 | 1.586533923 | up | 0.043865215 | 0.623333169 |
| NMB | neuromedin B | 1.586281012 | 1.586281012 | up | 0.002015572 | 0.280363933 |
| FZD6 | frizzled class receptor 6 | 1.586204047 | 1.586204047 | up | 0.011146171 | 0.489992559 |
| USP1 | ubiquitin specific peptidase 1 | 1.586079445 | 1.586079445 | up | 0.001062586 | 0.221476037 |
| STK11IP | serine/threonine kinase 11 interacting protein | 1.586031806 | 1.586031806 | up | 0.036254723 | 0.603215176 |
| TMEM68 | transmembrane protein 68 | 1.585877903 | 1.585877903 | up | 0.037579565 | 0.609777239 |
| PTP4A3 | protein tyrosine phosphatase type IVA, member 3 | 1.585819278 | 1.585819278 | up | 0.027881582 | 0.572241118 |
| PPIP5K2 | diphosphoinositol pentakisphosphate kinase 2 | 1.585745999 | 1.585745999 | up | 0.044748257 | 0.623333169 |
| NUCB1 | nucleobindin 1 | 1.58572768 | 1.58572768 | up | 0.02904419 | 0.574540338 |
| RCC1 | regulator of chromosome condensation 1 | 1.585108617 | 1.585108617 | up | 0.009756015 | 0.477233577 |
| RGPD4 | RANBP2-like and GRIP domain containing 4 | 1.585009736 | 1.585009736 | up | 0.021761722 | 0.550085548 |
| CDC7 | cell division cycle 7 | 1.5846875 | 1.5846875 | up | 0.046727157 | 0.627185789 |
| MMP11 | matrix metallopeptidase 11 | 1.584548372 | 1.584548372 | up | 0.048257024 | 0.627892392 |
| EDN1 | endothelin 1 | 1.584530067 | 1.584530067 | up | 0.03601471 | 0.603119509 |
| PPAT | phosphoribosyl pyrophosphate amidotransferase | 1.584508101 | 1.584508101 | up | 0.021261758 | 0.549146 |
| IQSEC1 | IQ motif and Sec7 domain 1 | 1.584174986 | 1.584174986 | up | 0.031646956 | 0.589022431 |
| SMC1A | structural maintenance of chromosomes 1A | 1.583900493 | 1.583900493 | up | 0.012438978 | 0.503406041 |
| VCL | vinculin | 1.583900493 | 1.583900493 | up | 0.018779602 | 0.539772279 |
| SPOCD1 | SPOC domain containing 1 | 1.583863898 | 1.583863898 | up | 0.036662475 | 0.604603394 |
| REEP4 | receptor accessory protein 4 | 1.583834622 | 1.583834622 | up | 0.045306914 | 0.624555255 |
| CASC10 | cancer susceptibility candidate 10 | 1.583805347 | 1.583805347 | up | 0.034837509 | 0.59807176 |
| NRGN | neurogranin | 1.583472379 | 1.583472379 | up | 0.048291723 | 0.627892392 |
| PYCR1 | pyrroline-5-carboxylate reductase 1 | 1.583307751 | 1.583307751 | up | 0.006988194 | 0.432531861 |
| SDHA | succinate dehydrogenase complex subunit A, flavoprotein (Fp) | 1.583168745 | 1.583168745 | up | 0.010266768 | 0.485753032 |
| SDHAP2 | succinate dehydrogenase complex subunit A, flavoprotein pseudogene 2 | 1.583168745 | 1.583168745 | up | 0.010266768 | 0.485753032 |
| SDHAP1 | succinate dehydrogenase complex subunit A, flavoprotein pseudogene 1 | 1.583168745 | 1.583168745 | up | 0.010266768 | 0.485753032 |
| SAR1A | secretion associated, Ras related GTPase 1A | 1.583117535 | 1.583117535 | up | 0.013207826 | 0.509292857 |
| ZBTB21 | zinc finger and BTB domain containing 21 | 1.582857854 | 1.582857854 | up | 0.041796277 | 0.623333169 |

| | | | | | | |
|---|---|---|---|---|---|---|
| TRABD | TraB domain containing | 1.582857854 | 1.582857854 | up | 0.02650164 | 0.568317266 |
| CKLF-CMTM1 | CKLF-CMTM1 readthrough | 1.582784712 | 1.582784712 | up | 0.038832113 | 0.613615561 |
| CKLF | chemokine-like factor | 1.582784712 | 1.582784712 | up | 0.038832113 | 0.613615561 |
| SGOL2 | shugoshin-like 2 (S. pombe) | 1.582601872 | 1.582601872 | up | 0.025882575 | 0.566523191 |
| GGT5 | gamma-glutamyltransferase 5 | 1.582419054 | 1.582419054 | up | 0.049747355 | 0.630405293 |
| IVNS1ABP | influenza virus NS1A binding protein | 1.582283781 | 1.582283781 | up | 0.01187753 | 0.495320532 |
| PCGF5 | polycomb group ring finger 5 | 1.582272814 | 1.582272814 | up | 0.014541127 | 0.515525808 |
| ALDH1A3 | aldehyde dehydrogenase 1 family member A3 | 1.582185076 | 1.582185076 | up | 0.002964586 | 0.326391956 |
| RPL31 | ribosomal protein L31 | 1.582042513 | 1.582042513 | up | 0.047714947 | 0.627854743 |
| FABP3 | fatty acid binding protein 3, muscle and heart | 1.582027892 | 1.582027892 | up | 0.020921636 | 0.549146 |
| MALAT1 | metastasis associated lung adenocarcinoma transcript 1 (non-protein coding) | 1.581856104 | 1.581856104 | up | 0.01843715 | 0.537128348 |
| LYRM9 | LYR motif containing 9 | 1.581848795 | 1.581848795 | up | 0.037057938 | 0.606563707 |
| WDR89 | WD repeat domain 89 | 1.581717226 | 1.581717226 | up | 0.042143396 | 0.623333169 |
| EIF4G3 | eukaryotic translation initiation factor 4 gamma, 3 | 1.581666063 | 1.581666063 | up | 0.025394585 | 0.565656951 |
| MFN1 | mitofusin 1 | 1.58162221 | 1.58162221 | up | 0.02697148 | 0.568317266 |
| CTSA | cathepsin A | 1.58159663 | 1.58159663 | up | 0.000953379 | 0.216386548 |
| DUSP3 | dual specificity phosphatase 3 | 1.581501622 | 1.581501622 | up | 0.017734797 | 0.532517378 |
| GKAP1 | G kinase anchoring protein 1 | 1.581421235 | 1.581421235 | up | 0.034129207 | 0.595139711 |
| PRKACA | protein kinase, cAMP-dependent, alpha catalytic subunit | 1.581169139 | 1.581169139 | up | 0.011816594 | 0.495320532 |
| PRSS22 | protease, serine 22 | 1.580997445 | 1.580997445 | up | 0.038009103 | 0.612253826 |
| C1orf53 | chromosome 1 open reading frame 53 | 1.580971875 | 1.580971875 | up | 0.022564544 | 0.551250506 |
| ETFB | electron-transfer-flavoprotein, beta polypeptide | 1.580781939 | 1.580781939 | up | 0.002385266 | 0.300161496 |
| IQGAP1 | IQ motif containing GTPase activating protein 1 | 1.580628547 | 1.580628547 | up | 0.007867021 | 0.446814625 |
| SLC35F6 | solute carrier family 35 member F6 | 1.580475169 | 1.580475169 | up | 0.027850183 | 0.572241118 |
| HLA-DMB | major histocompatibility complex, class II, DM beta | 1.580475169 | 1.580475169 | up | 0.03901594 | 0.613717043 |
| ARRDC1-AS1 | ARRDC1 antisense RNA 1 | 1.580325458 | 1.580325458 | up | 0.032029384 | 0.589351082 |
| GPR160 | G protein-coupled receptor 160 | 1.580066235 | 1.580066235 | up | 0.031224614 | 0.585954505 |
| HLA-B | major histocompatibility complex, class I, B | 1.579777853 | 1.579777853 | up | 0.005494232 | 0.401886731 |
| RNASET2 | ribonuclease T2 | 1.579774203 | 1.579774203 | up | 0.01159286 | 0.495320532 |
| ATF6 | activating transcription factor 6 | 1.579252332 | 1.579252332 | up | 0.043234479 | 0.623333169 |
| TRPV6 | transient receptor potential cation channel, subfamily V, member 6 | 1.579230439 | 1.579230439 | up | 0.025641505 | 0.566329497 |
| IFI35 | interferon induced protein 35 | 1.579058955 | 1.579058955 | up | 0.013819461 | 0.511408446 |
| CHM | choroideremia (Rab escort protein 1) | 1.578927618 | 1.578927618 | up | 0.019882867 | 0.549146 |
| USF2 | upstream transcription factor 2, c-fos interacting | 1.578927618 | 1.578927618 | up | 0.045054076 | 0.623612409 |
| PLXNA2 | plexin A2 | 1.57876711 | 1.57876711 | up | 0.017321426 | 0.531030242 |
| TTC13 | tetratricopeptide repeat domain 13 | 1.578654034 | 1.578654034 | up | 0.045256218 | 0.624555255 |
| TRA2A | transformer 2 alpha homolog (Drosophila) | 1.578602971 | 1.578602971 | up | 0.01819429 | 0.536306821 |
| DCAF7 | DDB1 and CUL4 associated factor 7 | 1.578581087 | 1.578581087 | up | 0.020850899 | 0.549146 |
| LRG1 | leucine-rich alpha-2-glycoprotein 1 | 1.578515437 | 1.578515437 | up | 0.025293159 | 0.565656951 |
| ADIPOR2 | adiponectin receptor 2 | 1.578369558 | 1.578369558 | up | 0.037905508 | 0.611672854 |
| KRR1 | KRR1, small subunit (SSU) processome component, homolog (yeast) | 1.577986689 | 1.577986689 | up | 0.008156557 | 0.454504307 |
| BCLAF1 | BCL2-associated transcription factor 1 | 1.577939293 | 1.577939293 | up | 0.006643167 | 0.42771383 |
| CLCN3 | chloride channel, voltage-sensitive 3 | 1.577749722 | 1.577749722 | up | 0.024162876 | 0.562764151 |
| DECR1 | 2,4-dienoyl-CoA reductase 1, mitochondrial | 1.577705978 | 1.577705978 | up | 0.014914941 | 0.516117046 |
| ACTN4 | actinin, alpha 4 | 1.577582044 | 1.577582044 | up | 0.001256651 | 0.240074176 |
| UACA | uveal autoantigen with coiled-coil domains and ankyrin repeats | 1.577552884 | 1.577552884 | up | 0.038754697 | 0.613615561 |
| THOC6 | THO complex 6 | 1.577541949 | 1.577541949 | up | 0.023157006 | 0.55642591 |
| RBM25 | RNA binding motif protein 25 | 1.577541949 | 1.577541949 | up | 0.02339942 | 0.556698922 |
| HIC2 | hypermethylated in cancer 2 | 1.577421672 | 1.577421672 | up | 0.022264776 | 0.550353494 |
| MTF1 | metal-regulatory transcription factor 1 | 1.577377937 | 1.577377937 | up | 0.035059706 | 0.600153938 |
| LCN2 | lipocalin 2 | 1.577305049 | 1.577305049 | up | 0.036743418 | 0.605190228 |
| VANGL1 | VANGL planar cell polarity protein 1 | 1.577170214 | 1.577170214 | up | 0.023419114 | 0.556698922 |
| ITSN1 | intersectin 1 | 1.57713013 | 1.57713013 | up | 0.0132434 | 0.509292857 |
| EDC3 | enhancer of mRNA decapping 3 | 1.577086403 | 1.577086403 | up | 0.027132099 | 0.568317266 |

| | | | | | | |
|---|---|---|---|---|---|---|
| LRRC8B | leucine-rich repeat containing 8 family member B | 1.576926082 | 1.576926082 | up | 0.023201254 | 0.55642591 |
| IFITM1 | interferon induced transmembrane protein 1 | 1.576783993 | 1.576783993 | up | 0.005848217 | 0.409879029 |
| TMEM56 | transmembrane protein 56 | 1.576722061 | 1.576722061 | up | 0.030810493 | 0.582823189 |
| TMEM161A | transmembrane protein 161A | 1.576459786 | 1.576459786 | up | 0.036421163 | 0.603912936 |
| MCM2 | minichromosome maintenance complex component 2 | 1.576459786 | 1.576459786 | up | 0.018237238 | 0.536306821 |
| GATAD2B | GATA zinc finger domain containing 2B | 1.576401509 | 1.576401509 | up | 0.043148402 | 0.623333169 |
| ACAA2 | acetyl-CoA acyltransferase 2 | 1.576252184 | 1.576252184 | up | 0.01811047 | 0.536306821 |
| PPP2R5C | protein phosphatase 2 regulatory subunit B', gamma | 1.576008194 | 1.576008194 | up | 0.02351055 | 0.557200091 |
| TBCK | TBC1 domain containing kinase | 1.575767883 | 1.575767883 | up | 0.023637062 | 0.559178454 |
| LMNB2 | lamin B2 | 1.575727835 | 1.575727835 | up | 0.033601054 | 0.593014207 |
| PPP1R21 | protein phosphatase 1 regulatory subunit 21 | 1.575633179 | 1.575633179 | up | 0.025677275 | 0.566329497 |
| TPD52L1 | tumor protein D52-like 1 | 1.575618618 | 1.575618618 | up | 0.001608378 | 0.255663523 |
| DCK | deoxycytidine kinase | 1.575534889 | 1.575534889 | up | 0.010463751 | 0.486801343 |
| CAPG | capping protein (actin filament), gelsolin-like | 1.575200021 | 1.575200021 | up | 0.007032267 | 0.432539346 |
| NOP14 | NOP14 nucleolar protein | 1.575181824 | 1.575181824 | up | 0.019941658 | 0.549146 |
| LIMK2 | LIM domain kinase 2 | 1.575170906 | 1.575170906 | up | 0.022670031 | 0.551250506 |
| PIGX | phosphatidylinositol glycan anchor biosynthesis class X | 1.574807006 | 1.574807006 | up | 0.005511674 | 0.401886731 |
| ANXA4 | annexin A4 | 1.574770621 | 1.574770621 | up | 0.01029418 | 0.485753032 |
| NADK2 | NAD kinase 2, mitochondrial | 1.574643278 | 1.574643278 | up | 0.015647793 | 0.522063677 |
| DHRS3 | dehydrogenase/reductase (SDR family) member 3 | 1.574330424 | 1.574330424 | up | 0.033736863 | 0.593014207 |
| POLR2M | polymerase (RNA) II (DNA directed) polypeptide M | 1.57410128 | 1.57410128 | up | 0.023186496 | 0.55642591 |
| GCOM1 | GRINL1A complex locus 1 | 1.57410128 | 1.57410128 | up | 0.023186496 | 0.55642591 |
| ZDHHC9 | zinc finger, DHHC-type containing 9 | 1.573668543 | 1.573668543 | up | 0.043353605 | 0.623333169 |
| SUMF2 | sulfatase modifying factor 2 | 1.573410412 | 1.573410412 | up | 0.046414823 | 0.627185789 |
| ZNF362 | zinc finger protein 362 | 1.573265005 | 1.573265005 | up | 0.008214785 | 0.45571887 |
| MAGED2 | MAGE family member D2 | 1.573170497 | 1.573170497 | up | 0.015926246 | 0.523501532 |
| ALDOC | aldolase, fructose-bisphosphate C | 1.572999671 | 1.572999671 | up | 0.027088354 | 0.568317266 |
| PLTP | phospholipid transfer protein | 1.572854302 | 1.572854302 | up | 0.025324411 | 0.565656951 |
| HLA-A | major histocompatibility complex, class I, A | 1.572821596 | 1.572821596 | up | 0.002305827 | 0.297602824 |
| HLA-E | major histocompatibility complex, class I, E | 1.572556337 | 1.572556337 | up | 0.03180108 | 0.589351082 |
| TXNIP | thioredoxin interacting protein | 1.572476404 | 1.572476404 | up | 0.003461177 | 0.339991958 |
| ATP5L | ATP synthase, H+ transporting, mitochondrial Fo complex subunit G | 1.57238921 | 1.57238921 | up | 0.042341157 | 0.623333169 |
| PSMB9 | proteasome subunit beta 9 | 1.572294755 | 1.572294755 | up | 0.018461442 | 0.537128348 |
| UPK3B | uroplakin 3B | 1.572189408 | 1.572189408 | up | 0.012882748 | 0.50592937 |
| LOC105375355 | uroplakin-3b | 1.572189408 | 1.572189408 | up | 0.012882748 | 0.50592937 |
| COQ5 | coenzyme Q5, methyltransferase | 1.572153084 | 1.572153084 | up | 0.034239398 | 0.595139711 |
| TAF6 | TATA-box binding protein associated factor 6 | 1.572145819 | 1.572145819 | up | 0.021391874 | 0.549146 |
| PPL | periplakin | 1.572105863 | 1.572105863 | up | 0.042121253 | 0.623333169 |
| GTF2IRD1 | GTF2I repeat domain containing 1 | 1.572033217 | 1.572033217 | up | 0.019048908 | 0.541720736 |
| FKBP9P1 | FK506 binding protein 9 pseudogene 1 | 1.572025953 | 1.572025953 | up | 0.020482204 | 0.549146 |
| FKBP9 | FK506 binding protein 9 | 1.572025953 | 1.572025953 | up | 0.020482204 | 0.549146 |
| RNH1 | ribonuclease/angiogenin inhibitor 1 | 1.571844356 | 1.571844356 | up | 0.01816636 | 0.536306821 |
| ZAK | sterile alpha motif and leucine zipper containing kinase AZK | 1.571699094 | 1.571699094 | up | 0.026911318 | 0.568317266 |
| SMARCC2 | SWI/SNF related, matrix associated, actin dependent regulator of chromatin, subfamily c, member 2 | 1.571688199 | 1.571688199 | up | 0.028654405 | 0.574350948 |
| MPP1 | membrane protein, palmitoylated 1 | 1.57166278 | 1.57166278 | up | 0.026452448 | 0.568317266 |
| DXO | decapping exoribonuclease | 1.571637361 | 1.571637361 | up | 0.017997376 | 0.535584855 |
| FXN | frataxin | 1.571637361 | 1.571637361 | up | 0.028221302 | 0.57421459 |
| ENAH | enabled homolog (Drosophila) | 1.571056468 | 1.571056468 | up | 0.044181816 | 0.623333169 |
| TMC4 | transmembrane channel like 4 | 1.570791507 | 1.570791507 | up | 0.025228369 | 0.565656951 |
| B3GALT6 | UDP-Gal:betaGal beta 1,3-galactosyltransferase 6 | 1.570733439 | 1.570733439 | up | 0.018752526 | 0.539772279 |
| MOCOS | molybdenum cofactor sulfurase | 1.570548362 | 1.570548362 | up | 0.035418975 | 0.600698736 |
| PPP1R10 | protein phosphatase 1 regulatory subunit 10 | 1.569808275 | 1.569808275 | up | 0.043329625 | 0.623333169 |
| YWHAG | tyrosine 3-monooxygenase/tryptophan 5-monooxygenase activation protein, gamma | 1.569688587 | 1.569688587 | up | 0.032404508 | 0.590283874 |
| NDUFAF6 | NADH dehydrogenase (ubiquinone) complex I, assembly factor 6 | 1.569427483 | 1.569427483 | up | 0.044820943 | 0.623333169 |

| | | | | | | |
|---|---|---|---|---|---|---|
| TFPI2 | tissue factor pathway inhibitor 2 | 1.569271566 | 1.569271566 | up | 0.028500921 | 0.574350948 |
| KBTBD2 | kelch repeat and BTB (POZ) domain containing 2 | 1.569061285 | 1.569061285 | up | 0.046532357 | 0.627185789 |
| SMC3 | structural maintenance of chromosomes 3 | 1.568017545 | 1.568017545 | up | 0.0163224 | 0.524267534 |
| SHB | Src homology 2 domain containing adaptor protein B | 1.567977694 | 1.567977694 | up | 0.043887486 | 0.623333169 |
| GSTZ1 | glutathione S-transferase zeta 1 | 1.567756719 | 1.567756719 | up | 0.03521285 | 0.600433528 |
| SLC50A1 | solute carrier family 50 (sugar efflux transporter), member 1 | 1.567590102 | 1.567590102 | up | 0.029978967 | 0.579152964 |
| NUDT1 | nudix hydrolase 1 | 1.56754664 | 1.56754664 | up | 0.013018353 | 0.508400247 |
| EXOC5 | exocyst complex component 5 | 1.567503179 | 1.567503179 | up | 0.004876929 | 0.383960598 |
| MTERF3 | mitochondrial transcription termination factor 3 | 1.567412639 | 1.567412639 | up | 0.047996313 | 0.627854743 |
| ANKH | ANKH inorganic pyrophosphate transport regulator | 1.567383668 | 1.567383668 | up | 0.049088788 | 0.62943979 |
| VKORC1L1 | vitamin K epoxide reductase complex subunit 1 like 1 | 1.567293135 | 1.567293135 | up | 0.048065056 | 0.627892392 |
| TTC9 | tetratricopeptide repeat domain 9 | 1.567235196 | 1.567235196 | up | 0.035891913 | 0.603048422 |
| TBCD | tubulin folding cofactor D | 1.567180881 | 1.567180881 | up | 0.017000277 | 0.530571823 |
| CREM | cAMP responsive element modulator | 1.56717726 | 1.56717726 | up | 0.045681873 | 0.625770788 |
| CAPS | calcyphosine | 1.567122947 | 1.567122947 | up | 0.048116231 | 0.627892392 |
| DPYSL5 | dihydropyrimidinase like 5 | 1.566902092 | 1.566902092 | up | 0.041786877 | 0.623333169 |
| PPP3CB | protein phosphatase 3, catalytic subunit, beta isozyme | 1.566858649 | 1.566858649 | up | 0.014708494 | 0.515525808 |
| COPB2 | coatomer protein complex subunit beta 2 (beta prime) | 1.566844168 | 1.566844168 | up | 0.013842746 | 0.511661675 |
| CHMP1A | charged multivesicular body protein 1A | 1.566543722 | 1.566543722 | up | 0.040492849 | 0.623005985 |
| SMARCC1 | SWI/SNF related, matrix associated, actin dependent regulator of chromatin, subfamily c, member 1 | 1.566500289 | 1.566500289 | up | 0.026108569 | 0.567825319 |
| MT1X | metallothionein 1X | 1.566427903 | 1.566427903 | up | 0.021080444 | 0.549146 |
| BRE | brain and reproductive organ-expressed (TNFRSF1A modulator) | 1.566424284 | 1.566424284 | up | 0.013699197 | 0.511408446 |
| UQCC2 | ubiquinol-cytochrome c reductase complex assembly factor 2 | 1.566333806 | 1.566333806 | up | 0.007366709 | 0.437407205 |
| HCFC1R1 | host cell factor C1 regulator 1 (XPO1 dependent) | 1.566268665 | 1.566268665 | up | 0.018529126 | 0.537153195 |
| AMFR | autocrine motility factor receptor, E3 ubiquitin protein ligase | 1.566069641 | 1.566069641 | up | 0.021291985 | 0.549146 |
| GPATCH11 | G-patch domain containing 11 | 1.565805521 | 1.565805521 | up | 0.047731464 | 0.627854743 |
| COPS7A | COP9 signalosome subunit 7A | 1.565747638 | 1.565747638 | up | 0.038876088 | 0.613615561 |
| SIGMAR1 | sigma non-opioid intracellular receptor 1 | 1.565631877 | 1.565631877 | up | 0.012805875 | 0.5057776 |
| MPDU1 | mannose-P-dolichol utilization defect 1 | 1.565584852 | 1.565584852 | up | 0.022000937 | 0.550085548 |
| ACP2 | acid phosphatase 2, lysosomal | 1.565393149 | 1.565393149 | up | 0.018689386 | 0.539545418 |
| USP7 | ubiquitin specific peptidase 7 (herpes virus-associated) | 1.565237633 | 1.565237633 | up | 0.02677327 | 0.568317266 |
| HEXA | hexosaminidase A (alpha polypeptide) | 1.565042356 | 1.565042356 | up | 0.049082022 | 0.62943979 |
| PAWR | PRKC, apoptosis, WT1, regulator | 1.564886875 | 1.564886875 | up | 0.015544054 | 0.52192494 |
| ZDHHC8 | zinc finger, DHHC-type containing 8 | 1.564792871 | 1.564792871 | up | 0.026176 | 0.568317266 |
| UBXN2B | UBX domain protein 2B | 1.564651875 | 1.564651875 | up | 0.01646444 | 0.52581211 |
| ASAH1 | N-acylsphingosine amidohydrolase (acid ceramidase) 1 | 1.564622954 | 1.564622954 | up | 0.017609257 | 0.53179491 |
| KCTD10 | potassium channel tetramerization domain containing 10 | 1.564478359 | 1.564478359 | up | 0.012368894 | 0.503186668 |
| ECH1 | enoyl-CoA hydratase 1, peroxisomal | 1.564257877 | 1.564257877 | up | 0.010006036 | 0.479491277 |
| HNRNPL | heterogeneous nuclear ribonucleoprotein L | 1.564257877 | 1.564257877 | up | 0.010006036 | 0.479491277 |
| BIN1 | bridging integrator 1 | 1.564221736 | 1.564221736 | up | 0.024661695 | 0.563613185 |
| OLFM1 | olfactomedin 1 | 1.564181981 | 1.564181981 | up | 0.024177704 | 0.562764151 |
| PHGDH | phosphoglycerate dehydrogenase | 1.564022972 | 1.564022972 | up | 0.001689231 | 0.255663523 |
| PLA2G12A | phospholipase A2 group XIIA | 1.563784488 | 1.563784488 | up | 0.02981061 | 0.578931754 |
| RAB11A | RAB11A, member RAS oncogene family | 1.563755584 | 1.563755584 | up | 0.021762254 | 0.550085548 |
| C6orf120 | chromosome 6 open reading frame 120 | 1.563643583 | 1.563643583 | up | 0.041830444 | 0.623333169 |
| MAGED1 | MAGE family member D1 | 1.563574942 | 1.563574942 | up | 0.011034444 | 0.489992559 |
| ARF3 | ADP ribosylation factor 3 | 1.563459342 | 1.563459342 | up | 0.041324744 | 0.623333169 |
| SSBP3 | single stranded DNA binding protein 3 | 1.563426831 | 1.563426831 | up | 0.01489936 | 0.516117046 |
| GPHN | gephyrin | 1.563379872 | 1.563379872 | up | 0.046137209 | 0.627185789 |
| AP3B1 | adaptor-related protein complex 3, beta 1 subunit | 1.56317038 | 1.56317038 | up | 0.01626581 | 0.524267534 |
| AGO1 | argonaute 1, RISC catalytic component | 1.562718984 | 1.562718984 | up | 0.028085307 | 0.573730198 |
| AGO3 | argonaute 3, RISC catalytic component | 1.562718984 | 1.562718984 | up | 0.028085307 | 0.573730198 |
| RNFT1 | ring finger protein, transmembrane 1 | 1.562718984 | 1.562718984 | up | 0.030673694 | 0.582474665 |
| ITM2C | integral membrane protein 2C | 1.562708152 | 1.562708152 | up | 0.038562472 | 0.61336314 |

| | | | | | | |
|---|---|---|---|---|---|---|
| CDC27 | cell division cycle 27 | 1.562657604 | 1.562657604 | up | 0.021824825 | 0.550085548 |
| SCYL2 | SCY1-like, kinase-like 2 | 1.562599837 | 1.562599837 | up | 0.042820298 | 0.623333169 |
| TCF7L2 | transcription factor 7-like 2 (T-cell specific, HMG-box) | 1.562578175 | 1.562578175 | up | 0.02700413 | 0.568317266 |
| ABHD11 | abhydrolase domain containing 11 | 1.562473479 | 1.562473479 | up | 0.037845158 | 0.611314999 |
| PHIP | pleckstrin homology domain interacting protein | 1.562459039 | 1.562459039 | up | 0.047602574 | 0.62776393 |
| PDLIM1 | PDZ and LIM domain 1 | 1.562419329 | 1.562419329 | up | 0.002428671 | 0.302546299 |
| LHB | luteinizing hormone beta polypeptide | 1.562166653 | 1.562166653 | up | 0.035975242 | 0.603119509 |
| AIDA | axin interactor, dorsalization associated | 1.562112513 | 1.562112513 | up | 0.01396085 | 0.511949108 |
| MIER1 | mesoderm induction early response 1, transcriptional regulator | 1.562094467 | 1.562094467 | up | 0.030722356 | 0.582533534 |
| SLC3A2 | solute carrier family 3 (amino acid transporter heavy chain), member 2 | 1.562043939 | 1.562043939 | up | 0.007141063 | 0.434730278 |
| AKAP12 | A-kinase anchoring protein 12 | 1.562033112 | 1.562033112 | up | 0.005211119 | 0.39485568 |
| RBM23 | RNA binding motif protein 23 | 1.562015066 | 1.562015066 | up | 0.040451599 | 0.623005985 |
| FBLIM1 | filamin binding LIM protein 1 | 1.562011457 | 1.562011457 | up | 0.026362169 | 0.568317266 |
| NSL1 | NSL1, MIS12 kinetochore complex component | 1.561282607 | 1.561282607 | up | 0.016197777 | 0.524267534 |
| DECR2 | 2,4-dienoyl-CoA reductase 2, peroxisomal | 1.56116357 | 1.56116357 | up | 0.020457712 | 0.549146 |
| ASS1 | argininosuccinate synthase 1 | 1.561044541 | 1.561044541 | up | 0.018405339 | 0.537128348 |
| LZIC | leucine zipper and CTNNBIP1 domain containing | 1.560676693 | 1.560676693 | up | 0.038199692 | 0.612253826 |
| IER3IP1 | immediate early response 3 interacting protein 1 | 1.560319747 | 1.560319747 | up | 0.015701807 | 0.522063677 |
| NAB1 | NGFI-A binding protein 1 | 1.560265672 | 1.560265672 | up | 0.038394062 | 0.61288818 |
| ASXL1 | additional sex combs like 1, transcriptional regulator | 1.560186364 | 1.560186364 | up | 0.02824356 | 0.57421459 |
| RPL7L1 | ribosomal protein L7 like 1 | 1.560060201 | 1.560060201 | up | 0.015101081 | 0.517817132 |
| DCUN1D4 | DCN1, defective in cullin neddylation 1, domain containing 4 | 1.560016948 | 1.560016948 | up | 0.045287023 | 0.624555255 |
| GLMP | glycosylated lysosomal membrane protein | 1.559854758 | 1.559854758 | up | 0.017800563 | 0.53367796 |
| OLR1 | oxidized low density lipoprotein (lectin-like) receptor 1 | 1.559775471 | 1.559775471 | up | 0.021359342 | 0.549146 |
| PPP2R4 | protein phosphatase 2A regulatory subunit 4 | 1.559523223 | 1.559523223 | up | 0.030111559 | 0.57934581 |
| EPS8 | epidermal growth factor receptor pathway substrate 8 | 1.559516016 | 1.559516016 | up | 0.014193591 | 0.514636149 |
| PDGFC | platelet derived growth factor C | 1.559400716 | 1.559400716 | up | 0.014720466 | 0.515525808 |
| ZNF512 | zinc finger protein 512 | 1.559274617 | 1.559274617 | up | 0.039523393 | 0.616906565 |
| MARCKSL1 | MARCKS-like 1 | 1.558979225 | 1.558979225 | up | 0.014342706 | 0.514676952 |
| GALE | UDP-galactose-4-epimerase | 1.558752315 | 1.558752315 | up | 0.026425939 | 0.568317266 |
| PLXNB1 | plexin B1 | 1.558460622 | 1.558460622 | up | 0.038576004 | 0.61336314 |
| PPM1B | protein phosphatase, Mg2+/Mn2+ dependent 1B | 1.558374205 | 1.558374205 | up | 0.04301456 | 0.623333169 |
| RPP25L | ribonuclease P/MRP 25kDa subunit-like | 1.558327398 | 1.558327398 | up | 0.019008473 | 0.541720736 |
| TROAP | trophinin associated protein | 1.558111383 | 1.558111383 | up | 0.022601585 | 0.551250506 |
| SIPA1L3 | signal-induced proliferation-associated 1 like 3 | 1.558060983 | 1.558060983 | up | 0.016096181 | 0.524267534 |
| BCL3 | B-cell CLL/lymphoma 3 | 1.55781621 | 1.55781621 | up | 0.037141639 | 0.607428592 |
| CTBS | chitobiase, di-N-acetyl- | 1.557791015 | 1.557791015 | up | 0.038962741 | 0.613717043 |
| LGMN | legumain | 1.557683041 | 1.557683041 | up | 0.039831045 | 0.619742718 |
| CHMP4B | charged multivesicular body protein 4B | 1.55758947 | 1.55758947 | up | 0.009785046 | 0.477233577 |
| ASH2L | ash2 (absent, small, or homeotic)-like (Drosophila) | 1.557222436 | 1.557222436 | up | 0.024660566 | 0.563613185 |
| HP1BP3 | heterochromatin protein 1, binding protein 3 | 1.557107306 | 1.557107306 | up | 0.035171184 | 0.600433528 |
| PAM | peptidylglycine alpha-amidating monooxygenase | 1.557017366 | 1.557017366 | up | 0.014223908 | 0.514636149 |
| ARL6IP6 | ADP ribosylation factor like GTPase 6 interacting protein 6 | 1.556603712 | 1.556603712 | up | 0.044191155 | 0.623333169 |
| CTNNB1 | catenin beta 1 | 1.556582133 | 1.556582133 | up | 0.021874712 | 0.550085548 |
| CCHCR1 | coiled-coil alpha-helical rod protein 1 | 1.556283655 | 1.556283655 | up | 0.044058223 | 0.623333169 |
| CD99L2 | CD99 molecule like 2 | 1.556258484 | 1.556258484 | up | 0.026397318 | 0.568317266 |
| FAM98B | family with sequence similarity 98 member B | 1.556193763 | 1.556193763 | up | 0.026007086 | 0.567499406 |
| POMGNT1 | protein O-linked mannose N-acetylglucosaminyltransferase 1 (beta 1,2- [illegible] | 1.556085899 | 1.556085899 | up | 0.029307242 | 0.576629742 |
| HDGF | hepatoma-derived growth factor | 1.556028375 | 1.556028375 | up | 0.026897369 | 0.568317266 |
| SYNJ2BP- [illegible] | SYNJ2BP-COX16 readthrough | 1.555827058 | 1.555827058 | up | 0.020811908 | 0.549146 |
| SYNJ2BP | synaptojanin 2 binding protein | 1.555827058 | 1.555827058 | up | 0.020811908 | 0.549146 |
| BANF1 | barrier to autointegration factor 1 | 1.555801895 | 1.555801895 | up | 0.043612575 | 0.623333169 |
| RAB14 | RAB14, member RAS oncogene family | 1.555704842 | 1.555704842 | up | 0.04480643 | 0.623333169 |
| EIF4G1 | eukaryotic translation initiation factor 4 gamma, 1 | 1.555697653 | 1.555697653 | up | 0.037750621 | 0.610579997 |

| | | | | | | |
|---|---|---|---|---|---|---|
| AKR1A1 | aldo-keto reductase family 1, member A1 (aldehyde reductase) | 1.555485596 | 1.555485596 | up | 0.033414872 | 0.593014207 |
| CFAP97 | cilia and flagella associated protein 97 | 1.555424501 | 1.555424501 | up | 0.035569451 | 0.601354231 |
| FAM173B | family with sequence similarity 173 member B | 1.555370595 | 1.555370595 | up | 0.043117697 | 0.623333169 |
| PSAT1 | phosphoserine aminotransferase 1 | 1.555331065 | 1.555331065 | up | 0.005149671 | 0.39362485 |
| TMEM43 | transmembrane protein 43 | 1.555165769 | 1.555165769 | up | 0.042269875 | 0.623333169 |
| PCYOX1 | prenylcysteine oxidase 1 | 1.555004084 | 1.555004084 | up | 0.047157906 | 0.627322764 |
| TAGLN | transgelin | 1.55474183 | 1.55474183 | up | 0.018152497 | 0.536306821 |
| ADGRF1 | adhesion G protein-coupled receptor F1 | 1.554684355 | 1.554684355 | up | 0.018695286 | 0.539545418 |
| GUCD1 | guanylyl cyclase domain containing 1 | 1.554397015 | 1.554397015 | up | 0.043505532 | 0.623333169 |
| SLC44A1 | solute carrier family 44 (choline transporter), member 1 | 1.554016371 | 1.554016371 | up | 0.007435914 | 0.438278902 |
| SYNGR2 | synaptogyrin 2 | 1.553937381 | 1.553937381 | up | 0.0474733 | 0.627322764 |
| PAPSS2 | 3'-phosphoadenosine 5'-phosphosulfate synthase 2 | 1.553754284 | 1.553754284 | up | 0.043813915 | 0.623333169 |
| CPNE3 | copine III | 1.553721975 | 1.553721975 | up | 0.044731394 | 0.623333169 |
| TTC7A | tetratricopeptide repeat domain 7A | 1.553707616 | 1.553707616 | up | 0.023354136 | 0.556498532 |
| COTL1 | coactosin-like F-actin binding protein 1 | 1.553599925 | 1.553599925 | up | 0.042386107 | 0.623333169 |
| ATP6AP1 | ATPase, H+ transporting, lysosomal accessory protein 1 | 1.553420456 | 1.553420456 | up | 0.020371572 | 0.549146 |
| GNAQ | guanine nucleotide binding protein (G protein), q polypeptide | 1.553370209 | 1.553370209 | up | 0.035803438 | 0.602823932 |
| CFL1 | cofilin 1 (non-muscle) | 1.553219476 | 1.553219476 | up | 0.008700457 | 0.466623412 |
| LONP2 | lon peptidase 2, peroxisomal | 1.552928818 | 1.552928818 | up | 0.049402953 | 0.630325871 |
| PLBD1 | phospholipase B domain containing 1 | 1.552914466 | 1.552914466 | up | 0.031787924 | 0.589351082 |
| SGSM3 | small G protein signaling modulator 3 | 1.55290729 | 1.55290729 | up | 0.046052762 | 0.627185789 |
| DNAJC9 | DnaJ heat shock protein family (Hsp40) member C9 | 1.552774541 | 1.552774541 | up | 0.01455117 | 0.515525808 |
| ARHGAP1 | Rho GTPase activating protein 1 | 1.552225724 | 1.552225724 | up | 0.032656635 | 0.590424587 |
| CNN2 | calponin 2 | 1.552164757 | 1.552164757 | up | 0.049597623 | 0.630325871 |
| BAZ1B | bromodomain adjacent to zinc finger domain 1B | 1.552014141 | 1.552014141 | up | 0.026176453 | 0.568317266 |
| TMEM209 | transmembrane protein 209 | 1.55179183 | 1.55179183 | up | 0.042904332 | 0.623333169 |
| GRN | granulin | 1.551598231 | 1.551598231 | up | 0.020008619 | 0.549146 |
| BCAS4 | breast carcinoma amplified sequence 4 | 1.551573136 | 1.551573136 | up | 0.043938769 | 0.623333169 |
| SORD | sorbitol dehydrogenase | 1.551239777 | 1.551239777 | up | 0.039934062 | 0.620691429 |
| POT1 | protection of telomeres 1 | 1.551178848 | 1.551178848 | up | 0.045257675 | 0.624555255 |
| BNIP3L | BCL2/adenovirus E1B 19kDa interacting protein 3-like | 1.551031911 | 1.551031911 | up | 0.029501646 | 0.576629742 |
| FOLR1 | folate receptor 1 (adult) | 1.550988908 | 1.550988908 | up | 0.049593075 | 0.630325871 |
| PAK4 | p21 protein (Cdc42/Rac)-activated kinase 4 | 1.550963824 | 1.550963824 | up | 0.010377704 | 0.485753032 |
| ERP27 | endoplasmic reticulum protein 27 | 1.550892156 | 1.550892156 | up | 0.00939216 | 0.473004364 |
| ASCC1 | activating signal cointegrator 1 complex subunit 1 | 1.550809741 | 1.550809741 | up | 0.02095291 | 0.549146 |
| GNAI2 | guanine nucleotide binding protein (G protein), alpha inhibiting activity polypeptide 2 | 1.550612682 | 1.550612682 | up | 0.018511952 | 0.537153195 |
| SDHC | succinate dehydrogenase complex, subunit C, integral membrane protein, 15kDa | 1.550562525 | 1.550562525 | up | 0.023928698 | 0.562024581 |
| FAM229B | family with sequence similarity 229 member B | 1.550501623 | 1.550501623 | up | 0.044552466 | 0.623333169 |
| TIMELESS | timeless circadian clock | 1.550082536 | 1.550082536 | up | 0.042647349 | 0.623333169 |
| RNF10 | ring finger protein 10 | 1.55006821 | 1.55006821 | up | 0.0363777 | 0.603458973 |
| TSEN15 | tRNA splicing endonuclease subunit 15 | 1.549975096 | 1.549975096 | up | 0.010987231 | 0.489992559 |
| FAM120A | family with sequence similarity 120A | 1.549899893 | 1.549899893 | up | 0.003782162 | 0.351292726 |
| DDIT4 | DNA damage inducible transcript 4 | 1.549577634 | 1.549577634 | up | 0.017441301 | 0.531030242 |
| RBMS1 | RNA binding motif, single stranded interacting protein 1 | 1.549459489 | 1.549459489 | up | 0.039267199 | 0.614613629 |
| KDELR1 | KDEL (Lys-Asp-Glu-Leu) endoplasmic reticulum protein retention receptor 1 | 1.549445169 | 1.549445169 | up | 0.020191082 | 0.549146 |
| CRIP1 | cysteine-rich protein 1 (intestinal) | 1.549330614 | 1.549330614 | up | 0.034621554 | 0.597640739 |
| HS2ST1 | heparan sulfate 2-O-sulfotransferase 1 | 1.549309136 | 1.549309136 | up | 0.018366499 | 0.537128348 |
| KLK6 | kallikrein related peptidase 6 | 1.549309136 | 1.549309136 | up | 0.016530324 | 0.52581211 |
| IFT27 | intraflagellar transport 27 | 1.549294817 | 1.549294817 | up | 0.025042072 | 0.565551322 |
| SMIM4 | small integral membrane protein 4 | 1.549248283 | 1.549248283 | up | 0.019553236 | 0.546194159 |
| BSG | basigin (Ok blood group) | 1.548689978 | 1.548689978 | up | 0.018173086 | 0.536306821 |
| MOB1A | MOB kinase activator 1A | 1.548439522 | 1.548439522 | up | 0.041112026 | 0.623333169 |
| COQ2 | coenzyme Q2 4-hydroxybenzoate polyprenyltransferase | 1.548364393 | 1.548364393 | up | 0.047489441 | 0.627322764 |
| CEBPD | CCAAT/enhancer binding protein (C/EBP), delta | 1.548335773 | 1.548335773 | up | 0.017148722 | 0.530571823 |

| | | | | | | | |
|---|---|---|---|---|---|---|---|
| TSPAN13 | tetraspanin 13 | 1.547852897 | 1.547852897 | up | 0.031694887 | 0.589351082 |
| CSNK2B | casein kinase 2, beta polypeptide | 1.547795678 | 1.547795678 | up | 0.008915755 | 0.466623412 |
| IRF3 | interferon regulatory factor 3 | 1.547520337 | 1.547520337 | up | 0.0342434 | 0.595139711 |
| ADGRG1 | adhesion G protein-coupled receptor G1 | 1.547241471 | 1.547241471 | up | 0.013374803 | 0.509292857 |
| MEST | mesoderm specific transcript | 1.547223596 | 1.547223596 | up | 0.009204337 | 0.469308003 |
| SLC25A1 | solute carrier family 25 (mitochondrial carrier; citrate transporter), member 1 | 1.546973378 | 1.546973378 | up | 0.01121599 | 0.489992559 |
| IFITM2 | interferon induced transmembrane protein 2 | 1.546430184 | 1.546430184 | up | 0.028554933 | 0.574350948 |
| RSU1 | Ras suppressor protein 1 | 1.546330143 | 1.546330143 | up | 0.032176397 | 0.589852866 |
| LMBR1 | limb development membrane protein 1 | 1.546080069 | 1.546080069 | up | 0.049220218 | 0.630249923 |
| SUV39H2 | suppressor of variegation 3-9 homolog 2 | 1.545905041 | 1.545905041 | up | 0.040719453 | 0.623259064 |
| BZW2 | basic leucine zipper and W2 domains 2 | 1.545855036 | 1.545855036 | up | 0.02947108 | 0.576629742 |
| EIF3B | eukaryotic translation initiation factor 3 subunit B | 1.545833606 | 1.545833606 | up | 0.015204989 | 0.518081034 |
| ABCC1 | ATP binding cassette subfamily C member 1 | 1.545587183 | 1.545587183 | up | 0.030072111 | 0.579152964 |
| SERBP1 | SERPINE1 mRNA binding protein 1 | 1.545480055 | 1.545480055 | up | 0.013425503 | 0.509292857 |
| DPY19L4 | dpy-19-like 4 (C. elegans) | 1.545415782 | 1.545415782 | up | 0.044403837 | 0.623333169 |
| VEGFA | vascular endothelial growth factor A | 1.545219407 | 1.545219407 | up | 0.016111714 | 0.524267534 |
| QSOX1 | quiescin sulfhydryl oxidase 1 | 1.545083745 | 1.545083745 | up | 0.044412606 | 0.623333169 |
| FLJ23867 | uncharacterized protein FLJ23867 | 1.545083745 | 1.545083745 | up | 0.044412606 | 0.623333169 |
| PGK1 | phosphoglycerate kinase 1 | 1.545065896 | 1.545065896 | up | 0.038800948 | 0.613615561 |
| PPM1H | protein phosphatase, Mg2+/Mn2+ dependent 1H | 1.544865996 | 1.544865996 | up | 0.038117688 | 0.612253826 |
| HLA-C | major histocompatibility complex, class I, C | 1.544637572 | 1.544637572 | up | 0.011406398 | 0.493420716 |
| ATP1A1 | ATPase, Na+/K+ transporting, alpha 1 polypeptide | 1.544530509 | 1.544530509 | up | 0.007031112 | 0.432539346 |
| AKR1B1 | aldo-keto reductase family 1, member B1 (aldose reductase) | 1.544323543 | 1.544323543 | up | 0.026134545 | 0.568076394 |
| NCKAP1 | NCK-associated protein 1 | 1.544212935 | 1.544212935 | up | 0.043065037 | 0.623333169 |
| CLK2 | CDC like kinase 2 | 1.544027416 | 1.544027416 | up | 0.015147007 | 0.518081034 |
| OXCT1 | 3-oxoacid CoA-transferase 1 | 1.543891858 | 1.543891858 | up | 0.023806096 | 0.560818064 |
| KRT80 | keratin 80, type II | 1.543845486 | 1.543845486 | up | 0.049569142 | 0.630325871 |
| UBE2V1 | ubiquitin conjugating enzyme E2 variant 1 | 1.543677844 | 1.543677844 | up | 0.027664584 | 0.570815756 |
| TMEM189-UBE2V1 | TMEM189-UBE2V1 readthrough | 1.543677844 | 1.543677844 | up | 0.027664584 | 0.570815756 |
| BST2 | bone marrow stromal cell antigen 2 | 1.543574414 | 1.543574414 | up | 0.039296094 | 0.614821434 |
| PTK2 | protein tyrosine kinase 2 | 1.543331917 | 1.543331917 | up | 0.038125018 | 0.612253826 |
| MCCC2 | methylcrotonoyl-CoA carboxylase 2 | 1.543210683 | 1.543210683 | up | 0.015715319 | 0.522063677 |
| BECN1 | beclin 1, autophagy related | 1.542996763 | 1.542996763 | up | 0.048978327 | 0.62943979 |
| DCXR | dicarbonyl/L-xylulose reductase | 1.542946853 | 1.542946853 | up | 0.017234176 | 0.530571823 |
| PPDPF | pancreatic progenitor cell differentiation and proliferation factor | 1.542647425 | 1.542647425 | up | 0.044837212 | 0.623333169 |
| ME1 | malic enzyme 1, NADP(+)-dependent, cytosolic | 1.542258968 | 1.542258968 | up | 0.046101443 | 0.627185789 |
| GID8 | GID complex subunit 8 | 1.541909797 | 1.541909797 | up | 0.018767596 | 0.539772279 |
| HMMR | hyaluronan-mediated motility receptor (RHAMM) | 1.541806486 | 1.541806486 | up | 0.013593985 | 0.5101407 |
| DAZAP2 | DAZ associated protein 2 | 1.541767301 | 1.541767301 | up | 0.01734358 | 0.531030242 |
| ACTG1 | actin gamma 1 | 1.541756614 | 1.541756614 | up | 0.030615101 | 0.58235385 |
| ABCB7 | ATP binding cassette subfamily B member 7 | 1.541599885 | 1.541599885 | up | 0.043003672 | 0.623333169 |
| CDC42BPA | CDC42 binding protein kinase alpha (DMPK-like) | 1.541582076 | 1.541582076 | up | 0.049329569 | 0.630325871 |
| RPL22 | ribosomal protein L22 | 1.541578514 | 1.541578514 | up | 0.027681628 | 0.570815756 |
| MRPS33 | mitochondrial ribosomal protein S33 | 1.541400434 | 1.541400434 | up | 0.02935957 | 0.576629742 |
| BMP7 | bone morphogenetic protein 7 | 1.541144035 | 1.541144035 | up | 0.036183045 | 0.603150244 |
| SPOCK1 | sparc/osteonectin, cwcv and kazal-like domains proteoglycan (testican) 1 | 1.541015852 | 1.541015852 | up | 0.010832765 | 0.488995826 |
| SUMF1 | sulfatase modifying factor 1 | 1.54100517 | 1.54100517 | up | 0.037386671 | 0.608981156 |
| HNRNPU | heterogeneous nuclear ribonucleoprotein U (scaffold attachment factor A) | 1.540958885 | 1.540958885 | up | 0.011113141 | 0.489992559 |
| LITAF | lipopolysaccharide-induced TNF factor | 1.540780877 | 1.540780877 | up | 0.032803046 | 0.590424587 |
| TMEM54 | transmembrane protein 54 | 1.5403573 | 1.5403573 | up | 0.04419233 | 0.623333169 |
| DTD2 | D-tyrosyl-tRNA deacylase 2 (putative) | 1.540250534 | 1.540250534 | up | 0.034526154 | 0.596676967 |
| FGFR2 | fibroblast growth factor receptor 2 | 1.539834218 | 1.539834218 | up | 0.043811373 | 0.623333169 |
| SLC25A13 | solute carrier family 25 (aspartate/glutamate carrier), member 13 | 1.539802199 | 1.539802199 | up | 0.027437528 | 0.568462255 |
| SPPL2A | signal peptide peptidase like 2A | 1.539752392 | 1.539752392 | up | 0.03700687 | 0.606231976 |

| | | | | | | |
|---|---|---|---|---|---|---|
| HSDL2 | hydroxysteroid dehydrogenase like 2 | 1.539030372 | 1.539030372 | up | 0.032674163 | 0.590424587 |
| HNRNPA0 | heterogeneous nuclear ribonucleoprotein A0 | 1.539016148 | 1.539016148 | up | 0.036203888 | 0.603150244 |
| CD9 | CD9 molecule | 1.538920142 | 1.538920142 | up | 0.025598155 | 0.566329497 |
| CLPTM1 | cleft lip and palate associated transmembrane protein 1 | 1.538898808 | 1.538898808 | up | 0.042597189 | 0.623333169 |
| SCARB2 | scavenger receptor class B, member 2 | 1.538600166 | 1.538600166 | up | 0.032705925 | 0.590424587 |
| EIF2S2 | eukaryotic translation initiation factor 2 subunit beta | 1.538443757 | 1.538443757 | up | 0.031313257 | 0.586144908 |
| ERRFI1 | ERBB receptor feedback inhibitor 1 | 1.538305136 | 1.538305136 | up | 0.010036855 | 0.479491277 |
| ACADM | acyl-CoA dehydrogenase, C-4 to C-12 straight chain | 1.538045698 | 1.538045698 | up | 0.033594856 | 0.593014207 |
| NBPF1 | neuroblastoma breakpoint family member 1 | 1.537910666 | 1.537910666 | up | 0.025075799 | 0.565551322 |
| BCAT2 | branched chain amino-acid transaminase 2, mitochondrial | 1.53768327 | 1.53768327 | up | 0.033067867 | 0.592343125 |
| ACIN1 | apoptotic chromatin condensation inducer 1 | 1.537384863 | 1.537384863 | up | 0.049691697 | 0.630325871 |
| PRPSAP1 | phosphoribosyl pyrophosphate synthetase-associated protein 1 | 1.537225026 | 1.537225026 | up | 0.042118772 | 0.623333169 |
| TECR | trans-2,3-enoyl-CoA reductase | 1.537150442 | 1.537150442 | up | 0.038780004 | 0.613615561 |
| LAPTM4A | lysosomal protein transmembrane 4 alpha | 1.537029693 | 1.537029693 | up | 0.017461472 | 0.531030242 |
| CTBP1 | C-terminal binding protein 1 | 1.537008385 | 1.537008385 | up | 0.026447572 | 0.568317266 |
| MCM3 | minichromosome maintenance complex component 3 | 1.536916056 | 1.536916056 | up | 0.044086504 | 0.623333169 |
| DEK | DEK proto-oncogene | 1.536901852 | 1.536901852 | up | 0.010816022 | 0.488995826 |
| SLC5A3 | solute carrier family 5 (sodium/myo-inositol cotransporter), member 3 | 1.536834384 | 1.536834384 | up | 0.048776013 | 0.62943979 |
| R3HDM2 | R3H domain containing 2 | 1.536781122 | 1.536781122 | up | 0.02941622 | 0.576629742 |
| TNFRSF21 | tumor necrosis factor receptor superfamily member 21 | 1.536724312 | 1.536724312 | up | 0.015929062 | 0.523501532 |
| YIPF6 | Yip1 domain family member 6 | 1.536688807 | 1.536688807 | up | 0.025690973 | 0.566329497 |
| FAM127B | family with sequence similarity 127 member B | 1.536585845 | 1.536585845 | up | 0.041793006 | 0.623333169 |
| ARF5 | ADP ribosylation factor 5 | 1.536571644 | 1.536571644 | up | 0.01432538 | 0.514676952 |
| CTSV | cathepsin V | 1.536564544 | 1.536564544 | up | 0.047851197 | 0.627854743 |
| CAPNS1 | calpain, small subunit 1 | 1.536220211 | 1.536220211 | up | 0.02248271 | 0.551250506 |
| BLVRB | biliverdin reductase B | 1.5360463 | 1.5360463 | up | 0.020374881 | 0.549146 |
| ACADVL | acyl-CoA dehydrogenase, very long chain | 1.535939833 | 1.535939833 | up | 0.039590142 | 0.617458765 |
| C11orf70 | chromosome 11 open reading frame 70 | 1.535801437 | 1.535801437 | up | 0.02878438 | 0.574350948 |
| VMP1 | vacuole membrane protein 1 | 1.535368586 | 1.535368586 | up | 0.020886987 | 0.549146 |
| MIR21 | microRNA 21 | 1.535368586 | 1.535368586 | up | 0.020886987 | 0.549146 |
| MPV17 | MpV17 mitochondrial inner membrane protein | 1.535262166 | 1.535262166 | up | 0.03619121 | 0.603150244 |
| RAB1B | RAB1B, member RAS oncogene family | 1.535201865 | 1.535201865 | up | 0.0479905 | 0.627854743 |
| RAB1A | RAB1A, member RAS oncogene family | 1.535201865 | 1.535201865 | up | 0.0479905 | 0.627854743 |
| SMDT1 | single-pass membrane protein with aspartate-rich tail 1 | 1.535198318 | 1.535198318 | up | 0.04822377 | 0.627892392 |
| POLR2E | polymerase (RNA) II (DNA directed) polypeptide E, 25kDa | 1.534981962 | 1.534981962 | up | 0.046915948 | 0.627322764 |
| GNG10 | guanine nucleotide binding protein (G protein), gamma 10 | 1.534563525 | 1.534563525 | up | 0.008958155 | 0.466623412 |
| DNAJC25-[illegible] | DNAJC25-GNG10 readthrough | 1.534563525 | 1.534563525 | up | 0.008958155 | 0.466623412 |
| TM9SF3 | transmembrane 9 superfamily member 3 | 1.534372075 | 1.534372075 | up | 0.028785766 | 0.574350948 |
| SQSTM1 | sequestosome 1 | 1.534148747 | 1.534148747 | up | 0.028522163 | 0.574350948 |
| TSPAN6 | tetraspanin 6 | 1.533765974 | 1.533765974 | up | 0.038089458 | 0.612253826 |
| IDH2 | isocitrate dehydrogenase 2 (NADP+), mitochondrial | 1.533454155 | 1.533454155 | up | 0.04897579 | 0.62943979 |
| HDLBP | high density lipoprotein binding protein | 1.533269929 | 1.533269929 | up | 0.019302279 | 0.543126657 |
| EIF4EBP1 | eukaryotic translation initiation factor 4E binding protein 1 | 1.533259301 | 1.533259301 | up | 0.041632979 | 0.623333169 |
| RXRA | retinoid X receptor alpha | 1.532766961 | 1.532766961 | up | 0.045592201 | 0.625770788 |
| FAM122B | family with sequence similarity 122B | 1.532529703 | 1.532529703 | up | 0.043646174 | 0.623333169 |
| MORC2 | MORC family CW-type zinc finger 2 | 1.532359749 | 1.532359749 | up | 0.043050531 | 0.623333169 |
| GPX3 | glutathione peroxidase 3 | 1.532172114 | 1.532172114 | up | 0.044571256 | 0.623333169 |
| TADA3 | transcriptional adaptor 3 | 1.532111934 | 1.532111934 | up | 0.038875274 | 0.613615561 |
| DBNL | drebrin-like | 1.531729669 | 1.531729669 | up | 0.032796723 | 0.590424587 |
| ZDHHC4 | zinc finger, DHHC-type containing 4 | 1.531694279 | 1.531694279 | up | 0.043006971 | 0.623333169 |
| UBE2D1 | ubiquitin conjugating enzyme E2D 1 | 1.531662429 | 1.531662429 | up | 0.032918365 | 0.590894404 |
| HSPB11 | heat shock protein family B (small) member 11 | 1.531241359 | 1.531241359 | up | 0.036281687 | 0.603215176 |
| ATP6V1A | ATPase, H+ transporting, lysosomal 70kDa, V1 subunit A | 1.531152913 | 1.531152913 | up | 0.026632315 | 0.568317266 |
| KLHL5 | kelch like family member 5 | 1.531032636 | 1.531032636 | up | 0.035337795 | 0.600698736 |

| | | | | | | |
|---|---|---|---|---|---|---|
| IARS | isoleucyl-tRNA synthetase | 1.530993724 | 1.530993724 | up | 0.007720151 | 0.44458454 |
| HNRNPA3 | heterogeneous nuclear ribonucleoprotein A3 | 1.530869922 | 1.530869922 | up | 0.049232021 | 0.630249923 |
| HNRNPA3P1 | heterogeneous nuclear ribonucleoprotein A3 pseudogene 1 | 1.530869922 | 1.530869922 | up | 0.049232021 | 0.630249923 |
| GBAP1 | glucosidase, beta, acid pseudogene 1 | 1.530831015 | 1.530831015 | up | 0.038806299 | 0.613615561 |
| GBA | glucosidase, beta, acid | 1.530831015 | 1.530831015 | up | 0.038806299 | 0.613615561 |
| PRCP | prolylcarboxypeptidase | 1.530785035 | 1.530785035 | up | 0.034072234 | 0.595139711 |
| TMSB4X | thymosin beta 4, X-linked | 1.530512721 | 1.530512721 | up | 0.046509764 | 0.627185789 |
| APMAP | adipocyte plasma membrane associated protein | 1.53006722 | 1.53006722 | up | 0.021404777 | 0.549146 |
| CDK4 | cyclin-dependent kinase 4 | 1.52920134 | 1.52920134 | up | 0.040104651 | 0.621238987 |
| OST4 | oligosaccharyltransferase complex subunit 4 (non-catalytic) | 1.528844527 | 1.528844527 | up | 0.038109428 | 0.612253826 |
| CHMP3 | charged multivesicular body protein 3 | 1.528321824 | 1.528321824 | up | 0.021294923 | 0.549146 |
| RNF103-CHMP3 | RNF103-CHMP3 readthrough | 1.528321824 | 1.528321824 | up | 0.021294923 | 0.549146 |
| ORMDL2 | ORMDL sphingolipid biosynthesis regulator 2 | 1.528215893 | 1.528215893 | up | 0.030071259 | 0.579152964 |
| LSM4 | LSM4 homolog, U6 small nuclear RNA and mRNA degradation associated | 1.527700464 | 1.527700464 | up | 0.040494536 | 0.623005985 |
| ATP5G2 | ATP synthase, H+ transporting, mitochondrial Fo complex subunit C2 (subunit 9) | 1.527531046 | 1.527531046 | up | 0.024265658 | 0.563333857 |
| TAX1BP1 | Tax1 (human T-cell leukemia virus type I) binding protein 1 | 1.527435757 | 1.527435757 | up | 0.008956165 | 0.466623412 |
| TARS | threonyl-tRNA synthetase | 1.527199324 | 1.527199324 | up | 0.042383418 | 0.623333169 |
| KHDRBS1 | KH domain containing, RNA binding, signal transduction associated 1 | 1.526790063 | 1.526790063 | up | 0.046512988 | 0.627185789 |
| UBE2I | ubiquitin conjugating enzyme E2I | 1.526529041 | 1.526529041 | up | 0.048346039 | 0.627892392 |
| GPX4 | glutathione peroxidase 4 | 1.525915461 | 1.525915461 | up | 0.01539173 | 0.521001176 |
| NDUFA13 | NADH:ubiquinone oxidoreductase subunit A13 | 1.525897833 | 1.525897833 | up | 0.037753124 | 0.610579997 |
| PRPF19 | pre-mRNA processing factor 19 | 1.525756816 | 1.525756816 | up | 0.041622013 | 0.623333169 |
| DNAJC15 | DnaJ heat shock protein family (Hsp40) member C15 | 1.525721564 | 1.525721564 | up | 0.039814378 | 0.619728343 |
| LOC102724770 | protein DGCR6 | 1.525573515 | 1.525573515 | up | 0.036961734 | 0.606003692 |
| DGCR6 | DiGeorge syndrome critical region gene 6 | 1.525573515 | 1.525573515 | up | 0.036961734 | 0.606003692 |
| DGCR6L | DiGeorge syndrome critical region gene 6-like | 1.525573515 | 1.525573515 | up | 0.036961734 | 0.606003692 |
| PDIA6 | protein disulfide isomerase family A member 6 | 1.525446626 | 1.525446626 | up | 0.026551529 | 0.568317266 |
| EIF3E | eukaryotic translation initiation factor 3 subunit E | 1.525347943 | 1.525347943 | up | 0.012580469 | 0.504821772 |
| CYBA | cytochrome b-245, alpha polypeptide | 1.524548136 | 1.524548136 | up | 0.032056947 | 0.589351082 |
| CCDC71L | coiled-coil domain containing 71-like | 1.524519956 | 1.524519956 | up | 0.043227528 | 0.623333169 |
| NADK | NAD kinase | 1.5244953 | 1.5244953 | up | 0.044508695 | 0.623333169 |
| SCNM1 | sodium channel modifier 1 | 1.523196113 | 1.523196113 | up | 0.046230461 | 0.627185789 |
| TNFAIP8L2-SCNM1 | TNFAIP8L2-SCNM1 readthrough | 1.523196113 | 1.523196113 | up | 0.046230461 | 0.627185789 |
| PRSS23 | protease, serine 23 | 1.523041271 | 1.523041271 | up | 0.033269969 | 0.593014207 |
| PTGR1 | prostaglandin reductase 1 | 1.522925149 | 1.522925149 | up | 0.049587145 | 0.630325871 |
| ITM2B | integral membrane protein 2B | 1.522840702 | 1.522840702 | up | 0.021697247 | 0.550085548 |
| MTPN | myotrophin | 1.522612016 | 1.522612016 | up | 0.0466582 | 0.627185789 |
| LUZP6 | leucine zipper protein 6 | 1.522612016 | 1.522612016 | up | 0.0466582 | 0.627185789 |
| IMPA2 | inositol(myo)-1(or 4)-monophosphatase 2 | 1.522527587 | 1.522527587 | up | 0.042622148 | 0.623333169 |
| EEF2 | eukaryotic translation elongation factor 2 | 1.52190155 | 1.52190155 | up | 0.036196648 | 0.603150244 |
| P4HB | prolyl 4-hydroxylase, beta polypeptide | 1.520657275 | 1.520657275 | up | 0.043162046 | 0.623333169 |
| YAP1 | Yes associated protein 1 | 1.520232205 | 1.520232205 | up | 0.033406455 | 0.593014207 |
| NGFRAP1 | nerve growth factor receptor (TNFRSF16) associated protein 1 | 1.519824812 | 1.519824812 | up | 0.012478859 | 0.503825939 |
| RPF2 | ribosome production factor 2 homolog | 1.519403485 | 1.519403485 | up | 0.045362708 | 0.624555255 |
| B2M | beta-2-microglobulin | 1.519396464 | 1.519396464 | up | 0.043116793 | 0.623333169 |
| GNAS | GNAS complex locus | 1.518582235 | 1.518582235 | up | 0.041762684 | 0.623333169 |
| TUBB | tubulin, beta class I | 1.51730211 | 1.51730211 | up | 0.048065592 | 0.627892392 |
| FTL | ferritin, light polypeptide | 1.515960016 | 1.515960016 | up | 0.01095097 | 0.489869256 |
| TPM3 | tropomyosin 3 | 1.515343682 | 1.515343682 | up | 0.03696469 | 0.606003692 |
| PSAP | prosaposin | 1.515060112 | 1.515060112 | up | 0.035983607 | 0.603119509 |
| KDELR2 | KDEL (Lys-Asp-Glu-Leu) endoplasmic reticulum protein retention receptor 2 | 1.514528125 | 1.514528125 | up | 0.03410894 | 0.595139711 |
| LSM3 | LSM3 homolog, U6 small nuclear RNA and mRNA degradation associated | 1.513964842 | 1.513964842 | up | 0.04850977 | 0.62898126 |
| SOD1 | superoxide dismutase 1, soluble | 1.512237815 | 1.512237815 | up | 0.047925308 | 0.627854743 |
| BRIX1 | BRX1, biogenesis of ribosomes | 1.51208758 | 1.51208758 | up | 0.044944075 | 0.623333169 |

| | | | | | | |
|---|---|---|---|---|---|---|
| SRP14 | signal recognition particle 14kDa | 1.511654427 | 1.511654427 | up | 0.035682037 | 0.602074261 |
| RPL13P12 | ribosomal protein L13 pseudogene 12 | 1.50565201 | 1.50565201 | up | 0.028655268 | 0.574350948 |
| RPL13 | ribosomal protein L13 | 1.50565201 | 1.50565201 | up | 0.028655268 | 0.574350948 |
| HMGN2 | high mobility group nucleosomal binding domain 2 | 1.503045173 | 1.503045173 | up | 0.049479018 | 0.630325871 |
| NPM1 | nucleophosmin (nucleolar phosphoprotein B23, numatrin) | -1.505805085 | 1.505805085 | down | 0.037835006 | 0.611314999 |
| CCT3 | chaperonin containing TCP1, subunit 3 (gamma) | -1.508357461 | 1.508357461 | down | 0.048724154 | 0.62943979 |
| NEDD8 | neural precursor cell expressed, developmentally down-regulated 8 | -1.508587492 | 1.508587492 | down | 0.044182417 | 0.623333169 |
| LOC100128775 | neural precursor cell expressed, developmentally down-regulated 8 pseudogene | -1.508587492 | 1.508587492 | down | 0.044182417 | 0.623333169 |
| ZFAS1 | ZNFX1 antisense RNA 1 | -1.508646748 | 1.508646748 | down | 0.049798998 | 0.630405293 |
| EFEMP1 | EGF containing fibulin-like extracellular matrix protein 1 | -1.508807099 | 1.508807099 | down | 0.046541312 | 0.627185789 |
| BRD2 | bromodomain containing 2 | -1.512842399 | 1.512842399 | down | 0.049790663 | 0.630405293 |
| VDAC2 | voltage-dependent anion channel 2 | -1.513908875 | 1.513908875 | down | 0.028331541 | 0.57421459 |
| PHB | prohibitin | -1.51445814 | 1.51445814 | down | 0.043594956 | 0.623333169 |
| PCBP2 | poly(rC) binding protein 2 | -1.514923598 | 1.514923598 | down | 0.030009603 | 0.579152964 |
| PRMT1 | protein arginine methyltransferase 1 | -1.515651818 | 1.515651818 | down | 0.042962185 | 0.623333169 |
| NUTF2 | nuclear transport factor 2 | -1.515767385 | 1.515767385 | down | 0.049517575 | 0.630325871 |
| ACLY | ATP citrate lyase | -1.515893468 | 1.515893468 | down | 0.0418006 | 0.623333169 |
| PSMB2 | proteasome subunit beta 2 | -1.516198212 | 1.516198212 | down | 0.046164995 | 0.627185789 |
| MRFAP1 | Morf4 family associated protein 1 | -1.517554542 | 1.517554542 | down | 0.045789182 | 0.626389846 |
| TUBB6 | tubulin, beta 6 class V | -1.51849452 | 1.51849452 | down | 0.030583949 | 0.582174647 |
| BIRC2 | baculoviral IAP repeat containing 2 | -1.519185846 | 1.519185846 | down | 0.038757002 | 0.613615561 |
| SNU13 | SNU13 homolog, small nuclear ribonucleoprotein (U4/U6.U5) | -1.519308703 | 1.519308703 | down | 0.034207282 | 0.595139711 |
| ITGA2 | integrin, alpha 2 (CD49B, alpha 2 subunit of VLA-2 receptor) | -1.51952636 | 1.51952636 | down | 0.03605931 | 0.603150244 |
| ELAC2 | elaC ribonuclease Z 2 | -1.520499177 | 1.520499177 | down | 0.025328645 | 0.565656951 |
| NOP56 | NOP56 ribonucleoprotein | -1.520530796 | 1.520530796 | down | 0.04196971 | 0.623333169 |
| F3 | coagulation factor III (thromboplastin, tissue factor) | -1.520653762 | 1.520653762 | down | 0.036888833 | 0.606003692 |
| MRPS12 | mitochondrial ribosomal protein S12 | -1.520832958 | 1.520832958 | down | 0.045669348 | 0.625770788 |
| DAP3 | death associated protein 3 | -1.521005148 | 1.521005148 | down | 0.034696574 | 0.597999402 |
| PSMC6 | proteasome 26S subunit, ATPase 6 | -1.521398797 | 1.521398797 | down | 0.045703528 | 0.625770788 |
| CPNE1 | copine I | -1.521430434 | 1.521430434 | down | 0.040837984 | 0.623333169 |
| LSM10 | LSM10, U7 small nuclear RNA associated | -1.521859355 | 1.521859355 | down | 0.043232646 | 0.623333169 |
| DDX1 | DEAD (Asp-Glu-Ala-Asp) box helicase 1 | -1.522059794 | 1.522059794 | down | 0.039264215 | 0.614613629 |
| TNFRSF12A | tumor necrosis factor receptor superfamily member 12A | -1.52240447 | 1.52240447 | down | 0.022956483 | 0.554273475 |
| NAP1L1 | nucleosome assembly protein 1-like 1 | -1.52275626 | 1.52275626 | down | 0.037234955 | 0.607707429 |
| PNN | pinin, desmosome associated protein | -1.523002562 | 1.523002562 | down | 0.02925059 | 0.576629742 |
| CNPY2 | canopy FGF signaling regulator 2 | -1.523150363 | 1.523150363 | down | 0.029922682 | 0.579152964 |
| PSMC2 | proteasome 26S subunit, ATPase 2 | -1.523238346 | 1.523238346 | down | 0.037990917 | 0.612253826 |
| ARPC5L | actin related protein 2/3 complex subunit 5-like | -1.523312256 | 1.523312256 | down | 0.027107361 | 0.568317266 |
| RPL26L1 | ribosomal protein L26 like 1 | -1.523773393 | 1.523773393 | down | 0.046728159 | 0.627185789 |
| SRSF1 | serine/arginine-rich splicing factor 1 | -1.52385437 | 1.52385437 | down | 0.019870056 | 0.549146 |
| ARL4A | ADP ribosylation factor like GTPase 4A | -1.523864933 | 1.523864933 | down | 0.028906405 | 0.574350948 |
| GSTO1 | glutathione S-transferase omega 1 | -1.524470644 | 1.524470644 | down | 0.037444138 | 0.609086153 |
| TUBA1C | tubulin, alpha 1c | -1.525217549 | 1.525217549 | down | 0.035195318 | 0.600433528 |
| DDA1 | DET1 and DDB1 associated 1 | -1.525605238 | 1.525605238 | down | 0.045566964 | 0.625770788 |
| LDHA | lactate dehydrogenase A | -1.526169326 | 1.526169326 | down | 0.015954471 | 0.523501532 |
| MED31 | mediator complex subunit 31 | -1.526324487 | 1.526324487 | down | 0.046437273 | 0.627185789 |
| BZW1 | basic leucine zipper and W2 domains 1 | -1.52688884 | 1.52688884 | down | 0.032797439 | 0.590424587 |
| CERS2 | ceramide synthase 2 | -1.52703349 | 1.52703349 | down | 0.027074168 | 0.568317266 |
| HACD3 | 3-hydroxyacyl-CoA dehydratase 3 | -1.527139339 | 1.527139339 | down | 0.032110751 | 0.589617364 |
| SEPT9 | septin 9 | -1.527993461 | 1.527993461 | down | 0.013405677 | 0.509292857 |
| PLAA | phospholipase A2-activating protein | -1.528943437 | 1.528943437 | down | 0.034182594 | 0.595139711 |
| EIF1 | eukaryotic translation initiation factor 1 | -1.528943437 | 1.528943437 | down | 0.039609268 | 0.617512407 |
| PTTG1IP | pituitary tumor-transforming 1 interacting protein | -1.528971698 | 1.528971698 | down | 0.022349219 | 0.551238726 |
| REXO2 | RNA exonuclease 2 | -1.528982297 | 1.528982297 | down | 0.020389176 | 0.549146 |

| | | | | | | |
|---|---|---|---|---|---|---|
| MRPL45 | mitochondrial ribosomal protein L45 | -1.529356809 | 1.529356809 | down | 0.04822931 | 0.627892392 |
| SELK | selenoprotein K | -1.529370943 | 1.529370943 | down | 0.048760437 | 0.62943979 |
| DDX3X | DEAD (Asp-Glu-Ala-Asp) box helicase 3, X-linked | -1.529614781 | 1.529614781 | down | 0.022081229 | 0.550353494 |
| SLC39A1 | solute carrier family 39 (zinc transporter), member 1 | -1.529657191 | 1.529657191 | down | 0.033866538 | 0.594363028 |
| ATP6V0B | ATPase, H+ transporting, lysosomal 21kDa, V0 subunit b | -1.530099037 | 1.530099037 | down | 0.025606132 | 0.566329497 |
| SH3BGRL3 | SH3 domain binding glutamate-rich protein like 3 | -1.530190957 | 1.530190957 | down | 0.031993458 | 0.589351082 |
| NTMT1 | N-terminal Xaa-Pro-Lys N-methyltransferase 1 | -1.530297026 | 1.530297026 | down | 0.030654313 | 0.582387558 |
| SRSF2 | serine/arginine-rich splicing factor 2 | -1.530311169 | 1.530311169 | down | 0.036101066 | 0.603150244 |
| ILF3 | interleukin enhancer binding factor 3 | -1.530487967 | 1.530487967 | down | 0.025082247 | 0.565551322 |
| NNMT | nicotinamide N-methyltransferase | -1.530597593 | 1.530597593 | down | 0.039410585 | 0.616122981 |
| TWISTNB | TWIST neighbor | -1.530608202 | 1.530608202 | down | 0.031844876 | 0.589351082 |
| NOLC1 | nucleolar and coiled-body phosphoprotein 1 | -1.530682469 | 1.530682469 | down | 0.024320982 | 0.563505276 |
| PSMB5 | proteasome subunit beta 5 | -1.530717836 | 1.530717836 | down | 0.030481483 | 0.581882081 |
| UBE2L3 | ubiquitin conjugating enzyme E2L 3 | -1.530795646 | 1.530795646 | down | 0.025793961 | 0.566329497 |
| DHX9 | DEAH (Asp-Glu-Ala-His) box helicase 9 | -1.530873459 | 1.530873459 | down | 0.0333365 | 0.593014207 |
| CDK9 | cyclin-dependent kinase 9 | -1.531050323 | 1.531050323 | down | 0.049642967 | 0.630325871 |
| ASL | argininosuccinate lyase | -1.531287352 | 1.531287352 | down | 0.046962122 | 0.627322764 |
| SNX17 | sorting nexin 17 | -1.531414726 | 1.531414726 | down | 0.046965928 | 0.627322764 |
| KIAA1143 | KIAA1143 | -1.531591653 | 1.531591653 | down | 0.04887554 | 0.62943979 |
| CCAR1 | cell division cycle and apoptosis regulator 1 | -1.531595191 | 1.531595191 | down | 0.045877505 | 0.627096746 |
| ZCCHC17 | zinc finger, CCHC domain containing 17 | -1.532023438 | 1.532023438 | down | 0.040163933 | 0.621238987 |
| MRPL3 | mitochondrial ribosomal protein L3 | -1.532377452 | 1.532377452 | down | 0.035278194 | 0.600698736 |
| TMEM18 | transmembrane protein 18 | -1.532575735 | 1.532575735 | down | 0.041651859 | 0.623333169 |
| SH3GLB1 | SH3-domain GRB2-like endophilin B1 | -1.532618228 | 1.532618228 | down | 0.036313823 | 0.603215176 |
| RAP1B | RAP1B, member of RAS oncogene family | -1.532621769 | 1.532621769 | down | 0.03557196 | 0.601354231 |
| FADD | Fas associated via death domain | -1.532667804 | 1.532667804 | down | 0.046476204 | 0.627185789 |
| EEF1E1 | eukaryotic translation elongation factor 1 epsilon 1 | -1.532809459 | 1.532809459 | down | 0.048277413 | 0.627892392 |
| EEF1E1-BLOC1S5 | EEF1E1-BLOC1S5 readthrough (NMD candidate) | -1.532809459 | 1.532809459 | down | 0.048277413 | 0.627892392 |
| ARL6IP4 | ADP ribosylation factor like GTPase 6 interacting protein 4 | -1.532844875 | 1.532844875 | down | 0.04670689 | 0.627185789 |
| NDUFB10 | NADH:ubiquinone oxidoreductase subunit B10 | -1.532890917 | 1.532890917 | down | 0.02787067 | 0.572241118 |
| ANXA2 | annexin A2 | -1.532933418 | 1.532933418 | down | 0.008767571 | 0.466623412 |
| PTGES3 | prostaglandin E synthase 3 | -1.533149485 | 1.533149485 | down | 0.04432582 | 0.623333169 |
| DCUN1D5 | DCN1, defective in cullin neddylation 1, domain containing 5 | -1.533365582 | 1.533365582 | down | 0.026955795 | 0.568317266 |
| S100A16 | S100 calcium binding protein A16 | -1.533574623 | 1.533574623 | down | 0.012339629 | 0.503186668 |
| TES | testin LIM domain protein | -1.53360297 | 1.53360297 | down | 0.031967969 | 0.589351082 |
| GLO1 | glyoxalase I | -1.53360297 | 1.53360297 | down | 0.027180465 | 0.568317266 |
| CXorf40A | chromosome X open reading frame 40A | -1.533765974 | 1.533765974 | down | 0.048314513 | 0.627892392 |
| CXorf40B | chromosome X open reading frame 40B | -1.533765974 | 1.533765974 | down | 0.048314513 | 0.627892392 |
| CST6 | cystatin E/M | -1.533943171 | 1.533943171 | down | 0.026327564 | 0.568317266 |
| MRPL14 | mitochondrial ribosomal protein L14 | -1.53395026 | 1.53395026 | down | 0.018226215 | 0.536306821 |
| GPN1 | GPN-loop GTPase 1 | -1.534021145 | 1.534021145 | down | 0.034411167 | 0.596656508 |
| CTU2 | cytosolic thiouridylase subunit 2 homolog (S. pombe) | -1.534028234 | 1.534028234 | down | 0.044191638 | 0.623333169 |
| PRKAR2A | protein kinase, cAMP-dependent, regulatory subunit type II alpha | -1.534028234 | 1.534028234 | down | 0.044191638 | 0.623333169 |
| C6orf62 | chromosome 6 open reading frame 62 | -1.534269269 | 1.534269269 | down | 0.049519091 | 0.630325871 |
| RNF181 | ring finger protein 181 | -1.534538706 | 1.534538706 | down | 0.029714944 | 0.577947966 |
| RARS | arginyl-tRNA synthetase | -1.534542252 | 1.534542252 | down | 0.027822864 | 0.572241118 |
| MRPS34 | mitochondrial ribosomal protein S34 | -1.534570616 | 1.534570616 | down | 0.020163178 | 0.549146 |
| LRRC47 | leucine rich repeat containing 47 | -1.534726631 | 1.534726631 | down | 0.007125271 | 0.434730278 |
| C21orf59 | chromosome 21 open reading frame 59 | -1.534747907 | 1.534747907 | down | 0.037739888 | 0.610579997 |
| PRPF8 | pre-mRNA processing factor 8 | -1.534769183 | 1.534769183 | down | 0.016840818 | 0.530351036 |
| PSMA5 | proteasome subunit alpha 5 | -1.534772729 | 1.534772729 | down | 0.025771987 | 0.566329497 |
| MRPL34 | mitochondrial ribosomal protein L34 | -1.534794006 | 1.534794006 | down | 0.019729817 | 0.548491703 |
| ADRM1 | adhesion regulating molecule 1 | -1.534950044 | 1.534950044 | down | 0.013980574 | 0.511949108 |
| TRIM28 | tripartite motif containing 28 | -1.534950044 | 1.534950044 | down | 0.034756196 | 0.598002352 |

| | | | | | | |
|---|---|---|---|---|---|---|
| DHX38 | DEAH (Asp-Glu-Ala-His) box polypeptide 38 | -1.534974869 | 1.534974869 | down | 0.024992677 | 0.565551322 |
| PSMC1 | proteasome 26S subunit, ATPase 1 | -1.535031615 | 1.535031615 | down | 0.01415835 | 0.514636149 |
| TRIM33 | tripartite motif containing 33 | -1.535031615 | 1.535031615 | down | 0.037475209 | 0.609325627 |
| PTPN2 | protein tyrosine phosphatase, non-receptor type 2 | -1.535265713 | 1.535265713 | down | 0.041475417 | 0.623333169 |
| CDCA5 | cell division cycle associated 5 | -1.535822727 | 1.535822727 | down | 0.033636927 | 0.593014207 |
| MFF | mitochondrial fission factor | -1.535854664 | 1.535854664 | down | 0.032591391 | 0.590424587 |
| TMEM14C | transmembrane protein 14C | -1.535886602 | 1.535886602 | down | 0.009067686 | 0.466623412 |
| MYL6B | myosin light chain 6B | -1.535922089 | 1.535922089 | down | 0.034931295 | 0.599159228 |
| UCHL3 | ubiquitin C-terminal hydrolase L3 | -1.536337347 | 1.536337347 | down | 0.031256398 | 0.585954505 |
| OLFML3 | olfactomedin like 3 | -1.53685924 | 1.53685924 | down | 0.035336457 | 0.600698736 |
| ORAI1 | ORAI calcium release-activated calcium modulator 1 | -1.537452355 | 1.537452355 | down | 0.047025521 | 0.627322764 |
| FLNB | filamin B, beta | -1.537484326 | 1.537484326 | down | 0.019059652 | 0.541720736 |
| ATP6V1D | ATPase, H+ transporting, lysosomal 34kDa, V1 subunit D | -1.537555374 | 1.537555374 | down | 0.015376867 | 0.521001176 |
| DUSP14 | dual specificity phosphatase 14 | -1.53768327 | 1.53768327 | down | 0.019922603 | 0.549146 |
| TGFBI | transforming growth factor beta induced | -1.53785026 | 1.53785026 | down | 0.040495085 | 0.623005985 |
| FAM195A | family with sequence similarity 195 member A | -1.537853814 | 1.537853814 | down | 0.032696869 | 0.590424587 |
| LOC100287175 | uncharacterized LOC100287175 | -1.537853814 | 1.537853814 | down | 0.032696869 | 0.590424587 |
| ZMIZ2 | zinc finger, MIZ-type containing 2 | -1.538194958 | 1.538194958 | down | 0.041883357 | 0.623333169 |
| KDM2A | lysine (K)-specific demethylase 2A | -1.538248269 | 1.538248269 | down | 0.041802976 | 0.623333169 |
| MAP3K7 | mitogen-activated protein kinase kinase kinase 7 | -1.53846864 | 1.53846864 | down | 0.046826343 | 0.627322764 |
| SLC39A10 | solute carrier family 39 (zinc transporter), member 10 | -1.53852196 | 1.53852196 | down | 0.019568987 | 0.546194159 |
| ACSL3 | acyl-CoA synthetase long-chain family member 3 | -1.538625051 | 1.538625051 | down | 0.018757624 | 0.539772279 |
| FAM171A1 | family with sequence similarity 171 member A1 | -1.538667711 | 1.538667711 | down | 0.038239204 | 0.612402879 |
| MRPL42 | mitochondrial ribosomal protein L42 | -1.538785033 | 1.538785033 | down | 0.043924802 | 0.623333169 |
| SNRNP70 | small nuclear ribonucleoprotein, U1 70kDa subunit | -1.538813476 | 1.538813476 | down | 0.01238633 | 0.503186668 |
| TXNL1 | thioredoxin like 1 | -1.539542509 | 1.539542509 | down | 0.021363149 | 0.549146 |
| SEC61A1 | Sec61 translocon alpha 1 subunit | -1.539837776 | 1.539837776 | down | 0.028826948 | 0.574350948 |
| KANSL2 | KAT8 regulatory NSL complex subunit 2 | -1.539944513 | 1.539944513 | down | 0.031492457 | 0.587257488 |
| NAA20 | N(alpha)-acetyltransferase 20, NatB catalytic subunit | -1.539962303 | 1.539962303 | down | 0.038976806 | 0.613717043 |
| PGP | phosphoglycolate phosphatase | -1.540343064 | 1.540343064 | down | 0.012083067 | 0.498584823 |
| GPR89A | G protein-coupled receptor 89A | -1.540510343 | 1.540510343 | down | 0.031398675 | 0.58634196 |
| GPR89B | G protein-coupled receptor 89B | -1.540510343 | 1.540510343 | down | 0.031398675 | 0.58634196 |
| DHX36 | DEAH (Asp-Glu-Ala-His) box polypeptide 36 | -1.54052814 | 1.54052814 | down | 0.039189401 | 0.614613629 |
| DUT | deoxyuridine triphosphatase | -1.540542378 | 1.540542378 | down | 0.017001633 | 0.530571823 |
| HIF1A | hypoxia inducible factor 1, alpha subunit (basic helix-loop-helix transcription factor) | -1.540670522 | 1.540670522 | down | 0.025207924 | 0.565656951 |
| ABL2 | ABL proto-oncogene 2, non-receptor tyrosine kinase | -1.541079942 | 1.541079942 | down | 0.023997399 | 0.562265417 |
| THAP7 | THAP domain containing 7 | -1.541236619 | 1.541236619 | down | 0.046738893 | 0.627185789 |
| TMEM181 | transmembrane protein 181 | -1.541297157 | 1.541297157 | down | 0.042865091 | 0.623333169 |
| RHOC | ras homolog family member C | -1.541347014 | 1.541347014 | down | 0.028141163 | 0.57421459 |
| VARS | valyl-tRNA synthetase | -1.541361259 | 1.541361259 | down | 0.024551246 | 0.563613185 |
| GTF2H2B | general transcription factor IIH subunit 2B (pseudogene) | -1.541546458 | 1.541546458 | down | 0.026983738 | 0.568317266 |
| GTF2H2C_2 | GTF2H2 family member C, copy 2 | -1.541546458 | 1.541546458 | down | 0.026983738 | 0.568317266 |
| GTF2H2C | GTF2H2 family member C | -1.541546458 | 1.541546458 | down | 0.026983738 | 0.568317266 |
| GTF2H2 | general transcription factor IIH subunit 2 | -1.541546458 | 1.541546458 | down | 0.026983738 | 0.568317266 |
| PAIP2 | poly(A) binding protein interacting protein 2 | -1.541756614 | 1.541756614 | down | 0.041456225 | 0.623333169 |
| UBR4 | ubiquitin protein ligase E3 component n-recognin 4 | -1.541874172 | 1.541874172 | down | 0.02005322 | 0.549146 |
| YWHAH | tyrosine 3-monooxygenase/tryptophan 5-monooxygenase activation | -1.541970362 | 1.541970362 | down | 0.011741659 | 0.495320532 |
| PSMG1 | proteasome (prosome, macropain) assembly chaperone 1 | -1.542262532 | 1.542262532 | down | 0.014743331 | 0.515525808 |
| AP5Z1 | adaptor-related protein complex 5, zeta 1 subunit | -1.542376564 | 1.542376564 | down | 0.043556902 | 0.623333169 |
| POLR2L | polymerase (RNA) II (DNA directed) polypeptide L, 7.6kDa | -1.542419329 | 1.542419329 | down | 0.008410873 | 0.4606964 |
| CRAT | carnitine O-acetyltransferase | -1.542611783 | 1.542611783 | down | 0.042409715 | 0.623333169 |
| PLEKHJ1 | pleckstrin homology domain containing J1 | -1.542615347 | 1.542615347 | down | 0.043518272 | 0.623333169 |
| GMPS | guanine monophosphate synthase | -1.542775744 | 1.542775744 | down | 0.009557811 | 0.475470418 |
| SAFB | scaffold attachment factor B | -1.542800697 | 1.542800697 | down | 0.049718324 | 0.630325871 |

| | | | | | | |
|---|---|---|---|---|---|---|
| ZFAND5 | zinc finger, AN1-type domain 5 | -1.542911204 | 1.542911204 | down | 0.021943401 | 0.550085548 |
| TMBIM1 | transmembrane BAX inhibitor motif containing 1 | -1.542975373 | 1.542975373 | down | 0.027239745 | 0.568317266 |
| ARNTL2 | aryl hydrocarbon receptor nuclear translocator like 2 | -1.543485257 | 1.543485257 | down | 0.040160058 | 0.621238987 |
| PTP4A2 | protein tyrosine phosphatase type IVA, member 2 | -1.543513787 | 1.543513787 | down | 0.038867503 | 0.613615561 |
| SSRP1 | structure specific recognition protein 1 | -1.54359938 | 1.54359938 | down | 0.028021186 | 0.573304608 |
| SAV1 | salvador family WW domain containing protein 1 | -1.543795548 | 1.543795548 | down | 0.042361981 | 0.623333169 |
| MYDGF | myeloid-derived growth factor | -1.543866888 | 1.543866888 | down | 0.015214065 | 0.518081034 |
| VTA1 | vesicle (multivesicular body) trafficking 1 | -1.543941799 | 1.543941799 | down | 0.027184708 | 0.568317266 |
| OSBP | oxysterol binding protein | -1.544113037 | 1.544113037 | down | 0.039520276 | 0.616906565 |
| RBM14 | RNA binding motif protein 14 | -1.544223638 | 1.544223638 | down | 0.014933502 | 0.516117046 |
| TFG | TRK-fused gene | -1.544691106 | 1.544691106 | down | 0.025870356 | 0.566523191 |
| PSMA3 | proteasome subunit alpha 3 | -1.544733934 | 1.544733934 | down | 0.047516526 | 0.627469987 |
| FARSB | phenylalanyl-tRNA synthetase beta subunit | -1.544766057 | 1.544766057 | down | 0.02051831 | 0.549146 |
| CKS1B | CDC28 protein kinase regulatory subunit 1B | -1.545126585 | 1.545126585 | down | 0.027411847 | 0.568462255 |
| NDC1 | NDC1 transmembrane nucleoporin | -1.545237258 | 1.545237258 | down | 0.044583893 | 0.623333169 |
| TMEM222 | transmembrane protein 222 | -1.545333658 | 1.545333658 | down | 0.044330437 | 0.623333169 |
| SS18L2 | synovial sarcoma translocation gene on chromosome 18-like 2 | -1.545712176 | 1.545712176 | down | 0.015176895 | 0.518081034 |
| LAMTOR2 | late endosomal/lysosomal adaptor, MAPK and MTOR activator 2 | -1.545733604 | 1.545733604 | down | 0.046762334 | 0.627185789 |
| COMMD6 | COMM domain containing 6 | -1.546001482 | 1.546001482 | down | 0.044837061 | 0.623333169 |
| ERBB3 | erb-b2 receptor tyrosine kinase 3 | -1.546037203 | 1.546037203 | down | 0.049683471 | 0.630325871 |
| DYNC1LI2 | dynein, cytoplasmic 1, light intermediate chain 2 | -1.546072924 | 1.546072924 | down | 0.036220169 | 0.603150244 |
| PSMD2 | proteasome 26S subunit, non-ATPase 2 | -1.546487354 | 1.546487354 | down | 0.025522109 | 0.566329497 |
| PAFAH1B1 | platelet activating factor acetylhydrolase 1b regulatory subunit 1 | -1.546619566 | 1.546619566 | down | 0.018799131 | 0.539772279 |
| TEAD4 | TEA domain family member 4 | -1.546783953 | 1.546783953 | down | 0.020326232 | 0.549146 |
| GPR180 | G protein-coupled receptor 180 | -1.547023418 | 1.547023418 | down | 0.035463581 | 0.600698736 |
| PTPRM | protein tyrosine phosphatase, receptor type, M | -1.547119929 | 1.547119929 | down | 0.035065517 | 0.600153938 |
| TINF2 | TERF1 (TRF1)-interacting nuclear factor 2 | -1.547198573 | 1.547198573 | down | 0.021886988 | 0.550085548 |
| PTPMT1 | protein tyrosine phosphatase, mitochondrial 1 | -1.54731297 | 1.54731297 | down | 0.044336685 | 0.623333169 |
| CCDC51 | coiled-coil domain containing 51 | -1.547484582 | 1.547484582 | down | 0.039445808 | 0.616428841 |
| WDR44 | WD repeat domain 44 | -1.547895813 | 1.547895813 | down | 0.044194908 | 0.623333169 |
| ARHGEF16 | Rho guanine nucleotide exchange factor 16 | -1.54796019 | 1.54796019 | down | 0.048831424 | 0.62943979 |
| GTF2H5 | general transcription factor IIH subunit 5 | -1.548020992 | 1.548020992 | down | 0.046918073 | 0.627322764 |
| STIL | SCL/TAL1 interrupting locus | -1.54806749 | 1.54806749 | down | 0.044633818 | 0.623333169 |
| DPH1 | diphthamide biosynthesis 1 | -1.548149759 | 1.548149759 | down | 0.028977481 | 0.574540338 |
| OVCA2 | ovarian tumor suppressor candidate 2 | -1.548149759 | 1.548149759 | down | 0.028977481 | 0.574540338 |
| MLLT4 | myeloid/lymphoid or mixed-lineage leukemia; translocated to, 4 | -1.548174798 | 1.548174798 | down | 0.029241591 | 0.576629742 |
| PI4KB | phosphatidylinositol 4-kinase, catalytic, beta | -1.548264226 | 1.548264226 | down | 0.046550257 | 0.627185789 |
| DNAJA1 | DnaJ heat shock protein family (Hsp40) member A1 | -1.548267804 | 1.548267804 | down | 0.02949314 | 0.576629742 |
| ARID5B | AT-rich interaction domain 5B | -1.548299999 | 1.548299999 | down | 0.030156473 | 0.57950115 |
| GPATCH4 | G-patch domain containing 4 | -1.548303577 | 1.548303577 | down | 0.011629569 | 0.495320532 |
| UPF1 | UPF1 regulator of nonsense transcripts homolog (yeast) | -1.548403745 | 1.548403745 | down | 0.041267307 | 0.623333169 |
| AIMP2 | aminoacyl tRNA synthetase complex-interacting multifunctional protein 2 | -1.548439522 | 1.548439522 | down | 0.007640341 | 0.443884559 |
| GTPBP4 | GTP binding protein 4 | -1.548543277 | 1.548543277 | down | 0.007102658 | 0.434730278 |
| ANKRD13A | ankyrin repeat domain 13A | -1.548697134 | 1.548697134 | down | 0.005494469 | 0.401886731 |
| RABGEF1 | RAB guanine nucleotide exchange factor (GEF) 1 | -1.54872576 | 1.54872576 | down | 0.034488676 | 0.596656508 |
| KCTD7 | potassium channel tetramerization domain containing 7 | -1.54872576 | 1.54872576 | down | 0.034488676 | 0.596656508 |
| TRMT2A | tRNA methyltransferase 2 homolog A | -1.54879375 | 1.54879375 | down | 0.040047462 | 0.621145923 |
| CTNNAL1 | catenin alpha-like 1 | -1.548961947 | 1.548961947 | down | 0.016880809 | 0.530489988 |
| NR2F6 | nuclear receptor subfamily 2 group F member 6 | -1.549123004 | 1.549123004 | down | 0.008644531 | 0.466623412 |
| KANSL3 | KAT8 regulatory NSL complex subunit 3 | -1.549237544 | 1.549237544 | down | 0.04045452 | 0.623005985 |
| FAM20C | family with sequence similarity 20 member C | -1.54940221 | 1.54940221 | down | 0.02339267 | 0.556698922 |
| TPBG | trophoblast glycoprotein | -1.549577634 | 1.549577634 | down | 0.044209351 | 0.623333169 |
| MGAT2 | mannosyl (alpha-1,6-)-glycoprotein beta-1,2-N-acetylglucosaminyltransferase | -1.549577634 | 1.549577634 | down | 0.022482998 | 0.551250506 |
| TMEM206 | transmembrane protein 206 | -1.549609857 | 1.549609857 | down | 0.048703407 | 0.62943979 |

| | | | | | | |
|---|---|---|---|---|---|---|
| PROSC | proline synthetase co-transcribed homolog (bacterial) | -1.549685046 | 1.549685046 | down | 0.034927106 | 0.599159228 |
| TCEA2 | transcription elongation factor A (SII), 2 | -1.549753078 | 1.549753078 | down | 0.014539623 | 0.515525808 |
| BRMS1 | breast cancer metastasis suppressor 1 | -1.549896312 | 1.549896312 | down | 0.010882069 | 0.489568753 |
| YWHAQ | tyrosine 3-monooxygenase/tryptophan 5-monooxygenase activation protein, theta | -1.549967934 | 1.549967934 | down | 0.028891881 | 0.574350948 |
| CSTB | cystatin B (stefin B) | -1.550010909 | 1.550010909 | down | 0.015528843 | 0.52192494 |
| NSFL1C | NSFL1 (p97) cofactor (p47) | -1.550032397 | 1.550032397 | down | 0.026962769 | 0.568317266 |
| MED15 | mediator complex subunit 15 | -1.550043141 | 1.550043141 | down | 0.032545429 | 0.590424587 |
| LINC01000 | long intergenic non-protein coding RNA 1000 | -1.55033684 | 1.55033684 | down | 0.048131981 | 0.627892392 |
| NPIPB11 | nuclear pore complex interacting protein family member B11 | -1.55054103 | 1.55054103 | down | 0.015962706 | 0.523501532 |
| NPIPB5 | nuclear pore complex interacting protein family, member B5 | -1.55054103 | 1.55054103 | down | 0.015962706 | 0.523501532 |
| NPIPB4 | nuclear pore complex interacting protein family member B4 | -1.55054103 | 1.55054103 | down | 0.015962706 | 0.523501532 |
| LOC105369248 | nuclear pore complex-interacting protein family member B5-like | -1.55054103 | 1.55054103 | down | 0.015962706 | 0.523501532 |
| NPIPB3 | nuclear pore complex interacting protein family, member B3 | -1.55054103 | 1.55054103 | down | 0.015962706 | 0.523501532 |
| AGFG1 | ArfGAP with FG repeats 1 | -1.550691502 | 1.550691502 | down | 0.040632007 | 0.623259064 |
| ANKRD10 | ankyrin repeat domain 10 | -1.551056997 | 1.551056997 | down | 0.041480481 | 0.623333169 |
| RAE1 | ribonucleic acid export 1 | -1.551200352 | 1.551200352 | down | 0.002464556 | 0.306048075 |
| RTN4 | reticulon 4 | -1.551243361 | 1.551243361 | down | 0.003668981 | 0.349787905 |
| TPD52 | tumor protein D52 | -1.551275618 | 1.551275618 | down | 0.026414913 | 0.568317266 |
| GBP3 | guanylate binding protein 3 | -1.551401071 | 1.551401071 | down | 0.037400904 | 0.608981156 |
| SLC25A3 | solute carrier family 25 (mitochondrial carrier; phosphate carrier), member 3 | -1.551533703 | 1.551533703 | down | 0.038757189 | 0.613615561 |
| IPO5 | importin 5 | -1.551555212 | 1.551555212 | down | 0.045334904 | 0.624555255 |
| FAM168B | family with sequence similarity 168 member B | -1.551573136 | 1.551573136 | down | 0.010332816 | 0.485753032 |
| MCFD2 | multiple coagulation factor deficiency 2 | -1.551576721 | 1.551576721 | down | 0.044572411 | 0.623333169 |
| UBAC2 | UBA domain containing 2 | -1.551684272 | 1.551684272 | down | 0.045395072 | 0.624598741 |
| TERF2IP | telomeric repeat binding factor 2, interacting protein | -1.55173088 | 1.55173088 | down | 0.044541875 | 0.623333169 |
| FNDC3A | fibronectin type III domain containing 3A | -1.551824099 | 1.551824099 | down | 0.049694274 | 0.630325871 |
| ABI1 | abl-interactor 1 | -1.551877882 | 1.551877882 | down | 0.023209738 | 0.55642591 |
| RAF1 | Raf-1 proto-oncogene, serine/threonine kinase | -1.552039242 | 1.552039242 | down | 0.018457698 | 0.537128348 |
| PPIB | peptidylprolyl isomerase B (cyclophilin B) | -1.552078689 | 1.552078689 | down | 0.004123008 | 0.358673662 |
| NMU | neuromedin U | -1.552118136 | 1.552118136 | down | 0.015773252 | 0.522063677 |
| TXNDC9 | thioredoxin domain containing 9 | -1.552153998 | 1.552153998 | down | 0.045622547 | 0.625770788 |
| APLP2 | amyloid beta (A4) precursor-like protein 2 | -1.552440923 | 1.552440923 | down | 0.001976686 | 0.276804943 |
| SLU7 | SLU7 homolog, splicing factor | -1.552548534 | 1.552548534 | down | 0.020672035 | 0.549146 |
| WHAMM | WAS protein homolog associated with actin, golgi membranes and microtubules | -1.552577232 | 1.552577232 | down | 0.038080463 | 0.612253826 |
| CTDNEP1 | CTD nuclear envelope phosphatase 1 | -1.552853472 | 1.552853472 | down | 0.030244257 | 0.579778515 |
| GRPEL2 | GrpE-like 2, mitochondrial (E. coli) | -1.552878587 | 1.552878587 | down | 0.018131428 | 0.536306821 |
| METTL17 | methyltransferase like 17 | -1.55304364 | 1.55304364 | down | 0.032817198 | 0.590424587 |
| PPIL2 | peptidylprolyl isomerase like 2 | -1.553241008 | 1.553241008 | down | 0.048936954 | 0.62943979 |
| LGALS8 | lectin, galactoside-binding, soluble, 8 | -1.553248186 | 1.553248186 | down | 0.020113985 | 0.549146 |
| MYO5B | myosin VB | -1.553273307 | 1.553273307 | down | 0.031942407 | 0.589351082 |
| KPNB1 | karyopherin (importin) beta 1 | -1.553305607 | 1.553305607 | down | 0.047263456 | 0.627322764 |
| YTHDF2 | YTH N(6)-methyladenosine RNA binding protein 2 | -1.553413278 | 1.553413278 | down | 0.03915237 | 0.614281799 |
| CCDC86 | coiled-coil domain containing 86 | -1.553420456 | 1.553420456 | down | 0.032565144 | 0.590424587 |
| POMP | proteasome maturation protein | -1.553581977 | 1.553581977 | down | 0.049711664 | 0.630325871 |
| SAC3D1 | SAC3 domain containing 1 | -1.553761464 | 1.553761464 | down | 0.025405347 | 0.565656951 |
| ITGA3 | integrin alpha 3 | -1.553829674 | 1.553829674 | down | 0.043415621 | 0.623333169 |
| REPS1 | RALBP1 associated Eps domain containing 1 | -1.553991238 | 1.553991238 | down | 0.033691167 | 0.593014207 |
| HIST2H4A | histone cluster 2, H4a | -1.554016371 | 1.554016371 | down | 0.013302529 | 0.509292857 |
| HIST2H4B | histone cluster 2, H4b | -1.554016371 | 1.554016371 | down | 0.013302529 | 0.509292857 |
| LOC102724364 | vesicle-trafficking protein SEC22b-like | -1.55416 | 1.55416 | down | 0.025206313 | 0.565656951 |
| SEC22B | SEC22 homolog B, vesicle trafficking protein (gene/pseudogene) | -1.55416 | 1.55416 | down | 0.025206313 | 0.565656951 |
| LOC100996716 | vesicle-trafficking protein SEC22b-like | -1.55416 | 1.55416 | down | 0.025206313 | 0.565656951 |
| LOC100996517 | vesicle-trafficking protein SEC22b-like | -1.55416 | 1.55416 | down | 0.025206313 | 0.565656951 |
| PRPF38A | pre-mRNA processing factor 38A | -1.554174363 | 1.554174363 | down | 0.017412392 | 0.531030242 |

| | | | | | | |
|---|---|---|---|---|---|---|
| ZFAND2A | zinc finger, AN1-type domain 2A | -1.554217455 | 1.554217455 | down | 0.00930489 | 0.470801192 |
| SRSF10 | serine/arginine-rich splicing factor 10 | -1.554274912 | 1.554274912 | down | 0.022802691 | 0.551784312 |
| BAD | BCL2 associated agonist of cell death | -1.554274912 | 1.554274912 | down | 0.036332356 | 0.603215176 |
| ARGLU1 | arginine and glutamate rich 1 | -1.554386241 | 1.554386241 | down | 0.008934836 | 0.466623412 |
| DLGAP4 | discs, large (Drosophila) homolog-associated protein 4 | -1.554458071 | 1.554458071 | down | 0.029989635 | 0.579152964 |
| KPNA4 | karyopherin alpha 4 (importin alpha 3) | -1.554594556 | 1.554594556 | down | 0.005654093 | 0.402842701 |
| GRWD1 | glutamate-rich WD repeat containing 1 | -1.554655619 | 1.554655619 | down | 0.048498594 | 0.62898126 |
| MAGOH | mago homolog, exon junction complex core component | -1.554731053 | 1.554731053 | down | 0.016253992 | 0.524267534 |
| ADI1 | acireductone dioxygenase 1 | -1.554738238 | 1.554738238 | down | 0.012382156 | 0.503186668 |
| EDEM1 | ER degradation enhancer, mannosidase alpha-like 1 | -1.554810083 | 1.554810083 | down | 0.02652483 | 0.568317266 |
| CHPF2 | chondroitin polymerizing factor 2 | -1.555086721 | 1.555086721 | down | 0.029788161 | 0.578781315 |
| TFDP2 | transcription factor Dp-2 (E2F dimerization partner 2) | -1.555119058 | 1.555119058 | down | 0.032200194 | 0.589852866 |
| NOC2L | NOC2-like nucleolar associated transcriptional repressor | -1.555216075 | 1.555216075 | down | 0.026587185 | 0.568317266 |
| ZC3HAV1 | zinc finger CCCH-type, antiviral 1 | -1.555248415 | 1.555248415 | down | 0.030524567 | 0.581882081 |
| LRRFIP2 | leucine rich repeat (in FLII) interacting protein 2 | -1.555259195 | 1.555259195 | down | 0.03677209 | 0.605213936 |
| KRIT1 | KRIT1, ankyrin repeat containing | -1.555313097 | 1.555313097 | down | 0.035757588 | 0.60230957 |
| TSC22D2 | TSC22 domain family member 2 | -1.555467627 | 1.555467627 | down | 0.030521918 | 0.581882081 |
| TLDC1 | TBC/LysM-associated domain containing 1 | -1.555888169 | 1.555888169 | down | 0.031317494 | 0.586144908 |
| RBMX | RNA binding motif protein, X-linked | -1.555909739 | 1.555909739 | down | 0.016190411 | 0.524267534 |
| RBMXL1 | RNA binding motif protein, X-linked-like 1 | -1.555909739 | 1.555909739 | down | 0.016190411 | 0.524267534 |
| SNW1 | SNW domain containing 1 | -1.556129044 | 1.556129044 | down | 0.042014441 | 0.623333169 |
| EDF1 | endothelial differentiation-related factor 1 | -1.556251293 | 1.556251293 | down | 0.004670706 | 0.377936677 |
| KIFC1 | kinesin family member C1 | -1.55626208 | 1.55626208 | down | 0.041725148 | 0.623333169 |
| PKMYT1 | protein kinase, membrane associated tyrosine/threonine 1 | -1.5563304 | 1.5563304 | down | 0.030539016 | 0.581882081 |
| RAD23A | RAD23 homolog A, nucleotide excision repair protein | -1.556351976 | 1.556351976 | down | 0.03178915 | 0.589351082 |
| ING1 | inhibitor of growth family member 1 | -1.556481435 | 1.556481435 | down | 0.040863449 | 0.623333169 |
| TIMM9 | translocase of inner mitochondrial membrane 9 homolog (yeast) | -1.556485031 | 1.556485031 | down | 0.01864298 | 0.539455255 |
| SDF2L1 | stromal cell-derived factor 2-like 1 | -1.556520994 | 1.556520994 | down | 0.001208005 | 0.23896047 |
| CDK8 | cyclin-dependent kinase 8 | -1.556837503 | 1.556837503 | down | 0.034800451 | 0.598019712 |
| CENPN | centromere protein N | -1.556909446 | 1.556909446 | down | 0.01145638 | 0.494496053 |
| FOXRED1 | FAD-dependent oxidoreductase domain containing 1 | -1.556941821 | 1.556941821 | down | 0.044305387 | 0.623333169 |
| ZNF410 | zinc finger protein 410 | -1.557013769 | 1.557013769 | down | 0.031851545 | 0.589351082 |
| LIN52 | lin-52 DREAM MuvB core complex component | -1.557348369 | 1.557348369 | down | 0.048313034 | 0.627892392 |
| IER5 | immediate early response 5 | -1.557445525 | 1.557445525 | down | 0.021350373 | 0.549146 |
| UBL7 | ubiquitin like 7 | -1.557783817 | 1.557783817 | down | 0.018415766 | 0.537128348 |
| LDHB | lactate dehydrogenase B | -1.558089783 | 1.558089783 | down | 0.018207689 | 0.536306821 |
| YIPF1 | Yip1 domain family member 1 | -1.558111383 | 1.558111383 | down | 0.017526416 | 0.531030242 |
| FPGS | folylpolyglutamate synthase | -1.558251789 | 1.558251789 | down | 0.013294343 | 0.509292857 |
| HNRNPA1 | heterogeneous nuclear ribonucleoprotein A1 | -1.558251789 | 1.558251789 | down | 0.029379401 | 0.576629742 |
| POLR1D | polymerase (RNA) I polypeptide D | -1.558388607 | 1.558388607 | down | 0.038576325 | 0.61336314 |
| PI3 | peptidase inhibitor 3, skin-derived | -1.558442618 | 1.558442618 | down | 0.008754604 | 0.466623412 |
| GOLM1 | golgi membrane protein 1 | -1.558583054 | 1.558583054 | down | 0.015293722 | 0.51989411 |
| RALY | RALY heterogeneous nuclear ribonucleoprotein | -1.558687489 | 1.558687489 | down | 0.048002594 | 0.627854743 |
| S100A13 | S100 calcium binding protein A13 | -1.558701895 | 1.558701895 | down | 0.001815339 | 0.265169566 |
| RBBP6 | retinoblastoma binding protein 6 | -1.559000837 | 1.559000837 | down | 0.020518109 | 0.549146 |
| RPL22L1 | ribosomal protein L22-like 1 | -1.559087289 | 1.559087289 | down | 0.013368517 | 0.509292857 |
| PARP2 | poly(ADP-ribose) polymerase 2 | -1.559249399 | 1.559249399 | down | 0.045654024 | 0.625770788 |
| THEM6 | thioesterase superfamily member 6 | -1.55934307 | 1.55934307 | down | 0.031937068 | 0.589351082 |
| SREBF1 | sterol regulatory element binding transcription factor 1 | -1.559397113 | 1.559397113 | down | 0.038597165 | 0.61339418 |
| ARID3B | AT-rich interaction domain 3B | -1.559436747 | 1.559436747 | down | 0.043915285 | 0.623333169 |
| DESI1 | desumoylating isopeptidase 1 | -1.559454762 | 1.559454762 | down | 0.025040801 | 0.565551322 |
| TSPAN1 | tetraspanin 1 | -1.559620514 | 1.559620514 | down | 0.011824442 | 0.495320532 |
| DDX24 | DEAD (Asp-Glu-Ala-Asp) box helicase 24 | -1.559688982 | 1.559688982 | down | 0.013966397 | 0.511949108 |
| MFSD11 | major facilitator superfamily domain containing 11 | -1.559753848 | 1.559753848 | down | 0.047589252 | 0.62776393 |

| | | | | | | |
|---|---|---|---|---|---|---|
| R3HCC1L | R3H domain and coiled-coil containing 1 like | -1.559890799 | 1.559890799 | down | 0.045795757 | 0.626389846 |
| ADK | adenosine kinase | -1.560081828 | 1.560081828 | down | 0.005013366 | 0.38925276 |
| TSPO | translocator protein | -1.560626211 | 1.560626211 | down | 0.015709171 | 0.522063677 |
| KCNK1 | potassium channel, two pore domain subfamily K, member 1 | -1.560723571 | 1.560723571 | down | 0.03753814 | 0.609356245 |
| NAA15 | N(alpha)-acetyltransferase 15, NatA auxiliary subunit | -1.560774056 | 1.560774056 | down | 0.0137811 | 0.511408446 |
| OSMR | oncostatin M receptor | -1.560795694 | 1.560795694 | down | 0.048709833 | 0.62943979 |
| MRPS7 | mitochondrial ribosomal protein S7 | -1.561004867 | 1.561004867 | down | 0.003376807 | 0.338252205 |
| ICT1 | immature colon carcinoma transcript 1 | -1.561015687 | 1.561015687 | down | 0.035437429 | 0.600698736 |
| TRIM13 | tripartite motif containing 13 | -1.561174391 | 1.561174391 | down | 0.022965071 | 0.554273475 |
| TIAL1 | TIA1 cytotoxic granule-associated RNA binding protein-like 1 | -1.561185212 | 1.561185212 | down | 0.047336662 | 0.627322764 |
| TBL3 | transducin (beta)-like 3 | -1.561188819 | 1.561188819 | down | 0.018664754 | 0.539455255 |
| EIF3A | eukaryotic translation initiation factor 3 subunit A | -1.561192426 | 1.561192426 | down | 0.013638655 | 0.510833181 |
| MRFAP1L1 | Morf4 family associated protein 1-like 1 | -1.56123932 | 1.56123932 | down | 0.049619859 | 0.630325871 |
| NCK1 | NCK adaptor protein 1 | -1.56134754 | 1.56134754 | down | 0.036221191 | 0.603150244 |
| SYAP1 | synapse associated protein 1 | -1.56134754 | 1.56134754 | down | 0.022198484 | 0.550353494 |
| INF2 | inverted formin, FH2 and WH2 domain containing | -1.5613764 | 1.5613764 | down | 0.034740388 | 0.598002352 |
| MXRA8 | matrix-remodelling associated 8 | -1.56155318 | 1.56155318 | down | 0.031002528 | 0.584489712 |
| MT2A | metallothionein 2A | -1.561654206 | 1.561654206 | down | 0.000664267 | 0.18477288 |
| MB21D2 | Mab-21 domain containing 2 | -1.561766064 | 1.561766064 | down | 0.011888545 | 0.495320532 |
| MIDN | midnolin | -1.56200063 | 1.56200063 | down | 0.029108803 | 0.57512885 |
| LDLR | low density lipoprotein receptor | -1.562130559 | 1.562130559 | down | 0.026584573 | 0.568317266 |
| NPEPPS | aminopeptidase puromycin sensitive | -1.562148606 | 1.562148606 | down | 0.003215685 | 0.333998479 |
| CNBP | CCHC-type zinc finger, nucleic acid binding protein | -1.562173871 | 1.562173871 | down | 0.043778577 | 0.623333169 |
| MEPCE | methylphosphate capping enzyme | -1.562220794 | 1.562220794 | down | 0.026004608 | 0.567499406 |
| IQCB1 | IQ motif containing B1 | -1.562422939 | 1.562422939 | down | 0.03819638 | 0.612253826 |
| NOP16 | NOP16 nucleolar protein | -1.562722595 | 1.562722595 | down | 0.026073871 | 0.567622316 |
| C15orf52 | chromosome 15 open reading frame 52 | -1.562964527 | 1.562964527 | down | 0.018880556 | 0.539906054 |
| LIN37 | lin-37 DREAM MuvB core complex component | -1.563015085 | 1.563015085 | down | 0.035161159 | 0.600433528 |
| TWF1 | twinfilin actin binding protein 1 | -1.563213721 | 1.563213721 | down | 0.032844372 | 0.590643545 |
| UBE2J1 | ubiquitin conjugating enzyme E2, J1 | -1.563246228 | 1.563246228 | down | 0.005340204 | 0.399652337 |
| PSMD1 | proteasome 26S subunit, non-ATPase 1 | -1.563643583 | 1.563643583 | down | 0.006046041 | 0.414619018 |
| FZD2 | frizzled class receptor 2 | -1.563658035 | 1.563658035 | down | 0.012633598 | 0.5051069 |
| MGAT1 | mannosyl (alpha-1,3-)-glycoprotein beta-1,2-N-acetylglucosaminyltransferase | -1.563712228 | 1.563712228 | down | 0.028421524 | 0.57421459 |
| PIP5K1C | phosphatidylinositol-4-phosphate 5-kinase, type I, gamma | -1.563748358 | 1.563748358 | down | 0.039087848 | 0.613747319 |
| HIST1H2BD | histone cluster 1, H2bd | -1.563979608 | 1.563979608 | down | 0.013362165 | 0.509292857 |
| GTF3C5 | general transcription factor IIIC subunit 5 | -1.563983222 | 1.563983222 | down | 0.007168614 | 0.434730278 |
| BTG1 | B-cell translocation gene 1, anti-proliferative | -1.564095247 | 1.564095247 | down | 0.011309372 | 0.491885982 |
| PWWP2B | PWWP domain containing 2B | -1.564138613 | 1.564138613 | down | 0.033152383 | 0.59236436 |
| HNRNPF | heterogeneous nuclear ribonucleoprotein F | -1.564171139 | 1.564171139 | down | 0.027255126 | 0.568317266 |
| ABCE1 | ATP binding cassette subfamily E member 1 | -1.564192823 | 1.564192823 | down | 0.040110784 | 0.621238987 |
| VPS33B | vacuolar protein sorting 33 homolog B (yeast) | -1.56431932 | 1.56431932 | down | 0.035044166 | 0.600153938 |
| DCPS | decapping enzyme, scavenger | -1.564359078 | 1.564359078 | down | 0.041088444 | 0.623333169 |
| LMF2 | lipase maturation factor 2 | -1.564387994 | 1.564387994 | down | 0.027672893 | 0.570815756 |
| HRAS | Harvey rat sarcoma viral oncogene homolog | -1.564644645 | 1.564644645 | down | 0.04073908 | 0.623259064 |
| CKB | creatine kinase, brain | -1.564854334 | 1.564854334 | down | 0.02154905 | 0.550085548 |
| KIAA0368 | KIAA0368 | -1.565147224 | 1.565147224 | down | 0.01912323 | 0.541887884 |
| CASC4 | cancer susceptibility candidate 4 | -1.565183387 | 1.565183387 | down | 0.04649862 | 0.627185789 |
| ABHD17C | abhydrolase domain containing 17C | -1.565215934 | 1.565215934 | down | 0.013590592 | 0.5101407 |
| SLC31A2 | solute carrier family 31 (copper transporter), member 2 | -1.565306347 | 1.565306347 | down | 0.040780595 | 0.623333169 |
| GBE1 | glucan (1,4-alpha-), branching enzyme 1 | -1.565364214 | 1.565364214 | down | 0.033104404 | 0.592343125 |
| HSPA4 | heat shock protein family A (Hsp70) member 4 | -1.565411233 | 1.565411233 | down | 0.044379561 | 0.623333169 |
| AK4 | adenylate kinase 4 | -1.565483572 | 1.565483572 | down | 0.01844175 | 0.537128348 |
| AK4P1 | adenylate kinase 4 pseudogene 1 | -1.565483572 | 1.565483572 | down | 0.01844175 | 0.537128348 |
| ANXA2P2 | annexin A2 pseudogene 2 | -1.565602939 | 1.565602939 | down | 0.002551406 | 0.309987919 |

| | | | | | | |
|---|---|---|---|---|---|---|
| STK17A | serine/threonine kinase 17a | -1.565751256 | 1.565751256 | down | 0.037041019 | 0.606538982 |
| CAPN2 | calpain 2, (m/II) large subunit | -1.565765726 | 1.565765726 | down | 0.03778385 | 0.610825976 |
| LOC105369243 | sulfotransferase 1A3-like | -1.565939385 | 1.565939385 | down | 0.003906238 | 0.353492125 |
| SLX1A-SULT1A3 | SLX1A-SULT1A3 readthrough (NMD candidate) | -1.565939385 | 1.565939385 | down | 0.003906238 | 0.353492125 |
| SULT1A4 | sulfotransferase family 1A member 4 | -1.565939385 | 1.565939385 | down | 0.003906238 | 0.353492125 |
| SLX1B-SULT1A4 | SLX1B-SULT1A4 readthrough (NMD candidate) | -1.565939385 | 1.565939385 | down | 0.003906238 | 0.353492125 |
| SULT1A3 | sulfotransferase family 1A member 3 | -1.565939385 | 1.565939385 | down | 0.003906238 | 0.353492125 |
| REEP5 | receptor accessory protein 5 | -1.566113063 | 1.566113063 | down | 0.030915273 | 0.583508935 |
| PFDN5 | prefoldin subunit 5 | -1.566138392 | 1.566138392 | down | 0.01063506 | 0.486801343 |
| TMEM14B | transmembrane protein 14B | -1.566322949 | 1.566322949 | down | 0.014207488 | 0.514636149 |
| EXOC4 | exocyst complex component 4 | -1.566460476 | 1.566460476 | down | 0.047354598 | 0.627322764 |
| PDCD5 | programmed cell death 5 | -1.566648691 | 1.566648691 | down | 0.02424889 | 0.563333857 |
| WDR90 | WD repeat domain 90 | -1.566724707 | 1.566724707 | down | 0.020144543 | 0.549146 |
| NDUFA10 | NADH:ubiquinone oxidoreductase subunit A10 | -1.566757286 | 1.566757286 | down | 0.00295977 | 0.326391956 |
| PRMT5 | protein arginine methyltransferase 5 | -1.56686951 | 1.56686951 | down | 0.00294268 | 0.326391956 |
| EXOSC4 | exosome component 4 | -1.567003464 | 1.567003464 | down | 0.018478347 | 0.537153195 |
| NPLOC4 | NPL4 homolog, ubiquitin recognition factor | -1.567068635 | 1.567068635 | down | 0.022554478 | 0.551250506 |
| DROSHA | drosha, ribonuclease type III | -1.567072256 | 1.567072256 | down | 0.016830363 | 0.530351036 |
| RMND5A | required for meiotic nuclear division 5 homolog A | -1.567075877 | 1.567075877 | down | 0.028348456 | 0.57421459 |
| CHD4 | chromodomain helicase DNA binding protein 4 | -1.567086739 | 1.567086739 | down | 0.016331035 | 0.524267534 |
| FOXO3B | forkhead box O3B pseudogene | -1.567112084 | 1.567112084 | down | 0.034533034 | 0.596676967 |
| FOXO3 | forkhead box O3 | -1.567112084 | 1.567112084 | down | 0.034533034 | 0.596676967 |
| PTPN12 | protein tyrosine phosphatase, non-receptor type 12 | -1.567314862 | 1.567314862 | down | 0.045321613 | 0.624555255 |
| SMARCE1 | SWI/SNF related, matrix associated, actin dependent regulator of chromatin, subfamily e, member 1 | -1.567322105 | 1.567322105 | down | 0.037894032 | 0.611672854 |
| PKP4 | plakophilin 4 | -1.567488692 | 1.567488692 | down | 0.03028691 | 0.579885323 |
| ERCC6-PGBD3 | ERCC6-PGBD3 readthrough | -1.567528531 | 1.567528531 | down | 0.028271011 | 0.57421459 |
| PGBD3 | piggyBac transposable element derived 3 | -1.567528531 | 1.567528531 | down | 0.028271011 | 0.57421459 |
| MAX | MYC associated factor X | -1.567597346 | 1.567597346 | down | 0.04747861 | 0.627322764 |
| DDX39A | DEAD (Asp-Glu-Ala-Asp) box polypeptide 39A | -1.567615456 | 1.567615456 | down | 0.014288404 | 0.514676952 |
| TAF5 | TATA-box binding protein associated factor 5 | -1.567745852 | 1.567745852 | down | 0.03697754 | 0.606003692 |
| PRCC | papillary renal cell carcinoma (translocation-associated) | -1.567977694 | 1.567977694 | down | 0.00927258 | 0.470380292 |
| MED6 | mediator complex subunit 6 | -1.568129859 | 1.568129859 | down | 0.034809285 | 0.598019712 |
| EDEM3 | ER degradation enhancer, mannosidase alpha-like 3 | -1.568234934 | 1.568234934 | down | 0.021469135 | 0.549146 |
| MAP7D1 | MAP7 domain containing 1 | -1.568343639 | 1.568343639 | down | 0.022248299 | 0.550353494 |
| ARHGAP10 | Rho GTPase activating protein 10 | -1.5683835 | 1.5683835 | down | 0.02434138 | 0.563613185 |
| FRMD6 | FERM domain containing 6 | -1.568702421 | 1.568702421 | down | 0.01469383 | 0.515525808 |
| IER5L | immediate early response 5-like | -1.568706045 | 1.568706045 | down | 0.039070519 | 0.613731439 |
| FAM73B | family with sequence similarity 73 member B | -1.56888003 | 1.56888003 | down | 0.016367961 | 0.524267534 |
| ATP6V1G1 | ATPase, H+ transporting, lysosomal 13kDa, V1 subunit G1 | -1.568956154 | 1.568956154 | down | 0.00336853 | 0.338252205 |
| TRIM27 | tripartite motif containing 27 | -1.569006906 | 1.569006906 | down | 0.013660216 | 0.511154388 |
| FOXC1 | forkhead box C1 | -1.569133792 | 1.569133792 | down | 0.004001132 | 0.357261063 |
| ACOX1 | acyl-CoA oxidase 1, palmitoyl | -1.569463745 | 1.569463745 | down | 0.032648253 | 0.590424587 |
| CHMP2B | charged multivesicular body protein 2B | -1.56950726 | 1.56950726 | down | 0.027269255 | 0.568317266 |
| FKBPL | FK506 binding protein like | -1.569819156 | 1.569819156 | down | 0.043364144 | 0.623333169 |
| KLF5 | Kruppel-like factor 5 (intestinal) | -1.570200042 | 1.570200042 | down | 0.025767598 | 0.566329497 |
| DSE | dermatan sulfate epimerase | -1.57023995 | 1.57023995 | down | 0.010108794 | 0.480015301 |
| ENDOG | endonuclease G | -1.570276231 | 1.570276231 | down | 0.030750786 | 0.582533534 |
| UMPS | uridine monophosphate synthetase | -1.570312512 | 1.570312512 | down | 0.019181554 | 0.542443884 |
| C15orf48 | chromosome 15 open reading frame 48 | -1.570479418 | 1.570479418 | down | 0.047840769 | 0.627854743 |
| HMGA1 | high mobility group AT-hook 1 | -1.57089676 | 1.57089676 | down | 0.041699445 | 0.623333169 |
| DHX29 | DEAH (Asp-Glu-Ala-His) box polypeptide 29 | -1.571143588 | 1.571143588 | down | 0.019257457 | 0.543126657 |
| PALM2-AKAP2 | PALM2-AKAP2 readthrough | -1.57118715 | 1.57118715 | down | 0.002548268 | 0.309987919 |
| AKAP2 | A-kinase anchoring protein 2 | -1.57118715 | 1.57118715 | down | 0.002548268 | 0.309987919 |
| APEX2 | APEX nuclease (apurinic/apyrimidinic endonuclease) 2 | -1.571227083 | 1.571227083 | down | 0.010477945 | 0.486801343 |

| | | | | | | |
|---|---|---|---|---|---|---|
| IGFBP3 | insulin like growth factor binding protein 3 | -1.571372302 | 1.571372302 | down | 0.040374262 | 0.622535379 |
| GXYLT1 | glucoside xylosyltransferase 1 | -1.571510272 | 1.571510272 | down | 0.039246894 | 0.614613629 |
| SLC33A1 | solute carrier family 33 (acetyl-CoA transporter), member 1 | -1.571572 | 1.571572 | down | 0.020931423 | 0.549146 |
| CHEK1 | checkpoint kinase 1 | -1.571579262 | 1.571579262 | down | 0.011741392 | 0.495320532 |
| PIBF1 | progesterone immunomodulatory binding factor 1 | -1.571822566 | 1.571822566 | down | 0.049317128 | 0.630325871 |
| RPL7 | ribosomal protein L7 | -1.571887937 | 1.571887937 | down | 0.047204116 | 0.627322764 |
| MBNL2 | muscleblind-like splicing regulator 2 | -1.571971472 | 1.571971472 | down | 0.020117336 | 0.549146 |
| TMEM208 | transmembrane protein 208 | -1.572076804 | 1.572076804 | down | 0.006125123 | 0.414771056 |
| TPM2 | tropomyosin 2 (beta) | -1.572105863 | 1.572105863 | down | 0.003631327 | 0.349504153 |
| GATC | glutamyl-tRNA(Gln) amidotransferase, subunit C | -1.572113127 | 1.572113127 | down | 0.036790981 | 0.605213936 |
| ERCC3 | excision repair cross-complementation group 3 | -1.572200306 | 1.572200306 | down | 0.037390971 | 0.608981156 |
| RAC2 | ras-related C3 botulinum toxin substrate 2 (rho family, small GTP binding protein Rac2) | -1.572211204 | 1.572211204 | down | 0.015186465 | 0.518081034 |
| TM9SF1 | transmembrane 9 superfamily member 1 | -1.572229367 | 1.572229367 | down | 0.036473771 | 0.603912936 |
| RAPGEF2 | Rap guanine nucleotide exchange factor 2 | -1.572392843 | 1.572392843 | down | 0.014600257 | 0.515525808 |
| DCP2 | decapping mRNA 2 | -1.572425541 | 1.572425541 | down | 0.043621386 | 0.623333169 |
| HSPA1B | heat shock protein family A (Hsp70) member 1B | -1.572668975 | 1.572668975 | down | 0.045373571 | 0.624555255 |
| HSPA1A | heat shock protein family A (Hsp70) member 1A | -1.572668975 | 1.572668975 | down | 0.045373571 | 0.624555255 |
| EDARADD | EDAR-associated death domain | -1.572734382 | 1.572734382 | down | 0.000956465 | 0.216386548 |
| ENO1 | enolase 1, (alpha) | -1.572734382 | 1.572734382 | down | 0.000956465 | 0.216386548 |
| STAG3L1 | stromal antigen 3-like 1 (pseudogene) | -1.572937888 | 1.572937888 | down | 0.016503043 | 0.52581211 |
| STAG3L3 | stromal antigen 3-like 3 (pseudogene) | -1.572937888 | 1.572937888 | down | 0.016503043 | 0.52581211 |
| TUBGCP3 | tubulin, gamma complex associated protein 3 | -1.573145054 | 1.573145054 | down | 0.026585714 | 0.568317266 |
| FDPS | farnesyl diphosphate synthase | -1.573279545 | 1.573279545 | down | 0.004552318 | 0.373337469 |
| ZNF652 | zinc finger protein 652 | -1.573326801 | 1.573326801 | down | 0.01762968 | 0.53179491 |
| LSM14A | LSM14A mRNA processing body assembly factor | -1.573526748 | 1.573526748 | down | 0.020206261 | 0.549146 |
| STT3A | STT3A, subunit of the oligosaccharyltransferase complex (catalytic) | -1.57379217 | 1.57379217 | down | 0.006062361 | 0.414619018 |
| MAP4K3 | mitogen-activated protein kinase kinase kinase kinase 3 | -1.573973992 | 1.573973992 | down | 0.025397982 | 0.565656951 |
| TSPAN10 | tetraspanin 10 | -1.574264951 | 1.574264951 | down | 0.025919174 | 0.566523191 |
| HN1 | hematological and neurological expressed 1 | -1.574315875 | 1.574315875 | down | 0.008988258 | 0.466623412 |
| RTCA | RNA 3'-terminal phosphate cyclase | -1.574566878 | 1.574566878 | down | 0.014813675 | 0.515597089 |
| CLDN7 | claudin 7 | -1.574938 | 1.574938 | down | 0.003375091 | 0.338252205 |
| PSMC4 | proteasome 26S subunit, ATPase 4 | -1.574945278 | 1.574945278 | down | 0.005657002 | 0.402842701 |
| NR2C2AP | nuclear receptor 2C2-associated protein | -1.574967111 | 1.574967111 | down | 0.048182811 | 0.627892392 |
| ABCF2 | ATP binding cassette subfamily F member 2 | -1.57509448 | 1.57509448 | down | 0.01415013 | 0.514636149 |
| HAGH | hydroxyacylglutathione hydrolase | -1.575192742 | 1.575192742 | down | 0.031876787 | 0.589351082 |
| NDFIP2 | Nedd4 family interacting protein 2 | -1.575367446 | 1.575367446 | down | 0.048262931 | 0.627892392 |
| SNX19 | sorting nexin 19 | -1.575418405 | 1.575418405 | down | 0.020595019 | 0.549146 |
| POLE3 | polymerase (DNA directed), epsilon 3, accessory subunit | -1.575440246 | 1.575440246 | down | 0.008379986 | 0.4606964 |
| MAP2K7 | mitogen-activated protein kinase kinase 7 | -1.575607696 | 1.575607696 | down | 0.022349142 | 0.551238726 |
| MRPS27 | mitochondrial ribosomal protein S27 | -1.575847983 | 1.575847983 | down | 0.038540198 | 0.61336314 |
| ITGA6 | integrin alpha 6 | -1.576135647 | 1.576135647 | down | 0.016416418 | 0.524965293 |
| GLA | galactosidase alpha | -1.576288603 | 1.576288603 | down | 0.00898151 | 0.466623412 |
| SFPQ | splicing factor proline/glutamine-rich | -1.576430648 | 1.576430648 | down | 0.012809863 | 0.5057776 |
| SFPQ | splicing factor proline/glutamine-rich | -1.576430648 | 1.576430648 | down | 0.012809863 | 0.5057776 |
| METTL21A | methyltransferase like 21A | -1.576663774 | 1.576663774 | down | 0.027170656 | 0.568317266 |
| SPTBN1 | spectrin, beta, non-erythrocytic 1 | -1.57669656 | 1.57669656 | down | 0.02386554 | 0.561713077 |
| IGFBP4 | insulin like growth factor binding protein 4 | -1.576707489 | 1.576707489 | down | 0.011049034 | 0.489992559 |
| BRCC3 | BRCA1/BRCA2-containing complex subunit 3 | -1.576834998 | 1.576834998 | down | 0.017865682 | 0.534409263 |
| LIMA1 | LIM domain and actin binding 1 | -1.57713013 | 1.57713013 | down | 0.047667585 | 0.627854743 |
| RSL1D1 | ribosomal L1 domain containing 1 | -1.57714835 | 1.57714835 | down | 0.002586827 | 0.311888575 |
| SLCO2A1 | solute carrier organic anion transporter family member 2A1 | -1.577224875 | 1.577224875 | down | 0.0483436 | 0.627892392 |
| ZNF267 | zinc finger protein 267 | -1.577862733 | 1.577862733 | down | 0.028153785 | 0.57421459 |
| LDLRAD3 | low density lipoprotein receptor class A domain containing 3 | -1.577891898 | 1.577891898 | down | 0.03124192 | 0.585954505 |
| WDR13 | WD repeat domain 13 | -1.57795023 | 1.57795023 | down | 0.03016153 | 0.57950115 |

| | | | | | | |
|---|---|---|---|---|---|---|
| GEMIN6 | gem nuclear organelle associated protein 6 | -1.57812524 | 1.57812524 | down | 0.006348739 | 0.417034806 |
| ECM1 | extracellular matrix protein 1 | -1.578158057 | 1.578158057 | down | 0.032152242 | 0.58979223 |
| HNRNPH1 | heterogeneous nuclear ribonucleoprotein H1 (H) | -1.578234631 | 1.578234631 | down | 0.01709982 | 0.530571823 |
| CASP8 | caspase 8, apoptosis-related cysteine peptidase | -1.578478966 | 1.578478966 | down | 0.035887494 | 0.603048422 |
| HSPD1P5 | heat shock protein family D (Hsp60) member 1 pseudogene 5 | -1.578581087 | 1.578581087 | down | 0.011915878 | 0.495320532 |
| HSPD1 | heat shock protein family D (Hsp60) member 1 | -1.578581087 | 1.578581087 | down | 0.011915878 | 0.495320532 |
| OGDH | oxoglutarate (alpha-ketoglutarate) dehydrogenase (lipoamide) | -1.578584734 | 1.578584734 | down | 0.038740354 | 0.613615561 |
| SLC25A44 | solute carrier family 25, member 44 | -1.5790699 | 1.5790699 | down | 0.028777041 | 0.574350948 |
| SLC35E1 | solute carrier family 35 member E1 | -1.579277874 | 1.579277874 | down | 0.028761825 | 0.574350948 |
| PIF1 | PIF1 5'-to-3' DNA helicase | -1.579431136 | 1.579431136 | down | 0.044191699 | 0.623333169 |
| HGS | hepatocyte growth factor-regulated tyrosine kinase substrate | -1.579540617 | 1.579540617 | down | 0.047438148 | 0.627322764 |
| FAM136A | family with sequence similarity 136 member A | -1.579544267 | 1.579544267 | down | 0.008860946 | 0.466623412 |
| MOB3B | MOB kinase activator 3B | -1.579573463 | 1.579573463 | down | 0.027336155 | 0.56844604 |
| LTBR | lymphotoxin beta receptor | -1.579796104 | 1.579796104 | down | 0.018410934 | 0.537128348 |
| CUL1 | cullin 1 | -1.579865457 | 1.579865457 | down | 0.015617525 | 0.522063677 |
| FRG1CP | FSHD region gene 1 family member C, pseudogene | -1.579942115 | 1.579942115 | down | 0.045930024 | 0.627149649 |
| LOC102723780 | protein FRG1-like | -1.579942115 | 1.579942115 | down | 0.045930024 | 0.627149649 |
| FRG1EP | FSHD region gene 1 family member E, pseudogene | -1.579942115 | 1.579942115 | down | 0.045930024 | 0.627149649 |
| LOC102724813 | protein FRG1B | -1.579942115 | 1.579942115 | down | 0.045930024 | 0.627149649 |
| FRG1BP | FSHD region gene 1 family member B, pseudogene | -1.579942115 | 1.579942115 | down | 0.045930024 | 0.627149649 |
| FRG1DP | FSHD region gene 1 family member D, pseudogene | -1.579942115 | 1.579942115 | down | 0.045930024 | 0.627149649 |
| FRG1 | FSHD region gene 1 | -1.579942115 | 1.579942115 | down | 0.045930024 | 0.627149649 |
| LOC101930531 | uncharacterized LOC101930531 | -1.579942115 | 1.579942115 | down | 0.045930024 | 0.627149649 |
| MB21D1 | Mab-21 domain containing 1 | -1.579945765 | 1.579945765 | down | 0.034964185 | 0.599462172 |
| SCO2 | SCO2 cytochrome c oxidase assembly protein | -1.580058933 | 1.580058933 | down | 0.025594652 | 0.566329497 |
| PITRM1 | pitrilysin metallopeptidase 1 | -1.580789244 | 1.580789244 | down | 0.008886322 | 0.466623412 |
| KANSL1 | KAT8 regulatory NSL complex subunit 1 | -1.580800201 | 1.580800201 | down | 0.020640956 | 0.549146 |
| TRA2B | transformer 2 beta homolog (Drosophila) | -1.580840378 | 1.580840378 | down | 0.01084449 | 0.488995826 |
| HARS | histidyl-tRNA synthetase | -1.580946305 | 1.580946305 | down | 0.00772503 | 0.44458454 |
| EGFR | epidermal growth factor receptor | -1.581315277 | 1.581315277 | down | 0.006876782 | 0.430926948 |
| CD55 | CD55 molecule, decay accelerating factor for complement (Cromer [illegible] | -1.581366428 | 1.581366428 | down | 0.018544168 | 0.537153195 |
| PM20D2 | peptidase M20 domain containing 2 | -1.581636828 | 1.581636828 | down | 0.045935179 | 0.627149649 |
| SRSF9 | serine/arginine-rich splicing factor 9 | -1.581841485 | 1.581841485 | down | 0.002180552 | 0.288675084 |
| PIK3R3 | phosphoinositide-3-kinase regulatory subunit 3 | -1.582440991 | 1.582440991 | down | 0.027014144 | 0.568317266 |
| ASF1A | anti-silencing function 1A histone chaperone | -1.582495835 | 1.582495835 | down | 0.037699631 | 0.610579997 |
| WBP5 | WW domain binding protein 5 | -1.582678662 | 1.582678662 | down | 0.005123252 | 0.392367316 |
| CTH | cystathionine gamma-lyase | -1.582733515 | 1.582733515 | down | 0.012399988 | 0.503222212 |
| MPHOSPH8 | M-phase phosphoprotein 8 | -1.582821283 | 1.582821283 | down | 0.021017403 | 0.549146 |
| KPNA6 | karyopherin alpha 6 (importin alpha 7) | -1.582909055 | 1.582909055 | down | 0.0061654 | 0.414771056 |
| SMURF2 | SMAD specific E3 ubiquitin protein ligase 2 | -1.582930999 | 1.582930999 | down | 0.002679133 | 0.315760691 |
| SCRIB | scribbled planar cell polarity protein | -1.583026093 | 1.583026093 | down | 0.04986095 | 0.630405293 |
| TOP3B | topoisomerase (DNA) III beta | -1.583088273 | 1.583088273 | down | 0.013822903 | 0.511408446 |
| MUM1 | melanoma associated antigen (mutated) 1 | -1.583110219 | 1.583110219 | down | 0.025788318 | 0.566329497 |
| RIOK2 | RIO kinase 2 | -1.583384575 | 1.583384575 | down | 0.017939498 | 0.535584855 |
| TTC9C | tetratricopeptide repeat domain 9C | -1.58349799 | 1.58349799 | down | 0.036535729 | 0.604140802 |
| PHLDB2 | pleckstrin homology-like domain, family B, member 2 | -1.583633366 | 1.583633366 | down | 0.000827797 | 0.199221133 |
| BIRC3 | baculoviral IAP repeat containing 3 | -1.583644343 | 1.583644343 | down | 0.02090571 | 0.549146 |
| HIST1H2AC | histone cluster 1, H2ac | -1.58378705 | 1.58378705 | down | 0.017000332 | 0.530571823 |
| RASSF1 | Ras association (RalGDS/AF-6) domain family member 1 | -1.583830962 | 1.583830962 | down | 0.046766697 | 0.627185789 |
| RAET1L | retinoic acid early transcript 1L | -1.584138384 | 1.584138384 | down | 0.013564851 | 0.5101407 |
| RAET1G | retinoic acid early transcript 1G | -1.584138384 | 1.584138384 | down | 0.013564851 | 0.5101407 |
| ULBP2 | UL16 binding protein 2 | -1.584138384 | 1.584138384 | down | 0.013564851 | 0.5101407 |
| TCEB1P2 | transcription elongation factor B (SIII), polypeptide 1 pseudogene 2 | -1.584153025 | 1.584153025 | down | 0.009805586 | 0.477233577 |
| PDE12 | phosphodiesterase 12 | -1.584299438 | 1.584299438 | down | 0.043938908 | 0.623333169 |

| | | | | | | |
|---|---|---|---|---|---|---|
| ZNF639 | zinc finger protein 639 | -1.584321402 | 1.584321402 | down | 0.044081905 | 0.623333169 |
| MRPS5 | mitochondrial ribosomal protein S5 | -1.584423901 | 1.584423901 | down | 0.024973282 | 0.565551322 |
| YWHAE | tyrosine 3-monooxygenase/tryptophan 5-monooxygenase activation protein, epsilon | -1.584881566 | 1.584881566 | down | 0.001449106 | 0.250769966 |
| SLC16A1 | solute carrier family 16 (monocarboxylate transporter), member 1 | -1.584940157 | 1.584940157 | down | 0.029442359 | 0.576629742 |
| PHC1 | polyhomeotic homolog 1 (Drosophila) | -1.585020723 | 1.585020723 | down | 0.036787737 | 0.605213936 |
| FLII | flightless I actin binding protein | -1.585035372 | 1.585035372 | down | 0.000841338 | 0.19951366 |
| PPP2R3A | protein phosphatase 2 regulatory subunit B'', alpha | -1.585057345 | 1.585057345 | down | 0.017370157 | 0.531030242 |
| TREX1 | three prime repair exonuclease 1 | -1.585178204 | 1.585178204 | down | 0.010394909 | 0.485980503 |
| METTL3 | methyltransferase like 3 | -1.585200179 | 1.585200179 | down | 0.008789017 | 0.466623412 |
| TRMT6 | tRNA methyltransferase 6 | -1.585233143 | 1.585233143 | down | 0.024702951 | 0.563837901 |
| FAM63A | family with sequence similarity 63 member A | -1.585247794 | 1.585247794 | down | 0.03234803 | 0.590074234 |
| MROH6 | maestro heat-like repeat family member 6 | -1.585841262 | 1.585841262 | down | 0.047802761 | 0.627854743 |
| FERMT1 | fermitin family member 1 | -1.585855919 | 1.585855919 | down | 0.004723608 | 0.377936677 |
| CFL2 | cofilin 2 (muscle) | -1.586006154 | 1.586006154 | down | 0.001332013 | 0.241913463 |
| FAM83A | family with sequence similarity 83 member A | -1.586097768 | 1.586097768 | down | 0.042029922 | 0.623333169 |
| CENPL | centromere protein L | -1.586149074 | 1.586149074 | down | 0.049073609 | 0.62943979 |
| EZR | ezrin | -1.586178393 | 1.586178393 | down | 0.023159465 | 0.55642591 |
| ASPH | aspartate beta-hydroxylase | -1.586229701 | 1.586229701 | down | 0.024960156 | 0.565551322 |
| CHST3 | carbohydrate (chondroitin 6) sulfotransferase 3 | -1.586431288 | 1.586431288 | down | 0.028596343 | 0.574350948 |
| FLNC | filamin C, gamma | -1.586471608 | 1.586471608 | down | 0.001937707 | 0.274380687 |
| BCAR3 | breast cancer anti-estrogen resistance 3 | -1.586775876 | 1.586775876 | down | 0.029474124 | 0.576629742 |
| NUF2 | NUF2, NDC80 kinetochore complex component | -1.586808872 | 1.586808872 | down | 0.034544057 | 0.596676967 |
| FASTK | Fas-activated serine/threonine kinase | -1.586849202 | 1.586849202 | down | 0.022580262 | 0.551250506 |
| LRRFIP1 | leucine rich repeat (in FLII) interacting protein 1 | -1.586999532 | 1.586999532 | down | 0.043021185 | 0.623333169 |
| PGAM5 | PGAM family member 5, serine/threonine protein phosphatase, mitochondrial | -1.587058201 | 1.587058201 | down | 0.02360836 | 0.558835288 |
| KLF13 | Kruppel-like factor 13 | -1.587417596 | 1.587417596 | down | 0.030649264 | 0.582387558 |
| ALKBH2 | alkB homolog 2, alpha-ketoglutarate-dependent dioxygenase | -1.587490952 | 1.587490952 | down | 0.033382524 | 0.593014207 |
| ARHGAP5 | Rho GTPase activating protein 5 | -1.58783577 | 1.58783577 | down | 0.026783873 | 0.568317266 |
| MED16 | mediator complex subunit 16 | -1.587879795 | 1.587879795 | down | 0.019564379 | 0.546194159 |
| ERCC5 | excision repair cross-complementation group 5 | -1.587982524 | 1.587982524 | down | 0.010548001 | 0.486801343 |
| BIVM-ERCC5 | BIVM-ERCC5 readthrough | -1.587982524 | 1.587982524 | down | 0.010548001 | 0.486801343 |
| CWF19L1 | CWF19-like 1, cell cycle control (S. pombe) | -1.588081591 | 1.588081591 | down | 0.023482301 | 0.557191546 |
| IMMT | inner membrane protein, mitochondrial | -1.588151308 | 1.588151308 | down | 0.002960323 | 0.326391956 |
| ENO2 | enolase 2 (gamma, neuronal) | -1.588243046 | 1.588243046 | down | 0.028601399 | 0.574350948 |
| PEA15 | phosphoprotein enriched in astrocytes 15 | -1.58850361 | 1.58850361 | down | 0.003792694 | 0.351292726 |
| HSPA5 | heat shock protein family A (Hsp70) member 5 | -1.588621062 | 1.588621062 | down | 0.019577796 | 0.546194159 |
| PFDN6 | prefoldin subunit 6 | -1.588764218 | 1.588764218 | down | 0.012890821 | 0.50592937 |
| IMP3 | IMP3, U3 small nucleolar ribonucleoprotein | -1.588889031 | 1.588889031 | down | 0.003459223 | 0.339991958 |
| SHROOM3 | shroom family member 3 | -1.589138686 | 1.589138686 | down | 0.022397074 | 0.551250506 |
| C8orf58 | chromosome 8 open reading frame 58 | -1.589142358 | 1.589142358 | down | 0.028868455 | 0.574350948 |
| YLPM1 | YLP motif containing 1 | -1.589274544 | 1.589274544 | down | 0.014860882 | 0.516115951 |
| GOLGA8B | golgin A8 family member B | -1.589292904 | 1.589292904 | down | 0.029175524 | 0.575975177 |
| GOLGA8A | golgin A8 family member A | -1.589292904 | 1.589292904 | down | 0.029175524 | 0.575975177 |
| ARF6 | ADP ribosylation factor 6 | -1.589362675 | 1.589362675 | down | 0.004587045 | 0.374624543 |
| AJUBA | ajuba LIM protein | -1.589425104 | 1.589425104 | down | 0.010937775 | 0.489869256 |
| GOLPH3L | golgi phosphoprotein 3-like | -1.58974463 | 1.58974463 | down | 0.033654482 | 0.593014207 |
| CORO6 | coronin 6 | -1.589770342 | 1.589770342 | down | 0.007928245 | 0.447128041 |
| NLRP1 | NLR family, pyrin domain containing 1 | -1.589902581 | 1.589902581 | down | 0.027551046 | 0.569914309 |
| AXL | AXL receptor tyrosine kinase | -1.590137699 | 1.590137699 | down | 0.000412546 | 0.144894892 |
| C8orf33 | chromosome 8 open reading frame 33 | -1.590589665 | 1.590589665 | down | 0.006188018 | 0.414771056 |
| EXOSC10 | exosome component 10 | -1.590725648 | 1.590725648 | down | 0.01283031 | 0.505878264 |
| ZC3H3 | zinc finger CCCH-type containing 3 | -1.590740349 | 1.590740349 | down | 0.044658317 | 0.623333169 |
| TRIM8 | tripartite motif containing 8 | -1.590751376 | 1.590751376 | down | 0.008773113 | 0.466623412 |
| DNAJC6 | DnaJ heat shock protein family (Hsp40) member C6 | -1.590883696 | 1.590883696 | down | 0.048419617 | 0.628445433 |

| | | | | | | |
|---|---|---|---|---|---|---|
| SH2D3A | SH2 domain containing 3A | -1.591082197 | 1.591082197 | down | 0.009805503 | 0.477233577 |
| RMI1 | RecQ mediated genome instability 1 | -1.591129988 | 1.591129988 | down | 0.020677965 | 0.549146 |
| ZFP36L2 | ZFP36 ring finger protein-like 2 | -1.591196163 | 1.591196163 | down | 0.010543274 | 0.486801343 |
| ENOSF1 | enolase superfamily member 1 | -1.591328521 | 1.591328521 | down | 0.043524583 | 0.623333169 |
| TNFAIP3 | TNF alpha induced protein 3 | -1.591328521 | 1.591328521 | down | 0.027842173 | 0.572241118 |
| TNPO2 | transportin 2 | -1.59147192 | 1.59147192 | down | 0.005072954 | 0.39233172 |
| CREB1 | cAMP responsive element binding protein 1 | -1.591721981 | 1.591721981 | down | 0.031258846 | 0.585954505 |
| RETSAT | retinol saturase (all-trans-retinol 13,14-reductase) | -1.591817603 | 1.591817603 | down | 0.017060092 | 0.530571823 |
| RBM12 | RNA binding motif protein 12 | -1.592005186 | 1.592005186 | down | 0.017017554 | 0.530571823 |
| RNF26 | ring finger protein 26 | -1.592266368 | 1.592266368 | down | 0.043101065 | 0.623333169 |
| ZNF623 | zinc finger protein 623 | -1.592409852 | 1.592409852 | down | 0.031143632 | 0.58574728 |
| DNTTIP2 | deoxynucleotidyltransferase, terminal, interacting protein 2 | -1.592637982 | 1.592637982 | down | 0.036114371 | 0.603150244 |
| RBP7 | retinol binding protein 7 | -1.592645341 | 1.592645341 | down | 0.004748631 | 0.37840054 |
| RPS19BP1 | ribosomal protein S19 binding protein 1 | -1.592711579 | 1.592711579 | down | 0.003458679 | 0.339991958 |
| SPCS2P4 | signal peptidase complex subunit 2 homolog (S. cerevisiae) pseudogene 4 | -1.593028086 | 1.593028086 | down | 0.017207788 | 0.530571823 |
| SPCS2 | signal peptidase complex subunit 2 | -1.593028086 | 1.593028086 | down | 0.017207788 | 0.530571823 |
| EMC1 | ER membrane protein complex subunit 1 | -1.593061212 | 1.593061212 | down | 0.015211594 | 0.518081034 |
| SOAT1 | sterol O-acyltransferase 1 | -1.593079616 | 1.593079616 | down | 0.009591025 | 0.475470418 |
| DNAJB6 | DnaJ heat shock protein family (Hsp40) member B6 | -1.593204768 | 1.593204768 | down | 0.023301883 | 0.556498532 |
| CD59 | CD59 molecule, complement regulatory protein | -1.593458783 | 1.593458783 | down | 0.01853346 | 0.537153195 |
| DVL1 | dishevelled segment polarity protein 1 | -1.59383804 | 1.59383804 | down | 0.002086805 | 0.287025087 |
| CDC16 | cell division cycle 16 | -1.594217388 | 1.594217388 | down | 0.001728592 | 0.25677745 |
| CFB | complement factor B | -1.594504721 | 1.594504721 | down | 0.038306868 | 0.612498409 |
| PAGR1 | PAXIP1 associated glutamate-rich protein 1 | -1.594817899 | 1.594817899 | down | 0.039987858 | 0.620933229 |
| MRS2 | MRS2, magnesium transporter | -1.594869487 | 1.594869487 | down | 0.012719852 | 0.50529726 |
| TGFB1 | transforming growth factor beta 1 | -1.594902652 | 1.594902652 | down | 0.033539499 | 0.593014207 |
| ELP6 | elongator acetyltransferase complex subunit 6 | -1.594917392 | 1.594917392 | down | 0.013303132 | 0.509292857 |
| NARFL | nuclear prelamin A recognition factor-like | -1.595009521 | 1.595009521 | down | 0.029318808 | 0.576629742 |
| SRPRA | SRP receptor alpha subunit | -1.595090599 | 1.595090599 | down | 0.00221756 | 0.290980894 |
| NIN | ninein | -1.595101655 | 1.595101655 | down | 0.011856258 | 0.495320532 |
| AMZ1 | archaelysin family metallopeptidase 1 | -1.595186423 | 1.595186423 | down | 0.037235878 | 0.607707429 |
| MDFI | MyoD family inhibitor | -1.595363345 | 1.595363345 | down | 0.026844558 | 0.568317266 |
| PPP1R12A | protein phosphatase 1 regulatory subunit 12A | -1.595695126 | 1.595695126 | down | 0.016157193 | 0.524267534 |
| FEM1C | fem-1 homolog c (C. elegans) | -1.595934788 | 1.595934788 | down | 0.033475465 | 0.593014207 |
| KLHL2 | kelch like family member 2 | -1.595982725 | 1.595982725 | down | 0.029563552 | 0.576757894 |
| LOC100996724 | phosphodiesterase 4D interacting protein-like | -1.596023288 | 1.596023288 | down | 0.002777928 | 0.315798154 |
| PDE4DIP | phosphodiesterase 4D interacting protein | -1.596023288 | 1.596023288 | down | 0.002777928 | 0.315798154 |
| NFX1 | nuclear transcription factor, X-box binding 1 | -1.59613392 | 1.59613392 | down | 0.010578736 | 0.486801343 |
| DICER1 | dicer 1, ribonuclease type III | -1.596167111 | 1.596167111 | down | 0.027829129 | 0.572241118 |
| TTLL12 | tubulin tyrosine ligase like 12 | -1.596576523 | 1.596576523 | down | 0.003748198 | 0.350843151 |
| EAPP | E2F-associated phosphoprotein | -1.59687904 | 1.59687904 | down | 0.012651731 | 0.5051069 |
| UAP1 | UDP-N-acetylglucosamine pyrophosphorylase 1 | -1.597454717 | 1.597454717 | down | 0.000547811 | 0.165072126 |
| MICA | MHC class I polypeptide-related sequence A | -1.597499009 | 1.597499009 | down | 0.009030269 | 0.466623412 |
| COX18 | COX18 cytochrome c oxidase assembly factor | -1.597532228 | 1.597532228 | down | 0.036932789 | 0.606003692 |
| INTS10 | integrator complex subunit 10 | -1.597532228 | 1.597532228 | down | 0.021794698 | 0.550085548 |
| RHOF | ras homolog family member F (in filopodia) | -1.597831234 | 1.597831234 | down | 0.003017428 | 0.326391956 |
| ZNF786 | zinc finger protein 786 | -1.597975219 | 1.597975219 | down | 0.031109117 | 0.585377824 |
| ARSJ | arylsulfatase family member J | -1.598444186 | 1.598444186 | down | 0.041365622 | 0.623333169 |
| CASP4 | caspase 4 | -1.598466345 | 1.598466345 | down | 0.010746785 | 0.488995826 |
| KRT33A | keratin 33A, type I | -1.598503278 | 1.598503278 | down | 0.046800592 | 0.627274538 |
| SNORA7B | small nucleolar RNA, H/ACA box 7B | -1.598547598 | 1.598547598 | down | 0.016413331 | 0.524965293 |
| ZFC3H1 | zinc finger, C3H1-type containing | -1.598547598 | 1.598547598 | down | 0.02747902 | 0.568723258 |
| SNORA7A | small nucleolar RNA, H/ACA box 7A | -1.598547598 | 1.598547598 | down | 0.016413331 | 0.524965293 |
| RPL32 | ribosomal protein L32 | -1.598547598 | 1.598547598 | down | 0.016413331 | 0.524965293 |

| | | | | | | |
|---|---|---|---|---|---|---|
| RPL32P3 | ribosomal protein L32 pseudogene 3 | -1.598547598 | 1.598547598 | down | 0.016413331 | 0.524965293 |
| TSPAN5 | tetraspanin 5 | -1.598599307 | 1.598599307 | down | 0.040068831 | 0.621232589 |
| TSPAN4 | tetraspanin 4 | -1.598832018 | 1.598832018 | down | 0.019679553 | 0.547868191 |
| ZNF622 | zinc finger protein 622 | -1.599061067 | 1.599061067 | down | 0.001484298 | 0.250769966 |
| RAPH1 | Ras association (RalGDS/AF-6) and pleckstrin homology domains 1 | -1.599123877 | 1.599123877 | down | 0.018174816 | 0.536306821 |
| CDC42 | cell division cycle 42 | -1.599345578 | 1.599345578 | down | 0.001412313 | 0.248565272 |
| RPP14 | ribonuclease P/MRP 14kDa subunit | -1.599497092 | 1.599497092 | down | 0.008103489 | 0.454407182 |
| TIFA | TRAF-interacting protein with forkhead-associated domain | -1.600081107 | 1.600081107 | down | 0.009012218 | 0.466623412 |
| DNLZ | DNL-type zinc finger | -1.600125471 | 1.600125471 | down | 0.00633871 | 0.417034806 |
| BACE2 | beta-site APP-cleaving enzyme 2 | -1.600424962 | 1.600424962 | down | 0.046242256 | 0.627185789 |
| SRSF11 | serine/arginine-rich splicing factor 11 | -1.600546993 | 1.600546993 | down | 0.031775322 | 0.589351082 |
| WBP4 | WW domain binding protein 4 | -1.600561785 | 1.600561785 | down | 0.008790924 | 0.466623412 |
| EIF4A2 | eukaryotic translation initiation factor 4A2 | -1.600587672 | 1.600587672 | down | 0.000460424 | 0.152307542 |
| ATP5D | ATP synthase, H+ transporting, mitochondrial F1 complex, delta subunit | -1.600761494 | 1.600761494 | down | 0.002779658 | 0.315798154 |
| INTS3 | integrator complex subunit 3 | -1.601101796 | 1.601101796 | down | 0.025570925 | 0.566329497 |
| ERICH1 | glutamate rich 1 | -1.601449571 | 1.601449571 | down | 0.036606845 | 0.604560136 |
| ARG2 | arginase 2 | -1.601678996 | 1.601678996 | down | 0.033725674 | 0.593014207 |
| CCNC | cyclin C | -1.601845534 | 1.601845534 | down | 0.008096471 | 0.454407182 |
| XRCC6 | X-ray repair complementing defective repair in Chinese hamster cells 6 | -1.602052807 | 1.602052807 | down | 0.007385446 | 0.437407205 |
| RBM24 | RNA binding motif protein 24 | -1.602137944 | 1.602137944 | down | 0.049884732 | 0.630405293 |
| C9orf85 | chromosome 9 open reading frame 85 | -1.602467431 | 1.602467431 | down | 0.034732184 | 0.598002352 |
| PHLDA3 | pleckstrin homology-like domain, family A, member 3 | -1.602574807 | 1.602574807 | down | 0.005008718 | 0.38925276 |
| NFYC | nuclear transcription factor Y subunit gamma | -1.602663675 | 1.602663675 | down | 0.013525865 | 0.5101407 |
| SAT1 | spermidine/spermine N1-acetyltransferase 1 | -1.602830316 | 1.602830316 | down | 0.010290704 | 0.485753032 |
| ZMAT5 | zinc finger, matrin-type 5 | -1.603156242 | 1.603156242 | down | 0.011182084 | 0.489992559 |
| IDI1 | isopentenyl-diphosphate delta isomerase 1 | -1.603519282 | 1.603519282 | down | 0.014549875 | 0.515525808 |
| B4GAT1 | beta-1,4-glucuronyltransferase 1 | -1.603782353 | 1.603782353 | down | 0.029528234 | 0.576757894 |
| RAP1A | RAP1A, member of RAS oncogene family | -1.603841643 | 1.603841643 | down | 1.31714E-05 | 0.01517475 |
| PIP5K1A | phosphatidylinositol-4-phosphate 5-kinase, type I, alpha | -1.604345692 | 1.604345692 | down | 0.028060923 | 0.573529712 |
| CHKB | choline kinase beta | -1.604364226 | 1.604364226 | down | 0.033744489 | 0.593014207 |
| CHKB-CPT1B | CHKB-CPT1B readthrough (NMD candidate) | -1.604364226 | 1.604364226 | down | 0.033744489 | 0.593014207 |
| PELO | pelota homolog (Drosophila) | -1.604367933 | 1.604367933 | down | 0.007867356 | 0.446814625 |
| HIST1H2BJ | histone cluster 1, H2bj | -1.604608898 | 1.604608898 | down | 0.019890038 | 0.549146 |
| CHRAC1 | chromatin accessibility complex 1 | -1.604894396 | 1.604894396 | down | 0.01122757 | 0.489992559 |
| SNTB2 | syntrophin, beta 2 (dystrophin-associated protein A1, 59kDa, basic component 2) | -1.605498929 | 1.605498929 | down | 0.046366568 | 0.627185789 |
| PPP1R14C | protein phosphatase 1 regulatory inhibitor subunit 14C | -1.605506348 | 1.605506348 | down | 0.035697735 | 0.602074261 |
| SERHL | serine hydrolase-like (pseudogene) | -1.605536024 | 1.605536024 | down | 0.044589854 | 0.623333169 |
| SERHL2 | serine hydrolase-like 2 | -1.605536024 | 1.605536024 | down | 0.044589854 | 0.623333169 |
| MICB | MHC class I polypeptide-related sequence B | -1.60558054 | 1.60558054 | down | 0.009967923 | 0.478633621 |
| PIDD1 | p53-induced death domain protein 1 | -1.605643605 | 1.605643605 | down | 0.036080376 | 0.603150244 |
| SYTL4 | synaptotagmin like 4 | -1.605817977 | 1.605817977 | down | 0.02212474 | 0.550353494 |
| GLS | glutaminase | -1.60594784 | 1.60594784 | down | 0.016358181 | 0.524267534 |
| TMOD3 | tropomodulin 3 | -1.606036895 | 1.606036895 | down | 0.012035606 | 0.497669765 |
| TRIM25 | tripartite motif containing 25 | -1.606133377 | 1.606133377 | down | 0.00650104 | 0.423104193 |
| PSMD3 | proteasome 26S subunit, non-ATPase 3 | -1.606274399 | 1.606274399 | down | 9.39444E-05 | 0.060624968 |
| SNRNP35 | small nuclear ribonucleoprotein, U11/U12 35kDa subunit | -1.606467398 | 1.606467398 | down | 0.006254961 | 0.416626954 |
| BPNT1 | 3'(2'), 5'-bisphosphate nucleotidase 1 | -1.606474821 | 1.606474821 | down | 0.006502681 | 0.423104193 |
| BRF2 | BRF2, RNA polymerase III transcription initiation factor 50 kDa subunit | -1.606500804 | 1.606500804 | down | 0.004692629 | 0.377936677 |
| SUCO | SUN domain containing ossification factor | -1.606886878 | 1.606886878 | down | 0.048420551 | 0.628445433 |
| HIST1H4H | histone cluster 1, H4h | -1.606998262 | 1.606998262 | down | 0.041575205 | 0.623333169 |
| CAP2 | CAP, adenylate cyclase-associated protein, 2 (yeast) | -1.607109655 | 1.607109655 | down | 0.0084146 | 0.4606964 |
| SNIP1 | Smad nuclear interacting protein 1 | -1.607113368 | 1.607113368 | down | 0.046641835 | 0.627185789 |
| DMKN | dermokine | -1.607325036 | 1.607325036 | down | 0.016602242 | 0.526872128 |
| TLK1 | tousled like kinase 1 | -1.607481019 | 1.607481019 | down | 0.0318487 | 0.589351082 |

| | | | | | | |
|---|---|---|---|---|---|---|
| TCEB1 | transcription elongation factor B (SIII), polypeptide 1 (15kDa, elongin C) | -1.607804176 | 1.607804176 | down | 0.011099822 | 0.489992559 |
| C20orf24 | chromosome 20 open reading frame 24 | -1.607897049 | 1.607897049 | down | 0.000776098 | 0.195840366 |
| TADA2A | transcriptional adaptor 2A | -1.607937915 | 1.607937915 | down | 0.02675819 | 0.568317266 |
| STOX1 | storkhead box 1 | -1.608160837 | 1.608160837 | down | 0.024712284 | 0.563837901 |
| DCBLD2 | discoidin, CUB and LCCL domain containing 2 | -1.608669961 | 1.608669961 | down | 0.002359345 | 0.300161496 |
| C10orf76 | chromosome 10 open reading frame 76 | -1.608781469 | 1.608781469 | down | 0.007795981 | 0.446499763 |
| ZNF747 | zinc finger protein 747 | -1.608837226 | 1.608837226 | down | 0.027330973 | 0.56844604 |
| VEZT | vezatin, adherens junctions transmembrane protein | -1.608971051 | 1.608971051 | down | 0.017437865 | 0.531030242 |
| SLC35C1 | solute carrier family 35 (GDP-fucose transporter), member C1 | -1.609104887 | 1.609104887 | down | 0.040017975 | 0.620933229 |
| SRSF5 | serine/arginine-rich splicing factor 5 | -1.609149502 | 1.609149502 | down | 0.045969888 | 0.627185789 |
| CDK13 | cyclin-dependent kinase 13 | -1.609372593 | 1.609372593 | down | 0.021249136 | 0.549146 |
| MCM10 | minichromosome maintenance 10 replication initiation factor | -1.609755638 | 1.609755638 | down | 0.006894556 | 0.430926948 |
| HOXA7 | homeobox A7 | -1.610317354 | 1.610317354 | down | 0.037520775 | 0.609325627 |
| GPX8 | glutathione peroxidase 8 (putative) | -1.610384326 | 1.610384326 | down | 0.019375412 | 0.544017898 |
| C1orf116 | chromosome 1 open reading frame 116 | -1.610481069 | 1.610481069 | down | 0.024616703 | 0.563613185 |
| LOC100507412 | uncharacterized LOC100507412 | -1.610823438 | 1.610823438 | down | 0.004062845 | 0.35779398 |
| MYLIP | myosin regulatory light chain interacting protein | -1.611028149 | 1.611028149 | down | 0.00948767 | 0.474564332 |
| ST3GAL4 | ST3 beta-galactoside alpha-2,3-sialyltransferase 4 | -1.611519563 | 1.611519563 | down | 0.016157989 | 0.524267534 |
| MYPN | myopalladin | -1.611772775 | 1.611772775 | down | 0.029949813 | 0.579152964 |
| EWSR1 | EWS RNA binding protein 1 | -1.611936638 | 1.611936638 | down | 0.009741516 | 0.477233577 |
| TUG1 | taurine up-regulated 1 (non-protein coding) | -1.611936638 | 1.611936638 | down | 0.009741516 | 0.477233577 |
| TNFSF9 | tumor necrosis factor superfamily member 9 | -1.611977607 | 1.611977607 | down | 0.006342796 | 0.417034806 |
| STMN3 | stathmin 3 | -1.612141491 | 1.612141491 | down | 0.01929694 | 0.543126657 |
| MBD1 | methyl-CpG binding domain protein 1 | -1.612279316 | 1.612279316 | down | 0.037434462 | 0.609086153 |
| GPATCH3 | G-patch domain containing 3 | -1.61246931 | 1.61246931 | down | 0.045286887 | 0.624555255 |
| LYSMD4 | LysM, putative peptidoglycan-binding, domain containing 4 | -1.612525195 | 1.612525195 | down | 0.032789265 | 0.590424587 |
| CACNG1 | calcium channel, voltage-dependent, gamma subunit 1 | -1.612923897 | 1.612923897 | down | 0.021462981 | 0.549146 |
| STAG3L2 | stromal antigen 3-like 2 (pseudogene) | -1.613121421 | 1.613121421 | down | 0.002099917 | 0.287025087 |
| SLC4A1AP | solute carrier family 4 (anion exchanger), member 1, adaptor protein | -1.613166147 | 1.613166147 | down | 0.002266563 | 0.294466121 |
| RHPN2 | rhophilin, Rho GTPase binding protein 2 | -1.613192238 | 1.613192238 | down | 0.003806817 | 0.351759623 |
| NT5E | 5'-nucleotidase, ecto (CD73) | -1.613352518 | 1.613352518 | down | 0.043507942 | 0.623333169 |
| NDST1 | N-deacetylase/N-sulfotransferase (heparan glucosaminyl) 1 | -1.613568736 | 1.613568736 | down | 0.005480232 | 0.401886731 |
| RHOV | ras homolog family member V | -1.613617202 | 1.613617202 | down | 0.007335881 | 0.437407205 |
| SHQ1 | SHQ1, H/ACA ribonucleoprotein assembly factor | -1.613658213 | 1.613658213 | down | 0.044954728 | 0.623333169 |
| HYI | hydroxypyruvate isomerase (putative) | -1.613997528 | 1.613997528 | down | 0.000405554 | 0.144894892 |
| MARCH4 | membrane associated ring-CH-type finger 4 | -1.614381674 | 1.614381674 | down | 0.00690753 | 0.430926948 |
| CIPC | CLOCK-interacting pacemaker | -1.614750987 | 1.614750987 | down | 0.020861418 | 0.549146 |
| RABGGTB | Rab geranylgeranyltransferase, beta subunit | -1.615112922 | 1.615112922 | down | 0.028944066 | 0.574350948 |
| YY1 | YY1 transcription factor | -1.615213681 | 1.615213681 | down | 0.004540913 | 0.373292465 |
| ABCB8 | ATP binding cassette subfamily B member 8 | -1.615340572 | 1.615340572 | down | 0.042073158 | 0.623333169 |
| BID | BH3 interacting domain death agonist | -1.61543388 | 1.61543388 | down | 0.001175844 | 0.236158592 |
| AMIGO2 | adhesion molecule with Ig-like domain 2 | -1.615583185 | 1.615583185 | down | 0.019338065 | 0.543356841 |
| MRPS16 | mitochondrial ribosomal protein S16 | -1.615889303 | 1.615889303 | down | 0.026714554 | 0.568317266 |
| MT1A | metallothionein 1A | -1.616131998 | 1.616131998 | down | 0.044236088 | 0.623333169 |
| TBC1D1 | TBC1 domain family member 1 | -1.616251493 | 1.616251493 | down | 0.044972882 | 0.623365324 |
| DENND3 | DENN/MADD domain containing 3 | -1.61633365 | 1.61633365 | down | 0.042076315 | 0.623333169 |
| KDM5B | lysine (K)-specific demethylase 5B | -1.616363527 | 1.616363527 | down | 0.020273663 | 0.549146 |
| RBFOX2 | RNA binding protein, fox-1 homolog (C. elegans) 2 | -1.616561472 | 1.616561472 | down | 0.047381987 | 0.627322764 |
| ANKRD39 | ankyrin repeat domain 39 | -1.616587618 | 1.616587618 | down | 0.013347732 | 0.509292857 |
| SERPINB1 | serpin peptidase inhibitor, clade B (ovalbumin), member 1 | -1.616684733 | 1.616684733 | down | 0.001701501 | 0.255663523 |
| CPSF6 | cleavage and polyadenylation specific factor 6 | -1.616946228 | 1.616946228 | down | 0.00064183 | 0.184765442 |
| GRTP1 | growth hormone regulated TBC protein 1 | -1.617069519 | 1.617069519 | down | 0.044611892 | 0.623333169 |
| ANKS3 | ankyrin repeat and sterile alpha motif domain containing 3 | -1.617091936 | 1.617091936 | down | 0.030258233 | 0.579778515 |
| SKA1 | spindle and kinetochore associated complex subunit 1 | -1.617174136 | 1.617174136 | down | 0.046866683 | 0.627322764 |

| | | | | | | |
|---|---|---|---|---|---|---|
| SON | SON DNA binding protein | -1.617260077 | 1.617260077 | down | 0.046126122 | 0.627185789 |
| DPP7 | dipeptidyl-peptidase 7 | -1.61743571 | 1.61743571 | down | 0.008219494 | 0.45571887 |
| SMTN | smoothelin | -1.617577725 | 1.617577725 | down | 0.020678183 | 0.549146 |
| ADCY7 | adenylate cyclase 7 | -1.618011321 | 1.618011321 | down | 0.034630218 | 0.597640739 |
| CRLF3 | cytokine receptor-like factor 3 | -1.618183297 | 1.618183297 | down | 0.015087238 | 0.517793473 |
| SH2D4A | SH2 domain containing 4A | -1.618646974 | 1.618646974 | down | 0.000943146 | 0.215943958 |
| RAP1GDS1 | RAP1, GTP-GDP dissociation stimulator 1 | -1.618721773 | 1.618721773 | down | 0.007645211 | 0.443884559 |
| TMOD1 | tropomodulin 1 | -1.618789095 | 1.618789095 | down | 0.044140073 | 0.623333169 |
| HIST1H2BK | histone cluster 1, H2bk | -1.618807796 | 1.618807796 | down | 0.000520616 | 0.161370449 |
| CMTM5 | CKLF-like MARVEL transmembrane domain containing 5 | -1.618890084 | 1.618890084 | down | 0.040950897 | 0.623333169 |
| STK4 | serine/threonine kinase 4 | -1.618991079 | 1.618991079 | down | 0.000662477 | 0.18477288 |
| SNX33 | sorting nexin 33 | -1.619320291 | 1.619320291 | down | 0.003033983 | 0.326391956 |
| ARHGAP26 | Rho GTPase activating protein 26 | -1.619395121 | 1.619395121 | down | 0.049600261 | 0.630325871 |
| DGCR2 | DiGeorge syndrome critical region gene 2 | -1.620121153 | 1.620121153 | down | 0.032246312 | 0.589858765 |
| STK32C | serine/threonine kinase 32C | -1.620270891 | 1.620270891 | down | 0.040852157 | 0.623333169 |
| BIRC5 | baculoviral IAP repeat containing 5 | -1.620315815 | 1.620315815 | down | 0.037406743 | 0.608981156 |
| DOLPP1 | dolichyldiphosphatase 1 | -1.620517989 | 1.620517989 | down | 0.012758503 | 0.5057776 |
| CHFR | checkpoint with forkhead and ring finger domains, E3 ubiquitin protein ligase | -1.6209711 | 1.6209711 | down | 0.019876329 | 0.549146 |
| ZDHHC7 | zinc finger, DHHC-type containing 7 | -1.621274492 | 1.621274492 | down | 0.023318093 | 0.556498532 |
| GAS6 | growth arrest specific 6 | -1.621439322 | 1.621439322 | down | 0.001331656 | 0.241913463 |
| MBD2 | methyl-CpG binding domain protein 2 | -1.621532983 | 1.621532983 | down | 0.026644285 | 0.568317266 |
| SERPINE1 | serpin peptidase inhibitor, clade E (nexin, plasminogen activator inhibitor type 1), member 1 | -1.621918922 | 1.621918922 | down | 0.008329349 | 0.460228374 |
| EMILIN1 | elastin microfibril interfacer 1 | -1.622597348 | 1.622597348 | down | 0.046677077 | 0.627185789 |
| SETD2 | SET domain containing 2 | -1.622634839 | 1.622634839 | down | 0.006672566 | 0.42771383 |
| XAB2 | XPA binding protein 2 | -1.62285605 | 1.62285605 | down | 0.045018918 | 0.623564284 |
| MCM4 | minichromosome maintenance complex component 4 | -1.623309814 | 1.623309814 | down | 0.016720353 | 0.528635316 |
| ZNF808 | zinc finger protein 808 | -1.62343359 | 1.62343359 | down | 0.033698502 | 0.593014207 |
| ATF5 | activating transcription factor 5 | -1.623838741 | 1.623838741 | down | 0.015784862 | 0.522063677 |
| LPAR1 | lysophosphatidic acid receptor 1 | -1.6238575 | 1.6238575 | down | 0.029023081 | 0.574540338 |
| IL6ST | interleukin 6 signal transducer | -1.623928788 | 1.623928788 | down | 0.007044712 | 0.432628838 |
| NFIB | nuclear factor I/B | -1.624315297 | 1.624315297 | down | 0.033354403 | 0.593014207 |
| PA2G4 | proliferation-associated 2G4 | -1.624431643 | 1.624431643 | down | 0.001980509 | 0.276804943 |
| LAPTM5 | lysosomal protein transmembrane 5 | -1.624566765 | 1.624566765 | down | 0.024819966 | 0.56430245 |
| TRMU | tRNA 5-methylaminomethyl-2-thiouridylate methyltransferase | -1.62459304 | 1.62459304 | down | 0.005851696 | 0.409879029 |
| MKI67 | marker of proliferation Ki-67 | -1.624716914 | 1.624716914 | down | 0.011392231 | 0.493350006 |
| MAMSTR | MEF2 activating motif and SAP domain containing transcriptional regulator | -1.624893357 | 1.624893357 | down | 0.035952663 | 0.603119509 |
| LRP5L | LDL receptor related protein 5 like | -1.625306382 | 1.625306382 | down | 0.016196916 | 0.524267534 |
| SELPLG | selectin P ligand | -1.625546736 | 1.625546736 | down | 0.045946897 | 0.627149649 |
| EMP3 | epithelial membrane protein 3 | -1.625948658 | 1.625948658 | down | 0.010943916 | 0.489869256 |
| FAM73A | family with sequence similarity 73 member A | -1.625982469 | 1.625982469 | down | 0.035572711 | 0.601354231 |
| CHMP7 | charged multivesicular body protein 7 | -1.626189107 | 1.626189107 | down | 0.017567847 | 0.531150768 |
| TCOF1 | Treacher Collins-Franceschetti syndrome 1 | -1.626294314 | 1.626294314 | down | 0.003846672 | 0.352149401 |
| DZANK1 | double zinc ribbon and ankyrin repeat domains 1 | -1.626437107 | 1.626437107 | down | 0.031233979 | 0.585954505 |
| TBCEL | tubulin folding cofactor E-like | -1.626572396 | 1.626572396 | down | 0.028353494 | 0.57421459 |
| MAP7D3 | MAP7 domain containing 3 | -1.626764075 | 1.626764075 | down | 0.011482023 | 0.494546847 |
| KIF21A | kinesin family member 21A | -1.626891873 | 1.626891873 | down | 0.035450352 | 0.600698736 |
| MAP4K4 | mitogen-activated protein kinase kinase kinase kinase 4 | -1.627049755 | 1.627049755 | down | 0.002548305 | 0.309987919 |
| FANCM | Fanconi anemia complementation group M | -1.627155018 | 1.627155018 | down | 0.046858 | 0.627322764 |
| CHRNB1 | cholinergic receptor, nicotinic beta 1 | -1.627587421 | 1.627587421 | down | 0.009264891 | 0.470380292 |
| PSMD12 | proteasome 26S subunit, non-ATPase 12 | -1.627666394 | 1.627666394 | down | 0.000486085 | 0.154312327 |
| MGLL | monoglyceride lipase | -1.627809307 | 1.627809307 | down | 0.001555301 | 0.251952305 |
| EEA1 | early endosome antigen 1 | -1.62791462 | 1.62791462 | down | 0.034687686 | 0.597999402 |
| SPATA5L1 | spermatogenesis associated 5-like 1 | -1.628192978 | 1.628192978 | down | 0.036481772 | 0.603912936 |
| MATN2 | matrilin 2 | -1.628200502 | 1.628200502 | down | 0.008563481 | 0.466623412 |

| | | | | | | |
|---|---|---|---|---|---|---|
| CNOT4 | CCR4-NOT transcription complex subunit 4 | -1.628384848 | 1.628384848 | down | 0.030178354 | 0.57950115 |
| STEAP3 | STEAP3 metalloreductase | -1.628595554 | 1.628595554 | down | 0.020497434 | 0.549146 |
| ADGRF4 | adhesion G protein-coupled receptor F4 | -1.628810051 | 1.628810051 | down | 0.02080565 | 0.549146 |
| SIPA1L1 | signal-induced proliferation-associated 1 like 1 | -1.628817578 | 1.628817578 | down | 0.008645681 | 0.466623412 |
| TOR3A | torsin family 3, member A | -1.62889661 | 1.62889661 | down | 0.000484366 | 0.154312327 |
| DENND5B | DENN/MADD domain containing 5B | -1.629081035 | 1.629081035 | down | 0.01722672 | 0.530571823 |
| GPRC5C | G protein-coupled receptor, class C, group 5, member C | -1.629216544 | 1.629216544 | down | 0.030516181 | 0.581882081 |
| POLR3C | polymerase (RNA) III (DNA directed) polypeptide C (62kD) | -1.629344534 | 1.629344534 | down | 0.028592641 | 0.574350948 |
| TBRG1 | transforming growth factor beta regulator 1 | -1.629348299 | 1.629348299 | down | 0.021385539 | 0.549146 |
| TCHP | trichoplein, keratin filament binding | -1.629416063 | 1.629416063 | down | 0.035622958 | 0.601820319 |
| MTERF4 | mitochondrial transcription termination factor 4 | -1.62956666 | 1.62956666 | down | 0.023352298 | 0.556498532 |
| NUDT16L1 | nudix hydrolase 16 like 1 | -1.630033599 | 1.630033599 | down | 0.000771667 | 0.195840366 |
| SDCCAG8 | serologically defined colon cancer antigen 8 | -1.63018802 | 1.63018802 | down | 0.020011114 | 0.549146 |
| IL6R | interleukin 6 receptor | -1.630365057 | 1.630365057 | down | 0.019860502 | 0.549146 |
| ING5 | inhibitor of growth family member 5 | -1.630421562 | 1.630421562 | down | 0.02389581 | 0.56192268 |
| GFOD1 | glucose-fructose oxidoreductase domain containing 1 | -1.63053081 | 1.63053081 | down | 0.010773073 | 0.488995826 |
| VEGFC | vascular endothelial growth factor C | -1.630873673 | 1.630873673 | down | 0.032036409 | 0.589351082 |
| DDHD1 | DDHD domain containing 1 | -1.630956574 | 1.630956574 | down | 0.043426267 | 0.623333169 |
| ATG13 | autophagy related 13 | -1.631039479 | 1.631039479 | down | 0.045711257 | 0.625770788 |
| LOC102724229 | uncharacterized LOC102724229 | -1.631092239 | 1.631092239 | down | 0.048785385 | 0.62943979 |
| RASA4B | RAS p21 protein activator 4B | -1.631092239 | 1.631092239 | down | 0.048785385 | 0.62943979 |
| POLR2J4 | polymerase (RNA) II (DNA directed) polypeptide J4, pseudogene | -1.631092239 | 1.631092239 | down | 0.048785385 | 0.62943979 |
| RASA4 | RAS p21 protein activator 4 | -1.631092239 | 1.631092239 | down | 0.048785385 | 0.62943979 |
| ZNF532 | zinc finger protein 532 | -1.631190226 | 1.631190226 | down | 0.029637577 | 0.57728016 |
| SLC39A7 | solute carrier family 39 (zinc transporter), member 7 | -1.631536997 | 1.631536997 | down | 0.006907482 | 0.430926948 |
| NR3C1 | nuclear receptor subfamily 3 group C member 1 | -1.632019584 | 1.632019584 | down | 0.040368539 | 0.622535379 |
| TBX6 | T-box 6 | -1.632030896 | 1.632030896 | down | 0.021938234 | 0.550085548 |
| SSH2 | slingshot protein phosphatase 2 | -1.632219446 | 1.632219446 | down | 0.032068843 | 0.589351082 |
| GJA5 | gap junction protein alpha 5 | -1.632272244 | 1.632272244 | down | 0.009108079 | 0.466623412 |
| ELL2 | elongation factor, RNA polymerase II, 2 | -1.633177619 | 1.633177619 | down | 0.004038617 | 0.35779398 |
| FUBP1 | far upstream element (FUSE) binding protein 1 | -1.633181393 | 1.633181393 | down | 0.000767762 | 0.195840366 |
| MRTO4 | MRT4 homolog, ribosome maturation factor | -1.633309695 | 1.633309695 | down | 0.005209844 | 0.39485568 |
| NFRKB | nuclear factor related to kappaB binding protein | -1.633426685 | 1.633426685 | down | 0.009729158 | 0.477233577 |
| HMHA1 | histocompatibility (minor) HA-1 | -1.633521039 | 1.633521039 | down | 0.0440474 | 0.623333169 |
| ANP32D | acidic nuclear phosphoprotein 32 family member D | -1.633630495 | 1.633630495 | down | 0.044074475 | 0.623333169 |
| NCEH1 | neutral cholesterol ester hydrolase 1 | -1.63363427 | 1.63363427 | down | 0.005704439 | 0.404604007 |
| ACP7 | acid phosphatase 7, tartrate resistant (putative) | -1.633736184 | 1.633736184 | down | 0.019411804 | 0.544651223 |
| AP1S1 | adaptor-related protein complex 1 sigma 1 subunit | -1.633909831 | 1.633909831 | down | 0.007628388 | 0.443884559 |
| UBE3D | ubiquitin protein ligase E3D | -1.63431382 | 1.63431382 | down | 0.029958589 | 0.579152964 |
| ATAT1 | alpha tubulin acetyltransferase 1 | -1.634396896 | 1.634396896 | down | 0.017018818 | 0.530571823 |
| TP73 | tumor protein p73 | -1.634559283 | 1.634559283 | down | 0.030910253 | 0.583508935 |
| POLR3F | polymerase (RNA) III (DNA directed) polypeptide F, 39 kDa | -1.634955878 | 1.634955878 | down | 0.007470726 | 0.438278902 |
| TPCN2 | two pore segment channel 2 | -1.635201437 | 1.635201437 | down | 0.021107901 | 0.549146 |
| NSD1 | nuclear receptor binding SET domain protein 1 | -1.635379018 | 1.635379018 | down | 0.017676658 | 0.531989019 |
| MPC2 | mitochondrial pyruvate carrier 2 | -1.635836284 | 1.635836284 | down | 0.028210063 | 0.57421459 |
| PCMTD2 | protein-L-isoaspartate (D-aspartate) O-methyltransferase domain [illegible] | -1.635968575 | 1.635968575 | down | 0.014338624 | 0.514676952 |
| RBMS2 | RNA binding motif, single stranded interacting protein 2 | -1.636059295 | 1.636059295 | down | 0.032469275 | 0.590424587 |
| COL6A1 | collagen, type VI, alpha 1 | -1.636289897 | 1.636289897 | down | 0.041073286 | 0.623333169 |
| KRTAP2-3 | keratin associated protein 2-3 | -1.636395758 | 1.636395758 | down | 0.005535436 | 0.401886731 |
| CRCP | CGRP receptor component | -1.636471377 | 1.636471377 | down | 0.024635952 | 0.563613185 |
| CCDC150 | coiled-coil domain containing 150 | -1.636823053 | 1.636823053 | down | 0.007921015 | 0.447128041 |
| KIAA0907 | KIAA0907 | -1.636940295 | 1.636940295 | down | 0.002771385 | 0.315798154 |
| UCHL5 | ubiquitin C-terminal hydrolase L5 | -1.637091587 | 1.637091587 | down | 0.040859527 | 0.623333169 |
| FST | follistatin | -1.637148325 | 1.637148325 | down | 0.032231005 | 0.589852866 |

| | | | | | | |
|---|---|---|---|---|---|---|
| LOC100294341 | ADP-ribosylation factor-like protein 17 | -1.63751528 | 1.63751528 | down | 0.01301295 | 0.508400247 |
| LOC100996709 | ADP-ribosylation factor-like protein 17 | -1.63751528 | 1.63751528 | down | 0.01301295 | 0.508400247 |
| ARL17B | ADP ribosylation factor like GTPase 17B | -1.63751528 | 1.63751528 | down | 0.01301295 | 0.508400247 |
| ARL17A | ADP-ribosylation factor like GTPase 17A | -1.63751528 | 1.63751528 | down | 0.01301295 | 0.508400247 |
| SH3KBP1 | SH3-domain kinase binding protein 1 | -1.637651491 | 1.637651491 | down | 0.007148641 | 0.434730278 |
| FARP1 | FERM, ARH/RhoGEF and pleckstrin domain protein 1 | -1.637840691 | 1.637840691 | down | 0.031797789 | 0.589351082 |
| APOL1 | apolipoprotein L1 | -1.637992066 | 1.637992066 | down | 0.011439002 | 0.494287923 |
| APOL2 | apolipoprotein L, 2 | -1.637992066 | 1.637992066 | down | 0.011439002 | 0.494287923 |
| NUMBL | numb homolog (Drosophila)-like | -1.638116962 | 1.638116962 | down | 0.018492808 | 0.537153195 |
| LOC100506766 | uncharacterized LOC100506766 | -1.638397065 | 1.638397065 | down | 0.047895553 | 0.627854743 |
| BICD1 | bicaudal D homolog 1 (Drosophila) | -1.638662071 | 1.638662071 | down | 0.035189135 | 0.600433528 |
| ZNF230 | zinc finger protein 230 | -1.638764299 | 1.638764299 | down | 0.041615228 | 0.623333169 |
| PPP3R1 | protein phosphatase 3 regulatory subunit B, alpha | -1.638862747 | 1.638862747 | down | 0.025547847 | 0.566329497 |
| PCSK9 | proprotein convertase subtilisin/kexin type 9 | -1.638976349 | 1.638976349 | down | 0.046772787 | 0.627185789 |
| CYTH4 | cytohesin 4 | -1.639124042 | 1.639124042 | down | 0.030982502 | 0.584392041 |
| PCID2 | PCI domain containing 2 | -1.639302049 | 1.639302049 | down | 0.00242046 | 0.302480607 |
| IRF5 | interferon regulatory factor 5 | -1.639540685 | 1.639540685 | down | 0.047560568 | 0.627760335 |
| FRMD4A | FERM domain containing 4A | -1.639707372 | 1.639707372 | down | 0.031645417 | 0.589022431 |
| FNBP1 | formin binding protein 1 | -1.639828609 | 1.639828609 | down | 0.009312755 | 0.470801192 |
| AP4B1 | adaptor-related protein complex 4, beta 1 subunit | -1.639866498 | 1.639866498 | down | 0.004110958 | 0.358673662 |
| PNMA6A | paraneoplastic Ma antigen family member 6A | -1.640010482 | 1.640010482 | down | 0.027982963 | 0.572828574 |
| CCDC151 | coiled-coil domain containing 151 | -1.640215113 | 1.640215113 | down | 0.024206961 | 0.562851157 |
| ACAT2 | acetyl-CoA acetyltransferase 2 | -1.640870863 | 1.640870863 | down | 0.000291049 | 0.123163556 |
| CDT1 | chromatin licensing and DNA replication factor 1 | -1.640961854 | 1.640961854 | down | 0.001513501 | 0.251952305 |
| IGDCC4 | immunoglobulin superfamily, DCC subclass, member 4 | -1.642251446 | 1.642251446 | down | 0.047353884 | 0.627322764 |
| SLC6A17 | solute carrier family 6 (neutral amino acid transporter), member 17 | -1.64225524 | 1.64225524 | down | 0.038866447 | 0.613615561 |
| PTCD3 | pentatricopeptide repeat domain 3 | -1.642300774 | 1.642300774 | down | 0.001280169 | 0.240074176 |
| HDAC8 | histone deacetylase 8 | -1.642304568 | 1.642304568 | down | 0.032702054 | 0.590424587 |
| GJB3 | gap junction protein beta 3 | -1.642410819 | 1.642410819 | down | 0.007189366 | 0.434730278 |
| CISD3 | CDGSH iron sulfur domain 3 | -1.642695451 | 1.642695451 | down | 0.028417926 | 0.57421459 |
| DDX55 | DEAD (Asp-Glu-Ala-Asp) box polypeptide 55 | -1.642832093 | 1.642832093 | down | 0.013470012 | 0.509292857 |
| MET | MET proto-oncogene, receptor tyrosine kinase | -1.64306365 | 1.64306365 | down | 3.39392E-05 | 0.029689231 |
| OSCP1 | organic solute carrier partner 1 | -1.643416742 | 1.643416742 | down | 0.011570882 | 0.495320532 |
| ZNF200 | zinc finger protein 200 | -1.643439525 | 1.643439525 | down | 0.011482683 | 0.494546847 |
| HES2 | hes family bHLH transcription factor 2 | -1.643933229 | 1.643933229 | down | 0.032227308 | 0.589852866 |
| ACTR3C | ARP3 actin-related protein 3 homolog C (yeast) | -1.644248517 | 1.644248517 | down | 0.03619457 | 0.603150244 |
| KIAA1468 | KIAA1468 | -1.644563866 | 1.644563866 | down | 0.047175148 | 0.627322764 |
| CARNS1 | carnosine synthase 1 | -1.644715863 | 1.644715863 | down | 0.048991017 | 0.62943979 |
| BNC1 | basonuclin 1 | -1.644765265 | 1.644765265 | down | 0.017120007 | 0.530571823 |
| SETDB1 | SET domain, bifurcated 1 | -1.644791867 | 1.644791867 | down | 0.021343286 | 0.549146 |
| SUN2 | Sad1 and UNC84 domain containing 2 | -1.644837471 | 1.644837471 | down | 0.043122181 | 0.623333169 |
| MAP3K10 | mitogen-activated protein kinase kinase kinase 10 | -1.64524036 | 1.64524036 | down | 0.01889928 | 0.539906054 |
| NRL | neural retina leucine zipper | -1.645282175 | 1.645282175 | down | 0.024509392 | 0.563613185 |
| RFWD3 | ring finger and WD repeat domain 3 | -1.645396222 | 1.645396222 | down | 0.008124967 | 0.454504307 |
| SP110 | SP110 nuclear body protein | -1.645464653 | 1.645464653 | down | 0.025122517 | 0.565551322 |
| NPAS2 | neuronal PAS domain protein 2 | -1.645514078 | 1.645514078 | down | 0.042047074 | 0.623333169 |
| NUCKS1 | nuclear casein kinase and cyclin-dependent kinase substrate 1 | -1.64584488 | 1.64584488 | down | 0.04801602 | 0.627854743 |
| IGLJ3 | immunoglobulin lambda joining 3 | -1.646533314 | 1.646533314 | down | 0.024667389 | 0.563613185 |
| IGLL5 | immunoglobulin lambda-like polypeptide 5 | -1.646533314 | 1.646533314 | down | 0.024667389 | 0.563613185 |
| IGLV3-19 | immunoglobulin lambda variable 3-19 | -1.646533314 | 1.646533314 | down | 0.024667389 | 0.563613185 |
| INS-IGF2 | INS-IGF2 readthrough | -1.646811052 | 1.646811052 | down | 0.026614619 | 0.568317266 |
| INS | insulin | -1.646811052 | 1.646811052 | down | 0.026614619 | 0.568317266 |
| ERP44 | endoplasmic reticulum protein 44 | -1.646940425 | 1.646940425 | down | 0.025959932 | 0.567099175 |
| ATP6V0E2 | ATPase, H+ transporting V0 subunit e2 | -1.647073614 | 1.647073614 | down | 0.048674996 | 0.62943979 |

| | | | | | | |
|---|---|---|---|---|---|---|
| CLK4 | CDC like kinase 4 | -1.647195396 | 1.647195396 | down | 0.040658104 | 0.623259064 |
| PRRT2 | proline-rich transmembrane protein 2 | -1.647222037 | 1.647222037 | down | 0.037205168 | 0.607707429 |
| ATP9B | ATPase, class II, type 9B | -1.647362861 | 1.647362861 | down | 0.042626244 | 0.623333169 |
| S100A3 | S100 calcium binding protein A3 | -1.64787678 | 1.64787678 | down | 0.025303769 | 0.565656951 |
| VWA5A | von Willebrand factor A domain containing 5A | -1.648230907 | 1.648230907 | down | 0.006863172 | 0.430926948 |
| HN1L | hematological and neurological expressed 1-like | -1.648554637 | 1.648554637 | down | 0.000819569 | 0.199221133 |
| MKKS | McKusick-Kaufman syndrome | -1.648760335 | 1.648760335 | down | 0.027172852 | 0.568317266 |
| SOX9 | SRY-box 9 | -1.649019397 | 1.649019397 | down | 0.010515995 | 0.486801343 |
| KCNS1 | potassium voltage-gated channel, modifier subfamily S, member 1 | -1.64927469 | 1.64927469 | down | 0.006332997 | 0.417034806 |
| SOCS3 | suppressor of cytokine signaling 3 | -1.649747276 | 1.649747276 | down | 0.001443136 | 0.250769966 |
| ANKRD13C | ankyrin repeat domain 13C | -1.649888316 | 1.649888316 | down | 0.009893011 | 0.477252924 |
| SLC37A3 | solute carrier family 37, member 3 | -1.649953122 | 1.649953122 | down | 0.035456132 | 0.600698736 |
| RUNX3 | runt-related transcription factor 3 | -1.649991245 | 1.649991245 | down | 0.020446482 | 0.549146 |
| PPM1L | protein phosphatase, Mg2+/Mn2+ dependent 1L | -1.650101805 | 1.650101805 | down | 0.031599744 | 0.588700386 |
| LOC101928168 | uncharacterized LOC101928168 | -1.650143743 | 1.650143743 | down | 0.032461003 | 0.590424587 |
| LINC00656 | long intergenic non-protein coding RNA 656 | -1.650178057 | 1.650178057 | down | 0.035636813 | 0.601820319 |
| SEC14L1 | SEC14-like lipid binding 1 | -1.650544119 | 1.650544119 | down | 0.02327997 | 0.556498532 |
| LMNB1 | lamin B1 | -1.650704297 | 1.650704297 | down | 0.040262964 | 0.62179348 |
| ASB9 | ankyrin repeat and SOCS box containing 9 | -1.650830161 | 1.650830161 | down | 0.028292968 | 0.57421459 |
| GATS | GATS, stromal antigen 3 opposite strand | -1.650849233 | 1.650849233 | down | 0.047278328 | 0.627322764 |
| IL10 | interleukin 10 | -1.650879747 | 1.650879747 | down | 0.036973406 | 0.606003692 |
| LOC100506548 | uncharacterized LOC100506548 | -1.65095985 | 1.65095985 | down | 0.038737928 | 0.613615561 |
| RPL37 | ribosomal protein L37 | -1.65095985 | 1.65095985 | down | 0.038737928 | 0.613615561 |
| FHOD3 | formin homology 2 domain containing 3 | -1.65132227 | 1.65132227 | down | 0.013480813 | 0.509292857 |
| CSAG1 | chondrosarcoma associated gene 1 | -1.651429104 | 1.651429104 | down | 0.03694097 | 0.606003692 |
| PHF10 | PHD finger protein 10 | -1.65149397 | 1.65149397 | down | 0.001492246 | 0.251035295 |
| ARNT2 | aryl hydrocarbon receptor nuclear translocator 2 | -1.651501602 | 1.651501602 | down | 0.040196892 | 0.621304652 |
| IGHG3 | immunoglobulin heavy constant gamma 3 (G3m marker) | -1.652253482 | 1.652253482 | down | 0.046925531 | 0.627322764 |
| HMGA2 | high mobility group AT-hook 2 | -1.652322198 | 1.652322198 | down | 0.000197602 | 0.096064854 |
| ZNF24 | zinc finger protein 24 | -1.652452004 | 1.652452004 | down | 0.022479434 | 0.551250506 |
| YOD1 | YOD1 deubiquitinase | -1.653322732 | 1.653322732 | down | 0.030749751 | 0.582533534 |
| TMEM135 | transmembrane protein 135 | -1.653326552 | 1.653326552 | down | 0.035739878 | 0.602268962 |
| ANKRD1 | ankyrin repeat domain 1 (cardiac muscle) | -1.653360932 | 1.653360932 | down | 0.027158877 | 0.568317266 |
| LOC100128108 | putative ubiquitin-conjugating enzyme E2Q2-like protein | -1.653991365 | 1.653991365 | down | 0.021880922 | 0.550085548 |
| FOPNL | FGFR1OP N-terminal like | -1.65430476 | 1.65430476 | down | 0.002323943 | 0.298884417 |
| MSGN1 | mesogenin 1 | -1.654931729 | 1.654931729 | down | 0.020551601 | 0.549146 |
| NALCN | sodium leak channel, non selective | -1.655168815 | 1.655168815 | down | 0.014052199 | 0.513372826 |
| ASAP1-IT2 | ASAP1 intronic transcript 2 | -1.655310319 | 1.655310319 | down | 0.041918039 | 0.623333169 |
| SLC16A2 | solute carrier family 16, member 2 (thyroid hormone transporter) | -1.655604838 | 1.655604838 | down | 0.004142483 | 0.358673662 |
| KIF1A | kinesin family member 1A | -1.655631615 | 1.655631615 | down | 0.033776057 | 0.593304103 |
| PRKCDBP | protein kinase C, delta binding protein | -1.655754029 | 1.655754029 | down | 0.005257347 | 0.395708317 |
| LINC00893 | long intergenic non-protein coding RNA 893 | -1.655864976 | 1.655864976 | down | 0.012737463 | 0.505453867 |
| LINC00894 | long intergenic non-protein coding RNA 894 | -1.655864976 | 1.655864976 | down | 0.012737463 | 0.505453867 |
| GLB1L3 | galactosidase beta 1 like 3 | -1.656037148 | 1.656037148 | down | 0.045376045 | 0.624555255 |
| ZNF276 | zinc finger protein 276 | -1.65615194 | 1.65615194 | down | 0.014266835 | 0.514676952 |
| IHH | indian hedgehog | -1.656385374 | 1.656385374 | down | 0.027859973 | 0.572241118 |
| GATSL2 | GATS protein-like 2 | -1.656588221 | 1.656588221 | down | 0.029312115 | 0.576629742 |
| C1orf27 | chromosome 1 open reading frame 27 | -1.656615014 | 1.656615014 | down | 0.035578661 | 0.601354231 |
| PDE7A | phosphodiesterase 7A | -1.656733674 | 1.656733674 | down | 0.043662259 | 0.623333169 |
| BTBD11 | BTB (POZ) domain containing 11 | -1.657005475 | 1.657005475 | down | 0.015765743 | 0.522063677 |
| XYLT2 | xylosyltransferase II | -1.657239029 | 1.657239029 | down | 0.021134016 | 0.549146 |
| LAMC1 | laminin subunit gamma 1 | -1.65744581 | 1.65744581 | down | 0.016137019 | 0.524267534 |
| FCF1 | FCF1 rRNA-processing protein | -1.65755687 | 1.65755687 | down | 0.00303706 | 0.326391956 |
| ZHX3 | zinc fingers and homeoboxes 3 | -1.658154421 | 1.658154421 | down | 0.007877286 | 0.446814625 |

| | | | | | | |
|---|---|---|---|---|---|---|
| THBS1 | thrombospondin 1 | -1.658208058 | 1.658208058 | down | 0.001619693 | 0.255663523 |
| CRTAP | cartilage associated protein | -1.6588825 | 1.6588825 | down | 0.02391501 | 0.562024581 |
| GPR35 | G protein-coupled receptor 35 | -1.6600059 | 1.6600059 | down | 0.013480362 | 0.509292857 |
| PDDC1 | Parkinson disease 7 domain containing 1 | -1.660577477 | 1.660577477 | down | 0.019952846 | 0.549146 |
| TEC | transient erythroblastopenia of childhood | -1.660777 | 1.660777 | down | 0.028868726 | 0.574350948 |
| TEC | tec protein tyrosine kinase | -1.660777 | 1.660777 | down | 0.028868726 | 0.574350948 |
| KCNE5 | potassium channel, voltage gated subfamily E regulatory beta subunit 5 | -1.660949683 | 1.660949683 | down | 0.030262275 | 0.579778515 |
| TBC1D4 | TBC1 domain family member 4 | -1.661352681 | 1.661352681 | down | 0.001536653 | 0.251952305 |
| BAK1 | BCL2-antagonist/killer 1 | -1.661517747 | 1.661517747 | down | 0.041425204 | 0.623333169 |
| PIWIL2 | piwi-like RNA-mediated gene silencing 2 | -1.661928562 | 1.661928562 | down | 0.044730578 | 0.623333169 |
| IL16 | interleukin 16 | -1.662243461 | 1.662243461 | down | 0.047388147 | 0.627322764 |
| PSMD13 | proteasome 26S subunit, non-ATPase 13 | -1.662266505 | 1.662266505 | down | 0.000417697 | 0.144894892 |
| E2F8 | E2F transcription factor 8 | -1.662473913 | 1.662473913 | down | 0.013560207 | 0.5101407 |
| RPUSD4 | RNA pseudouridylate synthase domain containing 4 | -1.662485436 | 1.662485436 | down | 0.002590818 | 0.311888575 |
| KRTAP12-1 | keratin associated protein 12-1 | -1.662627565 | 1.662627565 | down | 0.039041504 | 0.613731439 |
| C10orf25 | chromosome 10 open reading frame 25 | -1.662658297 | 1.662658297 | down | 0.020292545 | 0.549146 |
| USP17L19 | ubiquitin specific peptidase 17-like family member 19 | -1.662738972 | 1.662738972 | down | 0.045341932 | 0.624555255 |
| USP17L21 | ubiquitin specific peptidase 17-like family member 21 | -1.662738972 | 1.662738972 | down | 0.045341932 | 0.624555255 |
| USP17L1 | ubiquitin specific peptidase 17-like family member 1 | -1.662738972 | 1.662738972 | down | 0.045341932 | 0.624555255 |
| USP17L2 | ubiquitin specific peptidase 17-like family member 2 | -1.662738972 | 1.662738972 | down | 0.045341932 | 0.624555255 |
| USP17L26 | ubiquitin specific peptidase 17-like family member 26 | -1.662738972 | 1.662738972 | down | 0.045341932 | 0.624555255 |
| USP17L30 | ubiquitin specific peptidase 17-like family member 30 | -1.662738972 | 1.662738972 | down | 0.045341932 | 0.624555255 |
| USP17L6P | ubiquitin specific peptidase 17-like family member 6, pseudogene | -1.662738972 | 1.662738972 | down | 0.045341932 | 0.624555255 |
| USP17L29 | ubiquitin specific peptidase 17-like family member 29 | -1.662738972 | 1.662738972 | down | 0.045341932 | 0.624555255 |
| USP17L10 | ubiquitin specific peptidase 17-like family member 10 | -1.662738972 | 1.662738972 | down | 0.045341932 | 0.624555255 |
| USP17L27 | ubiquitin specific peptidase 17-like family member 27 | -1.662738972 | 1.662738972 | down | 0.045341932 | 0.624555255 |
| USP17L20 | ubiquitin specific peptidase 17-like family member 20 | -1.662738972 | 1.662738972 | down | 0.045341932 | 0.624555255 |
| USP17L28 | ubiquitin specific peptidase 17-like family member 28 | -1.662738972 | 1.662738972 | down | 0.045341932 | 0.624555255 |
| USP17L5 | ubiquitin specific peptidase 17-like family member 5 | -1.662738972 | 1.662738972 | down | 0.045341932 | 0.624555255 |
| USP17L4 | ubiquitin specific peptidase 17-like family member 4 | -1.662738972 | 1.662738972 | down | 0.045341932 | 0.624555255 |
| USP17L14P | ubiquitin specific peptidase 17-like family member 14, pseudogene | -1.662738972 | 1.662738972 | down | 0.045341932 | 0.624555255 |
| USP17L12 | ubiquitin specific peptidase 17-like family member 12 | -1.662738972 | 1.662738972 | down | 0.045341932 | 0.624555255 |
| USP17L18 | ubiquitin specific peptidase 17-like family member 18 | -1.662738972 | 1.662738972 | down | 0.045341932 | 0.624555255 |
| USP17L9P | ubiquitin specific peptidase 17-like family member 9, pseudogene | -1.662738972 | 1.662738972 | down | 0.045341932 | 0.624555255 |
| USP17L15 | ubiquitin specific peptidase 17-like family member 15 | -1.662738972 | 1.662738972 | down | 0.045341932 | 0.624555255 |
| USP17L22 | ubiquitin specific peptidase 17-like family member 22 | -1.662738972 | 1.662738972 | down | 0.045341932 | 0.624555255 |
| USP17L8 | ubiquitin specific peptidase 17-like family member 8 | -1.662738972 | 1.662738972 | down | 0.045341932 | 0.624555255 |
| USP17L24 | ubiquitin specific peptidase 17-like family member 24 | -1.662738972 | 1.662738972 | down | 0.045341932 | 0.624555255 |
| USP17L17 | ubiquitin specific peptidase 17-like family member 17 | -1.662738972 | 1.662738972 | down | 0.045341932 | 0.624555255 |
| USP17L13 | ubiquitin specific peptidase 17-like family member 13 | -1.662738972 | 1.662738972 | down | 0.045341932 | 0.624555255 |
| USP17L3 | ubiquitin specific peptidase 17-like family member 3 | -1.662738972 | 1.662738972 | down | 0.045341932 | 0.624555255 |
| USP17L11 | ubiquitin specific peptidase 17-like family member 11 | -1.662738972 | 1.662738972 | down | 0.045341932 | 0.624555255 |
| USP17L25 | ubiquitin specific peptidase 17-like family member 25 | -1.662738972 | 1.662738972 | down | 0.045341932 | 0.624555255 |
| SPEM1 | spermatid maturation 1 | -1.662908017 | 1.662908017 | down | 0.020506075 | 0.549146 |
| EIF2AK1 | eukaryotic translation initiation factor 2 alpha kinase 1 | -1.663023285 | 1.663023285 | down | 0.018393851 | 0.537128348 |
| CDRT1 | CMT1A duplicated region transcript 1 | -1.664041833 | 1.664041833 | down | 0.034072705 | 0.595139711 |
| WASL | Wiskott-Aldrich syndrome-like | -1.66417256 | 1.66417256 | down | 0.02097506 | 0.549146 |
| CHN2 | chimerin 2 | -1.664572494 | 1.664572494 | down | 0.043037724 | 0.623333169 |
| RASA2 | RAS p21 protein activator 2 | -1.664984064 | 1.664984064 | down | 0.020859916 | 0.549146 |
| SYNCRIP | synaptotagmin binding, cytoplasmic RNA interacting protein | -1.665007146 | 1.665007146 | down | 0.022238031 | 0.550353494 |
| ALDOAP2 | aldolase, fructose-bisphosphate A pseudogene 2 | -1.665084087 | 1.665084087 | down | 0.047570407 | 0.627760335 |
| GPALPP1 | GPALPP motifs containing 1 | -1.665199506 | 1.665199506 | down | 0.034026105 | 0.595139711 |
| CSF1 | colony stimulating factor 1 (macrophage) | -1.665888338 | 1.665888338 | down | 0.030736657 | 0.582533534 |
| RBM48 | RNA binding motif protein 48 | -1.666569754 | 1.666569754 | down | 0.024635374 | 0.563613185 |

| | | | | | | |
|---|---|---|---|---|---|---|
| ABHD5 | abhydrolase domain containing 5 | -1.666635215 | 1.666635215 | down | 0.001092796 | 0.225224604 |
| GLYCTK | glycerate kinase | -1.666758444 | 1.666758444 | down | 0.03511315 | 0.600433528 |
| UBAP2 | ubiquitin associated protein 2 | -1.667039593 | 1.667039593 | down | 0.017424135 | 0.531030242 |
| MOK | MOK protein kinase | -1.667567356 | 1.667567356 | down | 0.000784046 | 0.196585701 |
| ZBED2 | zinc finger, BED-type containing 2 | -1.668149245 | 1.668149245 | down | 0.000739497 | 0.195840366 |
| EEF1D | eukaryotic translation elongation factor 1 delta (guanine nucleotide exchange protein) | -1.668823874 | 1.668823874 | down | 0.005502662 | 0.401886731 |
| HIST1H2AG | histone cluster 1, H2ag | -1.669637647 | 1.669637647 | down | 0.044082728 | 0.623333169 |
| NDUFS7 | NADH:ubiquinone oxidoreductase core subunit S7 | -1.66971866 | 1.66971866 | down | 0.02248349 | 0.551250506 |
| C16orf74 | chromosome 16 open reading frame 74 | -1.66987684 | 1.66987684 | down | 0.005608038 | 0.402842701 |
| XRCC2 | X-ray repair complementing defective repair in Chinese hamster cells 2 | -1.670054329 | 1.670054329 | down | 0.019135586 | 0.541887884 |
| AMOTL1 | angiomotin like 1 | -1.670154656 | 1.670154656 | down | 0.000735555 | 0.195840366 |
| FZD7 | frizzled class receptor 7 | -1.670818515 | 1.670818515 | down | 0.020752349 | 0.549146 |
| RGS2 | regulator of G-protein signaling 2 | -1.670942053 | 1.670942053 | down | 0.000774797 | 0.195840366 |
| RNF43 | ring finger protein 43 | -1.671015408 | 1.671015408 | down | 0.047354974 | 0.627322764 |
| CREB3L2 | cAMP responsive element binding protein 3-like 2 | -1.671505809 | 1.671505809 | down | 0.031380577 | 0.58634196 |
| PRKAB2 | protein kinase, AMP-activated, beta 2 non-catalytic subunit | -1.671536706 | 1.671536706 | down | 0.014590789 | 0.515525808 |
| LILRB3 | leukocyte immunoglobulin-like receptor, subfamily B (with TM and ITIM domains), member 3 | -1.671791622 | 1.671791622 | down | 0.027060803 | 0.568317266 |
| LOC102725015 | leukocyte immunoglobulin-like receptor subfamily B member 3 | -1.671791622 | 1.671791622 | down | 0.027060803 | 0.568317266 |
| LOC102725031 | leukocyte immunoglobulin-like receptor subfamily B member 3 | -1.671791622 | 1.671791622 | down | 0.027060803 | 0.568317266 |
| LILRB2 | leukocyte immunoglobulin-like receptor, subfamily B (with TM and ITIM domains), member 2 | -1.671791622 | 1.671791622 | down | 0.027060803 | 0.568317266 |
| LOC102725034 | leukocyte immunoglobulin-like receptor subfamily B member 3 | -1.671791622 | 1.671791622 | down | 0.027060803 | 0.568317266 |
| LOC102725029 | leukocyte immunoglobulin-like receptor subfamily B member 3 | -1.671791622 | 1.671791622 | down | 0.027060803 | 0.568317266 |
| LOC102725035 | leukocyte immunoglobulin-like receptor subfamily B member 3 | -1.671791622 | 1.671791622 | down | 0.027060803 | 0.568317266 |
| LILRA6 | leukocyte immunoglobulin-like receptor, subfamily A (with TM domain), member 6 | -1.671791622 | 1.671791622 | down | 0.027060803 | 0.568317266 |
| C14orf2 | chromosome 14 open reading frame 2 | -1.67205044 | 1.67205044 | down | 0.003719613 | 0.349787905 |
| ROBO4 | roundabout guidance receptor 4 | -1.672583654 | 1.672583654 | down | 0.036600487 | 0.604560136 |
| FAM163B | family with sequence similarity 163 member B | -1.672703458 | 1.672703458 | down | 0.043354957 | 0.623333169 |
| PPP1R1B | protein phosphatase 1 regulatory inhibitor subunit 1B | -1.673140233 | 1.673140233 | down | 0.040603324 | 0.623259064 |
| PROS1 | protein S (alpha) | -1.673364463 | 1.673364463 | down | 0.048118094 | 0.627892392 |
| ICAM3 | intercellular adhesion molecule 3 | -1.67346499 | 1.67346499 | down | 0.038160713 | 0.612253826 |
| KIAA1324 | KIAA1324 | -1.673685396 | 1.673685396 | down | 0.039022831 | 0.613717043 |
| IL17RD | interleukin 17 receptor D | -1.674586658 | 1.674586658 | down | 0.027040976 | 0.568317266 |
| ARHGAP32 | Rho GTPase activating protein 32 | -1.674729821 | 1.674729821 | down | 0.002359425 | 0.300161496 |
| BBS4 | Bardet-Biedl syndrome 4 | -1.675836849 | 1.675836849 | down | 0.010523697 | 0.486801343 |
| ITPR2 | inositol 1,4,5-trisphosphate receptor, type 2 | -1.67680513 | 1.67680513 | down | 0.024785773 | 0.56430245 |
| MLH1 | mutL homolog 1 | -1.677456129 | 1.677456129 | down | 0.041231525 | 0.623333169 |
| LMO1 | LIM domain only 1 | -1.677479384 | 1.677479384 | down | 0.031364324 | 0.58634196 |
| OR52M1 | olfactory receptor, family 52, subfamily M, member 1 | -1.678262478 | 1.678262478 | down | 0.030361032 | 0.580739562 |
| HERC2P9 | hect domain and RLD 2 pseudogene 9 | -1.678277989 | 1.678277989 | down | 0.015944998 | 0.523501532 |
| HERC2P2 | hect domain and RLD 2 pseudogene 2 | -1.678277989 | 1.678277989 | down | 0.015944998 | 0.523501532 |
| HERC2 | HECT and RLD domain containing E3 ubiquitin protein ligase 2 | -1.678277989 | 1.678277989 | down | 0.015944998 | 0.523501532 |
| LOC105369242 | E3 ubiquitin-protein ligase HERC2-like | -1.678277989 | 1.678277989 | down | 0.015944998 | 0.523501532 |
| MAP4 | microtubule associated protein 4 | -1.679185603 | 1.679185603 | down | 0.013723206 | 0.511408446 |
| TGM2 | transglutaminase 2 | -1.67924768 | 1.67924768 | down | 8.86038E-05 | 0.059972367 |
| MITF | microphthalmia-associated transcription factor | -1.67970945 | 1.67970945 | down | 0.006864537 | 0.430926948 |
| SSR3 | signal sequence receptor, gamma (translocon-associated protein gamma) | -1.680295576 | 1.680295576 | down | 0.002167038 | 0.288675084 |
| E2F6 | E2F transcription factor 6 | -1.680524648 | 1.680524648 | down | 0.048849467 | 0.62943979 |
| OSBPL5 | oxysterol binding protein like 5 | -1.680726567 | 1.680726567 | down | 0.040204251 | 0.621304652 |
| NAV3 | neuron navigator 3 | -1.680738217 | 1.680738217 | down | 0.018899798 | 0.539906054 |
| C22orf39 | chromosome 22 open reading frame 39 | -1.681215935 | 1.681215935 | down | 0.002700187 | 0.315798154 |
| RCN3 | reticulocalbin 3 | -1.68129751 | 1.68129751 | down | 0.03303675 | 0.592229281 |
| LINC00909 | long intergenic non-protein coding RNA 909 | -1.681445132 | 1.681445132 | down | 0.0149271 | 0.516117046 |
| ZDHHC17 | zinc finger, DHHC-type containing 17 | -1.681631621 | 1.681631621 | down | 0.022773469 | 0.551784312 |
| CYP2D6 | cytochrome P450, family 2, subfamily D, polypeptide 6 | -1.681732644 | 1.681732644 | down | 0.028916347 | 0.574350948 |

| | | | | | | |
|---|---|---|---|---|---|---|
| LOC101929829 | cytochrome P450, family 2, subfamily D, polypeptide 6 pseudogene | -1.681732644 | 1.681732644 | down | 0.028916347 | 0.574350948 |
| CYP2D7 | cytochrome P450, family 2, subfamily D, polypeptide 7 (gene/pseudogene) | -1.681732644 | 1.681732644 | down | 0.028916347 | 0.574350948 |
| ARHGEF28 | Rho guanine nucleotide exchange factor 28 | -1.682218417 | 1.682218417 | down | 0.021971814 | 0.550085548 |
| SRGN | serglycin | -1.68227672 | 1.68227672 | down | 9.0403E-05 | 0.059972367 |
| HIST1H3F | histone cluster 1, H3f | -1.682576036 | 1.682576036 | down | 0.022535574 | 0.551250506 |
| ZSCAN12 | zinc finger and SCAN domain containing 12 | -1.682723771 | 1.682723771 | down | 0.011346056 | 0.491891526 |
| UBE3A | ubiquitin protein ligase E3A | -1.684201828 | 1.684201828 | down | 0.009938289 | 0.478589231 |
| ZDHHC19 | zinc finger, DHHC-type containing 19 | -1.684291331 | 1.684291331 | down | 0.025668556 | 0.566329497 |
| TMPRSS4 | transmembrane protease, serine 4 | -1.684707777 | 1.684707777 | down | 0.005564036 | 0.401886731 |
| LPIN1 | lipin 1 | -1.68472724 | 1.68472724 | down | 0.002282903 | 0.295613397 |
| PKD1L2 | polycystic kidney disease 1-like 2 (gene/pseudogene) | -1.686160306 | 1.686160306 | down | 0.038513584 | 0.61336314 |
| SPDYE2B | speedy/RINGO cell cycle regulator family member E2B | -1.686238225 | 1.686238225 | down | 0.002162144 | 0.288675084 |
| RSPO1 | R-spondin 1 | -1.686347318 | 1.686347318 | down | 0.029044463 | 0.574540338 |
| TAF8 | TATA-box binding protein associated factor 8 | -1.68652266 | 1.68652266 | down | 0.023945342 | 0.562080133 |
| LST1 | leukocyte specific transcript 1 | -1.687044898 | 1.687044898 | down | 0.040674251 | 0.623259064 |
| ANXA5 | annexin A5 | -1.687103367 | 1.687103367 | down | 0.014718222 | 0.515525808 |
| TNFRSF1B | tumor necrosis factor receptor superfamily member 1B | -1.68711896 | 1.68711896 | down | 0.011787883 | 0.495320532 |
| H3F3B | H3 histone, family 3B (H3.3B) | -1.687407442 | 1.687407442 | down | 0.000536361 | 0.163673127 |
| PLAGL1 | pleiomorphic adenoma gene-like 1 | -1.687789561 | 1.687789561 | down | 0.044411186 | 0.623333169 |
| MAP1B | microtubule associated protein 1B | -1.688265382 | 1.688265382 | down | 0.007163201 | 0.434730278 |
| SLC35G4 | solute carrier family 35, member G4 | -1.688280985 | 1.688280985 | down | 0.007987865 | 0.449845949 |
| SLC35G5 | solute carrier family 35 member G5 | -1.688280985 | 1.688280985 | down | 0.007987865 | 0.449845949 |
| TMEM133 | transmembrane protein 133 | -1.688444825 | 1.688444825 | down | 0.000179146 | 0.089266768 |
| TRMT2B | tRNA methyltransferase 2 homolog B | -1.688468232 | 1.688468232 | down | 0.001236068 | 0.240074176 |
| FAM187B | family with sequence similarity 187 member B | -1.688678909 | 1.688678909 | down | 0.027265155 | 0.568317266 |
| SEBOX | SEBOX homeobox | -1.688753043 | 1.688753043 | down | 0.036651466 | 0.604603394 |
| ZBTB44 | zinc finger and BTB domain containing 44 | -1.689279875 | 1.689279875 | down | 0.034410128 | 0.596656508 |
| SYT4 | synaptotagmin 4 | -1.689564822 | 1.689564822 | down | 0.04702339 | 0.627322764 |
| LOC100506403 | uncharacterized LOC100506403 | -1.689924003 | 1.689924003 | down | 0.010591468 | 0.486801343 |
| RUNX1 | runt-related transcription factor 1 | -1.689924003 | 1.689924003 | down | 0.010591468 | 0.486801343 |
| LINC01270 | long intergenic non-protein coding RNA 1270 | -1.689966954 | 1.689966954 | down | 0.033484814 | 0.593014207 |
| ANO1 | anoctamin 1, calcium activated chloride channel | -1.690111432 | 1.690111432 | down | 0.000286805 | 0.123163556 |
| CH17-351M24.1 | uncharacterized LOC644767 | -1.690298882 | 1.690298882 | down | 0.013113154 | 0.508746116 |
| HNRNPK | heterogeneous nuclear ribonucleoprotein K | -1.69042386 | 1.69042386 | down | 0.005803949 | 0.409879029 |
| TCERG1L | transcription elongation regulator 1 like | -1.690638687 | 1.690638687 | down | 0.029419291 | 0.576629742 |
| GZF1 | GDNF-inducible zinc finger protein 1 | -1.690748065 | 1.690748065 | down | 0.013276324 | 0.509292857 |
| MTDH | metadherin | -1.690935585 | 1.690935585 | down | 0.006070049 | 0.414619018 |
| UCHL1 | ubiquitin C-terminal hydrolase L1 | -1.691279426 | 1.691279426 | down | 0.032894384 | 0.590894404 |
| LZTS1 | leucine zipper, putative tumor suppressor 1 | -1.691932136 | 1.691932136 | down | 0.026897086 | 0.568317266 |
| IGF1 | insulin like growth factor 1 | -1.692761088 | 1.692761088 | down | 0.041881276 | 0.623333169 |
| TMEM53 | transmembrane protein 53 | -1.692901894 | 1.692901894 | down | 0.01670342 | 0.528561191 |
| ZFP69 | ZFP69 zinc finger protein | -1.693539577 | 1.693539577 | down | 0.028357687 | 0.57421459 |
| BTNL9 | butyrophilin-like 9 | -1.694216646 | 1.694216646 | down | 0.042508105 | 0.623333169 |
| MED24 | mediator complex subunit 24 | -1.69445153 | 1.69445153 | down | 0.016836014 | 0.530351036 |
| FAAP100 | Fanconi anemia core complex associated protein 100 | -1.694690363 | 1.694690363 | down | 0.020579365 | 0.549146 |
| CELP | carboxyl ester lipase pseudogene | -1.694901817 | 1.694901817 | down | 0.047692724 | 0.627854743 |
| SEPT6 | septin 6 | -1.697186415 | 1.697186415 | down | 0.026949307 | 0.568317266 |
| RAD1 | RAD1 checkpoint DNA exonuclease | -1.697260922 | 1.697260922 | down | 0.013922637 | 0.511949108 |
| CLDN2 | claudin 2 | -1.697300137 | 1.697300137 | down | 0.017734132 | 0.532517378 |
| VAV3 | vav guanine nucleotide exchange factor 3 | -1.69739818 | 1.69739818 | down | 0.015321266 | 0.520381065 |
| TBC1D23 | TBC1 domain family member 23 | -1.697864941 | 1.697864941 | down | 0.001192955 | 0.237175216 |
| PDZD2 | PDZ domain containing 2 | -1.697951247 | 1.697951247 | down | 0.010069374 | 0.480015301 |
| EIF5 | eukaryotic translation initiation factor 5 | -1.699124659 | 1.699124659 | down | 0.040720111 | 0.623259064 |
| FUK | fucokinase | -1.699489799 | 1.699489799 | down | 0.009106089 | 0.466623412 |

| | | | | | | |
|---|---|---|---|---|---|---|
| LAMA3 | laminin subunit alpha 3 | -1.699623311 | 1.699623311 | down | 1.34921E-05 | 0.01517475 |
| CTSC | cathepsin C | -1.700499249 | 1.700499249 | down | 0.020861716 | 0.549146 |
| GPRIN3 | GPRIN family member 3 | -1.700754653 | 1.700754653 | down | 0.039797867 | 0.61971639 |
| ADAMTSL1 | ADAMTS like 1 | -1.700939353 | 1.700939353 | down | 0.022185888 | 0.550353494 |
| TOR1AIP2 | torsin A interacting protein 2 | -1.701124073 | 1.701124073 | down | 0.002673213 | 0.315760691 |
| AHNAK2 | AHNAK nucleoprotein 2 | -1.701230198 | 1.701230198 | down | 0.014490568 | 0.515525808 |
| CCDC163P | coiled-coil domain containing 163, pseudogene | -1.701324537 | 1.701324537 | down | 0.013904403 | 0.511949108 |
| S100A9 | S100 calcium binding protein A9 | -1.701583996 | 1.701583996 | down | 0.000405568 | 0.144894892 |
| MTFMT | mitochondrial methionyl-tRNA formyltransferase | -1.70192607 | 1.70192607 | down | 0.021665428 | 0.550085548 |
| MED13 | mediator complex subunit 13 | -1.703058945 | 1.703058945 | down | 0.00100368 | 0.218262407 |
| RECK | reversion-inducing-cysteine-rich protein with kazal motifs | -1.704074452 | 1.704074452 | down | 0.035385253 | 0.600698736 |
| ORAOV1 | oral cancer overexpressed 1 | -1.704283139 | 1.704283139 | down | 0.041324165 | 0.623333169 |
| LAMB3 | laminin subunit beta 3 | -1.704503666 | 1.704503666 | down | 3.81348E-06 | 0.006254904 |
| C10orf12 | chromosome 10 open reading frame 12 | -1.705133901 | 1.705133901 | down | 0.01121595 | 0.489992559 |
| HECW2 | HECT, C2 and WW domain containing E3 ubiquitin protein ligase 2 | -1.705421523 | 1.705421523 | down | 0.012455671 | 0.503406041 |
| ABCC4 | ATP binding cassette subfamily C member 4 | -1.706182182 | 1.706182182 | down | 0.002713182 | 0.315798154 |
| MTCH1 | mitochondrial carrier 1 | -1.706367472 | 1.706367472 | down | 0.024293745 | 0.563333857 |
| PRSS45 | protease, serine 45 | -1.706722338 | 1.706722338 | down | 0.038703891 | 0.613615561 |
| SEMA6D | sema domain, transmembrane domain (TM), and cytoplasmic domain, (semaphorin) 6D | -1.707850513 | 1.707850513 | down | 0.022529094 | 0.551250506 |
| KCNG4 | potassium channel, voltage gated modifier subfamily G, member 4 | -1.708095181 | 1.708095181 | down | 0.043232677 | 0.623333169 |
| HMBOX1 | homeobox containing 1 | -1.70814254 | 1.70814254 | down | 0.036677071 | 0.604603394 |
| AMIGO1 | adhesion molecule with Ig-like domain 1 | -1.709058408 | 1.709058408 | down | 0.02345077 | 0.556778981 |
| EHD2 | EH domain containing 2 | -1.709970815 | 1.709970815 | down | 0.003889604 | 0.353492125 |
| ZCCHC13 | zinc finger, CCHC domain containing 13 | -1.710943006 | 1.710943006 | down | 0.016599467 | 0.526872128 |
| ATP6V1C2 | ATPase, H+ transporting, lysosomal 42kDa, V1 subunit C2 | -1.711105092 | 1.711105092 | down | 0.017340017 | 0.531030242 |
| FOXL2 | forkhead box L2 | -1.712129353 | 1.712129353 | down | 0.019619904 | 0.546981245 |
| GBF1 | golgi brefeldin A resistant guanine nucleotide exchange factor 1 | -1.712224296 | 1.712224296 | down | 0.015757194 | 0.522063677 |
| GRASP | GRP1 (general receptor for phosphoinositides 1)-associated scaffold protein | -1.71274262 | 1.71274262 | down | 0.004240514 | 0.362099428 |
| FRAT1 | frequently rearranged in advanced T-cell lymphomas 1 | -1.712762407 | 1.712762407 | down | 0.026706381 | 0.568317266 |
| RIMS2 | regulating synaptic membrane exocytosis 2 | -1.713862896 | 1.713862896 | down | 0.005276254 | 0.396373556 |
| LAMC2 | laminin subunit gamma 2 | -1.714373794 | 1.714373794 | down | 0.006138313 | 0.414771056 |
| LIN54 | lin-54 DREAM MuvB core complex component | -1.715293003 | 1.715293003 | down | 0.033920452 | 0.594514074 |
| RIPK1 | receptor interacting serine/threonine kinase 1 | -1.715574411 | 1.715574411 | down | 0.003042843 | 0.326391956 |
| RASGRF2 | Ras protein specific guanine nucleotide releasing factor 2 | -1.717236055 | 1.717236055 | down | 0.018214853 | 0.536306821 |
| ABLIM1 | actin binding LIM protein 1 | -1.717497941 | 1.717497941 | down | 0.006175818 | 0.414771056 |
| NR1D1 | nuclear receptor subfamily 1 group D member 1 | -1.717755897 | 1.717755897 | down | 0.040696863 | 0.623259064 |
| THRA | thyroid hormone receptor, alpha | -1.717755897 | 1.717755897 | down | 0.040696863 | 0.623259064 |
| GAN | gigaxonin | -1.718073435 | 1.718073435 | down | 0.002548846 | 0.309987919 |
| IL23A | interleukin 23, alpha subunit p19 | -1.718823852 | 1.718823852 | down | 0.001252626 | 0.240074176 |
| KIAA1462 | KIAA1462 | -1.719089951 | 1.719089951 | down | 0.017469118 | 0.531030242 |
| ABCA1 | ATP binding cassette subfamily A member 1 | -1.719264726 | 1.719264726 | down | 0.024546573 | 0.563613185 |
| ZNF674 | zinc finger protein 674 | -1.719701739 | 1.719701739 | down | 0.033743866 | 0.593014207 |
| KRBOX4 | KRAB box domain containing 4 | -1.719701739 | 1.719701739 | down | 0.033743866 | 0.593014207 |
| HOXA4 | homeobox A4 | -1.720313745 | 1.720313745 | down | 0.01913876 | 0.541887884 |
| RAD52 | RAD52 homolog, DNA repair protein | -1.721271931 | 1.721271931 | down | 0.010672442 | 0.487945041 |
| LHX1 | LIM homeobox 1 | -1.721387267 | 1.721387267 | down | 0.00031181 | 0.127858332 |
| HIST1H1D | histone cluster 1, H1d | -1.723090372 | 1.723090372 | down | 0.011209625 | 0.489992559 |
| TALDO1 | transaldolase 1 | -1.724384742 | 1.724384742 | down | 0.045176838 | 0.624555255 |
| HCN3 | hyperpolarization activated cyclic nucleotide gated potassium channel 3 | -1.725125958 | 1.725125958 | down | 0.017515598 | 0.531030242 |
| FSTL3 | follistatin-like 3 (secreted glycoprotein) | -1.725137916 | 1.725137916 | down | 0.010634129 | 0.486801343 |
| INSIG1 | insulin induced gene 1 | -1.725221622 | 1.725221622 | down | 0.000479783 | 0.154312327 |
| VIM | vimentin | -1.72641388 | 1.72641388 | down | 5.23891E-06 | 0.007931918 |
| BMPER | BMP binding endothelial regulator | -1.72648967 | 1.72648967 | down | 0.013953243 | 0.511949108 |
| KRT6B | keratin 6B, type II | -1.726673176 | 1.726673176 | down | 3.07482E-05 | 0.02881915 |

| | | | | | | |
|---|---|---|---|---|---|---|
| KRT6C | keratin 6C, type II | -1.726673176 | 1.726673176 | down | 3.07482E-05 | 0.02881915 |
| FAM71C | family with sequence similarity 71 member C | -1.72694049 | 1.72694049 | down | 0.010843869 | 0.488995826 |
| UBQLNL | ubiquilin-like | -1.728417451 | 1.728417451 | down | 0.011699689 | 0.495320532 |
| CACTIN | cactin, spliceosome C complex subunit | -1.728521285 | 1.728521285 | down | 0.007350829 | 0.437407205 |
| ITGBL1 | integrin beta like 1 | -1.729795755 | 1.729795755 | down | 0.017977751 | 0.535584855 |
| TMC5 | transmembrane channel like 5 | -1.729975615 | 1.729975615 | down | 0.021597807 | 0.550085548 |
| FER | fer (fps/fes related) tyrosine kinase | -1.730031575 | 1.730031575 | down | 0.01237943 | 0.503186668 |
| HINT1 | histidine triad nucleotide binding protein 1 | -1.732043347 | 1.732043347 | down | 0.024526473 | 0.563613185 |
| CCDC107 | coiled-coil domain containing 107 | -1.732615709 | 1.732615709 | down | 0.001552191 | 0.251952305 |
| EMILIN3 | elastin microfibril interfacer 3 | -1.733184255 | 1.733184255 | down | 0.012947765 | 0.507494586 |
| FKSG29 | FKSG29 | -1.733380487 | 1.733380487 | down | 0.031328563 | 0.586144908 |
| PAPPA | pregnancy-associated plasma protein A, pappalysin 1 | -1.734365987 | 1.734365987 | down | 0.024903449 | 0.56554526 |
| UBASH3B | ubiquitin associated and SH3 domain containing B | -1.734390031 | 1.734390031 | down | 0.001063355 | 0.221476037 |
| TSC22D4 | TSC22 domain family member 4 | -1.735612685 | 1.735612685 | down | 0.015046934 | 0.516932843 |
| DAW1 | dynein assembly factor with WDR repeat domains 1 | -1.737229515 | 1.737229515 | down | 0.028805971 | 0.574350948 |
| CENPC | centromere protein C | -1.739599303 | 1.739599303 | down | 0.009083123 | 0.466623412 |
| NTRK2 | neurotrophic tyrosine kinase, receptor, type 2 | -1.7396194 | 1.7396194 | down | 0.017542327 | 0.531030242 |
| RAPGEF3 | Rap guanine nucleotide exchange factor 3 | -1.74051193 | 1.74051193 | down | 0.003998446 | 0.357261063 |
| SGK1 | serum/glucocorticoid regulated kinase 1 | -1.740821608 | 1.740821608 | down | 0.000636128 | 0.184765442 |
| NRP2 | neuropilin 2 | -1.741099158 | 1.741099158 | down | 0.008381018 | 0.4606964 |
| DEFB103B | defensin beta 103B | -1.741481366 | 1.741481366 | down | 0.019560668 | 0.546194159 |
| DEFB103A | defensin beta 103A | -1.741481366 | 1.741481366 | down | 0.019560668 | 0.546194159 |
| PRRX1 | paired related homeobox 1 | -1.741771094 | 1.741771094 | down | 0.013769371 | 0.511408446 |
| SLC30A10 | solute carrier family 30, member 10 | -1.742089047 | 1.742089047 | down | 0.042344802 | 0.623333169 |
| HIRA | histone cell cycle regulator | -1.742153449 | 1.742153449 | down | 0.013075685 | 0.508746116 |
| SLC25A35 | solute carrier family 25, member 35 | -1.744529954 | 1.744529954 | down | 0.023766964 | 0.560818064 |
| ZNF169 | zinc finger protein 169 | -1.746554549 | 1.746554549 | down | 0.043117439 | 0.623333169 |
| OAS3 | 2'-5'-oligoadenylate synthetase 3 | -1.747365853 | 1.747365853 | down | 0.008840107 | 0.466623412 |
| NXPE3 | neurexophilin and PC-esterase domain family member 3 | -1.74814522 | 1.74814522 | down | 0.033487151 | 0.593014207 |
| TSC2 | tuberous sclerosis 2 | -1.748621896 | 1.748621896 | down | 0.025065634 | 0.565551322 |
| GPC2 | glypican 2 | -1.751282352 | 1.751282352 | down | 0.013903134 | 0.511949108 |
| FMNL2 | formin like 2 | -1.752294225 | 1.752294225 | down | 0.02977225 | 0.578757844 |
| ATM | ATM serine/threonine kinase | -1.752840879 | 1.752840879 | down | 0.007286479 | 0.437407205 |
| ETV7 | ets variant 7 | -1.75487512 | 1.75487512 | down | 0.007389198 | 0.437407205 |
| ZNF451 | zinc finger protein 451 | -1.755572653 | 1.755572653 | down | 0.000406421 | 0.144894892 |
| S1PR1 | sphingosine-1-phosphate receptor 1 | -1.757435452 | 1.757435452 | down | 0.000832386 | 0.199221133 |
| ELK3 | ELK3, ETS-domain protein (SRF accessory protein 2) | -1.75774408 | 1.75774408 | down | 0.022671779 | 0.551250506 |
| CSTF2T | cleavage stimulation factor, 3' pre-RNA, subunit 2, tau variant | -1.758341186 | 1.758341186 | down | 0.002030679 | 0.281470725 |
| ZNF182 | zinc finger protein 182 | -1.758487446 | 1.758487446 | down | 0.048024149 | 0.627854743 |
| IL1R2 | interleukin 1 receptor, type II | -1.758686543 | 1.758686543 | down | 3.79073E-05 | 0.03174936 |
| SLC22A11 | solute carrier family 22 (organic anion/urate transporter), member 11 | -1.759166093 | 1.759166093 | down | 0.040563047 | 0.623005985 |
| ERVK-4 | endogenous retrovirus group K, member 4 | -1.759544136 | 1.759544136 | down | 0.020772866 | 0.549146 |
| MDM4 | MDM4, p53 regulator | -1.759592921 | 1.759592921 | down | 0.04690922 | 0.627322764 |
| BCAP29 | B-cell receptor-associated protein 29 | -1.759723023 | 1.759723023 | down | 0.004542286 | 0.373292465 |
| TBKBP1 | TBK1 binding protein 1 | -1.75999952 | 1.75999952 | down | 0.005378971 | 0.401028801 |
| APOA5 | apolipoprotein A-V | -1.764918669 | 1.764918669 | down | 0.00752849 | 0.441010426 |
| MMP1 | matrix metallopeptidase 1 | -1.765718104 | 1.765718104 | down | 0.015015063 | 0.516932843 |
| KRT6A | keratin 6A, type II | -1.766330161 | 1.766330161 | down | 0.000106223 | 0.064987588 |
| ERVK-6 | endogenous retrovirus group K, member 6 | -1.770182824 | 1.770182824 | down | 0.020014934 | 0.549146 |
| CTBP1-AS2 | CTBP1 antisense RNA 2 (head to head) | -1.772609851 | 1.772609851 | down | 0.007011794 | 0.432539346 |
| ALCAM | activated leukocyte cell adhesion molecule | -1.774900769 | 1.774900769 | down | 0.009801215 | 0.477233577 |
| RAD23B | RAD23 homolog B, nucleotide excision repair protein | -1.778377625 | 1.778377625 | down | 0.043218026 | 0.623333169 |
| HBEGF | heparin-binding EGF-like growth factor | -1.778994071 | 1.778994071 | down | 0.005794632 | 0.409879029 |
| MFAP5 | microfibrillar associated protein 5 | -1.780519664 | 1.780519664 | down | 0.00027954 | 0.122267645 |

| | | | | | | |
|---|---|---|---|---|---|---|
| KCND2 | potassium channel, voltage gated Shal related subfamily D, member 2 | -1.781169775 | 1.781169775 | down | 0.012668162 | 0.50525046 |
| ATAD3C | ATPase family, AAA domain containing 3C | -1.782347162 | 1.782347162 | down | 0.004327665 | 0.364793404 |
| NHLRC1 | NHL repeat containing E3 ubiquitin protein ligase 1 | -1.782623095 | 1.782623095 | down | 0.005524612 | 0.401886731 |
| ZDHHC22 | zinc finger, DHHC-type containing 22 | -1.782635452 | 1.782635452 | down | 0.044804063 | 0.623333169 |
| KISS1 | KiSS-1 metastasis-suppressor | -1.784139434 | 1.784139434 | down | 0.002560129 | 0.310090651 |
| NUFIP2 | nuclear fragile X mental retardation protein interacting protein 2 | -1.785504416 | 1.785504416 | down | 0.000752325 | 0.195840366 |
| AGER | advanced glycosylation end product-specific receptor | -1.786808516 | 1.786808516 | down | 0.010503862 | 0.486801343 |
| ZNF689 | zinc finger protein 689 | -1.786928244 | 1.786928244 | down | 0.003552702 | 0.34616393 |
| ZNF768 | zinc finger protein 768 | -1.788187935 | 1.788187935 | down | 0.003710759 | 0.349787905 |
| GTF2H4 | general transcription factor IIH subunit 4 | -1.788869778 | 1.788869778 | down | 0.022019621 | 0.550085548 |
| HCK | HCK proto-oncogene, Src family tyrosine kinase | -1.792982867 | 1.792982867 | down | 0.017470885 | 0.531030242 |
| UPF3A | UPF3 regulator of nonsense transcripts homolog A (yeast) | -1.793069865 | 1.793069865 | down | 0.00635643 | 0.417034806 |
| NCAPG | non-SMC condensin I complex subunit G | -1.793596087 | 1.793596087 | down | 0.005110288 | 0.392367316 |
| TNFRSF8 | tumor necrosis factor receptor superfamily member 8 | -1.793977383 | 1.793977383 | down | 0.005881301 | 0.411220967 |
| COG5 | component of oligomeric golgi complex 5 | -1.7942717 | 1.7942717 | down | 0.022651155 | 0.551250506 |
| ANKRD16 | ankyrin repeat domain 16 | -1.796619672 | 1.796619672 | down | 0.021147581 | 0.549146 |
| ATXN7L3 | ataxin 7-like 3 | -1.797225831 | 1.797225831 | down | 0.002097261 | 0.287025087 |
| SPANXC | SPANX family member C | -1.797906966 | 1.797906966 | down | 0.014799535 | 0.515560792 |
| SPANXD | SPANX family member D | -1.797906966 | 1.797906966 | down | 0.014799535 | 0.515560792 |
| SCEL | sciellin | -1.802369788 | 1.802369788 | down | 0.00565172 | 0.402842701 |
| XPR1 | xenotropic and polytropic retrovirus receptor 1 | -1.80312786 | 1.80312786 | down | 0.007718298 | 0.44458454 |
| ATP4A | ATPase, H+/K+ exchanging, alpha polypeptide | -1.806084053 | 1.806084053 | down | 0.003921513 | 0.354060426 |
| ADAM8 | ADAM metallopeptidase domain 8 | -1.809442215 | 1.809442215 | down | 0.002117675 | 0.288450795 |
| MRPL30 | mitochondrial ribosomal protein L30 | -1.809818517 | 1.809818517 | down | 0.000104422 | 0.064987588 |
| INHBE | inhibin beta E | -1.814470208 | 1.814470208 | down | 0.005008107 | 0.38925276 |
| COL13A1 | collagen, type XIII, alpha 1 | -1.819478541 | 1.819478541 | down | 0.002386652 | 0.300161496 |
| TAB3 | TGF-beta activated kinase 1/MAP3K7 binding protein 3 | -1.822676302 | 1.822676302 | down | 0.001483712 | 0.250769966 |
| CD44 | CD44 molecule (Indian blood group) | -1.82469462 | 1.82469462 | down | 6.14447E-05 | 0.047426848 |
| IL1A | interleukin 1 alpha | -1.826381776 | 1.826381776 | down | 0.003030825 | 0.326391956 |
| ZNF609 | zinc finger protein 609 | -1.82755949 | 1.82755949 | down | 0.004211545 | 0.361725211 |
| KIF5A | kinesin family member 5A | -1.828484465 | 1.828484465 | down | 0.004712566 | 0.377936677 |
| CFDP1 | craniofacial development protein 1 | -1.834552726 | 1.834552726 | down | 0.004978135 | 0.388818027 |
| MMP7 | matrix metallopeptidase 7 | -1.834662936 | 1.834662936 | down | 0.017824814 | 0.533998325 |
| C1orf56 | chromosome 1 open reading frame 56 | -1.838154929 | 1.838154929 | down | 0.037750238 | 0.610579997 |
| NEK10 | NIMA-related kinase 10 | -1.840543315 | 1.840543315 | down | 0.002172339 | 0.288675084 |
| GTF2H3 | general transcription factor IIH subunit 3 | -1.860940208 | 1.860940208 | down | 2.27211E-05 | 0.022933764 |
| TUBB7P | tubulin, beta 7, pseudogene | -1.864111775 | 1.864111775 | down | 0.000746395 | 0.195840366 |
| TUBB8P7 | tubulin, beta 8 class VIII pseudogene 7 | -1.864111775 | 1.864111775 | down | 0.000746395 | 0.195840366 |
| TUBBP5 | tubulin, beta pseudogene 5 | -1.864111775 | 1.864111775 | down | 0.000746395 | 0.195840366 |
| TUBB8 | tubulin, beta 8 class VIII | -1.864111775 | 1.864111775 | down | 0.000746395 | 0.195840366 |
| STRIP2 | striatin interacting protein 2 | -1.883690187 | 1.883690187 | down | 1.06243E-05 | 0.013770362 |
| SPINK6 | serine peptidase inhibitor, Kazal type 6 | -1.892585358 | 1.892585358 | down | 2.34472E-06 | 0.004570538 |
| TNN | tenascin N | -1.914279782 | 1.914279782 | down | 0.001672333 | 0.255663523 |
| RPS6KA3 | ribosomal protein S6 kinase, 90kDa, polypeptide 3 | -1.918610294 | 1.918610294 | down | 0.026067177 | 0.567622316 |
| PPP2R2C | protein phosphatase 2 regulatory subunit B, gamma | -1.92648155 | 1.92648155 | down | 0.000302622 | 0.125397133 |
| SCD | stearoyl-CoA desaturase (delta-9-desaturase) | -1.978442362 | 1.978442362 | down | 2.83632E-06 | 0.005075078 |
| GNB4 | guanine nucleotide binding protein (G protein), beta polypeptide 4 | -2.004156314 | 2.004156314 | down | 0.006701362 | 0.42771383 |
| ETS1 | v-ets avian erythroblastosis virus E26 oncogene homolog 1 | -2.017383042 | 2.017383042 | down | 0.013243848 | 0.509292857 |
| TLDC2 | TBC/LysM-associated domain containing 2 | -2.029125882 | 2.029125882 | down | 0.002403639 | 0.301335237 |
| APOL6 | apolipoprotein L, 6 | -2.099692949 | 2.099692949 | down | 3.75665E-05 | 0.03174936 |
| RAB11FIP4 | RAB11 family interacting protein 4 (class II) | -2.194859162 | 2.194859162 | down | 1.33265E-06 | 0.003085865 |
| EI24 | etoposide induced 2.4 | -2.669830901 | 2.669830901 | down | 1.42374E-11 | 1.58366E-07 |
