# Supplementary material for: EI24 Inhibits Cell Proliferation and Drug Resistance of Esophageal Squamous Cell Carcinoma
Source: Front Oncol. 2020 Aug 21;10:1570. doi: 10.3389/fonc.2020.01570 (PMC7471874; doi:10.3389/fonc.2020.01570)
Supplement: Supplementary file 3 [file Data_Sheet_3.pdf]

Table S1. Potential pathways involved in the upstream regulatory networks and downstream functions in which DEGs between EC109/EI24-OE and EC109/EI24-NC cells participate (top five).

**Regulators**	**Target Molecules in Dataset**	***Diseases & Functions***	***Consistency Score***
IL1A (interleukin 1 alpha)	APP, CCL20, CCL5, CD40, CXCL2, CXCL3, CXCL8, ICAM1, ITGAV, ITGB1, PLAU, PTGS2, S100A8, S100A9, SAA1, SERPINE1, WNT5A.	Migration of phagocytes	3.881
COL18A1 (collagen type XVIII alpha 1 chain)	EDN1, EFNA1, EGFR, F2RL1, F3, FN1, HIF1A, ITGAV, MCL1, PLAU, PTGS2, SERPINE1, STAT1, THBS1, VEGFA.	Cell proliferation of tumor cell lines	3.873
hydrogen peroxide	APP, CXCL3, CXCL8, EDN1, EGFR, F2RL1, FN1, HMGB1, ICAM1, ITGA2, LCN2, MDK, PTGS2, SERPINE1, VEGFA.	Chemotaxis	3.873
hydrogen peroxide	APP, CXCL3, CXCL8, EDN1, EGFR, F2RL1, FN1, HMGB1, ICAM1, ITGA2, LCN2, MDK, PTGS2, SERPINE1, VEGFA.	Homing of cells	3.873
AGT (angiotensinogen)	CCL5, CTSS, CXCL2, CXCL3, CXCL8, FN1, HIF1A, ICAM1, IL6ST, ITGAV, ITGB1, PLAU, PTGS2, RHOA, SERPINE1, VEGFA.	Migration of phagocytes	3.75

Table S2. Potential pathways involved in the upstream regulatory networks and downstream functions in which DEGs between TE-1/EI24-KO and TE-1/EI24-NC cells participate (top five).

**Regulators**	**Target Molecules in Dataset**	***Diseases & Functions***	***Consistency Score***
CDH1, LEP, RARB	BMP7, INS, NR3C1, SPP1, SREBF1, TGFB1, VEGFA	Glomerulosclerosis	2.268
NDP	BDNF, CNTF, FGF2, LIF	Growth of lesion	2
APEX1, NPM1, SAFB	B2M, CSF1, CXCL2, FTH1, HEXA, HLA-C, IGF1, IL10, IL6ST, PSMB9, TP53, VEGFA	Systemic autoimmune syndrome	0.866
CDH1, WISP2	BIRC5, CD44, CTNNB1, EGFR, ERBB3, HIF1A, IFITM1, LAMB3, SERPINE1, SOX9, VEGFA, VIM	Renal Cancer and Tumors	0.866
GNA12, miR-199a-3p	BRAF, CD44, EGFR, EZR, ITGA6, MET, PSMB5, VEGFA	Progression of tumor	0.707

Abbreviation: cadherin 1 (CDH1), leptin (LEP), retinoic acid receptor beta (RARB), norrin cystine knot growth factor NDP (NDP), apurinic/apyrimidinic endodeoxyribonuclease 1 (APEX1), nucleophosmin 1 (NPM1), scaffold attachment factor B (SAFB), WNT1 inducible signaling pathway protein 2 (WISP2), G protein subunit alpha 12 (GNA12), microRNA-199a-3p (miR-199a-3p).
