# Supplementary material for: EI24 Inhibits Cell Proliferation and Drug Resistance of Esophageal Squamous Cell Carcinoma
Source: Front Oncol. 2020 Aug 21;10:1570. doi: 10.3389/fonc.2020.01570 (PMC7471874; doi:10.3389/fonc.2020.01570)
Supplement: Supplementary file 4 [file Data_Sheet_4.pdf]

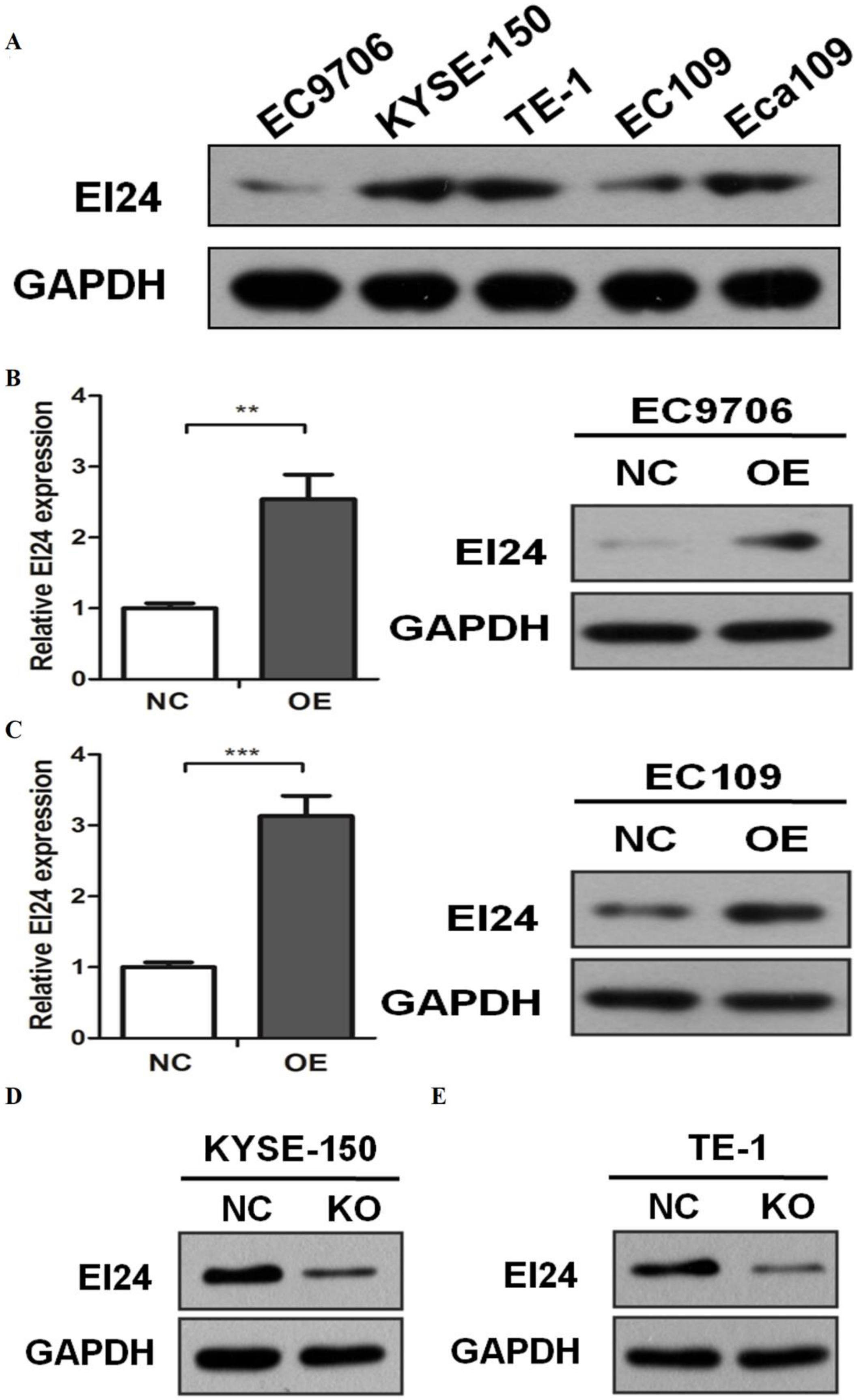

Figure S1. (A) Expression of EI24 in ESCC cell lines (EC9706, KYSE150, TE-1,

EC109, and Eca109) determined by Western blot analysis; (B and C) mRNA and protein expression following EI24 overexpression in EC9706 and EC109 cell lines examined by RT-qPCR and Western blot analysis; (D and E) Western blot analysis of EI24 protein in KYSE150/EI24-KO, KYSE150/EI24-NC, TE-1/EI24-KO and TE-1/EI24-NC cells.

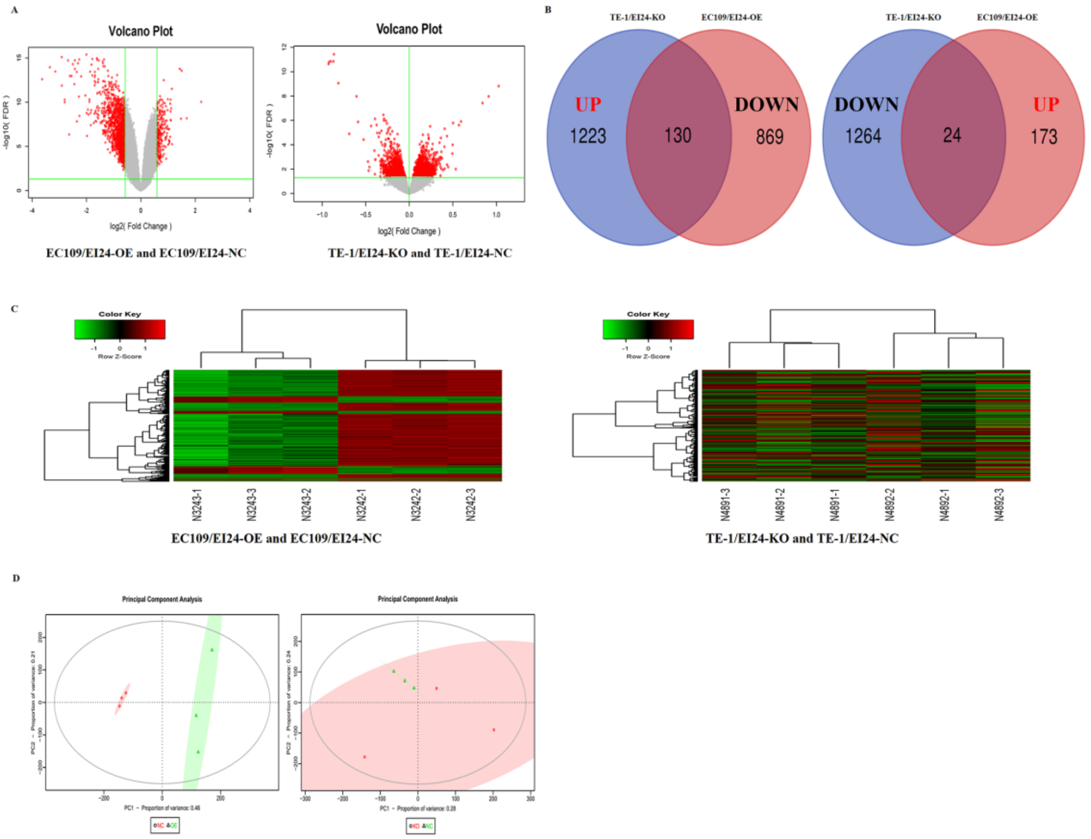

Figure S2. DEGs in EC109/EI24-OE and EC109/EI24-NC cells or in TE-1/EI24-KO and TE-1/EI24-NC cells based on microarray analysis. (A) Volcano plot: The X-axis represented the fold change values (log2-scaled), and the Y-axis indicated the false discovery rate (FDR) (log10-scaled) in EI24-OE group or the corrected P-values (log10-scaled) in EI24-NC group. The red dots in EI24-OE group represented DEGs with statistical significance (|Fold Change|>1.5 and FDR<0.05), while the red dots in EI24-KO group represented DEGs with statistical significance (P-value<0.05). Gray dots indicated no significant change in expression; (B) Venn diagram of DEGs between the EC109 cells and TE-1 cells; (C) Heatmap of the hierarchical cluster analysis. Each column represents a sample, and each row represents a gene. Above the heatmap, N3243-1, N3243-2 and N3243-3 represented the three samples of EC9706/EI24-OE cells; N3242-1, N3242-1 and N3242-1 represented the three samples of EC9706/EI24-NC cells; N4891-1, N4891-2 and N4891-3 represented the three samples of TE-1/EI24-KO cells; N4892-1, N4892-2 and N4892-3 represented the three samples of TE-1/EI24-NC cells; In the heatmap, red means upregulation, while green represents down-regulation. Color black means no difference expressed in this gene, and gray indicates that the signal intensity of gene is not detected; (D) Principal component analysis (PCA) plots generated with ggbiplot in R showing variation and clustering of samples in different groups.

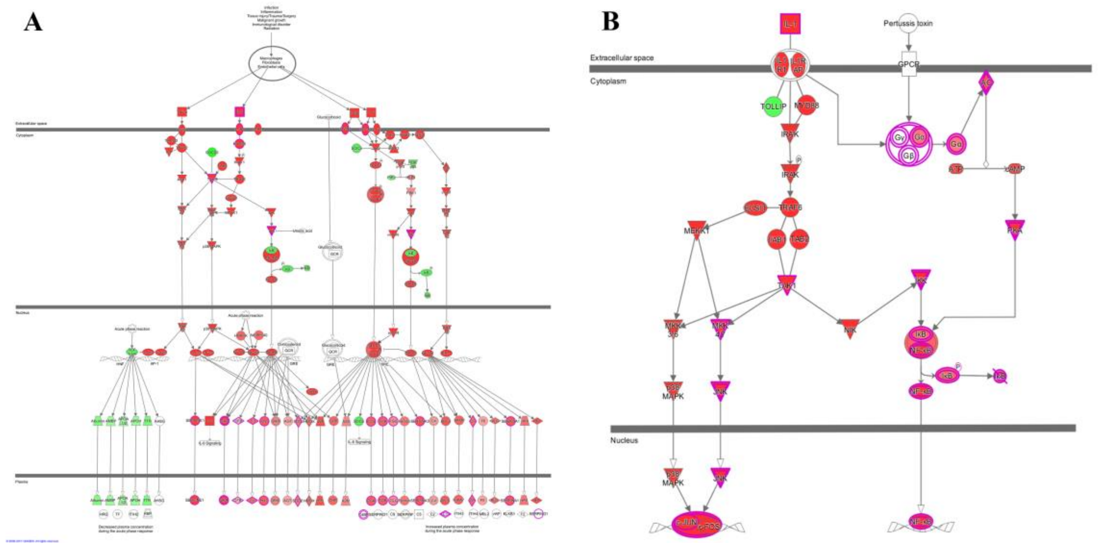

Figure S3. (A) Expression patterns of genes involving in activated Acute Phase Response Signaling Pathway were identified by literature mining; (B) Expression patterns of genes involving in activated IL-1 Signaling Pathway were identified by literature mining. Red indicated upregulation; green indicated downregulation.

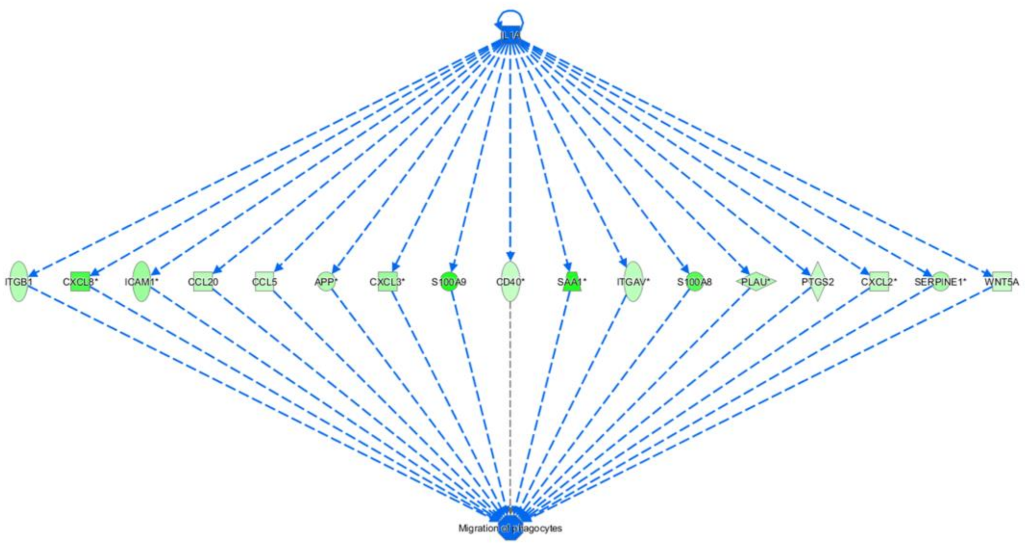

Figure S4. The top regulatory networks in regulatory effect analysis. Regulator IL1A could suppressed macrophage migration through regulating APP, CCL20, CCL5, CD40, CXCL2, CXCL3, CXCL8, ICAM1, ITGAV, ITGB1, PLAU, PTGS2, S100A8, S100A9, SAA1, SERPINE1 and WNT5A. Abbreviations: APP (amyloid beta precursor protein), CCL20 (C-C motif chemokine ligand 20), CCL5 (C-C motif chemokine ligand 5), CD40 (CD40 molecule), CXCL2 (C-X-C motif chemokine ligand 2), CXCL3 (C-X-C motif chemokine ligand 3), CXCL8 (C-X-C motif chemokine ligand 8), ICAM1 (intercellular adhesion molecule 1), ITGAV (integrin subunit alpha V), ITGB1 (integrin subunit beta 1), PLAU (plasminogen activator, urokinase), PTGS2 (prostaglandin-endoperoxide synthase 2), S100A8 (S100 calcium binding protein A8), S100A9 (S100 calcium binding protein A9), SAA1 (serum amyloid A1), SERPINE1 (serpin family E member 1) and WNT5A (Wnt family member 5A).
